# Neurobiological roots of psychopathy

**DOI:** 10.1038/s41380-019-0488-z

**Published:** 2019-08-27

**Authors:** Jari Tiihonen, Marja Koskuvi, Markku Lähteenvuo, Pekka L. J. Virtanen, Ilkka Ojansuu, Olli Vaurio, Yanyan Gao, Ida Hyötyläinen, Katja A. Puttonen, Eila Repo-Tiihonen, Tiina Paunio, Marja-Riitta Rautiainen, Sasu Tyni, Jari Koistinaho, Šárka Lehtonen

**Affiliations:** 1 Department of Forensic Psychiatry, Niuvanniemi Hospital, University of Eastern Finland, Niuvankuja 65, FI-70240 Kuopio, Finland; 2 Karolinska Institutet, Department of Clinical Neuroscience, Centre for Psychiatry Research, Stockholm County Council, Byggnad R5, SE-171 77 Stockholm, Sweden; 3 A.I. Virtanen Institute for Molecular Sciences, University of Eastern Finland, PO Box 1627, FI-70211 Kuopio, Finland; 4 Neuroscience Center, Helsinki Institute of Life Science, University of Helsinki, Haartmaninkatu 3, FI-00014 Helsinki, Finland; 5 Department of Mental Health and Substance Abuse Services and Public Health Genomics Unit, National Institute for Health and Welfare, PO Box 30, FI-00271 Helsinki, Finland; 6 Institute of Clinical Medicine, Department of Psychiatry, University of Helsinki, PO Box 22 (Välskärinkatu 12), FI-00014 Helsinki, Finland; 7 Department of Psychiatry, Helsinki University Central Hospital, PO Box 590 (Välskärinkatu 12), FI-00029 Helsinki, Finland; 8 Finnish Institute of Occupational Health, Development of Work and Work Organizations, Topeliuksenkatu 41 b, FI- 00290 Helsinki, Finland; 9 The Criminal Sanctions Agency, Lintulahdenkatu 5, FI-00530 Helsinki, Finland

**Keywords:** Psychiatric disorders, Neuroscience

## Abstract

Psychopathy is an extreme form of antisocial behavior, with about 1% prevalence in the general population, and 10–30% among incarcerated criminal offenders. Although the heritability of severe antisocial behavior is up to 50%, the genetic background is unclear. The underlying molecular mechanisms have remained unknown but several previous studies suggest that abnormal glucose metabolism and opioidergic neurotransmission contribute to violent offending and psychopathy. Here we show using iPSC-derived cortical neurons and astrocytes from six incarcerated extremely antisocial and violent offenders, three nonpsychopathic individuals with substance abuse, and six healthy controls that there are robust alterations in the expression of several genes and immune response-related molecular pathways which were specific for psychopathy. In neurons, psychopathy was associated with marked upregulation of *RPL10P9* and *ZNF132*, and downregulation of *CDH5* and *OPRD1*. In astrocytes, *RPL10P9* and *MT-RNR2* were upregulated. Expression of aforementioned genes explained 30–92% of the variance of psychopathic symptoms. The gene expression findings were confirmed with qPCR. These genes may be relevant to the lack of empathy and emotional callousness seen in psychopathy, since several studies have linked these genes to autism and social interaction.

## Introduction

In developed countries, a relatively small group of antisocial recidivistic offenders commits the majority of all violent crimes. The prevalence of antisocial personality disorder (ASPD) is 1–3% in the general population and 40–70% in prison populations, and the corresponding figures for its most severe manifestation, psychopathy, are about 1% in the general population and 10–30% among incarcerated offenders [1–5]. ASPD is characterized by aggression, hostility, callousness, manipulativeness, deceitfulness, and impulsivity, and psychopathy is an extreme manifestation of ASPD. Severe antisocial and criminal behavior has a substantial genetic component [6]. This far only one study has reported contributing genes reaching genome-wide significance for ASPD [7], although two studies have found association between single-nucleotide polymorphisms and broad spectrum of antisocial behavior [8, 9]. *LINC00951*, the gene associated with imprisoned offenders with ASPD, codes for long intergenic noncoding RNA, which is expressed especially in the frontal cortex and cerebellum. Its function is not known [7], but it has been linked to autoimmune disease [10]. The gene linked to adult antisocial behavior, *ABCB1*, is also highly expressed in the brain, and implicated in substance abuse [9]. No underlying molecular pathways of severe antisocial and criminal behavior are known, but there is preliminary evidence on dysregulation of the endogenous opioid system and brain opioid receptors [11–13] in antisocial individuals. Also abnormal glucose metabolism leading to hypoglycemia has been observed as the strongest predictor for violent crimes [14]. A recent study has also found association between immune-related gene sets and antisocial behavior [9]. We aimed to study the neurobiological background of psychopathy by using induced pluripotent stem cell (iPSC)-derived cortical neurons and astrocytes, and included also nonpsychopathic substance abusers in addition to healthy individuals as control groups in order to distinguish the putative role of the coexisting substance dependence.

## Material and methods

We generated and fully characterized iPSC lines from six antisocial violent offenders, three nonviolent substance abusers and six control subjects without antisocial traits or substance abuse disorders. Due to the explanatory nature of the study, no power analysis based on predefined effect size was done. The clinical and sociodemographic characteristics of the study subjects are shown in Table 1. We chose to differentiate the cells into cortical neurons expressing markers of glutamatergic and GABAergic neurons and to astrocytes. Methods for iPSC production and their characterization, derivation of neurons and astrocytes and their analyses are reported in detail in Supplementary Material.

**Table 1:** Clinical and sociodemographic characteristics of study subjects

	Age	Diagnosis	Number of committed homicides	Number of violent crimes	PCL-R score
Subject 1	30	Antisocial personality disorder, ADHD, alcohol dependence, benzodiazepine abuse, multiple sclerosis, asthma	2	19	37.0
Subject 2	42	Antisocial personality disorder, alcohol dependence	3	4	Not available
Subject 3	49	Antisocial personality disorder, alcohol dependence	2	11	30.0
Subject 4	43	Antisocial personality disorder, alcohol dependence, polysubstance dependence	2	7	33.7
Subject 5	30	Antisocial personality disorder, alcohol dependence, opioid dependence, cannabis dependence, benzodiazepine dependence, amphetamine dependence	3	8	36.0
Subject 6	47	Antisocial personality disorder, borderline personality disorder, paranoid personality disorder, alcohol dependence, polysubstance dependence, amphetamine dependence, hepatitis C	2	9	37.0
Subject 7	38	Alcohol dependence	0	0	2
Subject 8	25	Alcohol dependence	0	0	3
Subject 9	31	Alcohol dependence, cannabis dependence, bulimia	0	0	11
Subject 10	44	None	0	0	3
Subject 11	28	None	0	0	2
Subject 12	28	None	0	0	1
Subject 13	47	None	0	0	3
Subject 14	26	None	0	0	2
Subject 15	51	None	0	0	1

All individuals were males. Subjects 1–6 are violent offenders, 7–9 are individuals with substance abuse but without criminal behavior, and 10–15 are healthy controls. The biological fathers of Subject 1, Subject 3, and Subject 5 had prison convictions due to violent and nonviolent crimes. None of the biological mothers had been convicted into prison

*PCL-R* psychopathy checklist revised

### Description of subjects

Six male offenders were identified by the history of their criminal convictions from the Finnish National Crime Register and recruited through the penal system and classified as extremely violent offenders as described in Tiihonen et al. [5]. Three individuals having substance dependence without violent behavior were recruited from the local substance abuse rehabilitation center, and six healthy controls were recruited from the staff of Niuvanniemi Hospital. The participants were interviewed with Structured Clinical Interview for DSM-IV-Disorders to exclude individuals with a psychosis diagnosis, and to assess whether or not the subject fulfilled criteria for ASPD. Also, any history of substance abuse (alcohol, heroin, buprenorphine, amphetamine, cannabis, other) was obtained through a questionnaire. The history of criminal convictions was obtained from the National Crime Register [5]. Psychopathy ratings with the Hare Psychopathy Checklist revised (PCL-R) [1] were done by accredited rater OV using official crime register data and forensic mental examination reports (violent offenders), and clinical interview (individuals with substance dependence, healthy controls). Informed consent was obtained from all subjects. This study was approved by the Ethics Committee for Pediatrics, Adolescent Medicine and Psychiatry, Hospital District of Helsinki and Uusimaa, and the Criminal Sanctions Agency of Finland.

### Generation of hiPSCs and their characterization

The hiPSC lines were derived from individuals’ skin fibroblasts (Supplementary Table 1, Supplementary Figs. 1 and 2). The fibroblasts were isolated and expanded in fibroblast culture media containing Iscove’s DMEM media (Thermo Fisher Scientific) with 20% fetal bovine serum, 1% Penicillin–Streptomycin and 1% nonessential amino acids. iPSC reprogramming was performed by the CytoTunE−iPS 2.0 Sendai Reprogramming Kit (Thermo Fisher Scientific) according to the manufacturer’s instructions. The iPSCs were grown on Matrigel-coated dishes (BD Biosciences) in E8 medium (Gibco). Medium was changed every other day and hiPSC colonies were enzymatically passaged using 0.5 mM EDTA (Gibco). The pluripotency of hiPSCs was confirmed by expression of pluripotent markers using immunocytochemistry (Oct-4, Sox2, TRA-1-81, and SSEA4) and qPCR (*OCT-4*, *SOX-2*, *NANOG*, and *LIN-28*). The embryoid body formation assay showed hiPSCs properties to differentiate into all three germ layers. In this assay, hiPSCs were proliferated in low-adherent plates for 2 weeks after which the EBs were plated down on Matrigel-coated plates for an additional two weeks. The expression of smooth muscle actin positive cells (mesoderm), BIIITubulin-positive cells (ectoderm), and alpha-fetoprotein positive cells (endoderm) was confirmed by immunocytochemistry. The clearance of Sendai virus was measured by qPCR, and United Medix Laboratories Ltd in Helsinki (Finland) confirmed normal karyotype of each cell line.

### hiPSC differentiation to neural precursor cells (NPCs) and cortical neurons

Neural differentiation was performed according to Hicks et al. [15] with minor modifications. hiPSC colonies growing on Matrigel-coated plates are exposed to dual SMAD inhibitors (10 µM SB431542 and 200 nM LDN-193189) for 10 days in neural differentiation medium containing a 1:1 mix of DMEM/F12 and Neurobasal medium supplemented with 1% B27 supplement, 0.5% N2 supplement, 2 mM Glutamax, 50 IU/ml penicillin, and 50 μg/ml streptomycin (all from Gibco). After the induction, 25 ng/ml bFGF (R&D Systems) was added for additional 2 days to expand the differentiated neuroepithelial cells in rosettes. Rosettes were detached and plated into ultralow attachment dishes (Corning) in neural sphere medium (NSM), consisting of a 1:1 mix of DMEM/F12 and Neurobasal medium supplemented with 1% N_2_ supplement, 2 mM Glutamax, 50 IU/ml penicillin, and 50 μg/ml streptomycin (all from Gibco) supplemented with 25 ng/ml bFGF. During the differentiation, half of the medium was renewed every other day and the spheres were manually cut once a week to maintain NPC population. For experimental purposes, NPCs were dissociated with Accutase and plated in NSM media onto PORN/Matrigel-coated plates (with density 2–3 × 10^6^ cells/6 cm dish; 1 × 10^6^ cells/6-well plate or 100,000 cell/24-well plate). The neurons were matured for 1 week before experiments. Immunocytochemistry results showing the fractions of glutamatergic and GABAergic cells are presented in Supplementary Fig. 3.

### hiPSC differentiation to astrocytes

We have adapted a previously published protocol for the differentiation of hiPSC-derived astrocytes [16]. Briefly, due to the same origin of neurons and astrocytes, hiPSCs were differentiated into neuroepithelial cells by using the same procedure as for neuron differentiation for the first 10 days. The neural progenitors were detached to ultralow attachment dishes and expanded in astrocyte sphere medium, i.e., DMEM/F12 medium supplemented with 1% N_2_ supplement, 2 mM Glutamax, 50 IU/ml penicillin, and 50 μg/ml streptomycin (all from Gibco), 5000 KY/ml Heparin (LEO), 10 ng/ml bFGF and 10 ng/ml EGF (both from R&D Systems). Half of the medium was renewed every other day and the spheres were manually cut once a week. According to our experience, this method generates a homogenous population of astrocyte progenitor cells within 4 months of differentiation. The astrocytes were further maturated on Matrigel-coated plates by treatment with 10 ng/ml CNTF and 10 ng/ml BMP4 (both from PeproTech) for 1 week in a density of 600,000 cells/6-well plate or 80,000 cell/24-well plate.

Methods concerning immunocytochemistry, RNA isolation, gene expression profile, qRT-PCR, and quantitative proteomic analysis are described in detail in Supplementary Material.

## Results

**Fig. 1:**
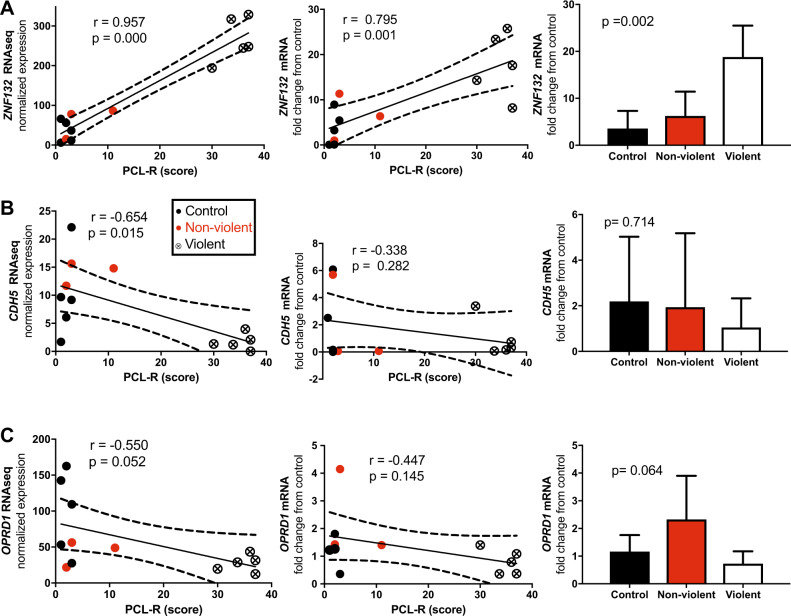
RNA expression analyses of hiPSCs-derived cortical neurons for **a** *ZNF132*, **b** *CDH5*, and **c** *OPRD1* genes. The first graph represents correlation with normalized expression levels and the second with gene expression levels validated by quantitative RT-PCR (qRT-PCR). The column graph presents mRNA expression levels of gene of interest measured by qRT-PCR. *r* indicates Pearson correlation coefficient. “Violent” indicates violent offenders, and “nonviolent” indicates individuals with substance abuse but without criminal behavior

**Fig. 2:**
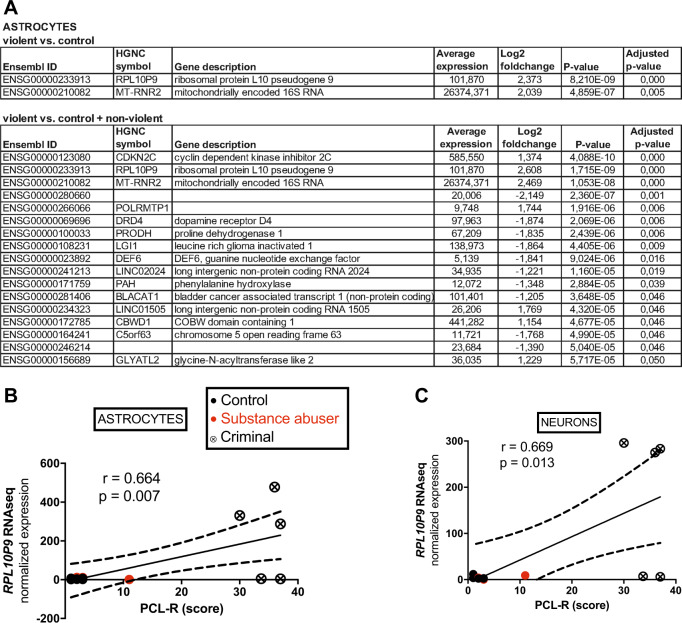
Transcriptome analyses of differentially expressed genes in hiPSCs-derived astrocytes. **a** The genes with adjusted *p*-value < 0.05 and at least twofold up- or downregulation are presented in the table. The correlation of PCL-R score with normalized expression levels for *RPL10P9* in **b** astrocytes and **c** neurons. *r* indicates the Pearson correlation coefficient. “Violent” indicates violent offenders, and “nonviolent” indicates individuals with substance abuse but without criminal behavior

Supplementary Tables 2 and 3 show all differentially expressed genes in neurons and astrocytes, respectively, up to nominal significance (*p* < 0.05) between violent offenders and control subjects. Since cultivation of neurons failed from cells of one healthy control, there were 14 individuals in the analyses concerning neurons, and 15 individuals in the analyses concerning astrocytes. Differentially expressed genes surviving correction for multiple comparisons in cortical neurons are shown in Table 2. Of these genes, in neurons, ribosomal *RPL10P9* pseudogene showed over tenfold upregulation in violent offenders as compared with healthy controls and nonviolent individuals with substance abuse. Also zinc finger protein 132 (*ZNF132*) gene was markedly upregulated, and cadherin 5 (*CDH5*) gene markedly downregulated among the neurons derived from cells of violent offenders. Figure 1 displays the qPCR replications of these results (except for *RPL10P9* due to no suitable primers being available), and correlations between gene expression levels and psychopathy score (PCL-R). Pearson’s correlations between gene expression and PCL-R score were 0.67 (*p* = 0.013, *N* = 13; see Fig. 2) for *RPL10P9*, 0.96 (*p* = 0.000, *N* = 13) for *ZNF132*, −0.65 (*p* = 0.015, *N* = 13) for *CDH5*, and −0.55 (*p* = 0.05, *N* = 13) for opioid receptor delta 1*(OPRD1)*. *CDH13* gene encoding a cadherin that regulates axon growth during neural differentiation, has been previously linked to extremely violent behavior [5], but it did not achieve statistically significance (*p* = 0.24) in this study, possibly because it is most prominently expressed by oligodendrocytes in the brain. Although the result for *RPL10P9* could not be verified with qPCR due to missing suitable primers, the same result for significant correlation with PCL-R score and upregulation of the gene in the gene array was also discovered in the astrocytes differentiated from the hiPSCs lines [Pearson’s correlation 0.66 (*p* = 0.007, *N* = 14)] (Fig. 2). Altogether, the data indicate the robustness of this finding and underline the importance of *RPL10P9* in the pathophysiology of psychopathy. In the astrocytes, also mitochondria encoded 16S RNA (*MT-RNR*) 2 showed a four- to sixfold upregulation of RNA expression in the violent offenders (Fig. 2).

**Table 2:** Transcriptome analysis of differentially expressed genes in hiPSCs-derived cortical neurons

Ensembl ID	HGNC symbol	Gene description	Average expression	Log2 fold change	*P*-value	Adjusted *p*-value
Violent vs. control						
ENSG00000233913	RPL10P9	Ribosomal protein L10 pseudogene 9	98.909	3.433	8.26E−10	4.29E−05
ENSG00000268912			79.387	2.447	3.48E−09	9.06E−05
ENSG00000131849	ZNF132	Zinc finger protein 132	138.380	2.407	4.37E−08	7.57E−04
ENSG00000256299			21.891	1.526	3.39E−06	4.40E−02
Violent vs. control + nonviolent						
ENSG00000233913	RPL10P9	Ribosomal protein L10 pseudogene 9	98.909	3.686	6.13E−10	1.84E−05
ENSG00000182397	DNM1P46	Dynamin 1 pseudogene 46	16.689	−1.595	1.70E−07	2.13E−03
ENSG00000211459	MT-RNR1	Mitochondrially encoded 12 S RNA	3236.254	−2.687	2.12E−07	2.13E−03
ENSG00000138347	MYPN	Myopalladin	7.066	−2.990	4.20E−07	3.16E−03
ENSG00000023839	ABCC2	ATP binding cassette subfamily C member 2	19.849	−1.972	1.76E−06	1.01E−02
ENSG00000128284	APOL3	Apolipoprotein L3	13.244	−2.741	2.02E−06	1.01E−02
ENSG00000235683			4.804	−2.446	5.86E−06	1.82E−02
ENSG00000140678	ITGAX	Integrin subunit alpha X	13.142	−2.747	6.24E−06	1.82E−02
ENSG00000210151	MT-TS1	Mitochondrially encoded tRNA serine 1 (UCN)	17.046	−2.165	7.14E−06	1.82E−02
ENSG00000172738	TMEM217	Transmembrane protein 217	40.619	−1.633	7.58E−06	1.82E−02
ENSG00000179776	CDH5	Cadherin 5	7.101	−2.516	8.16E−06	1.82E−02
ENSG00000260075	NSFP1		7.076	2.313	8.31E−06	1.82E−02
ENSG00000172785	CBWD1	COBW domain containing 1	320.593	1.208	8.47E−06	1.82E−02
ENSG00000204930	FAM221B	Family with sequence similarity 221 member B	7.214	−2.535	1.06E−05	2.09E−02
ENSG00000200503	SNORD115-5	Small nucleolar RNA, C/D box 115-5	28.237	2.062	1.11E−05	2.09E−02
ENSG00000225630	MTND2P28	Mitochondrially encoded NADH:ubiq.	86.559	−2.017	1.36E−05	2.24E−02
ENSG00000236064			9.175	−2.653	1.46E−05	2.24E−02
ENSG00000253426			5.636	−2.501	1.49E−05	2.24E−02
ENSG00000164659	KIAA1324L	KIAA1324 like	1178.161	1.248	1.56E−05	2.24E−02
ENSG00000267334			6.711	2.063	1.56E−05	2.24E−02
ENSG00000188585	CLEC20A	C-type lectin domain containing 20A	3.726	−2.730	1.70E−05	2.33E−02
ENSG00000013588	GPRC5A	G protein-coupled receptor class C group 5 member A	19.264	−2.356	2.02E−05	2.56E−02
ENSG00000257335	MGAM	MaltasE−glucoamylase	42.608	−1.951	2.05E−05	2.56E−02
ENSG00000198899	MT-ATP6	Mitochondrially encoded ATP synthase 6	629.862	−1.705	2.53E−05	3.04E−02
ENSG00000131042	LILRB2	Leukocyte immunoglobulin like receptor B2	2.933	−2.720	2.95E−05	3.41E−02
ENSG00000279301	OR2T11	Olfactory receptor family 2 subfamily T member 11	4.182	−2.634	3.13E−05	3.46E−02
ENSG00000268416			5.112	−2.644	3.22E−05	3.46E−02
ENSG00000268912			79.387	1.852	3.47E−05	3.59E−02
ENSG00000252906	SCARNA3	Small Cajal body-specific RNA 3	35.106	1.208	3.58E−05	3.59E−02
ENSG00000124731	TREM1	Triggering receptor expressed on myeloid cells 1	5.602	−2.372	4.35E−05	4.10E−02
ENSG00000101440	ASIP	Agouti signaling protein	3.816	−2.555	4.36E−05	4.10E−02
ENSG00000187812			7.086	−2.614	4.61E−05	4.20E−02
ENSG00000131849	ZNF132	Zinc finger protein 132	138.380	1.920	5.01E−05	4.43E−02
ENSG00000060709	RIMBP2	RIMS binding protein 2	152.792	1.619	5.68E−05	4.88E−02

Only genes with adjusted *p*-value < 0.05 and at least twofold up- or downregulation are presented

“Violent” indicates violent offenders, and “nonviolent” indicates individuals with substance abuse but without criminal behavior

Interestingly, opioid receptor delta 1 (*OPRD1*) gene was upregulated in nonviolent offenders as compared with normal controls, but the expression for *OPRD1* was even lower in the violent offenders as compared with the two other groups, although these results reached only borderline statistical significance (Fig. 1c, Suppl. Table 2). As the *OPRD1* gene codes for an opioid receptor delta protein, a protein involved in mediating the effects of opioids often used for substance abuse purposes, and as many violent antisocial offenders often suffer from substance abuse disorders, it was striking that the expression level of this protein was low in the violent offenders. Thus, we did further qPCR analysis for *OPRD1* in order to see whether differences between the groups arise in this more accurate analysis. The qPCR analysis pointed toward the same trend of lower expression of *OPRD1* in the violent offender group, although this result again reached only borderline statistical significance. Taking all together, *ZNF132*, *RPL10P9*, *CDH5*, and *OPRD1* genes explained 30–92% of variance of the psychopathy symptomatology, as measured by the PCL-score. Moreover, in fibroblasts, no differences between the studied groups were detected (Suppl. Fig. 4). These findings point out that *ZNF132* is mainly overexpressed in neurons and most likely affect the transcriptional regulation of other genes. Results from pathway analyses from neurons are displayed in Supplementary Table 4, and show enrichment in several immune response-related pathways. No statistically significant enrichments were observed in astrocytes.

**Fig. 3:**
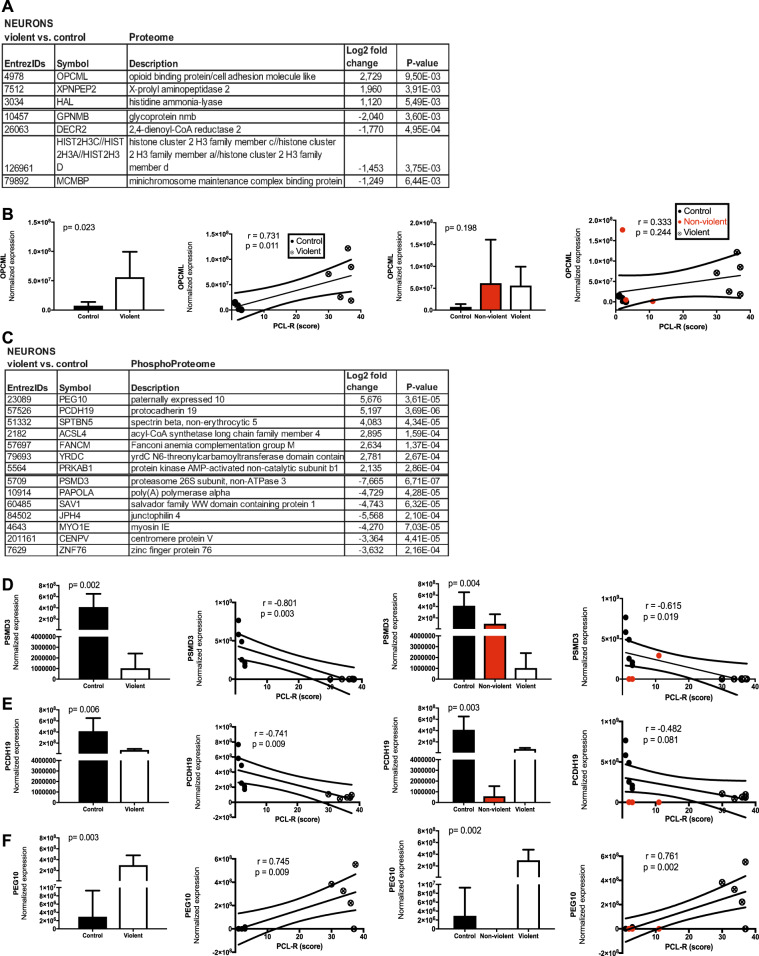
Proteomic analyses of differentially expressed proteins and phosphoproteins in hiPSCs-derived cortical neurons. **a** Proteins with adjusted *p*-value < 0.05 and at least twofold up- or downregulation are presented in the table. **b** The normalized expression of opioid-binding protein/cell-adhesion molecule (OPCML) and its correlation with PCL-R score. **c** Top ten list of phosphoproteins and normalized expression and its correlation with PCL-R score for **d** 26S proteasome non-ATPase regulatory subunit 3 (PSMD3), **e** Protocadherin 19 (PCDH19), **f** Retrotransposon-derived protein (PEG10). *p*-values shown in **a** and **c** are nominal values, and remained statistically significant (*p* < 0.05) after correction for multiple comparisons in **c**. r indicates the Pearson correlation coefficient

Data from proteomic analysis are shown in Fig. 3. Here the largest effect sizes were observed for opioid-binding protein/cell-adhesion molecule (OPCML) in the proteomic analysis and for PSMD3, PEG10, and PCDH19 in phosphoproteome analysis. Of the proteins with significantly higher levels in the proteome analysis, OPCML was the most elevated protein in the violent offenders as compared with controls (6.6-fold change, *p* = 9.5 × 10^−3^). However, also nonviolent substance abusers showed higher OPCML values than controls, indicating that this finding may not be necessarily specific for psychopathy but could be associated with substance dependence.

In the phosphoproteome analysis, in the violent criminals, paternally expressed 10 (PEG10) levels were 51-fold, protocadherin 19 (PCDH19) 37-fold, spectrin beta, nonerythrocytic 5 (SPTBN5) 17-fold, and acyl-CoA synthetase long chain family member 4 (ACSL4) 7-fold higher than controls. Concerning phosphoproteins with lower levels in the violent offenders, levels of a proteasome 26S subunit non-ATPase 3 (PSMD3) were 203-fold, and Myosin 1E (MYO1e) 19-fold lower than in controls.

## Discussion

To our knowledge, this is the first study to reveal significant alterations in gene expression related to psychopathy. Our results showed that expression levels of *RPL109*, *ZNF132*, *CDH5*, and *OPRD1* genes in neurons explained 30–92% of the severity of psychopathy, and *RPL109* expression was significantly associated with degree of psychopathy also in astrocytes. It is remarkable that all the aforementioned genes except *OPRD1* have been previously linked to autism [17–22], and might thus contribute to the emotional callousness and lack of empathy observed in psychopathic violent offenders. The strongest association was observed for *ZNF132*, a member of zinc finger Kröppel family associated with several developmental and malignant disorders [23]. It has been also reported that autism gene *CHD8* modifies the expression of *ZNF132* [18]. The exact function of *ZNF132* is unknown but it may be involved in transcriptional regulation. Interestingly, the highest expression levels of *ZNF132* mRNA were seen in cortical neurons of violent subjects, while in hiPSCs, no difference between violent and nonviolent subjects was observed. *ZNF132* is expressed highly in the cerebellum [24], and a recent study has found that cerebellum can regulate social behavior by controlling dopamine release [25], suggesting that this may contribute to mental disorders, such as autism and schizophrenia. Our results imply that cerebellum may also have a role in severe antisocial behavior.

We observed enrichment in several immune response-related pathways. This is an interesting finding since a recent study on adult antisocial behavior found enrichment in 7 gene sets, most of which being immune related [9]. This suggests that altered immune response contributes to the pathophysiology of antisocial behavior.

In proteomic analysis, the most robust finding was upregulation of OPCML. It has been shown to have an accessory role in opioid receptor function, and the gene encoding the protein is highly conserved in mammals. In rats, the accessory role to activate opioid receptors has been shown to be specific for the mu receptor ligands. Differences in *OPCML* gene expression have also been detected in patients with schizophrenia, although protein level measurements from post mortem brains have not differed between patients and healthy controls [26].

In phosphoprotein analysis, several proteins were upregulated. Of these, PEG10 is a paternally imprinted gene that uses a rare mechanism for encoding for two different protein products by using the −1 ribosomal frameshift translation, which is well known from retroviruses and retrotransposons, but is extremely rare in humans [27]. In adult mice, the protein is expressed only in the brain and testes, and blocks TGF-B signaling. A paternally imprinted gene such as this one could explain why psychopathy is inherited from father to son. In this study, three of six offenders had a biological father convicted into prison, while none of the mothers had been imprisoned. PCDH19 is a protocadherin, which has been linked to epilepsy [28], autism [29] and behavioral problems, aggression, and photosensitivity. PCDH19 is thought to be a calcium-dependent cell-adhesion protein that is primarily expressed in the brain, and has been shown to cause a decrease in the amount of neurosteroids, including adrenocorticotropic hormone, in females. ACSL4 has been associated with X-chromosome linked mental retardation [30] as well as insulin secretion [31]. On the other hand, PSMD3 and MYO1e were substantially downregulated compared with controls. Of these, PSMD3 is an enzyme, an aberration of which contributes to pathogenesis of neurodevelopmental and neurodegenerative disorders [32, 33] and insulin resistance [34]. This finding suggests that downregulation of PSMD3 contributes to abnormal glucose metabolism which results into impulsive violent behavior among severely antisocial individuals as has been reported in several studies [14, 35]. MYO1e has been associated with autism in a single study [36].

In conclusion, expression of *ZNF132* in neurons and *RPL10P9* in both neurons and astrocytes is markedly abnormal among habitually violent offenders and these findings are strongly associated with the degree of psychopathic symptoms. The changes in protein levels observed here point to alteration in insulin sensitivity and glucose metabolism, and previous literature has shown that abnormal glucose metabolism is the only predictor for violent crimes which can surpass the accuracy of PCL-R [35]. We also observed changes in the opioid system, which has been shown to support prosocial functions, such as empathy, among humans and nonhuman primates [12, 13, 37, 38]. Our results showing a decrease in the expression of opioid delta receptor gene are in line with these previous findings. A recent theory suggests that a deficient endogenous opioid system contributes to antisocial personality, proposing that antisocial individuals attempt to stimulate their dysfunctional opioid system by the rewarding effect of substance abuse, and impulsive, sensation-seeking, aggressive, and promiscuous behavior [11]. Our data suggest that dysfunction of the opioid system contributes to the phenotype of psychopathy, supporting the recently presented idea that partial opioid receptor agonists, such as (+)-naloxone might be the first effective treatment for psychopathy [11].

## Supplementary information

Supplementary Information

Supplementary Table 2

Supplementary Table 3

Supplementary Table 4

## Data Availability

All data needed to evaluate the conclusions in the manuscript are provided in the manuscript or the supplementary material.
