# Correction: Neurobiological roots of psychopathy

**DOI:** 10.1038/s41380-019-0528-8

**Published:** 2019-09-30

**Authors:** Jari Tiihonen, Marja Koskuvi, Markku Lähteenvuo, Pekka L. J. Virtanen, Ilkka Ojansuu, Olli Vaurio, Yanyan Gao, Ida Hyötyläinen, Katja A. Puttonen, Eila Repo-Tiihonen, Tiina Paunio, Marja-Riitta Rautiainen, Sasu Tyni, Jari Koistinaho, Šárka Lehtonen

**Affiliations:** 1 Department of Forensic Psychiatry, Niuvanniemi Hospital, University of Eastern Finland, Niuvankuja 65, FI-70240 Kuopio, Finland; 2 Karolinska Institutet, Department of Clinical Neuroscience, Centre for Psychiatry Research, Stockholm County Council, Byggnad R5, SE-171 77 Stockholm, Sweden; 3 A.I. Virtanen Institute for Molecular Sciences, University of Eastern Finland, PO Box 1627, FI-70211 Kuopio, Finland; 4 Neuroscience Center, Helsinki Institute of Life Science, University of Helsinki, Haartmaninkatu 3, FI-00014 Helsinki, Finland; 5 Department of Mental Health and Substance Abuse Services and Public Health Genomics Unit, National Institute for Health and Welfare, PO Box 30, FI-00271 Helsinki, Finland; 6 Institute of Clinical Medicine, Department of Psychiatry, University of Helsinki, PO Box 22, (Välskärinkatu 12), FI-00014 Helsinki, Finland; 7 Department of Psychiatry, Helsinki University Central Hospital, PO Box 590, (Välskärinkatu 12), FI-00029 Helsinki, Finland; 8 Finnish Institute of Occupational Health, Development of Work and Work Organizations, Topeliuksenkatu 41 b, FI- 00290 Helsinki, Finland; 9 The Criminal Sanctions Agency, Lintulahdenkatu 5, FI-00530 Helsinki, Finland

**Keywords:** Psychiatric disorders, Neuroscience

**Correction to: Molecular Psychiatry**

10.1038/s41380-019-0488-z published online 27 August 2019

In the original version of this article, decimal places were accidentally omitted for all of the ‘Log2 fold change’ values, and some of the ‘Average expression’ values in Table 2. The original, incorrect version of Table 2 is displayed below. This has been corrected in both the PDF and HTML versions of the article. The publisher would like to apologise for this error, and any inconvenience it may have caused.

**Table 2:** Transcriptome analysis of differentially expressed genes in hiPSCs-derived cortical neurons

Ensembl ID	HGNC symbol	Gene description	Average expression	Log2 fold change	*P*-value	Adjusted *p*-value
Violent vs. control						
ENSG00000233913	RPL10P9	Ribosomal protein L10 pseudogene 9	98,909	3433	8.26E−10	4.29E−05
ENSG00000268912			79,387	2447	3.48E−09	9.06E−05
ENSG00000131849	ZNF132	Zinc finger protein 132	138,380	2407	4.37E−08	7.57E−04
ENSG00000256299			21,891	1526	3.39E−06	4.40E−02
Violent vs. control + nonviolent						
ENSG00000233913	RPL10P9	Ribosomal protein L10 pseudogene 9	98,909	3686	6.13E−10	1.84E−05
ENSG00000182397	DNM1P46	Dynamin 1 pseudogene 46	16,689	−1595	1.70E−07	2.13E−03
ENSG00000211459	MT-RNR1	Mitochondrially encoded 12 S RNA	3236,254	−2687	2.12E−07	2.13E−03
ENSG00000138347	MYPN	Myopalladin	7066	−2990	4.20E−07	3.16E−03
ENSG00000023839	ABCC2	ATP binding cassette subfamily C member 2	19,849	−1972	1.76E−06	1.01E−02
ENSG00000128284	APOL3	Apolipoprotein L3	13,244	−2741	2.02E−06	1.01E−02
ENSG00000235683			4804	−2446	5.86E−06	1.82E−02
ENSG00000140678	ITGAX	Integrin subunit alpha X	13,142	−2747	6.24E−06	1.82E−02
ENSG00000210151	MT-TS1	Mitochondrially encoded tRNA serine 1 (UCN)	17,046	−2165	7.14E−06	1.82E−02
ENSG00000172738	TMEM217	Transmembrane protein 217	40,619	−1633	7.58E−06	1.82E−02
ENSG00000179776	CDH5	Cadherin 5	7101	−2516	8.16E−06	1.82E−02
ENSG00000260075	NSFP1		7076	2313	8.31E−06	1.82E−02
ENSG00000172785	CBWD1	COBW domain containing 1	320,593	1208	8.47E−06	1.82E−02
ENSG00000204930	FAM221B	Family with sequence similarity 221 member B	7214	−2535	1.06E−05	2.09E−02
ENSG00000200503	SNORD115-5	Small nucleolar RNA, C/D box 115-5	28,237	2062	1.11E−05	2.09E−02
ENSG00000225630	MTND2P28	Mitochondrially encoded NADH:ubiq.	86,559	−2017	1.36E−05	2.24E−02
ENSG00000236064			9175	−2653	1.46E−05	2.24E−02
ENSG00000253426			5636	−2501	1.49E−05	2.24E−02
ENSG00000164659	KIAA1324L	KIAA1324 like	1178,161	1248	1.56E−05	2.24E−02
ENSG00000267334			6711	2063	1.56E−05	2.24E−02
ENSG00000188585	CLEC20A	C-type lectin domain containing 20A	3726	−2730	1.70E−05	2.33E−02
ENSG00000013588	GPRC5A	G protein-coupled receptor class C group 5 member A	19,264	−2356	2.02E−05	2.56E−02
ENSG00000257335	MGAM	MaltasE−glucoamylase	42,608	−1951	2.05E−05	2.56E−02
ENSG00000198899	MT-ATP6	Mitochondrially encoded ATP synthase 6	629,862	−1705	2.53E−05	3.04E−02
ENSG00000131042	LILRB2	Leukocyte immunoglobulin like receptor B2	2933	−2720	2.95E−05	3.41E−02
ENSG00000279301	OR2T11	Olfactory receptor family 2 subfamily T member 11	4182	−2634	3.13E−05	3.46E−02
ENSG00000268416			5112	−2644	3.22E−05	3.46E−02
ENSG00000268912			79,387	1852	3.47E−05	3.59E−02
ENSG00000252906	SCARNA3	Small Cajal body-specific RNA 3	35,106	1208	3.58E−05	3.59E−02
ENSG00000124731	TREM1	Triggering receptor expressed on myeloid cells 1	5602	−2372	4.35E−05	4.10E−02
ENSG00000101440	ASIP	Agouti signaling protein	3816	−2555	4.36E−05	4.10E−02
ENSG00000187812			7086	−2614	4.61E−05	4.20E−02
ENSG00000131849	ZNF132	Zinc finger protein 132	138,380	1920	5.01E−05	4.43E−02
ENSG00000060709	RIMBP2	RIMS binding protein 2	152,792	1619	5.68E−05	4.88E−02

Only genes with adjusted *p*-value < 0.05 and at least twofold up- or downregulation are presented

“Violent” indicates violent offenders, and “nonviolent” indicates individuals with substance abuse but without criminal behavior

