# Supplementary material for: Neurobiological roots of psychopathy
Source: Mol Psychiatry. 2019 Aug 27;25(12):3432–41. doi: 10.1038/s41380-019-0488-z (PMC7714686; doi:10.1038/s41380-019-0488-z)
Supplement: Supplementary file 1 — Supplementary Information [file 41380_2019_488_MOESM1_ESM.docx]

**Supplementary Information for**

**Neurobiological roots of psychopathy**

Jari Tiihonen, Marja Koskuvi, Markku Lähteenvuo, Pekka L.J. Virtanen, Ilkka Ojansuu, Olli Vaurio, Yanyan Gao, Ida Hyötyläinen, Katja A. Puttonen, Eila Repo-Tiihonen, Tiina Paunio, Marja-Riitta Rautiainen, Sasu Tyni, Jari Koistinaho, and Šárka Lehtonen

**Methods**

Immunocytochemistry

After one-week of maturation the cells were fixed in 4% paraformaldehyde in PBS for 20 minutes at room temperature (RT). hiPSCs or hiPSC-derived neurons or astrocytes were permeabilized at RT for 1 h in 0.25% Triton X-100 in PBS. To block unspecific binding sites, fixed cells were incubated in 5% normal goat serum in PBS for 1h at RT. In exception to this, cells exposed to NANOG were blocked in 0.1% BSA. The following primary antibodies and dilutions were used: mouse anti-OCT4 (Chemicon MAB4401), 1:400; goat anti-NANOG (R&D Systems AF1997), 1:100, mouse anti-TRA 1-81 (Chemicon MAB4381), 1:200; mouse anti-SSEA4 (Chemicon MAB4304), 1:400; mouse anti-AFP (Sigma A8452),1:500; mouse anti-SMA (Sigma A5228), 1:500; mouse anti-TUJ1 (Biolegend 801202), 1:2000; rabbit VGLUT1 (Sigma V0389), 1:300; mouse GAD67 (Abcam ab 26116), 1:100; rabbit GABA (Sigma 2052), 1:600. Secondary antibodies were Alexa Fluor goat 488 and 568 anti-rabbit (Invitrogen) and Alexa goat 488 and 568 anti-mouse (Invitrogen), all were used at 1:300. For NANOG and OPCML Alexa Fluor donkey 568 anti-goat (Invitrogen) were used. The nuclei were stained with 0.5 μg/ml DAPI (4',6-diamidino-2-phenylindole) and coverslips mounted with Vectashield or Fluoromount. Immunocytochemistry results showing the fractions of glutamatergic and GABAergic cells are presented in Supplementary Figure 3.

RNA isolation

Gene expression analyses were performed with hiPSC-derived cortical neurons and astrocytes. RNA was isolated from the plated neurons by mirVana kit (Thermo Fisher Scientific) through phenol-chloroform extraction and purification on a glass fiber filter followed by elution in nuclease-free water according to the manufacturer’s protocol. RNA isolation for hiPSC-astrocytes was performed with RNeasy Mini kit (Qiagen) where RNA binds to silica-membrane spin columns and is eluted in nuclease-free water. RNA integrity was confirmed on 2100 Bioanalyzer (Agilent Technologies).

Gene expression profile

Whole transcriptome was sequenced using the Illumina Hiseq 2500 (Life Technologies) at the Functional Genomics Unit (Biomedicum, Helsinki). Using STAR aligner (version 2.5.2), RNA-Seq reads were aligned to human GRCh38 (hg38) genome assembly. Gene-level read counts were obtained simultaneously with the alignment process. Data were normalized using regularized log transformation function and differential expression analysis by R package DESeq2 [1]. The Benjamini-Hochberg method to correct for multiple testing [2] was selected for p-value adjustments. Genes with absolute log2 fold change >1 and adjusted p<0.05 were considered as significantly differentially expressed. Gene set enrichment analysis (GSEA) [3] in addition to Biological Process Gene Ontology (GOBP) term and Kyoto Encyclopedia of Genes and Genomes (KEGG) pathway term over-representation analyses were performed using R package clusterProfiler [4].

qRT-PCR

For RT-qPCR, cDNA was synthesized using Maxima reverse transcriptase enzyme (Thermo Fisher Scientific). Maxima Probe qPCR Master Mix (Thermo Fisher Scientific) and TaqMan probes from Thermo Fisher Scientific were used in the qPCR measurement. Primers used were: NANOG Hs02387400_g1; OCT4 (POU5F1) Hs00742896_s1; SOX2 Hs01053049_s1; LIN28A Hs00702808_s1; SEV Mr04269880_mr; and ZNF132 Hs0103687_m1. qPCR data were analyzed using double delta Ct analysis and normalized to beta-actin (ACTB).

Quantitative proteomic analysis

Proteins were isolated from hiPSC-derived neurons and dissolved in 8 M urea, 75 mM NaCl, 50 mM Tris, pH 8.2 solution. 500 μg of proteins were digested by trypsin according to a standard in-solution digestion protocol. Tryptic peptides were desalted by 96-well Sep-Pak C18 plate. Peptides were eluted by 0.1% formic acid / 60% acetonitrile and evaporated to dryness. For phosphopeptide enrichment, 99% of the peptides were taken. The rest of the samples were saved for total proteome quantitation. Phosphopeptides were enriched by High-SelectTM TiO2 Phosphopeptide Enrichment Kit (Thermo Fisher Scientific) according to manufacturer’s instructions. The LC-ESI-MS/MS (liquid chromatography-electrospray ionization-tandem mass spectrometry) analyses were performed at the Turku Proteomics Facility, University of Turku and Åbo Akademi University on a nanoflow HPLC system (Easy-nLC1200, Thermo Fisher Scientific) coupled to a Q Exactive HF mass spectrometer (Thermo Fisher Scientific, Bremen, Germany) equipped with a nano-electrospray ionization source. Peptides were loaded on a C18 column (75 μm x 40 cm, ReproSil-Pur 1.9 μm 120 Å C18-AQ, Dr. Maisch HPLC GmbH, Ammerbuch-Entringen, Germany) with a constant 800 bar pressure. The mobile phase consisted of water with 0.1% formic acid (solvent A) or acetonitrile/water (80:20 (v/v) with 0.1% formic acid (solvent B). A 50 min gradient from 6% to 21% B, followed by 50 min from 21% to 36% B was used to elute peptides. After this this the column was washed with 100% B. MS data was acquired automatically by using Thermo Xcalibur 3.1 software (Thermo Fisher Scientific). An information dependent acquisition method consisted of an Orbitrap MS survey scan of mass range 300-2000 m/z followed by HCD fragmentation for 10 most intensive ions.

Quantitative proteomic and phosphoproteomic analyses were performed by the Bioinformatics group at the Turku Centre for Biotechnology. Proteins were identified and quantified with ProteomeDiscoverer (version 2.2) Label Free Quantification (LFQ) workflow. Peptide search was performed using Mascot and Percolator against UniProt KB/Swiss-Prot human database (version 2017_09) using false discovery rate 0.01 as a threshold for peptide identification. Phosphosite localization was done using the PhosphoRS algorithm. In total, 5802 proteins were included in the downstream analysis of the proteome and 3098 proteins of the phosphoproteome respectively. Phosphoproteome samples were filtered time-based in such a way that LC retention time after 53 min was discarded. The data were normalized (median normalization) to remove variation between samples caused by non-biological reasons and to make the values comparable across the sample set.

**Supplementary Table 1. Summary of all hiPSC lines generated in this project and the assays employed for characterizing the hiPSC lines.**


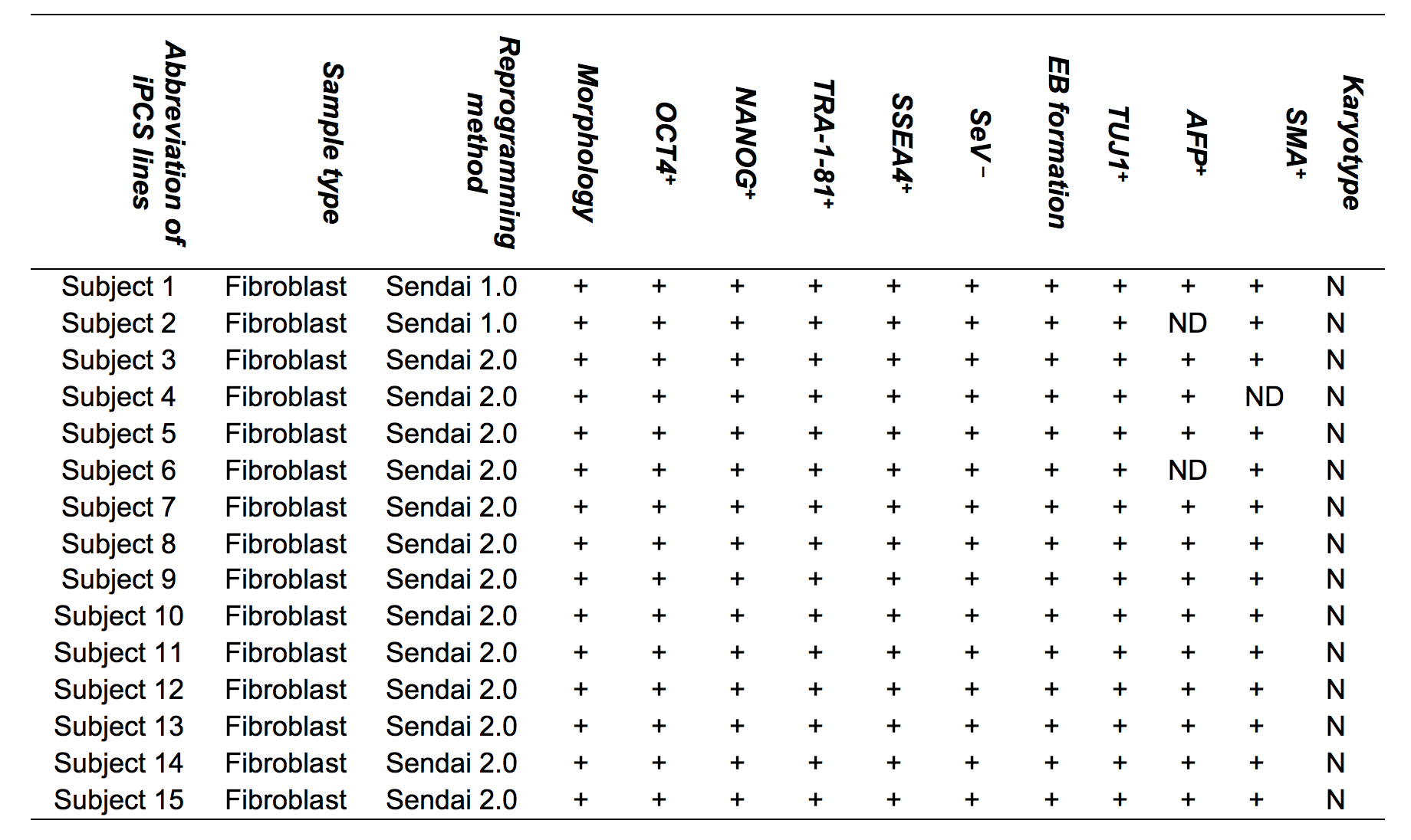


ND, not determined; N, normal


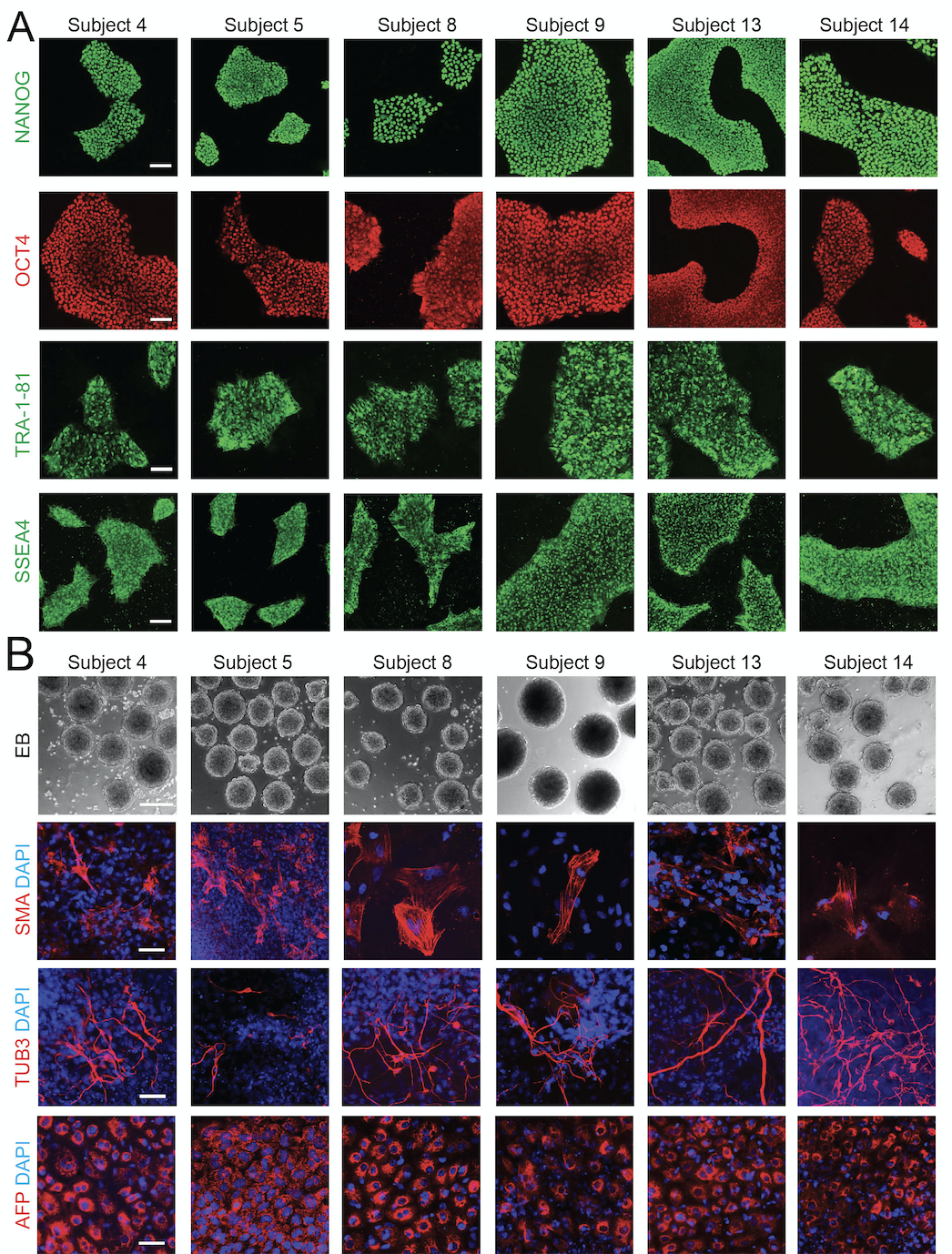


**Supplementary Figure 1.** **Characterization of hiPSC derived lines**. (A) Representative immunofluorescence images of OCT4, NANOG, TRA 1-81 and SSEA4 from iPS cells grown on top of Matrigel. Scale bars 100 μm. (B) Representative bright-field images of EBs (scale bar 200 μm) and immunofluorescence images (scale bars 50 µm) of the three embryonic germ layers, including smooth muscle actin (SMA, mesoderm; red), beta III tubulin (TUB3, ectoderm; red) and alpha-fetoprotein (AFP, endoderm; red) from Control lines: subjects 13 and 14, Non-violent: subjects 8 and 9 and Violent: subjects 4 and 5.

**Supplementary Figure 2.** **Characterization of hiPSC derived lines**. (A) Representative karyograms of studied hiPSC lines from Control lines: subjects 13 and 14, Non-violent: subjects 8 and 9 and Violent: subjects 4 and 5. (B) Beta-actin (*ACTB*) normalized gene expression levels of pluripotency-promoting genes including *NANOG, LIN28, OCT4,* and *SOX2*. The mRNA expression levels are presented as mean ± SEM.


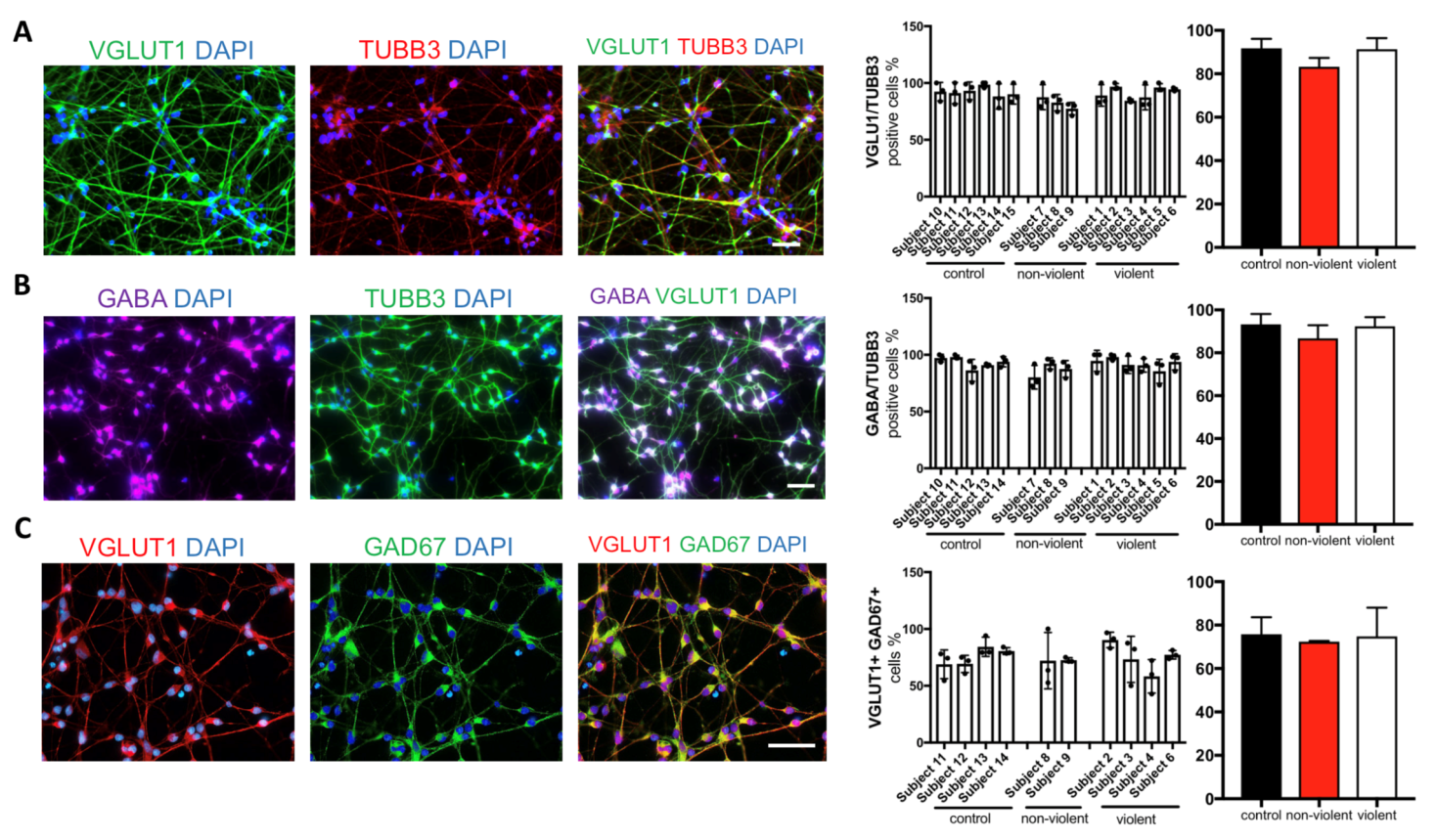
**Supplementary Figure 3. Immunocytochemistry results showing the fractions of glutamatergic and GABAergic cells.** hiPSC-derived neurons stained for (A) VGLUT1 (green), TUBB3 (red), (B) GABA (violet) and TUBB3 (green), (C) VGLUT1 (red) and GAD67 (green) and its quantifications. Nuclei are counterstained with DAPI. Altogether three images per each subject were scored. Number of positively stained neurons is normalized to DAPI and showed in %. Results are presented as mean +/- SD. Scale bar 50 μm. VGLUT1, vesicular glutamate transporter 1; TUBB3, tubulin Beta 3 Class III; GABA, γ-aminobutyric acid; GAD67, glutamate decarboxylase; DAPI, 4′,6-diamidino-2-phenylindole. Glutamatergic and GABAergic markers are partially co-expressed in the same neurons as has been reported in previous studies [5–7].

**Supplementary Figure 4. The expression of *ZNF132* mRNA in different cell types.** *ZNF13*2 mRNA expression levels measured by quantitative RT-PCR from (A) skin fibroblasts, (B) hiPSCs and (C) hiPSC-derived cortical neurons. The mRNA expression levels are presented as mean relative expression normalized to *ACTB* ± SD.

**References**

1. Love MI, Huber W, Anders S. Moderated estimation of fold change and dispersion for RNAseq data with DESeq2. *Genome Biol* 2014; **15:** 550.

2. Benjamini Y, Hochberg Y. Controlling the false discovery rate: a practical and powerful approach to multiple testing. *J Royal Stat Soc B* 1995; **57:** 289-300.

3. Subramanian A, Tamayo P, Mootha VK, Mukherjee S, Ebert BL, Gillette MA et al. Gene set enrichment analysis: A knowledgebased approach for interpreting genome-wide expression profiles. *Proc Natl Acad Sci USA* 2005; **102:** 15545-15550.

4. Yu G, Wang L-G, Han Y, He Q-Y. clusterProfiler: an R package for comparing biological themes among gene clusters. *OMICS* 2012; **16,** 284-287.

5. Fattorini G, Antonucci F, Menna E, Matteoli M, Conti F. Co-expression of VGLUT1 and VGAT sustains glutamate and GABA co-release and is regulated by activity in cortical neurons. *J Cell Sci* 2015; **128,** 1669-1673.

6. Root DH, Zhang S, Barker DJ, Miranda-Barrientos J, Liu B, Wang HL, Morales M. Selective brain distribution and distinctive synaptic architecture of dual glutamatergic-GABAergic neurons. *Cell Rep.* 2018; **23,** 3465-3479.

7. Cattaneo S, Zaghi M, Maddalena R, Bedogni F, Sessa A, Taverna S. Somatostatin-expressing interneurons co-release GABA and glutamate onto different postsynaptic targets in the striatum. https://www.biorxiv.org/content/10.1101/566984v2.
