# Supplementary material for: Neurobiological roots of psychopathy
Source: Mol Psychiatry. 2019 Aug 27;25(12):3432–41. doi: 10.1038/s41380-019-0488-z (PMC7714686; doi:10.1038/s41380-019-0488-z)
Supplement: Supplementary file 2 — Supplementary Table 2 [file 41380_2019_488_MOESM2_ESM.pdf]

Suppl Table 2_Differentially expressed genes in neurons among violent offenders versus healthy controls

Differential gene expression results							
**Ensembl ID**	**HGNC symbol**	**Gene description**	**Gene biotype**	**Average expression**	**Log2 foldchange**	**P-value**	**Adjusted p-value**
ENSG00000233913	RPL10P9	ribosomal protein L10 pseudogene 9 [Source:HGNC Symbol;Acc:HGNC:35579]	processed_pseudogene	98,9086155	3,432908226	8,25764357842892E-10	4,290589027E-05
ENSG00000268912			lincRNA	79,38681476	2,447221024	3,48649240512509E-09	9,057732944E-05
ENSG00000131849	ZNF132	zinc finger protein 132 [Source:HGNC Symbol;Acc:HGNC:12916]	protein_coding	138,3796984	2,407483358	4,37037575307365E-08	0,000756935
ENSG00000256299			lincRNA	21,89050185	1,526482228	3,39073342110157E-06	0,044044779
ENSG00000107807	TLX1	T-cell leukemia homeobox 1 [Source:HGNC Symbol;Acc:HGNC:5056]	protein_coding	9,11854719	-2,463966906	5,34441906367901E-06	0,055538134
ENSG00000185053	SGCZ	sarcoglycan zeta [Source:HGNC Symbol;Acc:HGNC:14075]	protein_coding	16,81532365	1,839777435	2,2789412672424E-05	0,187777155
ENSG00000128284	APOL3	apolipoprotein L3 [Source:HGNC Symbol;Acc:HGNC:14868]	protein_coding	13,24403807	-2,239235587	2,52976402105966E-05	0,187777155
ENSG00000175591	P2RY2	purinergic receptor P2Y2 [Source:HGNC Symbol;Acc:HGNC:8541]	protein_coding	5,405472127	-2,145541297	3,49002438783743E-05	0,226672721
ENSG00000164659	KIAA1324L	KIAA1324 like [Source:HGNC Symbol;Acc:HGNC:21945]	protein_coding	1178,160795	1,02934377	5,24334042834303E-05	0,27561063
ENSG00000116329	OPRD1	opioid receptor delta 1 [Source:HGNC Symbol;Acc:HGNC:8153]	protein_coding	56,0250477	-1,609495554	5,53505786226229E-05	0,27561063
ENSG00000165694	FRMD7	FERM domain containing 7 [Source:HGNC Symbol;Acc:HGNC:8079]	protein_coding	27,86647773	-2,310951729	5,90666911709552E-05	0,27561063
ENSG00000245213			antisense_RNA	13,49993796	-1,259987212	6,36526408196198E-05	0,27561063
ENSG00000203883	SOX18	SRY-box 18 [Source:HGNC Symbol;Acc:HGNC:11194]	protein_coding	6,424521207	-2,217293908	7,50793595461705E-05	0,300080649
ENSG00000204767	FAM196B	family with sequence similarity 196 member B [Source:HGNC Symbol;Acc:HGNC:37271]	protein_coding	32,66716158	1,670057643	8,22805496171345E-05	0,305372506
ENSG00000258590	NBEAP1	neurobeachin pseudogene 1 [Source:HGNC Symbol;Acc:HGNC:1007]	transcribed_unprocessed_pseudogene	8,587268184	-2,179944077	9,16605149287789E-05	0,317505913
ENSG00000069667	RORA	RAR related orphan receptor A [Source:HGNC Symbol;Acc:HGNC:10258]	protein_coding	542,2048328	1,035793386	0,000106192	0,344852186
ENSG00000179776	CDH5	cadherin 5 [Source:HGNC Symbol;Acc:HGNC:1764]	protein_coding	7,101006834	-1,973743001	0,000124994	0,382034492
ENSG00000082293	COL19A1	collagen type XIX alpha 1 chain [Source:HGNC Symbol;Acc:HGNC:2196]	protein_coding	154,2278045	2,160162521	0,000139696	0,403247315
ENSG00000164626	KCNK5	potassium two pore domain channel subfamily K member 5 [Source:HGNC Symbol;Acc:HGNC:6280]	protein_coding	9,115539844	-2,101937744	0,000155484	0,425199744
ENSG00000278012			sense_intronic	14,34855829	1,534044211	0,000167621	0,435469858
ENSG00000141448	GATA6	GATA binding protein 6 [Source:HGNC Symbol;Acc:HGNC:4174]	protein_coding	13,05844022	-2,099099522	0,000201046	0,497435933
ENSG00000174279	EVX2	even-skipped homeobox 2 [Source:HGNC Symbol;Acc:HGNC:3507]	protein_coding	11,3217572	-2,040176007	0,000221653	0,523492973
ENSG00000233073			antisense_RNA	13,35915368	1,627489043	0,000320407	0,7238268
ENSG00000215030	RPL13P12		processed_pseudogene	358,330596	1,856073735	0,000343173	0,742955341
ENSG00000117266	CDK18	cyclin dependent kinase 18 [Source:HGNC Symbol;Acc:HGNC:8751]	protein_coding	69,85665144	-1,098782369	0,000400628	0,75810953
ENSG00000172014	ANKRD20A4	ankyrin repeat domain 20 family member A4 [Source:HGNC Symbol;Acc:HGNC:31982]	protein_coding	6,842265564	1,814326205	0,000415971	0,75810953
ENSG00000171794	UTF1	undifferentiated embryonic cell transcription factor 1 [Source:HGNC Symbol;Acc:HGNC:12634]	protein_coding	6,052491352	-2,008194764	0,000421671	0,75810953
ENSG00000183153	GJD3	gap junction protein delta 3 [Source:HGNC Symbol;Acc:HGNC:19147]	protein_coding	4,606457183	-1,863874603	0,00043993	0,75810953
ENSG00000186564	FOXD2	forkhead box D2 [Source:HGNC Symbol;Acc:HGNC:3803]	protein_coding	12,14054752	-1,87000807	0,000443417	0,75810953
ENSG00000091879	ANGPT2	angiopoietin 2 [Source:HGNC Symbol;Acc:HGNC:485]	protein_coding	140,9867944	1,941336774	0,000467676	0,75810953
ENSG00000057657	PRDM1	PR/SET domain 1 [Source:HGNC Symbol;Acc:HGNC:9346]	protein_coding	29,95036281	-1,750285614	0,000472765	0,75810953
ENSG00000260389		WW domain binding protein 11 pseudogene 1 [Source:NCBI gene;Acc:441818]	transcribed_processed_pseudogene	2,245313557	-2,012633817	0,000474986	0,75810953
ENSG00000179520	SLC17A8	solute carrier family 17 member 8 [Source:HGNC Symbol;Acc:HGNC:20151]	protein_coding	49,66407403	1,788206853	0,000500272	0,75810953
ENSG00000224897	POT1-AS1	POT1 antisense RNA 1 [Source:HGNC Symbol;Acc:HGNC:49459]	antisense_RNA	92,81549418	0,719958817	0,000509719	0,75810953
ENSG00000175556	LONRF3	LON peptidase N-terminal domain and ring finger 3 [Source:HGNC Symbol;Acc:HGNC:21152]	protein_coding	57,05495113	-1,711374813	0,000510669	0,75810953
ENSG00000267984			antisense_RNA	10,33495487	1,961857538	0,000544032	0,772017923
ENSG00000149243	KLHL35	kelch like family member 35 [Source:HGNC Symbol;Acc:HGNC:26597]	protein_coding	274,5483801	-1,225729906	0,000549754	0,772017923
ENSG00000204612	FOXB2	forkhead box B2 [Source:HGNC Symbol;Acc:HGNC:23315]	protein_coding	2,45810178	-1,97868394	0,000578601	0,79114618
ENSG00000269646			lincRNA	5,644665801	1,549612525	0,000602858	0,800795804
ENSG00000113361	CDH6	cadherin 6 [Source:HGNC Symbol;Acc:HGNC:1765]	protein_coding	3171,656791	-1,300058513	0,000616483	0,800795804
ENSG00000182177	ASB18	ankyrin repeat and SOCS box containing 18 [Source:HGNC Symbol;Acc:HGNC:19770]	protein_coding	8,578089638	-1,897724875	0,000648199	0,810274832
ENSG00000005020	SKAP2	src kinase associated phosphoprotein 2 [Source:HGNC Symbol;Acc:HGNC:15687]	protein_coding	71,6115059	1,201332326	0,000654969	0,810274832
ENSG00000283632	EXOC3L2	exocyst complex component 3 like 2 [Source:HGNC Symbol;Acc:HGNC:30162]	protein_coding	9,011522284	-1,746235566	0,00070528	0,852224508
ENSG00000110237	ARHGEF17	Rho guanine nucleotide exchange factor 17 [Source:HGNC Symbol;Acc:HGNC:21726]	protein_coding	707,2191282	-0,560255277	0,000727489	0,859081893
ENSG00000079689	SCGN	secretagogin, EF-hand calcium binding protein [Source:HGNC Symbol;Acc:HGNC:16941]	protein_coding	1620,298505	-1,930069153	0,000745498	0,860785151
ENSG00000261614			processed_pseudogene	2,821778626	-1,909988202	0,000778189	0,869333666
ENSG00000178015	GPR150	G protein-coupled receptor 150 [Source:HGNC Symbol;Acc:HGNC:23628]	protein_coding	6,966203704	-1,748853317	0,000787638	0,869333666
ENSG00000204930	FAM221B	family with sequence similarity 221 member B [Source:HGNC Symbol;Acc:HGNC:30762]	protein_coding	7,214258489	-1,773572781	0,000803095	0,869333666
ENSG00000147403	RPL10	ribosomal protein L10 [Source:HGNC Symbol;Acc:HGNC:10298]	protein_coding	3842,2688031862	-0,698228443	0,000968964	1
ENSG00000230623			lincRNA	2,76651649	-1,800279401	0,000988035	1
ENSG00000151553	FAM160B1	family with sequence similarity 160 member B1 [Source:HGNC Symbol;Acc:HGNC:29320]	protein_coding	1244,55806	0,307302733	0,000993153	1
ENSG00000228075	BOD1L2	biorientation of chromosomes in cell division 1 like 2 [Source:HGNC Symbol;Acc:HGNC:28505]	protein_coding	3,055042757	-1,889175586	0,001035232	1
ENSG00000102445	RUBCNL	RUN and cysteine rich domain containing beclin 1 interacting protein like [Source:HGNC Symbol;Acc:HGNC:20420]	protein_coding	34,02321531	1,38565897	0,001077776	1
ENSG00000187391	MAGI2	membrane associated guanylate kinase, WW and PDZ domain containing 2 [Source:HGNC Symbol;Acc:HGNC:18957]	protein_coding	1622,578589	0,825093164	0,001098021	1
ENSG00000181201	HIST3H2BA	histone cluster 3 H2B family member a (pseudogene) [Source:HGNC Symbol;Acc:HGNC:20515]	unitary_pseudogene	101,2198542	-1,536254637	0,001110199	1
ENSG00000162676	GFI1	growth factor independent 1 transcriptional repressor [Source:HGNC Symbol;Acc:HGNC:4237]	protein_coding	9,25718347	-1,493988108	0,00112579	1
ENSG00000271344			lincRNA	14,19249128	1,31496859	0,001163638	1
ENSG00000279143			TEC	2,541572018	-1,863945467	0,001219489	1
ENSG00000164287	CDC20B	cell division cycle 20B [Source:HGNC Symbol;Acc:HGNC:24222]	protein_coding	19,31120132	-1,848943588	0,001225071	1
ENSG00000237550	RPL9P9	ribosomal protein L9 pseudogene 9 [Source:HGNC Symbol;Acc:HGNC:17251]	transcribed_processed_pseudogene	224,0962822	1,603236652	0,001316399	1
ENSG00000255181	CCDC166	coiled-coil domain containing 166 [Source:HGNC Symbol;Acc:HGNC:41910]	protein_coding	2,823509669	-1,737495705	0,00133777	1
ENSG00000204967	PCDHA4	protocadherin alpha 4 [Source:HGNC Symbol;Acc:HGNC:8670]	protein_coding	626,9908869	0,803974694	0,001361241	1
ENSG00000164093	PITX2	paired like homeodomain 2 [Source:HGNC Symbol;Acc:HGNC:9005]	protein_coding	14,64784909	-1,667492906	0,00138682	1
ENSG00000223403	MEG9	maternally expressed 9 (non-protein coding) [Source:HGNC Symbol;Acc:HGNC:43874]	lincRNA	31,94333829	1,839384806	0,001392596	1

ENSG00000230798	FOXD3-AS1	FOXD3 antisense RNA 1 (head to head) [Source:HGNC Symbol;Acc:HGNC:40241]	antisense_RNA	4,806736521	-1,652074233	0,001406176	1
ENSG00000133121	STARD13	StAR related lipid transfer domain containing 13 [Source:HGNC Symbol;Acc:HGNC:19164]	protein_coding	233,1380251	1,08429469	0,001483354	1
ENSG00000224855	OPA1-AS1	OPA1 antisense RNA 1 [Source:HGNC Symbol;Acc:HGNC:40421]	antisense_RNA	2,375937879	1,781684697	0,001509156	1
ENSG00000274317	LINC02334	long intergenic non-protein coding RNA 2334 [Source:HGNC Symbol;Acc:HGNC:53254]	lincRNA	17,58216952	-1,821287733	0,00151502	1
ENSG00000106852	LHX6	LIM homeobox 6 [Source:HGNC Symbol;Acc:HGNC:21735]	protein_coding	531,5941911	1,751123094	0,001570096	1
ENSG00000223652			antisense_RNA	2,664616053	-1,800806614	0,001646344	1
ENSG00000280385			TEC	25,04245632	1,410686047	0,001707755	1
ENSG00000237016			processed_pseudogene	2,494477629	-1,771877785	0,00176849	1
ENSG00000284188			protein_coding	3,447479448	-1,797211086	0,001802007	1
ENSG00000100156	SLC16A8	solute carrier family 16 member 8 [Source:HGNC Symbol;Acc:HGNC:16270]	protein_coding	5,479419163	-1,750308275	0,001819661	1
ENSG00000164651	SP8	Sp8 transcription factor [Source:HGNC Symbol;Acc:HGNC:19196]	protein_coding	1051,012425	-1,473346035	0,001939047	1
ENSG00000152953	STK32B	serine/threonine kinase 32B [Source:HGNC Symbol;Acc:HGNC:14217]	protein_coding	217,0630984	1,159503381	0,001983257	1
ENSG00000260409			lincRNA	3,645194622	-1,736492051	0,001983812	1
ENSG00000163497	FEV	FEV, ETS transcription factor [Source:HGNC Symbol;Acc:HGNC:18562]	protein_coding	3,816890096	-1,781415234	0,001995923	1
ENSG00000170748	RBMXL2	RNA binding motif protein, X-linked like 2 [Source:HGNC Symbol;Acc:HGNC:17886]	protein_coding	3,532928372	-1,771753767	0,002093169	1
ENSG00000261790			lincRNA	13,59431984	1,253924902	0,002097319	1
ENSG00000275910			antisense_RNA	3,858791277	1,737147102	0,002108353	1
ENSG00000152270	PDE3B	phosphodiesterase 3B [Source:HGNC Symbol;Acc:HGNC:8779]	protein_coding	370,2415606	-0,920581343	0,002145023	1
ENSG00000231389	HLA-DPA1	major histocompatibility complex, class II, DP alpha 1 [Source:HGNC Symbol;Acc:HGNC:4938]	protein_coding	58,56618525	-1,76071684	0,002185596	1
ENSG00000037280	FLT4	fms related tyrosine kinase 4 [Source:HGNC Symbol;Acc:HGNC:3767]	protein_coding	43,6997014	-1,154585279	0,002198777	1
ENSG00000157014	TATDN2	TatD DNase domain containing 2 [Source:HGNC Symbol;Acc:HGNC:28988]	protein_coding	287,6995839	0,46811066	0,00219995	1
ENSG00000173535	TNFRSF10C	TNF receptor superfamily member 10c [Source:HGNC Symbol;Acc:HGNC:11906]	protein_coding	11,30508058	-1,341108924	0,00222827	1
ENSG00000279301	OR2T11	olfactory receptor family 2 subfamily T member 11 (gene/pseudogene) [Source:HGNC Symbol;Acc:HGNC:19574]	polymorphic_pseudogene	4,181888374	-1,744046224	0,00223969	1
ENSG00000253426			lincRNA	5,635606026	-1,619544496	0,002246119	1
ENSG00000222033	LINC01124	long intergenic non-protein coding RNA 1124 [Source:HGNC Symbol;Acc:HGNC:49270]	lincRNA	5,321775568	-1,715698148	0,00228518	1
ENSG00000268509			antisense_RNA	6,909397926	-1,54495932	0,002311943	1
ENSG00000176383	B3GNT4	UDP-GlcNAc:betaGal beta-1,3-N-acetylglucosaminyltransferase 4 [Source:HGNC Symbol;Acc:HGNC:15683]	protein_coding	82,67451645	0,93642703	0,00240087	1
ENSG00000131462	TUBG1	tubulin gamma 1 [Source:HGNC Symbol;Acc:HGNC:12417]	protein_coding	571,513572	-0,470289393	0,002432755	1
ENSG00000119973	PRLHR	prolactin releasing hormone receptor [Source:HGNC Symbol;Acc:HGNC:4464]	protein_coding	68,93050778	1,74437806	0,002474159	1
ENSG00000189292	ALKAL2	ALK and LTK ligand 2 [Source:HGNC Symbol;Acc:HGNC:27683]	protein_coding	33,43354421	-1,527683512	0,002601882	1
ENSG00000164379	FOXQ1	forkhead box Q1 [Source:HGNC Symbol;Acc:HGNC:20951]	protein_coding	16,34328443	-1,585085467	0,002611703	1
ENSG00000252047		Y RNA [Source:RFAM;Acc:RF00019]	misc_RNA	1,949222183	1,689432254	0,002638155	1
ENSG00000224189	HAGLR	HOXD antisense growth-associated long non-coding RNA [Source:HGNC Symbol;Acc:HGNC:43755]	antisense_RNA	7,165404638	-1,66654073	0,002659823	1
ENSG00000074211	PPP2R2C	protein phosphatase 2 regulatory subunit Bgamma [Source:HGNC Symbol;Acc:HGNC:9306]	protein_coding	233,2090407803	1,040727274	0,002683695	1
ENSG00000170162	VGLL2	vestigial like family member 2 [Source:HGNC Symbol;Acc:HGNC:20232]	protein_coding	2,847041541	-1,707898414	0,002697969	1
ENSG00000132518	GUCY2D	guanylate cyclase 2D, retinal [Source:HGNC Symbol;Acc:HGNC:4689]	protein_coding	6,576696433	-1,626555027	0,002751145	1
ENSG00000171119	NRTN	neurturin [Source:HGNC Symbol;Acc:HGNC:8007]	protein_coding	18,31461167	-1,53245874	0,002758401	1
ENSG00000185761	ADAMTSL5	ADAMTS like 5 [Source:HGNC Symbol;Acc:HGNC:27912]	protein_coding	7,664319506	-1,643134965	0,00280277	1
ENSG00000268869	ESPNP	espin pseudogene [Source:HGNC Symbol;Acc:HGNC:23285]	transcribed_unprocessed_pseudogene	2,08574512	-1,690063679	0,002804738	1
ENSG00000266605	LONRF2P1		processed_pseudogene	2,017348587	-1,716220766	0,002905071	1
ENSG00000136931	NR5A1	nuclear receptor subfamily 5 group A member 1 [Source:HGNC Symbol;Acc:HGNC:7983]	protein_coding	5,89056931	-1,639552022	0,003011837	1
ENSG00000269946			lincRNA	8,183718942	-1,249211861	0,00305785	1
ENSG00000069011	PITX1	paired like homeodomain 1 [Source:HGNC Symbol;Acc:HGNC:9004]	protein_coding	14,21606806	-1,444262144	0,003065082	1
ENSG00000166006	KCNC2	potassium voltage-gated channel subfamily C member 2 [Source:HGNC Symbol;Acc:HGNC:6234]	protein_coding	152,5789579	1,580009393	0,00313657	1
ENSG00000187140	FOXD3	forkhead box D3 [Source:HGNC Symbol;Acc:HGNC:3804]	protein_coding	6,492024443	-1,701903033	0,003149819	1
ENSG00000142089	IFITM3	interferon induced transmembrane protein 3 [Source:HGNC Symbol;Acc:HGNC:5414]	protein_coding	274,1618736	-1,638073029	0,003178277	1
ENSG00000237732			transcribed_unprocessed_pseudogene	35,2275138	-1,591773427	0,003207882	1
ENSG00000158525	CPA5	carboxypeptidase A5 [Source:HGNC Symbol;Acc:HGNC:15722]	protein_coding	5,408917852	-1,4840777	0,003219207	1
ENSG00000267448			antisense_RNA	10,82735014	1,63456957	0,003319055	1
ENSG00000144057	ST6GAL2	ST6 beta-galactoside alpha-2,6-sialyltransferase 2 [Source:HGNC Symbol;Acc:HGNC:10861]	protein_coding	3398,930119	1,108859208	0,003420393	1
ENSG00000255126			antisense_RNA	6,508857962	1,661736418	0,003456134	1
ENSG00000091039	OSBPL8	oxysterol binding protein like 8 [Source:HGNC Symbol;Acc:HGNC:16396]	protein_coding	4486,723728	0,35980426	0,003469325	1
ENSG00000210196	MT-TP	mitochondrially encoded tRNA proline [Source:HGNC Symbol;Acc:HGNC:7494]	Mt_tRNA	331,7678768	1,333898795	0,00353789	1
ENSG00000039600	SOX30	SRY-box 30 [Source:HGNC Symbol;Acc:HGNC:30635]	protein_coding	2,608390649	-1,606705552	0,003566386	1
ENSG00000150722	PPP1R1C	protein phosphatase 1 regulatory inhibitor subunit 1C [Source:HGNC Symbol;Acc:HGNC:14940]	protein_coding	33,00838307	1,507871326	0,003591201	1
ENSG00000223779			unprocessed_pseudogene	3,733981053	-1,657005185	0,00359277	1
ENSG00000261594	TPBGL	trophoblast glycoprotein like [Source:HGNC Symbol;Acc:HGNC:44159]	protein_coding	11,595512	-1,580765988	0,00362312	1
ENSG00000177600	RPLP2	ribosomal protein lateral stalk subunit P2 [Source:HGNC Symbol;Acc:HGNC:10377]	protein_coding	3036,638517	-0,517195732	0,003631927	1
ENSG00000107854	TNKS2	tankyrase 2 [Source:HGNC Symbol;Acc:HGNC:15677]	protein_coding	2561,223984	0,350902076	0,00366009	1
ENSG00000207569	MIR433	microRNA 433 [Source:HGNC Symbol;Acc:HGNC:32026]	miRNA	4,258886891	1,619673819	0,003808136	1
ENSG00000277558			sense_intronic	6,816951964	-1,24423813	0,003922902	1
ENSG00000108813	DLX4	distal-less homeobox 4 [Source:HGNC Symbol;Acc:HGNC:2917]	protein_coding	3,741128375	-1,581234371	0,003941241	1
ENSG00000230729			antisense_RNA	13,07525556	1,561672787	0,004039363	1
ENSG00000268592		RAET1E antisense RNA 1 [Source:NCBI gene;Acc:100652739]	antisense_RNA	18,10604185	-0,901661082	0,004039948	1
ENSG00000170832	USP32	ubiquitin specific peptidase 32 [Source:HGNC Symbol;Acc:HGNC:19143]	protein_coding	1636,373246	0,291705969	0,004053068	1
ENSG00000259519			antisense_RNA	2,420752552	1,648577825	0,004170311	1
ENSG00000198488	B3GNT6	UDP-GlcNAc:betaGal beta-1,3-N-acetylglucosaminyltransferase 6 [Source:HGNC Symbol;Acc:HGNC:24141]	protein_coding	7,940669435	-1,587584205	0,004180454	1

ENSG00000213024	NUP62	nucleoporin 62 [Source:HGNC Symbol;Acc:HGNC:8066]	protein_coding	1216,239916	-0,427333774	0,004210449	1
ENSG00000075673	ATP12A	ATPase H+/K+ transporting non-gastric alpha2 subunit [Source:HGNC Symbol;Acc:HGNC:13816]	protein_coding	7,782840184	-1,418118018	0,004301094	1
ENSG00000210151	MT-TS1	mitochondrially encoded tRNA serine 1 (UCN) [Source:HGNC Symbol;Acc:HGNC:7497]	Mt_tRNA	17,04619595	-1,278074152	0,004314002	1
ENSG00000160224	AIRE	autoimmune regulator [Source:HGNC Symbol;Acc:HGNC:360]	protein_coding	3,769328749	-1,619459344	0,004344858	1
ENSG00000214552	COPS8P2	COP9 signalosome subunit 8 pseudogene 2 [Source:HGNC Symbol;Acc:HGNC:45270]	processed_pseudogene	6,849199826	1,313760848	0,004397712	1
ENSG00000284730			protein_coding	5,441266861	-1,631192212	0,004404107	1
ENSG00000226149			lincRNA	14,20991947	1,119663157	0,004501164	1
ENSG00000113319	RASGRF2	Ras protein specific guanine nucleotide releasing factor 2 [Source:HGNC Symbol;Acc:HGNC:9876]	protein_coding	510,3440164	1,536231432	0,004531107	1
ENSG00000282881			protein_coding	3,279211328	-1,635228914	0,004531837	1
ENSG00000170374	SP7	Sp7 transcription factor [Source:HGNC Symbol;Acc:HGNC:17321]	protein_coding	12,57228029	-1,577943699	0,004566667	1
ENSG00000261320			lincRNA	2,350476787	-1,621525563	0,004604788	1
ENSG00000265671			antisense_RNA	2,357517999	-1,628702149	0,004627743	1
ENSG00000272115			antisense_RNA	6,119140897	-1,354167153	0,004778091	1
ENSG00000243779			processed_pseudogene	3,929702658	-1,368436746	0,004778521	1
ENSG00000163285	GABRG1	gamma-aminobutyric acid type A receptor gamma1 subunit [Source:HGNC Symbol;Acc:HGNC:4086]	protein_coding	245,152831	1,438855548	0,004907504	1
ENSG00000185736	ADARB2	adenosine deaminase, RNA specific B2 (inactive) [Source:HGNC Symbol;Acc:HGNC:227]	protein_coding	185,7293386	-1,474084428	0,00493461	1
ENSG00000263574			lincRNA	4,713265952	-1,620759269	0,004934764	1
ENSG00000279792			TEC	53,71859283	0,565663871	0,004973908	1
ENSG00000186676	EEF1GP1	eukaryotic translation elongation factor 1 gamma pseudogene 1 [Source:HGNC Symbol;Acc:HGNC:44556]	processed_pseudogene	19,27823452	-1,388245749	0,004980469	1
ENSG00000234721	LINC02092	long intergenic non-protein coding RNA 2092 [Source:HGNC Symbol;Acc:HGNC:52943]	lincRNA	8,135047849	-1,616195463	0,00499308	1
ENSG00000120729	MYOT	myotilin [Source:HGNC Symbol;Acc:HGNC:12399]	protein_coding	16,85072348	-1,616022585	0,005056851	1
ENSG00000211574	MIR770	microRNA 770 [Source:HGNC Symbol;Acc:HGNC:33143]	miRNA	5,405669714	1,580374148	0,00508718	1
ENSG00000253523			lincRNA	2,032368626	1,582633257	0,005099516	1
ENSG00000214295	FOXO1B	forkhead box O1B pseudogene [Source:HGNC Symbol;Acc:HGNC:3820]	processed_pseudogene	3,775866989	-1,607874335	0,005107947	1
ENSG00000225649			processed_transcript	52,78404899	1,4227353	0,005132232	1
ENSG00000237004	ZNRF2P1	zinc and ring finger 2 pseudogene 1 [Source:HGNC Symbol;Acc:HGNC:42792]	processed_pseudogene	37,26902198	-0,803925923	0,005145427	1
ENSG00000071991	CDH19	cadherin 19 [Source:HGNC Symbol;Acc:HGNC:1758]	protein_coding	26,91210799	-1,603331099	0,005176677	1
ENSG00000185739	SRL	sarcalumenin [Source:HGNC Symbol;Acc:HGNC:11295]	protein_coding	10,16590182	-1,375463408	0,005228532	1
ENSG00000259029	DUX4L18	double homeobox 4 like 18 [Source:HGNC Symbol;Acc:HGNC:37716]	unprocessed_pseudogene	2,162102087	-1,586631008	0,005237986	1
ENSG00000207955	MIR219A2	microRNA 219a-2 [Source:HGNC Symbol;Acc:HGNC:31598]	lincRNA	27,20453685	-1,523531986	0,0052964	1
ENSG00000198929	NOS1AP	nitric oxide synthase 1 adaptor protein [Source:HGNC Symbol;Acc:HGNC:16859]	protein_coding	259,4500393	0,854243083	0,005380394	1
ENSG00000234715			antisense_RNA	2,443362345	-1,595243016	0,00548958	1
ENSG00000239268			lincRNA	225,2947256	1,027692671	0,005614051	1
ENSG00000254501			antisense_RNA	10,32911623	1,3225068	0,005637287	1
ENSG00000147676	MAL2	mal, T-cell differentiation protein 2 (gene/pseudogene) [Source:HGNC Symbol;Acc:HGNC:13634]	protein_coding	238,7539748	0,917635382	0,005765518	1
ENSG00000210194	MT-TE	mitochondrially encoded tRNA glutamic acid [Source:HGNC Symbol;Acc:HGNC:7479]	Mt_tRNA	31,3990947	-1,270421834	0,005769649	1
ENSG00000170178	HOXD12	homeobox D12 [Source:HGNC Symbol;Acc:HGNC:5135]	protein_coding	3,565335987	-1,591032266	0,00578422	1
ENSG00000121101	TEX14	testis expressed 14, intercellular bridge forming factor [Source:HGNC Symbol;Acc:HGNC:11737]	protein_coding	23,68185946	-0,803778793	0,00581727	1
ENSG00000197889	MEIG1	meiosis/spermiogenesis associated 1 [Source:HGNC Symbol;Acc:HGNC:23429]	protein_coding	11,74098466	-0,964740774	0,005840087	1
ENSG00000138347	MYPN	myopalladin [Source:HGNC Symbol;Acc:HGNC:23246]	protein_coding	7,065610437	-1,499182831	0,005851649	1
ENSG00000186197	EDARADD	EDAR associated death domain [Source:HGNC Symbol;Acc:HGNC:14341]	protein_coding	17,17420557	1,220593619	0,005868854	1
ENSG00000108511	HOXB6	homeobox B6 [Source:HGNC Symbol;Acc:HGNC:5117]	protein_coding	4,183321691	-1,528328141	0,005896413	1
ENSG00000266643	MIR3677	microRNA 3677 [Source:HGNC Symbol;Acc:HGNC:38932]	miRNA	1,706168959	1,566438833	0,005966976	1
ENSG00000121858	TNFSF10	TNF superfamily member 10 [Source:HGNC Symbol;Acc:HGNC:11925]	protein_coding	6,530974228	-1,530349683	0,006043082	1
ENSG00000182107	TMEM30B	transmembrane protein 30B [Source:HGNC Symbol;Acc:HGNC:27254]	protein_coding	6,067809629	-1,57478555	0,006054295	1
ENSG00000223823	LINC01342	long intergenic non-protein coding RNA 1342 [Source:HGNC Symbol;Acc:HGNC:50551]	lincRNA	2,538611307	-1,580779123	0,006055519	1
ENSG00000075388	FGF4	fibroblast growth factor 4 [Source:HGNC Symbol;Acc:HGNC:3682]	protein_coding	7,03661306	-1,559103351	0,006093024	1
ENSG00000136379	ABHD17C	abhydrolase domain containing 17C [Source:HGNC Symbol;Acc:HGNC:26925]	protein_coding	247,7954242	0,655477823	0,006142355	1
ENSG00000256751	PLBD1-AS1	PLBD1 antisense RNA 1 [Source:HGNC Symbol;Acc:HGNC:51143]	antisense_RNA	3,33673935	-1,383155684	0,006182886	1
ENSG00000272135			transcribed_unprocessed_pseudogene	1,609356373	-1,55070048	0,006225319	1
ENSG00000278949			TEC	15,27775549	1,100337986	0,00625579	1
ENSG00000123407	HOXC12	homeobox C12 [Source:HGNC Symbol;Acc:HGNC:5124]	protein_coding	4,736141619	-1,557567021	0,006296841	1
ENSG00000105323	HNRNPUL1	heterogeneous nuclear ribonucleoprotein U like 1 [Source:HGNC Symbol;Acc:HGNC:17011]	protein_coding	3500,406419	-0,354358242	0,006310163	1
ENSG00000198624	CCDC69	coiled-coil domain containing 69 [Source:HGNC Symbol;Acc:HGNC:24487]	protein_coding	22,91648561	-1,0773767	0,006410425	1
ENSG00000269888			lincRNA	4,428117244	1,564855931	0,00641578	1
ENSG00000167693	NXN	nucleoredoxin [Source:HGNC Symbol;Acc:HGNC:18008]	protein_coding	1684,178023	-0,471725002	0,006432047	1
ENSG00000144227	NXPH2	neurexophilin 2 [Source:HGNC Symbol;Acc:HGNC:8076]	protein_coding	31,70543394	1,433083444	0,006447093	1
ENSG00000271888			lincRNA	6,709814753	-1,245308691	0,006467693	1
ENSG00000239264	TXNDC5	thioredoxin domain containing 5 [Source:HGNC Symbol;Acc:HGNC:21073]	protein_coding	46,49800987	-0,900040955	0,006507623	1
ENSG00000248767			protein_coding	2,102028556	-1,549016178	0,006543833	1
ENSG00000225556	C2CD4D	C2 calcium dependent domain containing 4D [Source:HGNC Symbol;Acc:HGNC:37210]	protein_coding	6,566640524	-1,452780796	0,006545255	1
ENSG00000202337	RNU6-8	RNA, U6 small nuclear 8 [Source:HGNC Symbol;Acc:HGNC:34285]	snRNA	13,80876932	-1,243130057	0,006600138	1
ENSG00000135577	NMBR	neuromedin B receptor [Source:HGNC Symbol;Acc:HGNC:7843]	protein_coding	22,2575633	-1,511915927	0,006611303	1
ENSG00000012061	ERCC1	ERCC excision repair 1, endonuclease non-catalytic subunit [Source:HGNC Symbol;Acc:HGNC:3433]	protein_coding	1499,581321	-0,304664512	0,00663672	1
ENSG00000177340		uncharacterized LOC79857 [Source:NCBI gene;Acc:79857]	antisense_RNA	7,665264255	-1,338509549	0,006697115	1
ENSG00000259948			processed_pseudogene	9,180394926	1,352125921	0,006719776	1
ENSG00000274751			antisense_RNA	6,222827567	-1,521706135	0,006748367	1

ENSG00000172594	SMPDL3A	sphingomyelin phosphodiesterase acid like 3A [Source:HGNC Symbol;Acc:HGNC:17389]	protein_coding	193,5195645	-0,522470273	0,006764451	1
ENSG00000125734	GPR108	G protein-coupled receptor 108 [Source:HGNC Symbol;Acc:HGNC:17829]	protein_coding	560,2863878	-0,486339208	0,006804878	1
ENSG00000188282	RUFY4	RUN and FYVE domain containing 4 [Source:HGNC Symbol;Acc:HGNC:24804]	protein_coding	7,166475425	-1,490975638	0,006929431	1
ENSG00000101977	MCF2,00	MCF.2 cell line derived transforming sequence [Source:HGNC Symbol;Acc:HGNC:6940]	protein_coding	125,7270997	0,637626107	0,006970508	1
ENSG00000120332	TNN	tenascin N [Source:HGNC Symbol;Acc:HGNC:22942]	protein_coding	9,421590087	-1,384624872	0,006989729	1
ENSG00000237750			antisense_RNA	3,289163809	1,49446593	0,007027538	1
ENSG00000279636		long intergenic non-protein coding RNA 216 [Source:NCBI gene;Acc:55451]	lincRNA	3,888030705	1,390373624	0,007042435	1
ENSG00000103534	TMC5	transmembrane channel like 5 [Source:HGNC Symbol;Acc:HGNC:22999]	protein_coding	11,67996175	-1,437474439	0,007094976	1
ENSG00000260878			sense_overlapping	21,90700167	1,096334362	0,007095942	1
ENSG00000259424			antisense_RNA	3,602815061	-1,393344207	0,007105158	1
ENSG00000168612	ZSWIM1	zinc finger SWIM-type containing 1 [Source:HGNC Symbol;Acc:HGNC:16155]	protein_coding	260,8525328	-0,346057992	0,007133864	1
ENSG00000204588	LINC01123	long intergenic non-protein coding RNA 1123 [Source:HGNC Symbol;Acc:HGNC:49269]	lincRNA	12,44418818	1,39037724	0,007152961	1
ENSG00000260001	TGFBR3L	transforming growth factor beta receptor 3 like [Source:HGNC Symbol;Acc:HGNC:44152]	protein_coding	8,749015525	-1,308585933	0,007153045	1
ENSG00000231381	RNF2P1	ring finger protein 2 pseudogene 1 [Source:HGNC Symbol;Acc:HGNC:33987]	processed_pseudogene	1,521952957	1,545176905	0,007179309	1
ENSG00000101457	DNTTIP1	deoxynucleotidyltransferase terminal interacting protein 1 [Source:HGNC Symbol;Acc:HGNC:16160]	protein_coding	203,948795	0,952905693	0,007213388	1
ENSG00000189275	LINC01164	long intergenic non-protein coding RNA 1164 [Source:HGNC Symbol;Acc:HGNC:49533]	lincRNA	3,772728056	-1,505749303	0,00723475	1
ENSG00000188580	NKAIN2	sodium/potassium transporting ATPase interacting 2 [Source:HGNC Symbol;Acc:HGNC:16443]	protein_coding	458,9943264	1,197262257	0,007266607	1
ENSG00000205002	AARD	alanine and arginine rich domain containing protein [Source:HGNC Symbol;Acc:HGNC:33842]	protein_coding	12,16502917	-1,013349192	0,007268277	1
ENSG00000183463	URAD	ureidoimidazoline (2-oxo-4-hydroxy-4-carboxy-5-) decarboxylase [Source:HGNC Symbol;Acc:HGNC:17785]	protein_coding	2,815403922	-1,535776595	0,007334836	1
ENSG00000213380	COG8	component of oligomeric golgi complex 8 [Source:HGNC Symbol;Acc:HGNC:18623]	protein_coding	391,4017878	0,312861257	0,0073542	1
ENSG00000206028			lincRNA	49,90729519	1,14879158	0,007404865	1
ENSG00000277914	DDX6P2	DEAD-box helicase 6 pseudogene 2 [Source:HGNC Symbol;Acc:HGNC:37806]	processed_pseudogene	2,436991735	-1,531199508	0,007446579	1
ENSG00000106004	HOXA5	homeobox A5 [Source:HGNC Symbol;Acc:HGNC:5106]	protein_coding	3,089687038	-1,538980547	0,007478127	1
ENSG00000105996	HOXA2	homeobox A2 [Source:HGNC Symbol;Acc:HGNC:5103]	protein_coding	5,271270648	-1,538517587	0,007614345	1
ENSG00000180105			processed_pseudogene	1,951077079	-1,526305226	0,007758658	1
ENSG00000180229	HERC2P3	hect domain and RLD 2 pseudogene 3 [Source:HGNC Symbol;Acc:HGNC:4871]	transcribed_unprocessed_pseudogene	90,71366626	-1,251872271	0,007827054	1
ENSG00000249971			antisense_RNA	4,630568142	1,514168032	0,007837454	1
ENSG00000065923	SLC9A7	solute carrier family 9 member A7 [Source:HGNC Symbol;Acc:HGNC:17123]	protein_coding	1407,701265	0,525766485	0,007840618	1
ENSG00000282872	C1orf232	chromosome 1 open reading frame 230 [Source:HGNC Symbol;Acc:HGNC:53426]	protein_coding	5,154112698	-1,491071947	0,007852008	1
ENSG00000166736	HTR3A	5-hydroxytryptamine receptor 3A [Source:HGNC Symbol;Acc:HGNC:5297]	protein_coding	4,335151563	-1,51730956	0,007853834	1
ENSG00000257038			antisense_RNA	12,15758767	-1,18732293	0,007961853	1
ENSG00000228170			antisense_RNA	21,78372578	0,930950146	0,007998672	1
ENSG00000200832	SNORD114-4	small nucleolar RNA, C/D box 114-4 [Source:HGNC Symbol;Acc:HGNC:32992]	snoRNA	6,169888666	1,45055555	0,008310279	1
ENSG00000225339			processed_transcript	228,9013123	1,167128381	0,00833104	1
ENSG00000278239			lincRNA	2,594858693	-1,519847618	0,008375976	1
ENSG00000215003	RPL15P20		processed_pseudogene	1,796260386	1,480543134	0,008405954	1
ENSG00000017427	IGF1	insulin like growth factor 1 [Source:HGNC Symbol;Acc:HGNC:5464]	protein_coding	103,5864168	1,512759059	0,008477896	1
ENSG00000232063			lincRNA	5,71145072	-1,511519291	0,008518539	1
ENSG00000131018	SYNE1	spectrin repeat containing nuclear envelope protein 1 [Source:HGNC Symbol;Acc:HGNC:17089]	protein_coding	3013,246637	0,467097695	0,008522314	1
ENSG00000237356			processed_transcript	6,962453166	-1,386444716	0,008538256	1
ENSG00000157110	RBPMS	RNA binding protein with multiple splicing [Source:HGNC Symbol;Acc:HGNC:19097]	protein_coding	36,92021092	-1,149753855	0,008539626	1
ENSG00000141499	WRAP53	WD repeat containing antisense to TP53 [Source:HGNC Symbol;Acc:HGNC:25522]	protein_coding	368,1590203	-0,373918089	0,008592358	1
ENSG00000174059	CD34	CD34 molecule [Source:HGNC Symbol;Acc:HGNC:1662]	protein_coding	102,2414577	-1,405895991	0,008593725	1
ENSG00000279586			TEC	6,279642025	1,145166704	0,008635401	1
ENSG00000259855			lincRNA	3,209637721	-1,488132324	0,008709848	1
ENSG00000196071	OR2L13	olfactory receptor family 2 subfamily L member 13 [Source:HGNC Symbol;Acc:HGNC:19578]	protein_coding	11,52229656	-1,417728188	0,008809983	1
ENSG00000065361	ERBB3	erb-b2 receptor tyrosine kinase 3 [Source:HGNC Symbol;Acc:HGNC:3431]	protein_coding	53,45872745	-1,012179689	0,00881298	1
ENSG00000180053	NKX2-6	NK2 homeobox 6 [Source:HGNC Symbol;Acc:HGNC:32940]	protein_coding	2,016276341	-1,508853076	0,00882244	1
ENSG00000125510	OPRL1	opioid related nociceptin receptor 1 [Source:HGNC Symbol;Acc:HGNC:8155]	protein_coding	187,2566302	0,714366347	0,008835147	1
ENSG00000057468	MSH4	mutS homolog 4 [Source:HGNC Symbol;Acc:HGNC:7327]	protein_coding	32,07320235	1,142475143	0,008909616	1
ENSG00000077274	CAPN6	calpain 6 [Source:HGNC Symbol;Acc:HGNC:1483]	protein_coding	8,480868919	-1,116696518	0,008930201	1
ENSG00000110693	SOX6	SRY-box 6 [Source:HGNC Symbol;Acc:HGNC:16421]	protein_coding	1536,933644	1,072930946	0,008938348	1
ENSG00000104972	LILRB1	leukocyte immunoglobulin like receptor B1 [Source:HGNC Symbol;Acc:HGNC:6605]	protein_coding	3,896376241	-1,497610038	0,009063812	1
ENSG00000175868	CALCB	calcitonin related polypeptide beta [Source:HGNC Symbol;Acc:HGNC:1438]	protein_coding	8,65196785	-1,467706114	0,009123568	1
ENSG00000233608	TWIST2	twist family bHLH transcription factor 2 [Source:HGNC Symbol;Acc:HGNC:20670]	protein_coding	13,24100079	-1,474385889	0,009189432	1
ENSG00000124780	KCNK17	potassium two pore domain channel subfamily K member 17 [Source:HGNC Symbol;Acc:HGNC:14465]	protein_coding	3,149766906	-1,4997625	0,00926109	1
ENSG00000139323	POC1B	POC1 centriolar protein B [Source:HGNC Symbol;Acc:HGNC:30836]	protein_coding	309,0859761	-0,429386508	0,009350963	1
ENSG00000198353	HOXC4	homeobox C4 [Source:HGNC Symbol;Acc:HGNC:5126]	protein_coding	2,653389696	-1,493803088	0,00942854	1
ENSG00000260111			antisense_RNA	10,34639256	1,009477606	0,009440902	1
ENSG00000149635	OCSTAMP	osteoclast stimulatory transmembrane protein [Source:HGNC Symbol;Acc:HGNC:16116]	protein_coding	3,637441292	-1,385554679	0,009483508	1
ENSG00000247033			antisense_RNA	11,3807782	1,181835317	0,009541856	1
ENSG00000204252	HLA-DOA	major histocompatibility complex, class II, DO alpha [Source:HGNC Symbol;Acc:HGNC:4936]	protein_coding	35,16848401	-1,475459825	0,009546028	1
ENSG00000166828	SCNN1G	sodium channel epithelial 1 gamma subunit [Source:HGNC Symbol;Acc:HGNC:10602]	protein_coding	24,3004104	1,417367601	0,00956648	1
ENSG00000200367	SNORD113-8	small nucleolar RNA, C/D box 113-8 [Source:HGNC Symbol;Acc:HGNC:32987]	snoRNA	3,418778164	1,378926984	0,009594767	1
ENSG00000152804	HHEX	hematopoietically expressed homeobox [Source:HGNC Symbol;Acc:HGNC:4901]	protein_coding	3,529214779	-1,454872862	0,009687171	1
ENSG00000232581			antisense_RNA	5,302166051	1,414459779	0,009737277	1
ENSG00000140678	ITGAX	integrin subunit alpha X [Source:HGNC Symbol;Acc:HGNC:6152]	protein_coding	13,14164927	-1,455719897	0,009763253	1

ENSG00000171517	LPAR3	lysophosphatidic acid receptor 3 [Source:HGNC Symbol;Acc:HGNC:14298]	protein_coding	11,93134683	-1,292757344	0,009765247	1
ENSG00000197408	CYP2B6	cytochrome P450 family 2 subfamily B member 6 [Source:HGNC Symbol;Acc:HGNC:2615]	protein_coding	8,244546678	-1,47031231	0,009774932	1
ENSG00000140798	ABCC12	ATP binding cassette subfamily C member 12 [Source:HGNC Symbol;Acc:HGNC:14640]	protein_coding	4,510360523	-1,480599465	0,009795239	1
ENSG00000163288	GABRB1	gamma-aminobutyric acid type A receptor beta1 subunit [Source:HGNC Symbol;Acc:HGNC:4081]	protein_coding	88,24766506	0,985576945	0,009796188	1
ENSG00000163016	ALMS1P1	ALMS1, centrosome and basal body associated protein pseudogene 1 [Source:HGNC Symbol;Acc:HGNC:29586]	transcribed_unprocessed_pseudogene	21,11390181	0,974422014	0,009817536	1
ENSG00000120068	HOXB8	homeobox B8 [Source:HGNC Symbol;Acc:HGNC:5119]	protein_coding	7,978255855	-1,488561277	0,009820094	1
ENSG00000183770	FOXL2	forkhead box L2 [Source:HGNC Symbol;Acc:HGNC:1092]	protein_coding	11,54566436	-1,319683061	0,009823031	1
ENSG00000134470	IL15RA	interleukin 15 receptor subunit alpha [Source:HGNC Symbol;Acc:HGNC:5978]	protein_coding	6,976763027	-1,461724562	0,009951047	1
ENSG00000165807	PPP1R36	protein phosphatase 1 regulatory subunit 36 [Source:HGNC Symbol;Acc:HGNC:20097]	protein_coding	13,1658309	-0,804370279	0,010018321	1
ENSG00000188585	CLEC20A	C-type lectin domain containing 20A [Source:HGNC Symbol;Acc:HGNC:34521]	protein_coding	3,72632077	-1,472822842	0,010043004	1
ENSG00000236017	ASMTL-AS1	ASMTL antisense RNA 1 [Source:HGNC Symbol;Acc:HGNC:25811]	antisense_RNA	16,8801568	-0,900952156	0,010119701	1
ENSG00000157119	KLHL40	kelch like family member 40 [Source:HGNC Symbol;Acc:HGNC:30372]	protein_coding	2,690177817	-1,478761514	0,010269283	1
ENSG00000254497			antisense_RNA	3,807217193	1,471154814	0,010275306	1
ENSG00000279022			TEC	13,68413148	1,311916229	0,01031517	1
ENSG00000145708	CRHBP	corticotropin releasing hormone binding protein [Source:HGNC Symbol;Acc:HGNC:2356]	protein_coding	132,6409315	1,413393758	0,010338348	1
ENSG00000180448	ARHGAP45	Rho GTPase activating protein 45 [Source:HGNC Symbol;Acc:HGNC:17102]	protein_coding	40,48118487	-1,268725332	0,010441685	1
ENSG00000275632			lincRNA	41,00229047	-0,784368842	0,010558011	1
ENSG00000215263			processed_pseudogene	1,955327917	-1,455400127	0,010572915	1
ENSG00000225811			antisense_RNA	2,72044061	1,433630238	0,010594475	1
ENSG00000163082	SGPP2	sphingosine-1-phosphate phosphatase 2 [Source:HGNC Symbol;Acc:HGNC:19953]	protein_coding	73,39575242	-1,470110199	0,010601777	1
ENSG00000152217	SETBP1	SET binding protein 1 [Source:HGNC Symbol;Acc:HGNC:15573]	protein_coding	2906,25958	0,439174789	0,010604374	1
ENSG00000224769	MUC20P1	mucin 20, cell surface associated pseudogene 1 [Source:HGNC Symbol;Acc:HGNC:51921]	unprocessed_pseudogene	7,887330171	1,440001887	0,010688026	1
ENSG00000177465	ACOT4	acyl-CoA thioesterase 4 [Source:HGNC Symbol;Acc:HGNC:19748]	protein_coding	8,231922741	-1,227876965	0,01070297	1
ENSG00000130948	HSD17B3	hydroxysteroid 17-beta dehydrogenase 3 [Source:HGNC Symbol;Acc:HGNC:5212]	protein_coding	4,362125084	-1,214731659	0,01072368	1
ENSG00000053524	MCF2L2	MCF.2 cell line derived transforming sequence-like 2 [Source:HGNC Symbol;Acc:HGNC:30319]	protein_coding	360,3501443	0,6482129	0,010730144	1
ENSG00000257941			processed_pseudogene	4,467849391	1,351586482	0,010750864	1
ENSG00000100319	ZMAT5	zinc finger matrin-type 5 [Source:HGNC Symbol;Acc:HGNC:28046]	protein_coding	518,9443891	-0,381047459	0,010956078	1
ENSG00000267334			lincRNA	6,710584758	1,036677423	0,011074299	1
ENSG00000274825			lincRNA	5,840741506	-1,24944806	0,011081769	1
ENSG00000235768	BRD7P5	bromodomain containing 7 pseudogene 5 [Source:HGNC Symbol;Acc:HGNC:37631]	processed_pseudogene	3,75223301	-1,459711675	0,011082072	1
ENSG00000145416	MARCH1	membrane associated ring-CH-type finger 1 [Source:HGNC Symbol;Acc:HGNC:26077]	protein_coding	771,6540012	0,690090341	0,011087558	1
ENSG00000213889	PPM1N	protein phosphatase, Mg2+/Mn2+ dependent 1N (putative) [Source:HGNC Symbol;Acc:HGNC:26845]	protein_coding	17,73138734	-1,000108839	0,011099387	1
ENSG00000006016	CRLF1	cytokine receptor like factor 1 [Source:HGNC Symbol;Acc:HGNC:2364]	protein_coding	83,51124832	-1,222120127	0,011134867	1
ENSG00000213644	SAPCD2P1	suppressor APC domain containing 2 pseudogene 1 [Source:HGNC Symbol;Acc:HGNC:51277]	processed_pseudogene	2,972552548	-1,46003704	0,011248948	1
ENSG00000241213	LINC02024	long intergenic non-protein coding RNA 2024 [Source:HGNC Symbol;Acc:HGNC:52859]	lincRNA	29,63131403	1,221861697	0,011339718	1
ENSG00000258418			lincRNA	2,57237158	1,362712016	0,011437713	1
ENSG00000260742			antisense_RNA	6,31222001	-1,26208204	0,011456686	1
ENSG00000112294	ALDH5A1	aldehyde dehydrogenase 5 family member A1 [Source:HGNC Symbol;Acc:HGNC:408]	protein_coding	1021,744683	0,527606109	0,011463766	1
ENSG00000260618			antisense_RNA	6,46645856	1,356502418	0,011638094	1
ENSG00000276564			lincRNA	97,07243184	0,599913895	0,011717663	1
ENSG00000279107			TEC	6,326765352	-1,076021323	0,011760376	1
ENSG00000169860	P2RY1	purinergic receptor P2Y1 [Source:HGNC Symbol;Acc:HGNC:8539]	protein_coding	410,5276492	0,986873207	0,011770233	1
ENSG00000269834	ZNF528-AS1	ZNF528 antisense RNA 1 [Source:HGNC Symbol;Acc:HGNC:51305]	processed_transcript	294,4516965	1,220686662	0,011776472	1
ENSG00000182687	GALR2	galanin receptor 2 [Source:HGNC Symbol;Acc:HGNC:4133]	protein_coding	3,421062446	-1,444671413	0,01178102	1
ENSG00000203808	BVES-AS1	BVES antisense RNA 1 [Source:HGNC Symbol;Acc:HGNC:21223]	antisense_RNA	6,504847359	-1,330063279	0,011910192	1
ENSG00000254328			processed_pseudogene	7,705371156	1,026124299	0,011994177	1
ENSG00000164438	TLX3	T-cell leukemia homeobox 3 [Source:HGNC Symbol;Acc:HGNC:13532]	protein_coding	8,878202741	-1,446489252	0,01199501	1
ENSG00000181218	HIST3H2A	histone cluster 3 H2A [Source:HGNC Symbol;Acc:HGNC:20507]	protein_coding	895,7975245	-0,732518818	0,01204803	1
ENSG00000261093			antisense_RNA	11,68648865	1,19360761	0,012072894	1
ENSG00000271941			antisense_RNA	14,77204475	0,825956161	0,012125628	1
ENSG00000064195	DLX3	distal-less homeobox 3 [Source:HGNC Symbol;Acc:HGNC:2916]	protein_coding	4,303760828	-1,434558427	0,01215716	1
ENSG00000267469			antisense_RNA	79,84926908	1,352400275	0,012170633	1
ENSG00000197428	OR51D1	olfactory receptor family 51 subfamily D member 1 [Source:HGNC Symbol;Acc:HGNC:15193]	protein_coding	3,59772887	-1,442538833	0,012220935	1
ENSG00000149596	JPH2	junctophilin 2 [Source:HGNC Symbol;Acc:HGNC:14202]	protein_coding	23,91312246	-1,383685161	0,01224821	1
ENSG00000268416			lincRNA	5,112482523	-1,44031858	0,012293677	1
ENSG00000114648	KLHL18	kelch like family member 18 [Source:HGNC Symbol;Acc:HGNC:29120]	protein_coding	1070,47297	0,293716579	0,012547355	1
ENSG00000171116	HSFX1	heat shock transcription factor family, X-linked 1 [Source:HGNC Symbol;Acc:HGNC:29603]	protein_coding	2,322505983	1,434399515	0,012556918	1
ENSG00000229106	BTBD6P1	BTB domain containing 6 pseudogene 1 [Source:HGNC Symbol;Acc:HGNC:51542]	processed_pseudogene	1,888714435	-1,402595094	0,01257443	1
ENSG00000256218			lincRNA	3,27520149	-1,43202818	0,012580047	1
ENSG00000105664	COMP	cartilage oligomeric matrix protein [Source:HGNC Symbol;Acc:HGNC:2227]	protein_coding	5,846948284	-1,421833103	0,012607996	1
ENSG00000259678			antisense_RNA	8,636359362	1,278082833	0,012712104	1
ENSG00000102539	MLNR	motilin receptor [Source:HGNC Symbol;Acc:HGNC:4495]	protein_coding	6,655654248	-1,43110301	0,012762622	1
ENSG00000229931			antisense_RNA	15,22569377	1,019155855	0,012776669	1
ENSG00000265664			antisense_RNA	2,833844483	1,418481609	0,012806905	1
ENSG00000223911			antisense_RNA	18,55545162	1,348326142	0,012851437	1
ENSG00000272854			antisense_RNA	2,808460531	-1,289822843	0,012912678	1
ENSG00000174137	FAM53A	family with sequence similarity 53 member A [Source:HGNC Symbol;Acc:HGNC:31860]	protein_coding	26,14608006	-0,894041286	0,013055278	1

ENSG00000266872			antisense_RNA	3,264142476	1,417608116	0,013133038	1
ENSG00000225670	CADM3-AS1	CADM3 antisense RNA 1 [Source:HGNC Symbol;Acc:HGNC:40812]	antisense_RNA	62,9541353	1,279020836	0,013157223	1
ENSG00000280237	MIR4697HG	MIR4697 host gene [Source:HGNC Symbol;Acc:HGNC:27448]	TEC	103,1875264	0,679030395	0,013178835	1
ENSG00000272732			lincRNA	18,70263067	1,038795617	0,013249405	1
ENSG00000258861	MIR381HG	MIR381 host gene [Source:HGNC Symbol;Acc:HGNC:20136]	lincRNA	5,405813329	1,371977222	0,013317349	1
ENSG00000171368	TPPP	tubulin polymerization promoting protein [Source:HGNC Symbol;Acc:HGNC:24164]	protein_coding	699,4885952	0,726842422	0,013370387	1
ENSG00000238083	LRRC37A2	leucine rich repeat containing 37 member A2 [Source:HGNC Symbol;Acc:HGNC:32404]	protein_coding	56,02909505	0,691006899	0,013449339	1
ENSG00000263278			sense_intronic	11,84710152	1,073473296	0,013480643	1
ENSG00000166391	MOGAT2	monoacylglycerol O-acyltransferase 2 [Source:HGNC Symbol;Acc:HGNC:23248]	protein_coding	4,956664826	-1,381445538	0,013636287	1
ENSG00000160447	PKN3	protein kinase N3 [Source:HGNC Symbol;Acc:HGNC:17999]	protein_coding	151,8061995	-0,719166529	0,013783969	1
ENSG00000272447			lincRNA	138,8440941	-0,629899153	0,013813561	1
ENSG00000257319			antisense_RNA	2,72467379	-1,419078015	0,013837629	1
ENSG00000185565	LSAMP	limbic system-associated membrane protein [Source:HGNC Symbol;Acc:HGNC:6705]	protein_coding	4157,974742	0,73985095	0,01397249	1
ENSG00000255750			antisense_RNA	6,085025634	1,372775997	0,013973192	1
ENSG00000071564	TCF3	transcription factor 3 [Source:HGNC Symbol;Acc:HGNC:11633]	protein_coding	2867,823494	-0,450678899	0,014056994	1
ENSG00000232085			antisense_RNA	3,291027025	1,339194668	0,014082786	1
ENSG00000187944	C2orf66	chromosome 2 open reading frame 66 [Source:HGNC Symbol;Acc:HGNC:33809]	protein_coding	17,30707752	1,157249897	0,014083726	1
ENSG00000206203	TSSK2	testis specific serine kinase 2 [Source:HGNC Symbol;Acc:HGNC:11401]	protein_coding	4,149777546	1,385295431	0,014100879	1
ENSG00000132938	MTUS2	microtubule associated scaffold protein 2 [Source:HGNC Symbol;Acc:HGNC:20595]	protein_coding	212,0581878	1,083877095	0,014132889	1
ENSG00000142233	NTN5	netrin 5 [Source:HGNC Symbol;Acc:HGNC:25208]	protein_coding	7,348236047	-1,262798534	0,014147435	1
ENSG00000257270			antisense_RNA	4,319547389	1,298927462	0,014152809	1
ENSG00000234312	SAPCD2P4	suppressor APC domain containing 2 pseudogene 4 [Source:HGNC Symbol;Acc:HGNC:51280]	processed_pseudogene	2,124046993	-1,409012553	0,014238248	1
ENSG00000255164			antisense_RNA	3,662979094	1,408907666	0,014257454	1
ENSG00000255020			antisense_RNA	2,593914738	-1,389996389	0,014387801	1
ENSG00000175643	RMI2	RecQ mediated genome instability 2 [Source:HGNC Symbol;Acc:HGNC:28349]	protein_coding	92,13715721	-0,763503654	0,014424884	1
ENSG00000230982	DSTNP1	destrin, actin depolymerizing factor pseudogene 1 [Source:HGNC Symbol;Acc:HGNC:23769]	processed_pseudogene	7,841149549	1,23381948	0,014444177	1
ENSG00000104848	KCNA7	potassium voltage-gated channel subfamily A member 7 [Source:HGNC Symbol;Acc:HGNC:6226]	protein_coding	11,64938357	-1,338091965	0,014480046	1
ENSG00000183784	C9orf66	chromosome 9 open reading frame 66 [Source:HGNC Symbol;Acc:HGNC:26436]	protein_coding	6,294758537	-1,273738255	0,014488764	1
ENSG00000107821	KAZALD1	Kazal type serine peptidase inhibitor domain 1 [Source:HGNC Symbol;Acc:HGNC:25460]	protein_coding	79,90088019	-1,179591906	0,014515726	1
ENSG00000201229	SNORA63D	small nucleolar RNA, H/ACA box 63D [Source:HGNC Symbol;Acc:HGNC:52211]	snoRNA	39,98340079	0,794371609	0,0145651	1
ENSG00000172005	MAL	mal, T-cell differentiation protein [Source:HGNC Symbol;Acc:HGNC:6817]	protein_coding	17,48213287	-1,333763065	0,014565638	1
ENSG00000164442	CITED2	Cbp/p300 interacting transactivator with Glu/Asp rich carboxy-terminal domain 2 [Source:HGNC Symbol;Acc:HGNC:19	protein_coding	1968,169498	-0,743457414	0,01458922	1
ENSG00000284606			processed_transcript	14,54997977	1,080397355	0,014593589	1
ENSG00000214866	DCDC2C	doublecortin domain containing 2C [Source:HGNC Symbol;Acc:HGNC:32696]	protein_coding	4,570440238	-1,400492619	0,014594283	1
ENSG00000269720	CCDC194	coiled-coil domain containing 194 [Source:HGNC Symbol;Acc:HGNC:53438]	protein_coding	5,718738143	-1,384117385	0,014618068	1
ENSG00000205277	MUC12	mucin 12, cell surface associated [Source:HGNC Symbol;Acc:HGNC:7510]	protein_coding	16,09057653	-1,227253283	0,014649611	1
ENSG00000170613	FAM71B	family with sequence similarity 71 member B [Source:HGNC Symbol;Acc:HGNC:28397]	protein_coding	1,921587241	-1,39862494	0,014657924	1
ENSG00000171757	LRRC34	leucine rich repeat containing 34 [Source:HGNC Symbol;Acc:HGNC:28408]	protein_coding	57,38439397	-0,967403316	0,014661161	1
ENSG00000165061	ZMAT4	zinc finger matrin-type 4 [Source:HGNC Symbol;Acc:HGNC:25844]	protein_coding	217,9362203	0,971331113	0,014665723	1
ENSG00000213036			processed_pseudogene	1,521514353	-1,396927222	0,014672811	1
ENSG00000271776			antisense_RNA	3,37344644	-1,403450439	0,01473171	1
ENSG00000137868	STRA6	stimulated by retinoic acid 6 [Source:HGNC Symbol;Acc:HGNC:30650]	protein_coding	56,76568768	-1,358688286	0,014767845	1
ENSG00000222162	RN7SKP151	RNA, 7SK small nuclear pseudogene 151 [Source:HGNC Symbol;Acc:HGNC:45875]	misc_RNA	2,585529756	1,288357214	0,014889545	1
ENSG00000121931	LRIF1	ligand dependent nuclear receptor interacting factor 1 [Source:HGNC Symbol;Acc:HGNC:30299]	protein_coding	593,9419112	-0,282238108	0,014901858	1
ENSG00000154928	EPHB1	EPH receptor B1 [Source:HGNC Symbol;Acc:HGNC:3392]	protein_coding	429,2224737	0,716980924	0,014942872	1
ENSG00000162885	B3GALNT2	beta-1,3-N-acetylgalactosaminyltransferase 2 [Source:HGNC Symbol;Acc:HGNC:28596]	protein_coding	356,9162106	-0,509521306	0,014975583	1
ENSG00000196391	ZNF774	zinc finger protein 774 [Source:HGNC Symbol;Acc:HGNC:33108]	protein_coding	124,8825912	0,438488566	0,015025759	1
ENSG00000196890	HIST3H2BB	histone cluster 3 H2B family member b [Source:HGNC Symbol;Acc:HGNC:20514]	protein_coding	443,2249996	-0,904355018	0,015044744	1
ENSG00000167676	PLIN4	perilipin 4 [Source:HGNC Symbol;Acc:HGNC:29393]	protein_coding	31,0088605	1,179553634	0,015046141	1
ENSG00000259685	IDH2-DT	IDH2 divergent transcript [Source:HGNC Symbol;Acc:HGNC:53154]	lincRNA	2,773293547	-1,397306716	0,015068566	1
ENSG00000268729			antisense_RNA	2,083884386	1,391689631	0,01508194	1
ENSG00000102048	ASB9	ankyrin repeat and SOCS box containing 9 [Source:HGNC Symbol;Acc:HGNC:17184]	protein_coding	3,433338441	-1,396098186	0,015092794	1
ENSG00000130943	PKDREJ	polycystin family receptor for egg jelly [Source:HGNC Symbol;Acc:HGNC:9015]	protein_coding	7,254638835	-1,297183412	0,015225605	1
ENSG00000250846	EPHA5-AS1	EPHA5 antisense RNA 1 [Source:HGNC Symbol;Acc:HGNC:50602]	lincRNA	56,70449068	0,90962216	0,015238313	1
ENSG00000261371	PECAM1	platelet and endothelial cell adhesion molecule 1 [Source:HGNC Symbol;Acc:HGNC:8823]	protein_coding	19,81357937	-1,345403833	0,015360535	1
ENSG00000253167	WASHC5-AS1	WASHC5 antisense RNA 1 [Source:HGNC Symbol;Acc:HGNC:43440]	antisense_RNA	4,312740777	1,315043978	0,01544958	1
ENSG00000154917	RAB6B	RAB6B, member RAS oncogene family [Source:HGNC Symbol;Acc:HGNC:14902]	protein_coding	1506,508661	0,572926589	0,015461921	1
ENSG00000167757	KLK11	kallikrein related peptidase 11 [Source:HGNC Symbol;Acc:HGNC:6359]	protein_coding	6,705901178	-1,385307645	0,015520262	1
ENSG00000176681	LRRC37A	leucine rich repeat containing 37A [Source:HGNC Symbol;Acc:HGNC:29069]	protein_coding	8,591932252	1,112644644	0,015542592	1
ENSG00000248265		uncharacterized LOC440101 [Source:NCBI gene;Acc:440101]	lincRNA	4,851030483	-1,369213644	0,01554453	1
ENSG00000075891	PAX2	paired box 2 [Source:HGNC Symbol;Acc:HGNC:8616]	protein_coding	12,26432277	-1,389762541	0,015566229	1
ENSG00000157654	PALM2-AKAP2	PALM2-AKAP2 readthrough [Source:HGNC Symbol;Acc:HGNC:33529]	protein_coding	1,125255579	-1,261617154	0,015580536	1
ENSG00000266441			antisense_RNA	10,51281557	-0,968722142	0,015588177	1
ENSG00000254987			lincRNA	4,911578351	1,392437524	0,015626378	1
ENSG00000153029	MR1	major histocompatibility complex, class I-related [Source:HGNC Symbol;Acc:HGNC:4975]	protein_coding	17,29400392	-0,990755366	0,015713968	1
ENSG00000170927	PKHD1	PKHD1, fibrocystin/polyductin [Source:HGNC Symbol;Acc:HGNC:9016]	protein_coding	23,24061034	-1,373038101	0,015736028	1
ENSG00000115194	SLC30A3	solute carrier family 30 member 3 [Source:HGNC Symbol;Acc:HGNC:11014]	protein_coding	12,63475408	-1,285067567	0,015757433	1

ENSG00000113262	GRM6	glutamate metabotropic receptor 6 [Source:HGNC Symbol;Acc:HGNC:4598]	protein_coding	10,40233396	-1,368885777	0,015788992	1
ENSG00000149646	CNBD2	cyclic nucleotide binding domain containing 2 [Source:HGNC Symbol;Acc:HGNC:16145]	protein_coding	70,76001394	-0,563137866	0,015828145	1
ENSG00000270332	SMC2-AS1	SMC2 antisense RNA 1 (head to head) [Source:HGNC Symbol;Acc:HGNC:50827]	lincRNA	9,493709304	-1,000938944	0,015868755	1
ENSG00000272690	LINC02018	long intergenic non-protein coding RNA 2018 [Source:HGNC Symbol;Acc:HGNC:52853]	lincRNA	22,36462917	0,78712522	0,01587257	1
ENSG00000201700	SNORD113-3	small nucleolar RNA, C/D box 113-3 [Source:HGNC Symbol;Acc:HGNC:32982]	snoRNA	2,616246601	1,266083596	0,015879125	1
ENSG00000283556	MIR376B	microRNA 376b [Source:HGNC Symbol;Acc:HGNC:32066]	miRNA	2,592077376	1,306332047	0,015918658	1
ENSG00000225746	MEG8	maternally expressed 8, small nucleolar RNA host gene [Source:HGNC Symbol;Acc:HGNC:14574]	lincRNA	502,327707	1,343576458	0,015923811	1
ENSG00000253626	EIF5AL1	eukaryotic translation initiation factor 5A-like 1 [Source:HGNC Symbol;Acc:HGNC:17419]	protein_coding	11,07351486	1,310924925	0,015926511	1
ENSG00000176243	CDV3P1	CDV3 pseudogene 1 [Source:HGNC Symbol;Acc:HGNC:42387]	processed_pseudogene	1,357448259	-1,366980815	0,016038217	1
ENSG00000259661			antisense_RNA	9,619855329	1,252315441	0,016096534	1
ENSG00000078246	TULP3	tubby like protein 3 [Source:HGNC Symbol;Acc:HGNC:12425]	protein_coding	659,2637546	-0,42848026	0,01611703	1
ENSG00000253347			antisense_RNA	9,258482611	-1,06464542	0,016136119	1
ENSG00000267265			antisense_RNA	28,90328282	1,041819113	0,016148034	1
ENSG00000213592	PPIAP42	peptidylprolyl isomerase A pseudogene 42 [Source:HGNC Symbol;Acc:HGNC:53666]	processed_pseudogene	1,514109631	-1,384898293	0,016217186	1
ENSG00000152315	KCNK13	potassium two pore domain channel subfamily K member 13 [Source:HGNC Symbol;Acc:HGNC:6275]	protein_coding	15,0674446	-1,293058693	0,016224801	1
ENSG00000012124	CD22	CD22 molecule [Source:HGNC Symbol;Acc:HGNC:1643]	protein_coding	5,717505944	-1,382665771	0,016232017	1
ENSG00000239388	ASB14	ankyrin repeat and SOCS box containing 14 [Source:HGNC Symbol;Acc:HGNC:19766]	protein_coding	16,51504591	1,064103542	0,016235518	1
ENSG00000135477	KRT87P	keratin 87 pseudogene [Source:HGNC Symbol;Acc:HGNC:30198]	transcribed_unprocessed_pseudogene	2,105056936	-1,385406366	0,01623672	1
ENSG00000227195	MIR663AHG	MIR663A host gene [Source:HGNC Symbol;Acc:HGNC:27662]	processed_transcript	7,831405301	-1,177705964	0,016261813	1
ENSG00000283839			antisense_RNA	20,33937232	-0,809670468	0,016265726	1
ENSG00000219451	RPL23P8	ribosomal protein L23 pseudogene 8 [Source:HGNC Symbol;Acc:HGNC:36225]	processed_pseudogene	15,41044888	-1,124777773	0,016292545	1
ENSG00000142794	NBPF3	NBPF member 3 [Source:HGNC Symbol;Acc:HGNC:25076]	protein_coding	273,4972561726	0,545005251	0,016395194	1
ENSG00000163331	DAPL1	death associated protein like 1 [Source:HGNC Symbol;Acc:HGNC:21490]	protein_coding	21,78989952	-1,345410932	0,01642668	1
ENSG00000273275			antisense_RNA	14,59813406	1,120786922	0,016450618	1
ENSG00000249341			sense_intronic	2,777818088	-1,380308506	0,016625285	1
ENSG00000270938	RAP2CP1	RAP2C pseudogene 1 [Source:HGNC Symbol;Acc:HGNC:49146]	transcribed_processed_pseudogene	3,773444593	-1,355844289	0,016646651	1
ENSG00000276352	MIR668	microRNA 668 [Source:HGNC Symbol;Acc:HGNC:33135]	miRNA	4,549759351	1,327899074	0,016654335	1
ENSG00000197454	OR2L5	olfactory receptor family 2 subfamily L member 5 [Source:HGNC Symbol;Acc:HGNC:15011]	protein_coding	3,115542277	-1,376975508	0,016737375	1
ENSG00000036565	SLC18A1	solute carrier family 18 member A1 [Source:HGNC Symbol;Acc:HGNC:10934]	protein_coding	30,90900877	-1,270009554	0,016790318	1
ENSG00000236991	EDRF1-AS1	EDRF1 antisense RNA 1 [Source:HGNC Symbol;Acc:HGNC:49501]	antisense_RNA	6,617254311	1,218644782	0,016806766	1
ENSG00000104611	SH2D4A	SH2 domain containing 4A [Source:HGNC Symbol;Acc:HGNC:26102]	protein_coding	11,00345495	-1,150370597	0,01687825	1
ENSG00000174953	DHX36	DEAH-box helicase 36 [Source:HGNC Symbol;Acc:HGNC:14410]	protein_coding	1261,226354	0,237731615	0,016956085	1
ENSG00000130244	FAM98C	family with sequence similarity 98 member C [Source:HGNC Symbol;Acc:HGNC:27119]	protein_coding	329,8013492	0,328934407	0,016958451	1
ENSG00000243711	RPL21P116		processed_pseudogene	3,262825096	-1,296840771	0,017008636	1
ENSG00000163995	ABLIM2	actin binding LIM protein family member 2 [Source:HGNC Symbol;Acc:HGNC:19195]	protein_coding	176,0133191	0,584440083	0,017024699	1
ENSG00000178591	DEFB125	defensin beta 125 [Source:HGNC Symbol;Acc:HGNC:18105]	protein_coding	2,692872927	-1,365880014	0,017057701	1
ENSG00000212769	HMGN2P8	high mobility group nucleosomal binding domain 2 pseudogene 8 [Source:HGNC Symbol;Acc:HGNC:31662]	processed_pseudogene	4,20008506	-1,150522132	0,017118084	1
ENSG00000273559	CWC25	CWC25 spliceosome associated protein homolog [Source:HGNC Symbol;Acc:HGNC:25989]	protein_coding	416,7344381	-0,2753136	0,017118868	1
ENSG00000133937	GSC	goosecoid homeobox [Source:HGNC Symbol;Acc:HGNC:4612]	protein_coding	6,74012118	-1,303082578	0,017153322	1
ENSG00000256250			lincRNA	1,599160834	1,365638269	0,017164634	1
ENSG00000237668	RPS15AP38	ribosomal protein S15a pseudogene 38 [Source:HGNC Symbol;Acc:HGNC:36523]	processed_pseudogene	2,457282144	-1,283233236	0,017237415	1
ENSG00000272787			lincRNA	1,751741648	-1,364537689	0,017239554	1
ENSG00000164241	C5orf63	chromosome 5 open reading frame 63 [Source:HGNC Symbol;Acc:HGNC:40051]	protein_coding	12,7283278	-1,325749285	0,01732381	1
ENSG00000232192	KIF26B-AS1	KIF26B antisense RNA 1 [Source:HGNC Symbol;Acc:HGNC:41098]	antisense_RNA	2,072182074	-1,370881068	0,017386966	1
ENSG00000205899	BHLHA9	basic helix-loop-helix family member a9 [Source:HGNC Symbol;Acc:HGNC:35126]	protein_coding	1,889867045	-1,271904902	0,017397968	1
ENSG00000130700	GATA5	GATA binding protein 5 [Source:HGNC Symbol;Acc:HGNC:15802]	protein_coding	4,958134349	-1,293328814	0,01740771	1
ENSG00000104883	PEX11G	peroxisomal biogenesis factor 11 gamma [Source:HGNC Symbol;Acc:HGNC:20208]	protein_coding	38,02274235	-0,630476919	0,017444709	1
ENSG00000198576	ARC	activity regulated cytoskeleton associated protein [Source:HGNC Symbol;Acc:HGNC:648]	protein_coding	100,5338579	1,264283617	0,017469803	1
ENSG00000277935			lincRNA	2,988996254	1,365594601	0,017492307	1
ENSG00000092036	HAUS4	HAUS augmin like complex subunit 4 [Source:HGNC Symbol;Acc:HGNC:20163]	protein_coding	115,8750968	-0,724774604	0,017513692	1
ENSG00000278131			unprocessed_pseudogene	2,300627814	-1,364317767	0,01758637	1
ENSG00000118496	FBXO30	F-box protein 30 [Source:HGNC Symbol;Acc:HGNC:15600]	protein_coding	1949,567609	0,3719838	0,017600407	1
ENSG00000256642	LINC00273	long intergenic non-protein coding RNA 273 [Source:HGNC Symbol;Acc:HGNC:38595]	lincRNA	1,668613236	-1,30251538	0,017682601	1
ENSG00000114126	TFDP2	transcription factor Dp-2 [Source:HGNC Symbol;Acc:HGNC:11751]	protein_coding	2304,290551	-0,363861598	0,017695399	1
ENSG00000283269			transcribed_unprocessed_pseudogene	2,48679983	-1,345626052	0,017736408	1
ENSG00000095739	BAMBI	BMP and activin membrane bound inhibitor [Source:HGNC Symbol;Acc:HGNC:30251]	protein_coding	342,9566494	-1,184416264	0,017750618	1
ENSG00000185306	C12orf56	chromosome 12 open reading frame 56 [Source:HGNC Symbol;Acc:HGNC:26967]	protein_coding	7,524969921	-1,349913095	0,017761456	1
ENSG00000270872	SRGAP2D	SLIT-ROBO Rho GTPase activating protein 2D (pseudogene) [Source:HGNC Symbol;Acc:HGNC:43932]	unprocessed_pseudogene	4,855136162	1,253878525	0,017804092	1
ENSG00000223396	RPS10P7	ribosomal protein S10 pseudogene 7 [Source:HGNC Symbol;Acc:HGNC:36423]	transcribed_processed_pseudogene	7,58680451	-1,030292257	0,01780475	1
ENSG00000120659	TNFSF11	TNF superfamily member 11 [Source:HGNC Symbol;Acc:HGNC:11926]	protein_coding	2,282922252	-1,364539259	0,017919092	1
ENSG00000269292			antisense_RNA	90,73516817	1,318141013	0,017936348	1
ENSG00000108515	ENO3	enolase 3 [Source:HGNC Symbol;Acc:HGNC:3354]	protein_coding	106,2754528	-0,524252102	0,017944036	1
ENSG00000160951	PTGER1	prostaglandin E receptor 1 [Source:HGNC Symbol;Acc:HGNC:9593]	protein_coding	7,135868691	-1,262974409	0,017945273	1
ENSG00000124731	TREM1	triggering receptor expressed on myeloid cells 1 [Source:HGNC Symbol;Acc:HGNC:17760]	protein_coding	5,601853153	-1,272855805	0,017949913	1
ENSG00000196747	HIST1H2AI	histone cluster 1 H2A family member i [Source:HGNC Symbol;Acc:HGNC:4725]	protein_coding	845,4673861	-1,063644746	0,017976251	1
ENSG00000258504			lincRNA	14,188974	1,193272895	0,017980267	1
ENSG00000243398	RN7SL141P	RNA, 7SL, cytoplasmic 141, pseudogene [Source:HGNC Symbol;Acc:HGNC:46157]	misc_RNA	6,728121437	0,980759545	0,018006187	1

ENSG00000283414		U6 spliceosomal RNA [Source:RFAM;Acc:RF00026]	snRNA	1,16525932	-1,349096175	0,018028269	1
ENSG00000060656	PTPRU	protein tyrosine phosphatase, receptor type U [Source:HGNC Symbol;Acc:HGNC:9683]	protein_coding	393,3414236	-1,16504218	0,018058077	1
ENSG00000230190			sense_intronic	3,747599875	1,327387005	0,018083686	1
ENSG00000139880	CDH24	cadherin 24 [Source:HGNC Symbol;Acc:HGNC:14265]	protein_coding	743,2629746	-0,523872771	0,0181065	1
ENSG00000138079	SLC3A1	solute carrier family 3 member 1 [Source:HGNC Symbol;Acc:HGNC:11025]	protein_coding	28,34179862	0,989997508	0,018136995	1
ENSG00000120837	NFYB	nuclear transcription factor Y subunit beta [Source:HGNC Symbol;Acc:HGNC:7805]	protein_coding	1269,034855	-0,400107255	0,018150768	1
ENSG00000261257			lincRNA	1,598073938	-1,360124602	0,018173271	1
ENSG00000156795	WDYHV1	WDYHV motif containing 1 [Source:HGNC Symbol;Acc:HGNC:25490]	protein_coding	197,6337055	-0,403890629	0,018234907	1
ENSG00000258010			antisense_RNA	3,183419103	-1,287069011	0,018256432	1
ENSG00000149599	DUSP15	dual specificity phosphatase 15 [Source:HGNC Symbol;Acc:HGNC:16236]	protein_coding	126,3596414	0,482003865	0,018379535	1
ENSG00000204632	HLA-G	major histocompatibility complex, class I, G [Source:HGNC Symbol;Acc:HGNC:4964]	protein_coding	19,05363474	-1,237772725	0,018411588	1
ENSG00000100884	CPNE6	copine 6 [Source:HGNC Symbol;Acc:HGNC:2319]	protein_coding	241,189305	-1,353014285	0,018437494	1
ENSG00000184292	TACSTD2	tumor associated calcium signal transducer 2 [Source:HGNC Symbol;Acc:HGNC:11530]	protein_coding	4,664760351	-1,220974624	0,018493548	1
ENSG00000259446			antisense_RNA	3,511171296	-1,235718198	0,018525727	1
ENSG00000268812			antisense_RNA	5,122827797	-1,280752386	0,018528983	1
ENSG00000172987	HPSE2	heparanase 2 (inactive) [Source:HGNC Symbol;Acc:HGNC:18374]	protein_coding	12,64927282	-1,330733461	0,018617898	1
ENSG00000267615			antisense_RNA	4,81723388	1,353280412	0,018683455	1
ENSG00000177752	YIPF7	Yip1 domain family member 7 [Source:HGNC Symbol;Acc:HGNC:26825]	protein_coding	14,1820413	0,825040854	0,018690444	1
ENSG00000170486	KRT72	keratin 72 [Source:HGNC Symbol;Acc:HGNC:28932]	protein_coding	2,413725623	-1,352681226	0,018705821	1
ENSG00000140961	OSGIN1	oxidative stress induced growth inhibitor 1 [Source:HGNC Symbol;Acc:HGNC:30093]	protein_coding	44,55252823	1,19723066	0,018801004	1
ENSG00000204390	HSPA1L	heat shock protein family A (Hsp70) member 1 like [Source:HGNC Symbol;Acc:HGNC:5234]	protein_coding	46,6328239	0,848686145	0,018812747	1
ENSG00000243333	RN7SL174P	RNA, 7SL, cytoplasmic 174, pseudogene [Source:HGNC Symbol;Acc:HGNC:46190]	misc_RNA	2,70070885	1,262243215	0,018845243	1
ENSG00000230319			antisense_RNA	4,645128963	1,268422754	0,018870197	1
ENSG00000160007	ARHGAP35	Rho GTPase activating protein 35 [Source:HGNC Symbol;Acc:HGNC:4591]	protein_coding	4758,676669	0,303605327	0,018891133	1
ENSG00000180440	SERTM1	serine rich and transmembrane domain containing 1 [Source:HGNC Symbol;Acc:HGNC:33792]	protein_coding	76,6189717	-1,224309242	0,018957999	1
ENSG00000183971	NPW	neuropeptide W [Source:HGNC Symbol;Acc:HGNC:30509]	protein_coding	8,650323358	-1,053463532	0,019049106	1
ENSG00000267459			transcribed_processed_pseudogene	13,18101606	1,001942434	0,019073099	1
ENSG00000283189			protein_coding	7,498364834	0,994594708	0,019079593	1
ENSG00000184471	C1QTNF8	C1q and TNF related 8 [Source:HGNC Symbol;Acc:HGNC:31374]	protein_coding	4,634850241	-1,179807677	0,019110967	1
ENSG00000120937	NPPB	natriuretic peptide B [Source:HGNC Symbol;Acc:HGNC:7940]	protein_coding	0,990013178	-1,321132164	0,019125358	1
ENSG00000236498			antisense_RNA	13,77778834	1,302706925	0,019162778	1
ENSG00000198689	SLC9A6	solute carrier family 9 member A6 [Source:HGNC Symbol;Acc:HGNC:11079]	protein_coding	1953,790774	0,36805154	0,019220083	1
ENSG00000174885	NLRP6	NLR family pyrin domain containing 6 [Source:HGNC Symbol;Acc:HGNC:22944]	protein_coding	4,998724226	-1,344135549	0,019252549	1
ENSG00000235779			lincRNA	3,293108721	-1,349241778	0,019257259	1
ENSG00000224668	IPO8P1	importin 8 pseudogene 1 [Source:HGNC Symbol;Acc:HGNC:41955]	processed_pseudogene	2,106967094	-1,29458603	0,019312443	1
ENSG00000154227	CERS3	ceramide synthase 3 [Source:HGNC Symbol;Acc:HGNC:23752]	protein_coding	4,19656735	-1,262644163	0,01937606	1
ENSG00000012779	ALOX5	arachidonate 5-lipoxygenase [Source:HGNC Symbol;Acc:HGNC:435]	protein_coding	5,171453424	-1,291647505	0,019473004	1
ENSG00000235608	NKX1-1	NK1 homeobox 1 [Source:HGNC Symbol;Acc:HGNC:24975]	protein_coding	8,464216605	-1,344665828	0,019528231	1
ENSG00000258525			antisense_RNA	28,33437009	0,779292112	0,019537223	1
ENSG00000207426		Y RNA [Source:RFAM;Acc:RF00019]	misc_RNA	12,39853643	1,126824696	0,019538686	1
ENSG00000204922	UQCC3	ubiquinol-cytochrome c reductase complex assembly factor 3 [Source:HGNC Symbol;Acc:HGNC:34399]	protein_coding	292,0973182	-0,424608174	0,019557315	1
ENSG00000283314	LINC02357	long intergenic non-protein coding RNA 2357 [Source:HGNC Symbol;Acc:HGNC:53279]	lincRNA	1,386229678	-1,331743955	0,019634956	1
ENSG00000279528			TEC	60,99445518	0,486252201	0,019739078	1
ENSG00000282890			lincRNA	4,226369105	-1,26427131	0,019796589	1
ENSG00000231827			transcribed_unprocessed_pseudogene	3,944225644	-1,334423609	0,019856173	1
ENSG00000173530	TNFRSF10D	TNF receptor superfamily member 10d [Source:HGNC Symbol;Acc:HGNC:11907]	protein_coding	71,36267451	0,76945913	0,019990302	1
ENSG00000258429	PDF	peptide deformylase, mitochondrial [Source:HGNC Symbol;Acc:HGNC:30012]	protein_coding	30,8552947	-0,694038762	0,020036799	1
ENSG00000027644	INSRR	insulin receptor related receptor [Source:HGNC Symbol;Acc:HGNC:6093]	protein_coding	16,21056418	-1,107946206	0,020096492	1
ENSG00000215241	LINC02449	long intergenic non-protein coding RNA 2449 [Source:HGNC Symbol;Acc:HGNC:53381]	lincRNA	30,00266033	-0,797672622	0,020142038	1
ENSG00000112992	NNT	nicotinamide nucleotide transhydrogenase [Source:HGNC Symbol;Acc:HGNC:7863]	protein_coding	2583,901174	0,190034761	0,020276606	1
ENSG00000135127	BICDL1	BICD family like cargo adaptor 1 [Source:HGNC Symbol;Acc:HGNC:28095]	protein_coding	628,1645942	0,607942796	0,020326973	1
ENSG00000162105	SHANK2	SH3 and multiple ankyrin repeat domains 2 [Source:HGNC Symbol;Acc:HGNC:14295]	protein_coding	430,5367294	0,62301045	0,020337388	1
ENSG00000257658			processed_pseudogene	1,781768131	-1,334377121	0,020368828	1
ENSG00000160957	RECQL4	RecQ like helicase 4 [Source:HGNC Symbol;Acc:HGNC:9949]	protein_coding	444,1383911	-0,637122478	0,020376492	1
ENSG00000276071			lincRNA	29,33667215	0,568552975	0,020380265	1
ENSG00000100478	AP4S1	adaptor related protein complex 4 sigma 1 subunit [Source:HGNC Symbol;Acc:HGNC:575]	protein_coding	500,5946359	0,327468189	0,020385812	1
ENSG00000161609	CCDC155	coiled-coil domain containing 155 [Source:HGNC Symbol;Acc:HGNC:26520]	protein_coding	6,933623343	-1,329300201	0,020490823	1
ENSG00000125813	PAX1	paired box 1 [Source:HGNC Symbol;Acc:HGNC:8615]	protein_coding	8,562003314	-1,268696115	0,020545422	1
ENSG00000178919	FOXE1	forkhead box E1 [Source:HGNC Symbol;Acc:HGNC:3806]	protein_coding	7,010073559	-1,287217921	0,020560001	1
ENSG00000269113	TRABD2B	TraB domain containing 2B [Source:HGNC Symbol;Acc:HGNC:44200]	protein_coding	12,48933624	-1,281030067	0,020625602	1
ENSG00000272311	IFNL4P1	interferon lambda 4 pseudogene 1 [Source:HGNC Symbol;Acc:HGNC:45090]	unprocessed_pseudogene	1,056610389	-1,156041791	0,020658799	1
ENSG00000134463	ECHDC3	enoyl-CoA hydratase domain containing 3 [Source:HGNC Symbol;Acc:HGNC:23489]	protein_coding	47,22750178	-1,090457863	0,020757784	1
ENSG00000226605			antisense_RNA	3,317028563	1,291366431	0,020789951	1
ENSG00000262119			sense_intronic	114,059779	1,132209405	0,020802573	1
ENSG00000237341	SYP-AS1	SYP antisense RNA 1 [Source:HGNC Symbol;Acc:HGNC:40571]	antisense_RNA	4,555625649	1,323465986	0,020808308	1
ENSG00000226245	ZNF32-AS1	ZNF32 antisense RNA 1 [Source:HGNC Symbol;Acc:HGNC:23577]	antisense_RNA	6,521069352	1,315063293	0,020901998	1
ENSG00000272334			lincRNA	1,027228393	1,316298252	0,020914467	1

ENSG00000155966	AFF2	AF4/FMR2 family member 2 [Source:HGNC Symbol;Acc:HGNC:3776]	protein_coding	1597,044188	0,799278008	0,020936145	1
ENSG00000260704	LINC00543	long intergenic non-protein coding RNA 543 [Source:HGNC Symbol;Acc:HGNC:43678]	lincRNA	4,22170125	-1,307432968	0,020959313	1
ENSG00000225912			processed_pseudogene	2,154237611	-1,251976705	0,021096654	1
ENSG00000170088	TMEM192	transmembrane protein 192 [Source:HGNC Symbol;Acc:HGNC:26775]	protein_coding	625,0351034	0,300419506	0,021110029	1
ENSG00000259504			lincRNA	3,320942854	-1,285187291	0,021114873	1
ENSG00000105609	LILRB5	leukocyte immunoglobulin like receptor B5 [Source:HGNC Symbol;Acc:HGNC:6609]	protein_coding	4,899428142	-1,326962551	0,021135278	1
ENSG00000086205	FOLH1	folate hydrolase 1 [Source:HGNC Symbol;Acc:HGNC:3788]	protein_coding	28,63944391	-1,322813368	0,021188872	1
ENSG00000071539	TRIP13	thyroid hormone receptor interactor 13 [Source:HGNC Symbol;Acc:HGNC:12307]	protein_coding	356,8925201	-0,648312027	0,021271863	1
ENSG00000181408	UTS2R	urotensin 2 receptor [Source:HGNC Symbol;Acc:HGNC:4468]	protein_coding	3,109279862	-1,321704088	0,021278385	1
ENSG00000156113	KCNMA1	potassium calcium-activated channel subfamily M alpha 1 [Source:HGNC Symbol;Acc:HGNC:6284]	protein_coding	405,7595605	0,686201921	0,021369967	1
ENSG00000241625	RN7SL18P	RNA, 7SL, cytoplasmic 18, pseudogene [Source:HGNC Symbol;Acc:HGNC:46034]	misc_RNA	63,25036807	0,882539335	0,021380712	1
ENSG00000133019	CHRM3	cholinergic receptor muscarinic 3 [Source:HGNC Symbol;Acc:HGNC:1952]	protein_coding	171,1044099	0,977938536	0,021382346	1
ENSG00000151834	GABRA2	gamma-aminobutyric acid type A receptor alpha2 subunit [Source:HGNC Symbol;Acc:HGNC:4076]	protein_coding	439,8133634	0,977760469	0,021551956	1
ENSG00000276462			lincRNA	2,836585954	-1,318506144	0,021567505	1
ENSG00000181036	FCRL6	Fc receptor like 6 [Source:HGNC Symbol;Acc:HGNC:31910]	protein_coding	3,502252429	-1,324068647	0,021601598	1
ENSG00000196735	HLA-DQA1	major histocompatibility complex, class II, DQ alpha 1 [Source:HGNC Symbol;Acc:HGNC:4942]	protein_coding	13,27072104	-1,322424823	0,021677895	1
ENSG00000272892			lincRNA	27,79685235	-0,723923888	0,021686065	1
ENSG00000244945			antisense_RNA	10,17783595	1,00794752	0,021775639	1
ENSG00000261222			lincRNA	1,825104636	-1,321745761	0,021814287	1
ENSG00000274478			lincRNA	2,474168654	-1,319404262	0,021832716	1
ENSG00000218891	ZNF579	zinc finger protein 579 [Source:HGNC Symbol;Acc:HGNC:26646]	protein_coding	258,8882755	-0,431961039	0,021834605	1
ENSG00000204540	PSORS1C1	psoriasis susceptibility 1 candidate 1 [Source:HGNC Symbol;Acc:HGNC:17202]	protein_coding	11,96113319	-1,242857534	0,021876121	1
ENSG00000234292			lincRNA	2,632160127	-1,255510588	0,021882534	1
ENSG00000177468	OLIG3	oligodendrocyte transcription factor 3 [Source:HGNC Symbol;Acc:HGNC:18003]	protein_coding	28,18749136	-1,318808304	0,021941856	1
ENSG00000164107	HAND2	heart and neural crest derivatives expressed 2 [Source:HGNC Symbol;Acc:HGNC:4808]	protein_coding	8,608259302	-1,284746294	0,021955232	1
ENSG00000107742	SPOCK2	SPARC/osteonectin, cwcv and kazal like domains proteoglycan 2 [Source:HGNC Symbol;Acc:HGNC:13564]	protein_coding	5628,034861	1,086008375	0,02195603	1
ENSG00000110799	VWF	von Willebrand factor [Source:HGNC Symbol;Acc:HGNC:12726]	protein_coding	13,00437304	-1,162327951	0,02196842	1
ENSG00000230555			lincRNA	1,545355445	-1,319998341	0,021992067	1
ENSG00000250337	PURPL	p53 upregulated regulator of p53 levels [Source:HGNC Symbol;Acc:HGNC:48995]	lincRNA	89,99024573	-1,081504189	0,022010585	1
ENSG00000064961	HMG20B	high mobility group 20B [Source:HGNC Symbol;Acc:HGNC:5002]	protein_coding	1056,823284	-0,450983385	0,022015654	1
ENSG00000234518	PTGES3P1	prostaglandin E synthase 3 pseudogene 1 [Source:HGNC Symbol;Acc:HGNC:43824]	processed_pseudogene	5,099054222	-1,026803617	0,022049442	1
ENSG00000188778	ADRB3	adrenoceptor beta 3 [Source:HGNC Symbol;Acc:HGNC:288]	protein_coding	4,286852068	-1,31138564	0,022069333	1
ENSG00000224356			sense_intronic	5,333554985	-1,100547742	0,022098637	1
ENSG00000187812			transcribed_unprocessed_pseudogene	7,086177985	-1,318427176	0,022112896	1
ENSG00000240086			processed_transcript	12,27215165	-1,088837463	0,022157564	1
ENSG00000259895			antisense_RNA	15,45125905	-0,87601103	0,022164676	1
ENSG00000230521	HCG4P7		unprocessed_pseudogene	7,127224008	1,205972308	0,022281086	1
ENSG00000269994			lincRNA	101,2689222	1,227944463	0,022304949	1
ENSG00000180767	CHST13	carbohydrate sulfotransferase 13 [Source:HGNC Symbol;Acc:HGNC:21755]	protein_coding	4,472362797	-1,308152359	0,022317053	1
ENSG00000255545			processed_transcript	69,0942853	0,911518906	0,022357632	1
ENSG00000240393			processed_pseudogene	29,7384994	1,146592606	0,022369492	1
ENSG00000185269	NOTUM	notum, palmitoleoyl-protein carboxylesterase [Source:HGNC Symbol;Acc:HGNC:27106]	protein_coding	38,28628871	-1,087931373	0,022405944	1
ENSG00000273295			lincRNA	20,04534535	1,246914924	0,022430934	1
ENSG00000101190	TCFL5	transcription factor like 5 [Source:HGNC Symbol;Acc:HGNC:11646]	protein_coding	479,74425	-0,387790468	0,022444429	1
ENSG00000166265	CYYR1	cysteine and tyrosine rich 1 [Source:HGNC Symbol;Acc:HGNC:16274]	protein_coding	114,5722855	-1,051271297	0,022460875	1
ENSG00000159374	M1AP	meiosis 1 associated protein [Source:HGNC Symbol;Acc:HGNC:25183]	protein_coding	3,615210974	-1,295676485	0,022473259	1
ENSG00000240990	HOXA11-AS	HOXA11 antisense RNA [Source:HGNC Symbol;Acc:HGNC:24957]	antisense_RNA	6,048615978	-1,315398203	0,022498548	1
ENSG00000054793	ATP9A	ATPase phospholipid transporting 9A (putative) [Source:HGNC Symbol;Acc:HGNC:13540]	protein_coding	8475,700997	0,475654092	0,022524801	1
ENSG00000174950	CD164L2	CD164 molecule like 2 [Source:HGNC Symbol;Acc:HGNC:32043]	protein_coding	2,983334475	-1,279804641	0,022527293	1
ENSG00000272330			sense_intronic	6,423034334	1,260060497	0,022552453	1
ENSG00000135749	PCNX2	pecanex homolog 2 [Source:HGNC Symbol;Acc:HGNC:8736]	protein_coding	1148,163893	0,486923977	0,022560839	1
ENSG00000198754	OXCT2	3-oxoacid CoA-transferase 2 [Source:HGNC Symbol;Acc:HGNC:18606]	protein_coding	1,940312427	-1,31069106	0,022632853	1
ENSG00000242908	AADACL2-AS1	AADACL2 antisense RNA 1 [Source:HGNC Symbol;Acc:HGNC:50301]	antisense_RNA	1,260799356	-1,144553175	0,022675818	1
ENSG00000183598	HIST2H3D	histone cluster 2 H3 family member d [Source:HGNC Symbol;Acc:HGNC:25311]	protein_coding	216,9912648	-1,012083492	0,02271681	1
ENSG00000259554			sense_intronic	11,2632166	1,036284969	0,022740817	1
ENSG00000268996	MAN1B1-AS1	MAN1B1 antisense RNA 1 (head to head) [Source:HGNC Symbol;Acc:HGNC:48715]	antisense_RNA	34,69340207	-0,613089057	0,022756087	1
ENSG00000197532	OR6Y1	olfactory receptor family 6 subfamily Y member 1 [Source:HGNC Symbol;Acc:HGNC:14823]	protein_coding	6,682510503	-1,31044562	0,022791054	1
ENSG00000197641	SERPINB13	serpin family B member 13 [Source:HGNC Symbol;Acc:HGNC:8944]	protein_coding	4,930451808	-1,291573835	0,022846918	1
ENSG00000254369	HOXA-AS3	HOXA cluster antisense RNA 3 [Source:HGNC Symbol;Acc:HGNC:43748]	antisense_RNA	6,612537378	-1,28308534	0,022854112	1
ENSG00000184363	PKP3	plakophilin 3 [Source:HGNC Symbol;Acc:HGNC:9025]	protein_coding	4,514425953	-1,308285174	0,022862013	1
ENSG00000132026	RTBDN	retbindin [Source:HGNC Symbol;Acc:HGNC:30310]	protein_coding	6,299788835	-1,169636285	0,022879634	1
ENSG00000242512	LINC01206	long intergenic non-protein coding RNA 1206 [Source:HGNC Symbol;Acc:HGNC:49637]	lincRNA	23,26324562	-1,143105684	0,022936161	1
ENSG00000164161	HHIP	hedgehog interacting protein [Source:HGNC Symbol;Acc:HGNC:14866]	protein_coding	31,38381913	-1,174029211	0,022939577	1
ENSG00000260781	ARHGAP23P1	Rho GTPase activating protein 23 pseudogene 1 [Source:HGNC Symbol;Acc:HGNC:45039]	transcribed_unprocessed_pseudogene	1,613871827	-1,306587948	0,022959588	1
ENSG00000253308			lincRNA	2,029579849	-1,306800832	0,023062841	1
ENSG00000267233	HNRNPA3P16	heterogeneous nuclear ribonucleoprotein A3 pseudogene 16 [Source:HGNC Symbol;Acc:HGNC:48762]	processed_pseudogene	3,095722901	-1,293238233	0,023089726	1
ENSG00000279047			antisense_RNA	25,53658993	1,207578127	0,023101889	1

ENSG00000162551	ALPL	alkaline phosphatase, liver/bone/kidney [Source:HGNC Symbol;Acc:HGNC:438]	protein_coding	217,8652149	-1,064453568	0,023152084	1
ENSG00000272418			sense_intronic	2,703505186	1,259750564	0,023227907	1
ENSG00000175857	GAPT	GRB2 binding adaptor protein, transmembrane [Source:HGNC Symbol;Acc:HGNC:26588]	protein_coding	2,696752529	-1,306128016	0,023258887	1
ENSG00000100285	NEFH	neurofilament heavy [Source:HGNC Symbol;Acc:HGNC:7737]	protein_coding	30,27942204	-0,846564071	0,023281139	1
ENSG00000187922	LCN10	lipocalin 10 [Source:HGNC Symbol;Acc:HGNC:20892]	protein_coding	2,44955822	-1,299253012	0,023308751	1
ENSG00000228830			antisense_RNA	13,67090537	1,196747477	0,02335189	1
ENSG00000140839	CLEC18B	C-type lectin domain family 18 member B [Source:HGNC Symbol;Acc:HGNC:33849]	protein_coding	30,8725283	-1,072864088	0,023371367	1
ENSG00000088356	PDRG1	p53 and DNA damage regulated 1 [Source:HGNC Symbol;Acc:HGNC:16119]	protein_coding	305,4067111	0,439348364	0,023405648	1
ENSG00000177105	RHOG	ras homolog family member G [Source:HGNC Symbol;Acc:HGNC:672]	protein_coding	49,70347301	-0,576237534	0,023439253	1
ENSG00000173714	WFIKKN2	WAP, follistatin/kazal, immunoglobulin, kunitz and netrin domain containing 2 [Source:HGNC Symbol;Acc:HGNC:30916	protein_coding	91,37217915	1,300148642	0,023475573	1
ENSG00000260206			antisense_RNA	6,762071145	1,228511445	0,023501663	1
ENSG00000140932	CMTM2	CKLF like MARVEL transmembrane domain containing 2 [Source:HGNC Symbol;Acc:HGNC:19173]	protein_coding	7,474858373	-1,266819084	0,023505708	1
ENSG00000267472			processed_pseudogene	2,759150729	-1,258837978	0,023530419	1
ENSG00000198237			unprocessed_pseudogene	6,571898575	1,173550205	0,023541763	1
ENSG00000279259			TEC	58,32462671	-0,494847764	0,023599252	1
ENSG00000188000	OR7D2	olfactory receptor family 7 subfamily D member 2 [Source:HGNC Symbol;Acc:HGNC:8378]	protein_coding	5,327669596	-1,298627072	0,023662985	1
ENSG00000184389	A3GALT2	alpha 1,3-galactosyltransferase 2 [Source:HGNC Symbol;Acc:HGNC:30005]	protein_coding	7,91832103	-1,112776491	0,023685908	1
ENSG00000264443			lincRNA	9,615786649	1,22380269	0,023699853	1
ENSG00000205922	ONECUT3	one cut homeobox 3 [Source:HGNC Symbol;Acc:HGNC:13399]	protein_coding	16,8121356	-1,271061987	0,023817741	1
ENSG00000198963	RORB	RAR related orphan receptor B [Source:HGNC Symbol;Acc:HGNC:10259]	protein_coding	1018,255089	0,683315016	0,023829151	1
ENSG00000163815	CLEC3B	C-type lectin domain family 3 member B [Source:HGNC Symbol;Acc:HGNC:11891]	protein_coding	3,478357145	1,169857119	0,023896956	1
ENSG00000269951			sense_intronic	80,79541292	0,49253453	0,023913788	1
ENSG00000279924			unprocessed_pseudogene	1,540880918	-1,298097339	0,023957185	1
ENSG00000212153	RNU1-82P	RNA, U1 small nuclear 82, pseudogene [Source:HGNC Symbol;Acc:HGNC:48424]	snRNA	38,66482799	0,804385683	0,023977175	1
ENSG00000188488	SERPINA5	serpin family A member 5 [Source:HGNC Symbol;Acc:HGNC:8723]	protein_coding	25,54914556	-1,281451341	0,023993308	1
ENSG00000165617	DACT1	dishevelled binding antagonist of beta catenin 1 [Source:HGNC Symbol;Acc:HGNC:17748]	protein_coding	2348,049854	0,731393993	0,02402514	1
ENSG00000152061	RABGAP1L	RAB GTPase activating protein 1 like [Source:HGNC Symbol;Acc:HGNC:24663]	protein_coding	1431,282272	0,392343756	0,02403751	1
ENSG00000259049			antisense_RNA	21,59932152	0,732761519	0,024129543	1
ENSG00000210176	MT-TH	mitochondrially encoded tRNA histidine [Source:HGNC Symbol;Acc:HGNC:7487]	Mt_tRNA	24,47350646	1,236325193	0,024138848	1
ENSG00000141316	SPACA3	sperm acrosome associated 3 [Source:HGNC Symbol;Acc:HGNC:16260]	protein_coding	3,569851618	-1,267825774	0,024172454	1
ENSG00000277837			lincRNA	6,33398443	-1,288418015	0,024266513	1
ENSG00000181819	KCTD9P2	potassium channel tetramerization domain containing 9 pseudogene 2 [Source:HGNC Symbol;Acc:HGNC:29274]	processed_pseudogene	1,927323524	-1,285900642	0,024317842	1
ENSG00000167528	ZNF641	zinc finger protein 641 [Source:HGNC Symbol;Acc:HGNC:31834]	protein_coding	1007,111729	0,433902612	0,024462614	1
ENSG00000145864	GABRB2	gamma-aminobutyric acid type A receptor beta2 subunit [Source:HGNC Symbol;Acc:HGNC:4082]	protein_coding	577,3792974	0,815526476	0,024541149	1
ENSG00000136574	GATA4	GATA binding protein 4 [Source:HGNC Symbol;Acc:HGNC:4173]	protein_coding	6,166602907	-1,283073662	0,024551745	1
ENSG00000109062	SLC9A3R1	SLC9A3 regulator 1 [Source:HGNC Symbol;Acc:HGNC:11075]	protein_coding	458,1763631	-0,541636929	0,024552669	1
ENSG00000136573	BLK	BLK proto-oncogene, Src family tyrosine kinase [Source:HGNC Symbol;Acc:HGNC:1057]	protein_coding	5,043871017	-1,246150983	0,024573926	1
ENSG00000269220	LINC00528	long intergenic non-protein coding RNA 528 [Source:HGNC Symbol;Acc:HGNC:26875]	lincRNA	2,199177751	-1,258534378	0,024654184	1
ENSG00000165025	SYK	spleen associated tyrosine kinase [Source:HGNC Symbol;Acc:HGNC:11491]	protein_coding	5,535250921	-1,275776272	0,024679865	1
ENSG00000275377	MIR1299	microRNA 1299 [Source:HGNC Symbol;Acc:HGNC:35290]	miRNA	2,097390453	1,275624972	0,024706076	1
ENSG00000275880			antisense_RNA	13,52810734	-1,024915617	0,024727325	1
ENSG00000227560	RPS15AP30	ribosomal protein S15a pseudogene 30 [Source:HGNC Symbol;Acc:HGNC:35495]	processed_pseudogene	1,702670378	-1,291058807	0,02473573	1
ENSG00000103241	FOXF1	forkhead box F1 [Source:HGNC Symbol;Acc:HGNC:3809]	protein_coding	20,69105491	-1,142364625	0,024761999	1
ENSG00000265784			antisense_RNA	16,55119181	1,258811249	0,024766036	1
ENSG00000175274	TP53I11	tumor protein p53 inducible protein 11 [Source:HGNC Symbol;Acc:HGNC:16842]	protein_coding	1090,008092	-0,775611312	0,024795548	1
ENSG00000283703	VSIG10L2	V-set and immunoglobulin domain containing 10 like 2 [Source:HGNC Symbol;Acc:HGNC:27879]	protein_coding	6,717234307	1,269921424	0,024835612	1
ENSG00000233273	AMMECR1LP1	AMMECR1 like pseudogene 1 [Source:HGNC Symbol;Acc:HGNC:39809]	processed_pseudogene	2,964286195	1,289479331	0,02486751	1
ENSG00000170381	SEMA3E	semaphorin 3E [Source:HGNC Symbol;Acc:HGNC:10727]	protein_coding	158,5387203	1,22853403	0,024883023	1
ENSG00000244578	LINC01391	long intergenic non-protein coding RNA 1391 [Source:HGNC Symbol;Acc:HGNC:50666]	lincRNA	3,257371734	-1,27862822	0,02488869	1
ENSG00000162062	TEDC2	tubulin epsilon and delta complex 2 [Source:HGNC Symbol;Acc:HGNC:25849]	protein_coding	231,9549816	-0,652061581	0,024919383	1
ENSG00000271824	SMIM32	small integral membrane protein 32 [Source:HGNC Symbol;Acc:HGNC:53640]	protein_coding	9,509117639	-1,1995137	0,024929208	1
ENSG00000066230	SLC9A3	solute carrier family 9 member A3 [Source:HGNC Symbol;Acc:HGNC:11073]	protein_coding	49,07275679	1,222052275	0,02495254	1
ENSG00000258072			processed_pseudogene	2,881660469	-1,282581517	0,02497704	1
ENSG00000188620	HMX3	H6 family homeobox 3 [Source:HGNC Symbol;Acc:HGNC:5019]	protein_coding	2,347567924	-1,278720852	0,025035458	1
ENSG00000008256	CYTH3	cytohesin 3 [Source:HGNC Symbol;Acc:HGNC:9504]	protein_coding	968,2172354	0,244822859	0,025041526	1
ENSG00000278746	RN7SL660P	RNA, 7SL, cytoplasmic 660, pseudogene [Source:HGNC Symbol;Acc:HGNC:46676]	misc_RNA	5,864235201	1,051385426	0,025043803	1
ENSG00000267191			antisense_RNA	16,64309839	0,805418308	0,025060133	1
ENSG00000267673	FDX2	ferredoxin 2 [Source:HGNC Symbol;Acc:HGNC:30546]	protein_coding	2,686425082	1,216992674	0,025075597	1
ENSG00000234199	LINC01191	long intergenic non-protein coding RNA 1191 [Source:HGNC Symbol;Acc:HGNC:49595]	lincRNA	1,363913738	-1,267070445	0,025139343	1
ENSG00000225357	RPF2P1	ribosome production factor 2 homolog pseudogene 1 [Source:HGNC Symbol;Acc:HGNC:16186]	processed_pseudogene	1,343320528	-1,28232746	0,025228036	1
ENSG00000101440	ASIP	agouti signaling protein [Source:HGNC Symbol;Acc:HGNC:745]	protein_coding	3,81613963	-1,273906526	0,025228323	1
ENSG00000104731	KLHDC4	kelch domain containing 4 [Source:HGNC Symbol;Acc:HGNC:25272]	protein_coding	401,0430126	0,292697777	0,02522991	1
ENSG00000128652	HOXD3	homeobox D3 [Source:HGNC Symbol;Acc:HGNC:5137]	protein_coding	2,471934995	-1,283024197	0,025249094	1
ENSG00000161132			unprocessed_pseudogene	3,266389428	-1,178809239	0,025260215	1
ENSG00000180385	EMC3-AS1	EMC3 antisense RNA 1 [Source:HGNC Symbol;Acc:HGNC:49223]	transcribed_unprocessed_pseudogene	104,709809	-0,525232487	0,025275129	1
ENSG00000187672	ERC2	ELKS/RAB6-interacting/CAST family member 2 [Source:HGNC Symbol;Acc:HGNC:31922]	protein_coding	910,9234019	0,708067412	0,02533696	1
ENSG00000131042	LILRB2	leukocyte immunoglobulin like receptor B2 [Source:HGNC Symbol;Acc:HGNC:6606]	protein_coding	2,933044246	-1,284837176	0,025349999	1

ENSG00000197249	SERPINA1	serpin family A member 1 [Source:HGNC Symbol;Acc:HGNC:8941]	protein_coding	3,177491879	-1,288451644	0,025352082	1
ENSG00000116176	TPSG1	tryptase gamma 1 [Source:HGNC Symbol;Acc:HGNC:14134]	protein_coding	3,091833368	1,280469878	0,025421525	1
ENSG00000102174	PHEX	phosphate regulating endopeptidase homolog X-linked [Source:HGNC Symbol;Acc:HGNC:8918]	protein_coding	56,01568556	0,812785287	0,025449954	1
ENSG00000090530	P3H2	prolyl 3-hydroxylase 2 [Source:HGNC Symbol;Acc:HGNC:19317]	protein_coding	88,07556063	-0,998440789	0,025461631	1
ENSG00000125533	BHLHE23	basic helix-loop-helix family member e23 [Source:HGNC Symbol;Acc:HGNC:16093]	protein_coding	11,15420323	-0,971698448	0,02546187	1
ENSG00000231955			antisense_RNA	12,99857171	1,084654369	0,025519061	1
ENSG00000166869	CHP2	calcineurin like EF-hand protein 2 [Source:HGNC Symbol;Acc:HGNC:24927]	protein_coding	3,877198411	-1,287204113	0,025561604	1
ENSG00000272138	LINC01607	long intergenic non-protein coding RNA 1607 [Source:HGNC Symbol;Acc:HGNC:51660]	lincRNA	4,262820938	-1,11273984	0,025609694	1
ENSG00000173207	CKS1B	CDC28 protein kinase regulatory subunit 1B [Source:HGNC Symbol;Acc:HGNC:19083]	protein_coding	244,2520188	-0,832030959	0,025624005	1
ENSG00000210144	MT-TY	mitochondrially encoded tRNA tyrosine [Source:HGNC Symbol;Acc:HGNC:7502]	Mt_tRNA	37,37866314	1,193840479	0,025628169	1
ENSG00000040933	INPP4A	inositol polyphosphate-4-phosphatase type I A [Source:HGNC Symbol;Acc:HGNC:6074]	protein_coding	2468,613281	0,32667845	0,025629138	1
ENSG00000253767	PCDHGA8	protocadherin gamma subfamily A, 8 [Source:HGNC Symbol;Acc:HGNC:8706]	protein_coding	65,30011186	0,933961245	0,025727639	1
ENSG00000198604	BAZ1A	bromodomain adjacent to zinc finger domain 1A [Source:HGNC Symbol;Acc:HGNC:960]	protein_coding	1377,849997	-0,528543679	0,025743801	1
ENSG00000134762	DSC3	desmocollin 3 [Source:HGNC Symbol;Acc:HGNC:3037]	protein_coding	25,92248868	-1,186405297	0,025760894	1
ENSG00000248079	DPH6-AS1	DPH6 antisense RNA 1 (head to head) [Source:HGNC Symbol;Acc:HGNC:44147]	lincRNA	9,522812312	-0,931339512	0,025828822	1
ENSG00000011465	DCN	decorin [Source:HGNC Symbol;Acc:HGNC:2705]	protein_coding	307,9297137	1,186587489	0,025880625	1
ENSG00000120498	TEX11	testis expressed 11 [Source:HGNC Symbol;Acc:HGNC:11733]	protein_coding	3,618650717	-1,203324395	0,025938819	1
ENSG00000225174	OSTM1-AS1	OSTM1 antisense RNA 1 [Source:HGNC Symbol;Acc:HGNC:43666]	lincRNA	9,182491307	-0,992895157	0,026001935	1
ENSG00000141252	VPS53	VPS53, GARP complex subunit [Source:HGNC Symbol;Acc:HGNC:25608]	protein_coding	1373,767202	0,324740588	0,026016369	1
ENSG00000206503	HLA-A	major histocompatibility complex, class I, A [Source:HGNC Symbol;Acc:HGNC:4931]	protein_coding	2813,498732	0,956083407	0,026022754	1
ENSG00000258430			antisense_RNA	29,95950974	1,109651316	0,026097306	1
ENSG00000206579	XKR4	XK related 4 [Source:HGNC Symbol;Acc:HGNC:29394]	protein_coding	1112,418287	0,728868564	0,026116791	1
ENSG00000145975	FAM217A	family with sequence similarity 217 member A [Source:HGNC Symbol;Acc:HGNC:21362]	protein_coding	3,608396307	-1,271734997	0,026139188	1
ENSG00000264449			antisense_RNA	10,53393468	0,924132353	0,026197739	1
ENSG00000172840	PDP2	pyruvate dehyrogenase phosphatase catalytic subunit 2 [Source:HGNC Symbol;Acc:HGNC:30263]	protein_coding	284,0023791	0,363769777	0,026207567	1
ENSG00000248645			unprocessed_pseudogene	1,285306595	-1,280492422	0,026240625	1
ENSG00000184227	ACOT1	acyl-CoA thioesterase 1 [Source:HGNC Symbol;Acc:HGNC:33128]	protein_coding	2,819704514	1,272979577	0,026255152	1
ENSG00000153558	FBXL2	F-box and leucine rich repeat protein 2 [Source:HGNC Symbol;Acc:HGNC:13598]	protein_coding	967,2349332	0,486175556	0,026272174	1
ENSG00000167895	TMC8	transmembrane channel like 8 [Source:HGNC Symbol;Acc:HGNC:20474]	protein_coding	9,530525909	-0,985608542	0,026310048	1
ENSG00000107859	PITX3	paired like homeodomain 3 [Source:HGNC Symbol;Acc:HGNC:9006]	protein_coding	3,216368345	-1,248259679	0,026382117	1
ENSG00000234664	HMGN2P5	high mobility group nucleosomal binding domain 2 pseudogene 5 [Source:HGNC Symbol;Acc:HGNC:33568]	processed_pseudogene	132,4240339	-0,653170028	0,026395139	1
ENSG00000171723	GPHN	gephyrin [Source:HGNC Symbol;Acc:HGNC:15465]	protein_coding	675,8530948	0,411850357	0,026441079	1
ENSG00000102755	FLT1	fms related tyrosine kinase 1 [Source:HGNC Symbol;Acc:HGNC:3763]	protein_coding	23,83210045	-1,221963914	0,02645657	1
ENSG00000197576	HOXA4	homeobox A4 [Source:HGNC Symbol;Acc:HGNC:5105]	protein_coding	2,695880974	-1,278687046	0,026470019	1
ENSG00000259589			antisense_RNA	20,27063115	1,111669454	0,026492553	1
ENSG00000267784			antisense_RNA	33,37734182	0,930941733	0,026530739	1
ENSG00000185231	MC2R	melanocortin 2 receptor [Source:HGNC Symbol;Acc:HGNC:6930]	protein_coding	1,641073215	-1,277477921	0,026535351	1
ENSG00000104903	LYL1	LYL1, basic helix-loop-helix family member [Source:HGNC Symbol;Acc:HGNC:6734]	protein_coding	6,223392766	-1,203911791	0,026557297	1
ENSG00000272057			lincRNA	1,706079893	1,262764165	0,026559686	1
ENSG00000257548			antisense_RNA	2,914261512	-1,275707894	0,026662827	1
ENSG00000260948			sense_overlapping	39,67340694	0,579536836	0,026675235	1
ENSG00000148600	CDHR1	cadherin related family member 1 [Source:HGNC Symbol;Acc:HGNC:14550]	protein_coding	130,7088386	1,021617664	0,026724423	1
ENSG00000184937	WT1	Wilms tumor 1 [Source:HGNC Symbol;Acc:HGNC:12796]	protein_coding	3,184977453	-1,266075629	0,026755974	1
ENSG00000273820	USP27X	ubiquitin specific peptidase 27, X-linked [Source:HGNC Symbol;Acc:HGNC:13486]	protein_coding	266,0899263	0,312348886	0,026777762	1
ENSG00000253785			processed_pseudogene	4,286362056	1,070181138	0,026819661	1
ENSG00000182774	RPS17	ribosomal protein S17 [Source:HGNC Symbol;Acc:HGNC:10397]	protein_coding	8345,3690388188	-0,510067126	0,026828458	1
ENSG00000206177	HBM	hemoglobin subunit mu [Source:HGNC Symbol;Acc:HGNC:4826]	protein_coding	1,321227153	-1,22217789	0,026851737	1
ENSG00000154258	ABCA9	ATP binding cassette subfamily A member 9 [Source:HGNC Symbol;Acc:HGNC:39]	protein_coding	11,63750292	-1,172971609	0,02686466	1
ENSG00000158715	SLC45A3	solute carrier family 45 member 3 [Source:HGNC Symbol;Acc:HGNC:8642]	protein_coding	137,5293682	-0,659534629	0,026879369	1
ENSG00000200823	SNORD114-2	small nucleolar RNA, C/D box 114-2 [Source:HGNC Symbol;Acc:HGNC:32990]	snoRNA	4,179815484	1,139558072	0,027028285	1
ENSG00000238015			processed_pseudogene	5,043729316	1,205832935	0,027204831	1
ENSG00000117602	RCAN3	RCAN family member 3 [Source:HGNC Symbol;Acc:HGNC:3042]	protein_coding	1064,7583814467	0,683587774	0,027242333	1
ENSG00000156427	FGF18	fibroblast growth factor 18 [Source:HGNC Symbol;Acc:HGNC:3674]	protein_coding	61,50266415	-1,204671246	0,027245781	1
ENSG00000257346			antisense_RNA	3,897304035	-1,209724737	0,027285979	1
ENSG00000201031		Y RNA [Source:RFAM;Acc:RF00019]	misc_RNA	35,80414791	0,851846024	0,027338301	1
ENSG00000186567	CEACAM19	carcinoembryonic antigen related cell adhesion molecule 19 [Source:HGNC Symbol;Acc:HGNC:31951]	protein_coding	60,25666109	0,731534407	0,027373612	1
ENSG00000175749	EIF3KP1	eukaryotic translation initiation factor 3 subunit K pseudogene 1 [Source:HGNC Symbol;Acc:HGNC:44016]	processed_pseudogene	7,869243802	0,991775998	0,027407008	1
ENSG00000115008	IL1A	interleukin 1 alpha [Source:HGNC Symbol;Acc:HGNC:5991]	protein_coding	5,993273718	-1,270596677	0,027501433	1
ENSG00000257781			lincRNA	2,954703449	-1,229342974	0,027562432	1
ENSG00000154262	ABCA6	ATP binding cassette subfamily A member 6 [Source:HGNC Symbol;Acc:HGNC:36]	protein_coding	23,16841774	-1,198595827	0,027592376	1
ENSG00000060762	MPC1	mitochondrial pyruvate carrier 1 [Source:HGNC Symbol;Acc:HGNC:21606]	protein_coding	836,1692188	0,468961248	0,027636086	1
ENSG00000283526	PRRT1B	proline rich transmembrane protein 1B [Source:HGNC Symbol;Acc:HGNC:53642]	protein_coding	1,264327524	-1,2153027	0,027749103	1
ENSG00000178226	PRSS36	protease, serine 36 [Source:HGNC Symbol;Acc:HGNC:26906]	protein_coding	15,0375997211	-0,883162394	0,027779214	1
ENSG00000268231			sense_intronic	8,652351711	0,863339396	0,027821852	1
ENSG00000174021	GNG5	G protein subunit gamma 5 [Source:HGNC Symbol;Acc:HGNC:4408]	protein_coding	362,7803819	-0,711065188	0,027829383	1
ENSG00000145220	LYAR	Ly1 antibody reactive [Source:HGNC Symbol;Acc:HGNC:26021]	protein_coding	268,1097002	-0,366820193	0,027852859	1
ENSG00000203416	FAM32BP	family with sequence similarity 32 member B, pseudogene [Source:HGNC Symbol;Acc:HGNC:30945]	processed_pseudogene	1,504266119	-1,26369988	0,027860709	1

ENSG00000150394	CDH8	cadherin 8 [Source:HGNC Symbol;Acc:HGNC:1767]	protein_coding	362,9632502	1,029589076	0,027964792	1
ENSG00000272004			antisense_RNA	7,479601607	-0,88137149	0,028064265	1
ENSG00000213221	DNLZ	DNL-type zinc finger [Source:HGNC Symbol;Acc:HGNC:33879]	protein_coding	33,75153937	0,753211034	0,028095566	1
ENSG00000236301	MRGPRG-AS1	MRGPRG antisense RNA 1 [Source:HGNC Symbol;Acc:HGNC:26691]	antisense_RNA	1,054189301	-1,1880944	0,028138483	1
ENSG00000129347	KRI1	KRI1 homolog [Source:HGNC Symbol;Acc:HGNC:25769]	protein_coding	514,7137016	-0,362508507	0,028153731	1
ENSG00000178184	PARD6G	par-6 family cell polarity regulator gamma [Source:HGNC Symbol;Acc:HGNC:16076]	protein_coding	503,8757384	-0,540250933	0,028180351	1
ENSG00000279771			TEC	4,283349331	-1,228719602	0,028198279	1
ENSG00000130675	MNX1	motor neuron and pancreas homeobox 1 [Source:HGNC Symbol;Acc:HGNC:4979]	protein_coding	9,092313612	-1,111310299	0,028206517	1
ENSG00000259404	EFTUD1P1	elongation factor Tu GTP binding domain containing 1 pseudogene 1 [Source:HGNC Symbol;Acc:HGNC:31739]	transcribed_unprocessed_pseudogene	26,90105761	0,592336988	0,028270616	1
ENSG00000257239			lincRNA	1,780607169	-1,246852324	0,028439225	1
ENSG00000204584			antisense_RNA	7,603080518	0,979538247	0,028545854	1
ENSG00000244300	GATA2-AS1	GATA2 antisense RNA 1 [Source:HGNC Symbol;Acc:HGNC:51108]	antisense_RNA	4,654271999	-1,231434442	0,028553612	1
ENSG00000178752	ERFE	erythroferrone [Source:HGNC Symbol;Acc:HGNC:26727]	protein_coding	32,84603584	-0,846599833	0,028576232	1
ENSG00000258583	LINC01500	long intergenic non-protein coding RNA 1500 [Source:HGNC Symbol;Acc:HGNC:51166]	lincRNA	1,531077916	1,257571006	0,028634651	1
ENSG00000261513			antisense_RNA	3,312349885	-1,163087608	0,028672014	1
ENSG00000230500	MKX-AS1	MKX antisense RNA 1 [Source:HGNC Symbol;Acc:HGNC:50686]	antisense_RNA	6,786215157	-1,246697057	0,028827991	1
ENSG00000198934	MAGEE1	MAGE family member E1 [Source:HGNC Symbol;Acc:HGNC:24934]	protein_coding	282,4306585	0,440453926	0,028831343	1
ENSG00000228363			antisense_RNA	30,08788927	0,966885797	0,028875239	1
ENSG00000187134	AKR1C1	aldo-keto reductase family 1 member C1 [Source:HGNC Symbol;Acc:HGNC:384]	protein_coding	331,4983921	0,717257459	0,02904189	1
ENSG00000129355	CDKN2D	cyclin dependent kinase inhibitor 2D [Source:HGNC Symbol;Acc:HGNC:1790]	protein_coding	566,9104305	0,555708163	0,029042454	1
ENSG00000130528	HRC	histidine rich calcium binding protein [Source:HGNC Symbol;Acc:HGNC:5178]	protein_coding	5,758780364	-1,175518836	0,029085402	1
ENSG00000235368	SAPCD2P2	suppressor APC domain containing 2 pseudogene 2 [Source:HGNC Symbol;Acc:HGNC:51278]	processed_pseudogene	2,335095628	-1,251802847	0,029098343	1
ENSG00000279592			TEC	18,80589599	1,062184901	0,029111336	1
ENSG00000157005	SST	somatostatin [Source:HGNC Symbol;Acc:HGNC:11329]	protein_coding	300,7582633	1,182154477	0,02917709	1
ENSG00000141946	ZIM3	zinc finger imprinted 3 [Source:HGNC Symbol;Acc:HGNC:16366]	protein_coding	2,490990632	-1,23984587	0,029192152	1
ENSG00000104783	KCNN4	potassium calcium-activated channel subfamily N member 4 [Source:HGNC Symbol;Acc:HGNC:6293]	protein_coding	10,91693905	-1,163572072	0,029193092	1
ENSG00000220256			lincRNA	4,374725527	-1,191965859	0,029247067	1
ENSG00000125816	NKX2-4	NK2 homeobox 4 [Source:HGNC Symbol;Acc:HGNC:7837]	protein_coding	9,177656781	-1,254850994	0,029288838	1
ENSG00000227001	NBPF2P	NBPF member 2, pseudogene [Source:HGNC Symbol;Acc:HGNC:31987]	unprocessed_pseudogene	21,60304423	-0,81094538	0,029408354	1
ENSG00000137766	UNC13C	unc-13 homolog C [Source:HGNC Symbol;Acc:HGNC:23149]	protein_coding	297,9652585	0,891801151	0,029414762	1
ENSG00000154478	GPR26	G protein-coupled receptor 26 [Source:HGNC Symbol;Acc:HGNC:4481]	protein_coding	86,01318696	1,213426748	0,029485351	1
ENSG00000214776			protein_coding	17,47474964	-1,240528365	0,029657081	1
ENSG00000197153	HIST1H3J	histone cluster 1 H3 family member j [Source:HGNC Symbol;Acc:HGNC:4774]	protein_coding	1207,634304	-0,985341214	0,029676861	1
ENSG00000234545	FAM133B	family with sequence similarity 133 member B [Source:HGNC Symbol;Acc:HGNC:28629]	protein_coding	332,8277038	-0,324355761	0,02967908	1
ENSG00000272020	RNU1-30P	RNA, U1 small nuclear 30, pseudogene [Source:HGNC Symbol;Acc:HGNC:48372]	snRNA	9,129574807	0,934099768	0,029764862	1
ENSG00000183662	FAM19A1	family with sequence similarity 19 member A1, C-C motif chemokine like [Source:HGNC Symbol;Acc:HGNC:21587]	protein_coding	72,64570957	1,195330862	0,029776246	1
ENSG00000186766	FOXI2	forkhead box I2 [Source:HGNC Symbol;Acc:HGNC:32448]	protein_coding	3,685904359	-1,224806774	0,029794942	1
ENSG00000265894	RN7SL357P	RNA, 7SL, cytoplasmic 357, pseudogene [Source:HGNC Symbol;Acc:HGNC:46373]	misc_RNA	2,173977591	-1,252236245	0,029825631	1
ENSG00000261628			unprocessed_pseudogene	1,432968914	-1,249381916	0,029840857	1
ENSG00000165379	LRFN5	leucine rich repeat and fibronectin type III domain containing 5 [Source:HGNC Symbol;Acc:HGNC:20360]	protein_coding	360,0065763	0,903119241	0,029845356	1
ENSG00000200184	RNU1-20P	RNA, U1 small nuclear 20, pseudogene [Source:HGNC Symbol;Acc:HGNC:41946]	snRNA	24,06636848	0,880690002	0,029860989	1
ENSG00000166188	ZNF319	zinc finger protein 319 [Source:HGNC Symbol;Acc:HGNC:13644]	protein_coding	255,3571352	0,397792174	0,029863789	1
ENSG00000164506	STXBP5	syntaxin binding protein 5 [Source:HGNC Symbol;Acc:HGNC:19665]	protein_coding	1150,7331244382	0,50056737	0,029876264	1
ENSG00000188856	RPSAP47	ribosomal protein SA pseudogene 47 [Source:HGNC Symbol;Acc:HGNC:36162]	processed_pseudogene	2,168018415	-1,203339289	0,029885341	1
ENSG00000113396	SLC27A6	solute carrier family 27 member 6 [Source:HGNC Symbol;Acc:HGNC:11000]	protein_coding	21,3277653	-1,011819327	0,029981392	1
ENSG00000102934	PLLP	plasmolipin [Source:HGNC Symbol;Acc:HGNC:18553]	protein_coding	85,80695548	0,949024283	0,030061378	1
ENSG00000267396			antisense_RNA	8,661514379	1,108380135	0,030086066	1
ENSG00000271964			antisense_RNA	4,07823269	1,000984679	0,030125978	1
ENSG00000173681	BCLAF3	BCLAF1 and THRAP3 family member 3 [Source:HGNC Symbol;Acc:HGNC:27413]	protein_coding	895,1588165	0,308081503	0,030167817	1
ENSG00000206805		Y RNA [Source:RFAM;Acc:RF00019]	misc_RNA	25,7181488	0,935754317	0,030189245	1
ENSG00000154537	FAM27C	family with sequence similarity 27 member C [Source:HGNC Symbol;Acc:HGNC:23668]	lincRNA	2,944431064	-1,123407228	0,030209368	1
ENSG00000279263	OR2L8	olfactory receptor family 2 subfamily L member 8 (gene/pseudogene) [Source:HGNC Symbol;Acc:HGNC:15014]	polymorphic_pseudogene	1,04519311	-1,191790533	0,030215516	1
ENSG00000009954	BAZ1B	bromodomain adjacent to zinc finger domain 1B [Source:HGNC Symbol;Acc:HGNC:961]	protein_coding	3897,547731	-0,295524497	0,030240146	1
ENSG00000207151		Y RNA [Source:RFAM;Acc:RF00019]	misc_RNA	5,177047885	1,04119068	0,030293321	1
ENSG00000127743	IL17B	interleukin 17B [Source:HGNC Symbol;Acc:HGNC:5982]	protein_coding	1,480378841	-1,214753933	0,030305361	1
ENSG00000261842			lincRNA	1,507053016	-1,200164746	0,030370995	1
ENSG00000165606	DRGX	dorsal root ganglia homeobox [Source:HGNC Symbol;Acc:HGNC:21536]	protein_coding	3,019538146	-1,19253812	0,030554653	1
ENSG00000254396			lincRNA	2,030609959	1,213850985	0,030619762	1
ENSG00000178928	TPRX1	tetrapeptide repeat homeobox 1 [Source:HGNC Symbol;Acc:HGNC:32174]	protein_coding	1,07800455	-1,239888681	0,030649886	1
ENSG00000232230	TPM4P1	tropomyosin 4 pseudogene 1 [Source:HGNC Symbol;Acc:HGNC:45234]	processed_pseudogene	1,478598698	-1,237801262	0,03066128	1
ENSG00000271976			antisense_RNA	160,3535106	0,385517485	0,030715769	1
ENSG00000241015	TPM3P9	tropomyosin 3 pseudogene 9 [Source:HGNC Symbol;Acc:HGNC:44142]	transcribed_processed_pseudogene	160,8485855	0,627835118	0,030718001	1
ENSG00000119333	WDR34	WD repeat domain 34 [Source:HGNC Symbol;Acc:HGNC:28296]	protein_coding	1504,808265	-0,422592156	0,030732599	1
ENSG00000170615	SLC26A5	solute carrier family 26 member 5 [Source:HGNC Symbol;Acc:HGNC:9359]	protein_coding	2,593844889	-1,238171152	0,030796026	1
ENSG00000263756			antisense_RNA	11,19350473	1,149391694	0,030816924	1
ENSG00000266751	MIR3661	microRNA 3661 [Source:HGNC Symbol;Acc:HGNC:38892]	miRNA	5,908644984	0,984731052	0,030943339	1
ENSG00000146221	TCTE1	t-complex-associated-testis-expressed 1 [Source:HGNC Symbol;Acc:HGNC:11693]	protein_coding	40,38596303	0,771282995	0,030974632	1

ENSG00000174498	IGDCC3	immunoglobulin superfamily DCC subclass member 3 [Source:HGNC Symbol;Acc:HGNC:9700]	protein_coding	424,8785906	-0,718868412	0,03103269	1
ENSG00000261435			lincRNA	1,429098584	-1,16521172	0,031114001	1
ENSG00000255587	RAB44	RAB44, member RAS oncogene family [Source:HGNC Symbol;Acc:HGNC:21068]	protein_coding	9,044582381	-1,241472247	0,031166409	1
ENSG00000252906	SCARNA3	small Cajal body-specific RNA 3 [Source:HGNC Symbol;Acc:HGNC:32577]	scaRNA	35,10563569	0,511221838	0,031189649	1
ENSG00000234886	MTND5P26	mitochondrially encoded NADH:ubiquinone oxidoreductase core subunit 5 pseudogene 26 [Source:HGNC Symbol;Acc:I	processed_pseudogene	2,538513132	-1,16356027	0,031231464	1
ENSG00000175463	TBC1D10C	TBC1 domain family member 10C [Source:HGNC Symbol;Acc:HGNC:24702]	protein_coding	9,278253685	-1,174178376	0,031263938	1
ENSG00000228482	LINC01713	long intergenic non-protein coding RNA 1713 [Source:HGNC Symbol;Acc:HGNC:52500]	lincRNA	1,560321106	-1,206897786	0,031277449	1
ENSG00000259939			antisense_RNA	16,02144283	0,939514756	0,031291064	1
ENSG00000223989			antisense_RNA	10,59185455	1,231020159	0,031326025	1
ENSG00000170035	UBE2E3	ubiquitin conjugating enzyme E2 E3 [Source:HGNC Symbol;Acc:HGNC:12479]	protein_coding	847,5794554	-0,570615488	0,031328782	1
ENSG00000216904	SOCS5P5	suppressor of cytokine signaling 5 pseudogene 5 [Source:HGNC Symbol;Acc:HGNC:44601]	processed_pseudogene	1,891951178	-1,240573146	0,03133839	1
ENSG00000155961	RAB39B	RAB39B, member RAS oncogene family [Source:HGNC Symbol;Acc:HGNC:16499]	protein_coding	644,8143522	0,379359812	0,031356919	1
ENSG00000260651			antisense_RNA	2,409824498	-1,240212626	0,031368809	1
ENSG00000205111	CDKL4	cyclin dependent kinase like 4 [Source:HGNC Symbol;Acc:HGNC:19287]	protein_coding	28,97912943	0,613131333	0,031436893	1
ENSG00000268015			antisense_RNA	13,51413101	1,156546995	0,031439757	1
ENSG00000272661			antisense_RNA	42,70950822	0,751343233	0,031441224	1
ENSG00000225405	RPS15AP17	ribosomal protein S15a pseudogene 17 [Source:HGNC Symbol;Acc:HGNC:36888]	processed_pseudogene	3,477084282	1,229314724	0,03160107	1
ENSG00000235151			lincRNA	3,5765262	-1,178365412	0,031611768	1
ENSG00000236064			antisense_RNA	9,174869422	-1,21572822	0,031617491	1
ENSG00000109158	GABRA4	gamma-aminobutyric acid type A receptor alpha4 subunit [Source:HGNC Symbol;Acc:HGNC:4078]	protein_coding	223,7735988	1,015953797	0,031654933	1
ENSG00000235872			antisense_RNA	10,17099465	1,109092309	0,031670499	1
ENSG00000164649	CDCA7L	cell division cycle associated 7 like [Source:HGNC Symbol;Acc:HGNC:30777]	protein_coding	1496,784263	-0,880141923	0,031673157	1
ENSG00000239474	KLHL41	kelch like family member 41 [Source:HGNC Symbol;Acc:HGNC:16905]	protein_coding	27,10765653	0,720941197	0,031747674	1
ENSG00000163673	DCLK3	doublecortin like kinase 3 [Source:HGNC Symbol;Acc:HGNC:19005]	protein_coding	94,4077518	1,154079861	0,031853925	1
ENSG00000009694	TENM1	teneurin transmembrane protein 1 [Source:HGNC Symbol;Acc:HGNC:8117]	protein_coding	702,0299184	0,531489026	0,031934645	1
ENSG00000176435	CLEC14A	C-type lectin domain containing 14A [Source:HGNC Symbol;Acc:HGNC:19832]	protein_coding	1,733488231	-1,207416984	0,031979855	1
ENSG00000234206			antisense_RNA	2,107889326	-1,235914748	0,031980988	1
ENSG00000139910	NOVA1	NOVA alternative splicing regulator 1 [Source:HGNC Symbol;Acc:HGNC:7886]	protein_coding	1355,339904	0,889236606	0,032031101	1
ENSG00000224061			processed_pseudogene	3,56046335	1,119237956	0,032040325	1
ENSG00000234534	CSNK1G2P1	casein kinase 1 gamma 2 pseudogene 1 [Source:HGNC Symbol;Acc:HGNC:49739]	processed_pseudogene	1,903575082	-1,223791979	0,032163954	1
ENSG00000234575	CTSLP8	cathepsin L pseudogene 8 [Source:HGNC Symbol;Acc:HGNC:39084]	unprocessed_pseudogene	1,265539676	-1,212230067	0,032165667	1
ENSG00000200608	SNORD114-11	small nucleolar RNA, C/D box 114-11 [Source:HGNC Symbol;Acc:HGNC:32999]	snoRNA	3,070116258	1,134957334	0,032300887	1
ENSG00000258279	LINC00592	long intergenic non-protein coding RNA 592 [Source:HGNC Symbol;Acc:HGNC:27474]	lincRNA	2,145030624	-1,233240635	0,032313002	1
ENSG00000278886			TEC	6,726677987	1,004332165	0,032313801	1
ENSG00000249106			antisense_RNA	8,889365052	1,137462611	0,03237545	1
ENSG00000283047			unprocessed_pseudogene	1,576470121	-1,226510058	0,032440498	1
ENSG00000245870	LINC00682	long intergenic non-protein coding RNA 682 [Source:HGNC Symbol;Acc:HGNC:44466]	lincRNA	5,825349749	-1,202240263	0,032444567	1
ENSG00000169891	REPS2	RALBP1 associated Eps domain containing 2 [Source:HGNC Symbol;Acc:HGNC:9963]	protein_coding	214,4211241	0,957616648	0,032486293	1
ENSG00000142684	ZNF593	zinc finger protein 593 [Source:HGNC Symbol;Acc:HGNC:30943]	protein_coding	184,6114977	-0,425839966	0,032527224	1
ENSG00000258789			antisense_RNA	7,751803234	-1,07062729	0,03255147	1
ENSG00000243960			sense_overlapping	17,9536883381	0,655949905	0,032563458	1
ENSG00000267142			processed_pseudogene	1,252212104	-1,230705926	0,03260325	1
ENSG00000215193	PEX26	peroxisomal biogenesis factor 26 [Source:HGNC Symbol;Acc:HGNC:22965]	protein_coding	1643,812339	0,216012331	0,032609056	1
ENSG00000205420	KRT6A	keratin 6A [Source:HGNC Symbol;Acc:HGNC:6443]	protein_coding	1,696272659	-1,231556406	0,032641152	1
ENSG00000269987			lincRNA	10,5232697	1,166769962	0,032698222	1
ENSG00000261115	TMEM178B	transmembrane protein 178B [Source:HGNC Symbol;Acc:HGNC:44112]	protein_coding	3915,156479	0,575894703	0,032715572	1
ENSG00000168421	RHOH	ras homolog family member H [Source:HGNC Symbol;Acc:HGNC:686]	protein_coding	28,91305531	-1,18245925	0,032784573	1
ENSG00000249920	HNRNPA1P55	heterogeneous nuclear ribonucleoprotein A1 pseudogene 55 [Source:HGNC Symbol;Acc:HGNC:48785]	processed_pseudogene	1,833398255	-1,228836334	0,03282439	1
ENSG00000260934			antisense_RNA	7,737245288	1,120399386	0,032908494	1
ENSG00000264069	MIR3943	microRNA 3943 [Source:HGNC Symbol;Acc:HGNC:38888]	miRNA	4,345648773	1,228924641	0,03290993	1
ENSG00000166592	RRAD	RRAD, Ras related glycolysis inhibitor and calcium channel regulator [Source:HGNC Symbol;Acc:HGNC:10446]	protein_coding	24,94928312	-0,970691867	0,032951368	1
ENSG00000117594	HSD11B1	hydroxysteroid 11-beta dehydrogenase 1 [Source:HGNC Symbol;Acc:HGNC:5208]	protein_coding	1,5851284	-1,228188397	0,032962481	1
ENSG00000151150	ANK3	ankyrin 3 [Source:HGNC Symbol;Acc:HGNC:494]	protein_coding	2323,72356	0,777181409	0,032981048	1
ENSG00000251562	MALAT1	metastasis associated lung adenocarcinoma transcript 1 (non-protein coding) [Source:HGNC Symbol;Acc:HGNC:29665]	lincRNA	49310,8874566084	0,74243368	0,032997726	1
ENSG00000197410	DCHS2	dachsous cadherin-related 2 [Source:HGNC Symbol;Acc:HGNC:23111]	protein_coding	277,163045	0,844339405	0,033046073	1
ENSG00000187999	HNRNPA1P61	heterogeneous nuclear ribonucleoprotein A1 pseudogene 61 [Source:HGNC Symbol;Acc:HGNC:48791]	processed_pseudogene	1,747597796	-1,225301197	0,033088553	1
ENSG00000257809			antisense_RNA	14,10640189	1,008752813	0,033095993	1
ENSG00000267275			antisense_RNA	1,325628162	-1,222427432	0,033101137	1
ENSG00000229111	MED4-AS1	MED4 antisense RNA 1 [Source:HGNC Symbol;Acc:HGNC:39213]	antisense_RNA	12,46828686	1,202800861	0,033121382	1
ENSG00000170122	FOXD4	forkhead box D4 [Source:HGNC Symbol;Acc:HGNC:3805]	protein_coding	31,47982642	0,810858386	0,033155841	1
ENSG00000132915	PDE6A	phosphodiesterase 6A [Source:HGNC Symbol;Acc:HGNC:8785]	protein_coding	8,084697157	-1,181768707	0,033221963	1
ENSG00000130427	EPO	erythropoietin [Source:HGNC Symbol;Acc:HGNC:3415]	protein_coding	3,799898532	-1,188649323	0,033247433	1
ENSG00000164100	NDST3	N-deacetylase and N-sulfotransferase 3 [Source:HGNC Symbol;Acc:HGNC:7682]	protein_coding	327,3495128	0,974682988	0,033271022	1
ENSG00000237298	TTN-AS1	TTN antisense RNA 1 [Source:HGNC Symbol;Acc:HGNC:44124]	antisense_RNA	593,3114212	0,662585624	0,033454265	1
ENSG00000277476			lincRNA	239,7996954	0,404036742	0,033476944	1
ENSG00000188056	TREML4	triggering receptor expressed on myeloid cells like 4 [Source:HGNC Symbol;Acc:HGNC:30807]	protein_coding	3,406434635	-1,225095037	0,033524393	1
ENSG00000254392			processed_pseudogene	1,932652189	1,208803367	0,03353223	1

ENSG00000230221			processed_pseudogene	1,906829053	-1,192820645	0,03361657	1
ENSG00000259471	LINC01169	long intergenic non-protein coding RNA 1169 [Source:HGNC Symbol;Acc:HGNC:49541]	lincRNA	1,219449246	-1,223319269	0,033624279	1
ENSG00000250826	HNRNPA3P13	heterogeneous nuclear ribonucleoprotein A3 pseudogene 13 [Source:HGNC Symbol;Acc:HGNC:48759]	processed_pseudogene	3,224049705	-1,22147122	0,033633507	1
ENSG00000162699	DNAJA1P5	DnaJ heat shock protein family (Hsp40) member A1 pseudogene 5 [Source:HGNC Symbol;Acc:HGNC:39341]	processed_pseudogene	1,279648496	-1,212718355	0,033677657	1
ENSG00000143793	C1orf35	chromosome 1 open reading frame 35 [Source:HGNC Symbol;Acc:HGNC:19032]	protein_coding	377,6456207	-0,422779206	0,033726514	1
ENSG00000166527	CLEC4D	C-type lectin domain family 4 member D [Source:HGNC Symbol;Acc:HGNC:14554]	protein_coding	1,755267221	-1,216910055	0,033727126	1
ENSG00000170128	GPR25	G protein-coupled receptor 25 [Source:HGNC Symbol;Acc:HGNC:4480]	protein_coding	2,805481285	-1,219017424	0,033769449	1
ENSG00000214595	EML6	echinoderm microtubule associated protein like 6 [Source:HGNC Symbol;Acc:HGNC:35412]	protein_coding	376,7266784	0,91436487	0,033838089	1
ENSG00000170085	SIMC1	SUMO interacting motifs containing 1 [Source:HGNC Symbol;Acc:HGNC:24779]	protein_coding	267,7901046	-0,394162015	0,03384824	1
ENSG00000113141	IK	IK cytokine [Source:HGNC Symbol;Acc:HGNC:5958]	protein_coding	2244,192155	-0,157368429	0,033867668	1
ENSG00000258131			lincRNA	3,363576325	-1,222251497	0,033919942	1
ENSG00000278124	RN7SL186P	RNA, 7SL, cytoplasmic 186, pseudogene [Source:HGNC Symbol;Acc:HGNC:46202]	misc_RNA	1,389433518	1,218975581	0,033952855	1
ENSG00000170379	TCAF2	TRPM8 channel associated factor 2 [Source:HGNC Symbol;Acc:HGNC:26878]	protein_coding	11,06521963	0,976617375	0,034005779	1
ENSG00000264916	RN7SL230P	RNA, 7SL, cytoplasmic 230, pseudogene [Source:HGNC Symbol;Acc:HGNC:46246]	misc_RNA	51,62436628	0,663040339	0,034015232	1
ENSG00000250416	SEC63P2	SEC63 homolog, protein translocation regulator pseudogene 2 [Source:HGNC Symbol;Acc:HGNC:41954]	processed_pseudogene	2,265374345	-1,221901339	0,034024939	1
ENSG00000151474	FRMD4A	FERM domain containing 4A [Source:HGNC Symbol;Acc:HGNC:25491]	protein_coding	2004,387645	0,545520624	0,034037057	1
ENSG00000111816	FRK	fyn related Src family tyrosine kinase [Source:HGNC Symbol;Acc:HGNC:3955]	protein_coding	20,18816463	-1,102933222	0,034052328	1
ENSG00000246211	P4HA3-AS1	P4HA3 antisense RNA 1 [Source:HGNC Symbol;Acc:HGNC:53160]	lincRNA	1,206039412	-1,180978232	0,034076789	1
ENSG00000050426	LETMD1	LETM1 domain containing 1 [Source:HGNC Symbol;Acc:HGNC:24241]	protein_coding	474,5429462	0,51921657	0,034092473	1
ENSG00000262681			lincRNA	8,76934121	-0,932179903	0,034131634	1
ENSG00000165646	SLC18A2	solute carrier family 18 member A2 [Source:HGNC Symbol;Acc:HGNC:10935]	protein_coding	30,70611636	1,028051827	0,034149536	1
ENSG00000111275	ALDH2	aldehyde dehydrogenase 2 family (mitochondrial) [Source:HGNC Symbol;Acc:HGNC:404]	protein_coding	1119,874453	0,34991832	0,034168265	1
ENSG00000164532	TBX20	T-box 20 [Source:HGNC Symbol;Acc:HGNC:11598]	protein_coding	3,397898671	-1,219905339	0,034202625	1
ENSG00000205078	SYCE1L	synaptonemal complex central element protein 1 like [Source:HGNC Symbol;Acc:HGNC:37236]	protein_coding	111,918214681	-0,862773505	0,034261287	1
ENSG00000230387			lincRNA	4,372016455	-1,218857806	0,034267301	1
ENSG00000267938	EIF1P6	eukaryotic translation initiation factor 1 pseudogene 6 [Source:HGNC Symbol;Acc:HGNC:49619]	unprocessed_pseudogene	3,215566735	-1,170891087	0,034283461	1
ENSG00000227210			antisense_RNA	6,744001247	-0,932660057	0,034300623	1
ENSG00000261003			sense_overlapping	17,82162918	0,866247375	0,034340102	1
ENSG00000237481			antisense_RNA	7,683126607	1,11915869	0,034399535	1
ENSG00000104951	IL4I1	interleukin 4 induced 1 [Source:HGNC Symbol;Acc:HGNC:19094]	protein_coding	7,431334037	-1,032013038	0,034426872	1
ENSG00000198231	DDX42	DEAD-box helicase 42 [Source:HGNC Symbol;Acc:HGNC:18676]	protein_coding	1853,9762634546	0,283805283	0,034459523	1
ENSG00000080293	SCTR	secretin receptor [Source:HGNC Symbol;Acc:HGNC:10608]	protein_coding	7,370859774	-1,180240958	0,034488894	1
ENSG00000231417	IRX1P1	iroquois homeobox 1 pseudogene 1 [Source:HGNC Symbol;Acc:HGNC:33315]	unprocessed_pseudogene	2,130137052	-1,207593463	0,034504125	1
ENSG00000143319	ISG20L2	interferon stimulated exonuclease gene 20 like 2 [Source:HGNC Symbol;Acc:HGNC:25745]	protein_coding	451,7319684	-0,219310164	0,034630331	1
ENSG00000243364	EFNA4	ephrin A4 [Source:HGNC Symbol;Acc:HGNC:3224]	protein_coding	96,13362729	-0,858140735	0,034640128	1
ENSG00000017797	RALBP1	ralA binding protein 1 [Source:HGNC Symbol;Acc:HGNC:9841]	protein_coding	1074,999806	-0,351211278	0,034656884	1
ENSG00000234665			lincRNA	9,361853838	-1,121115111	0,03466276	1
ENSG00000257489			transcribed_unprocessed_pseudogene	9,568971348	-0,869187899	0,034676241	1
ENSG00000270574			antisense_RNA	30,58288229	0,796679641	0,03480502	1
ENSG00000019582	CD74	CD74 molecule [Source:HGNC Symbol;Acc:HGNC:1697]	protein_coding	99,02623326	-1,182198472	0,034806072	1
ENSG00000156687	UNC5D	unc-5 netrin receptor D [Source:HGNC Symbol;Acc:HGNC:18634]	protein_coding	869,8740149	0,718245727	0,03482016	1
ENSG00000171056	SOX7	SRY-box 7 [Source:HGNC Symbol;Acc:HGNC:18196]	protein_coding	5,866505534	-1,046567009	0,034854184	1
ENSG00000269802			antisense_RNA	2,102140082	-1,201350911	0,034868216	1
ENSG00000265630	GLUD1P8	glutamate dehydrogenase 1 pseudogene 8 [Source:HGNC Symbol;Acc:HGNC:23637]	unprocessed_pseudogene	4,648845816	0,98922327	0,034906357	1
ENSG00000267308	LINC01764	long intergenic non-protein coding RNA 1764 [Source:HGNC Symbol;Acc:HGNC:52553]	lincRNA	1,719288385	-1,1935196	0,034911034	1
ENSG00000260442	ATP2A1-AS1	ATP2A1 antisense RNA 1 [Source:HGNC Symbol;Acc:HGNC:51370]	antisense_RNA	33,517703	-0,721321447	0,034911851	1
ENSG00000284601			bidirectional_promoter_lncRNA	2,337514891	-1,212467076	0,034916314	1
ENSG00000237713			lincRNA	3,213339003	1,212208876	0,034983845	1
ENSG00000236953	ZDHHC20-IT1	ZDHHC20 intronic transcript 1 [Source:HGNC Symbol;Acc:HGNC:39900]	sense_intronic	3,744073911	-1,203319361	0,035005481	1
ENSG00000261624	NDUFB10P2	NADH:ubiquinone oxidoreductase subunit B10 pseudogene 2 [Source:HGNC Symbol;Acc:HGNC:52273]	processed_pseudogene	3,044107357	1,195126103	0,035095395	1
ENSG00000218226	TATDN2P2	TatD DNase domain containing 2 pseudogene 2 [Source:HGNC Symbol;Acc:HGNC:39255]	processed_pseudogene	1,247763728	1,210743594	0,035117864	1
ENSG00000196923	PDLIM7	PDZ and LIM domain 7 [Source:HGNC Symbol;Acc:HGNC:22958]	protein_coding	772,8401398	-0,726782321	0,035151599	1
ENSG00000120055	C10orf95	chromosome 10 open reading frame 95 [Source:HGNC Symbol;Acc:HGNC:25880]	protein_coding	7,958837581	-0,978920596	0,035180006	1
ENSG00000163794	UCN	urocortin [Source:HGNC Symbol;Acc:HGNC:12516]	protein_coding	32,24748063	-0,616082976	0,035199772	1
ENSG00000086619	ERO1B	endoplasmic reticulum oxidoreductase 1 beta [Source:HGNC Symbol;Acc:HGNC:14355]	protein_coding	225,3533418	0,715189051	0,035225806	1
ENSG00000274964			sense_intronic	15,60863013	0,851186334	0,03523833	1
ENSG00000065665	SEC61A2	Sec61 translocon alpha 2 subunit [Source:HGNC Symbol;Acc:HGNC:17702]	protein_coding	736,1442672	0,335160049	0,035318574	1
ENSG00000165140	FBP1	fructose-bisphosphatase 1 [Source:HGNC Symbol;Acc:HGNC:3606]	protein_coding	3,317865848	-1,149213805	0,03535137	1
ENSG00000250535	STK19B	serine/threonine kinase 19B (pseudogene) [Source:HGNC Symbol;Acc:HGNC:21668]	unprocessed_pseudogene	4,509001104	1,081437408	0,035409906	1
ENSG00000163071	SPATA18	spermatogenesis associated 18 [Source:HGNC Symbol;Acc:HGNC:29579]	protein_coding	97,24974489	-0,764694836	0,03542916	1
ENSG00000283071		Homo sapiens translation initiation factor IF-2-like (LOC107984640), mRNA. [Source:RefSeq mRNA;Acc:NM_00133023	protein_coding	2,797493129	-1,202566909	0,035453167	1
ENSG00000281508			antisense_RNA	2659,354668	-0,870472747	0,035545466	1
ENSG00000263986			antisense_RNA	48,47280986	1,063697485	0,035605829	1
ENSG00000254132	MTND6P3	mitochondrially encoded NADH:ubiquinone oxidoreductase core subunit 6 pseudogene 3 [Source:HGNC Symbol;Acc:H	processed_pseudogene	2,039456139	-1,193285057	0,035684133	1
ENSG00000072571	HMMR	hyaluronan mediated motility receptor [Source:HGNC Symbol;Acc:HGNC:5012]	protein_coding	617,2484504	-0,865739855	0,03573957	1
ENSG00000183734	ASCL2	achaete-scute family bHLH transcription factor 2 [Source:HGNC Symbol;Acc:HGNC:739]	protein_coding	14,56816958	-1,124326932	0,035781368	1
ENSG00000264044			antisense_RNA	13,98766558	1,048587362	0,035818461	1

ENSG00000272636	DOC2B	double C2 domain beta [Source:HGNC Symbol;Acc:HGNC:2986]	protein_coding	799,6746863	0,961281413	0,036036501	1
ENSG00000261040	WFDC21P	WAP four-disulfide core domain 21, pseudogene [Source:HGNC Symbol;Acc:HGNC:50357]	processed_transcript	2,376764423	1,156972769	0,036151778	1
ENSG00000139146	SINHCAF	SIN3-HDAC complex associated factor [Source:HGNC Symbol;Acc:HGNC:30702]	protein_coding	946,464279	-0,744875789	0,036159144	1
ENSG00000103034	NDRG4	NDRG family member 4 [Source:HGNC Symbol;Acc:HGNC:14466]	protein_coding	5629,175847	0,606756619	0,036200863	1
ENSG00000104432	IL7	interleukin 7 [Source:HGNC Symbol;Acc:HGNC:6023]	protein_coding	20,79340804	0,878185232	0,036223527	1
ENSG00000250909			antisense_RNA	11,44665413	1,111518925	0,036281409	1
ENSG00000234589			processed_pseudogene	4,214551976	-0,986160595	0,036305325	1
ENSG00000062524	LTK	leukocyte receptor tyrosine kinase [Source:HGNC Symbol;Acc:HGNC:6721]	protein_coding	6,584944971	-1,065077162	0,036311009	1
ENSG00000160973	FOXH1	forkhead box H1 [Source:HGNC Symbol;Acc:HGNC:3814]	protein_coding	15,46923734	1,055763536	0,036311202	1
ENSG00000137843	PAK6	p21 (RAC1) activated kinase 6 [Source:HGNC Symbol;Acc:HGNC:16061]	protein_coding	208,0855081	0,887162958	0,036325801	1
ENSG00000255087			lincRNA	2,789204386	-1,197810494	0,036370623	1
ENSG00000236051	MYCBP2-AS1	MYCBP2 antisense RNA 1 [Source:HGNC Symbol;Acc:HGNC:41023]	processed_transcript	14,47306497	1,018381818	0,03645945	1
ENSG00000270050			antisense_RNA	6,338060695	1,073066006	0,036496997	1
ENSG00000248356			antisense_RNA	1,849931281	1,203357731	0,036518331	1
ENSG00000223866			processed_pseudogene	19,74889336	-1,107325994	0,036520513	1
ENSG00000228549			lincRNA	19,76458938	-0,893379228	0,036686365	1
ENSG00000275672			antisense_RNA	6,99276955	-1,053199766	0,036702204	1
ENSG00000165682	CLEC1B	C-type lectin domain family 1 member B [Source:HGNC Symbol;Acc:HGNC:24356]	protein_coding	3,039738988	-1,203175277	0,036729786	1
ENSG00000161896	IP6K3	inositol hexakisphosphate kinase 3 [Source:HGNC Symbol;Acc:HGNC:17269]	protein_coding	4,457502566	-1,036169973	0,036734148	1
ENSG00000249383	LINC02071	long intergenic non-protein coding RNA 2071 [Source:HGNC Symbol;Acc:HGNC:52917]	lincRNA	3,026719394	-1,193780268	0,036739041	1
ENSG00000265749			antisense_RNA	5,375386857	1,107364492	0,036748871	1
ENSG00000261544			sense_intronic	4,642985888	1,128103439	0,036751018	1
ENSG00000202415	RN7SKP269	RNA, 7SK small nuclear pseudogene 269 [Source:HGNC Symbol;Acc:HGNC:45993]	misc_RNA	6,713996844	0,850833848	0,036780972	1
ENSG00000177453	NIM1K	NIM1 serine/threonine protein kinase [Source:HGNC Symbol;Acc:HGNC:28646]	protein_coding	123,9616888	0,80504163	0,036790249	1
ENSG00000055950	MRPL43	mitochondrial ribosomal protein L43 [Source:HGNC Symbol;Acc:HGNC:14517]	protein_coding	876,5742682	-0,365249548	0,036826059	1
ENSG00000250889	LINC01336	long intergenic non-protein coding RNA 1336 [Source:HGNC Symbol;Acc:HGNC:50543]	lincRNA	7,675707337	-0,899719581	0,036918059	1
ENSG00000166562	SEC11C	SEC11 homolog C, signal peptidase complex subunit [Source:HGNC Symbol;Acc:HGNC:23400]	protein_coding	1276,131843	0,332086464	0,036932789	1
ENSG00000130731	METTL26	methyltransferase like 26 [Source:HGNC Symbol;Acc:HGNC:14141]	protein_coding	625,9336272	-0,306159055	0,036997014	1
ENSG00000230289			antisense_RNA	4,370865997	1,173570113	0,037013004	1
ENSG00000233287			processed_pseudogene	1,667453335	-1,201173572	0,037039794	1
ENSG00000269119	HNRNPA1P52	heterogeneous nuclear ribonucleoprotein A1 pseudogene 52 [Source:HGNC Symbol;Acc:HGNC:48782]	processed_pseudogene	4,065052506	-1,042320081	0,03707027	1
ENSG00000261787	TCF24	transcription factor 24 [Source:HGNC Symbol;Acc:HGNC:32275]	protein_coding	4,842983688	-1,120769256	0,037079984	1
ENSG00000214132			processed_pseudogene	0,818778706	-1,089244322	0,037104366	1
ENSG00000199394	RNU6-600P	RNA, U6 small nuclear 600, pseudogene [Source:HGNC Symbol;Acc:HGNC:47563]	snRNA	1,916871432	-1,195803158	0,037159436	1
ENSG00000278673			pseudogene	4,182276111	-1,082116755	0,037182499	1
ENSG00000063438	AHRR	aryl-hydrocarbon receptor repressor [Source:HGNC Symbol;Acc:HGNC:346]	protein_coding	212,0417884	0,75190301	0,037192712	1
ENSG00000266036			antisense_RNA	3,916746603	-1,091733188	0,037236827	1
ENSG00000235016	SEMA3F-AS1	SEMA3F antisense RNA 1 [Source:HGNC Symbol;Acc:HGNC:40518]	antisense_RNA	23,1403232	0,92623345	0,037252628	1
ENSG00000137265	IRF4	interferon regulatory factor 4 [Source:HGNC Symbol;Acc:HGNC:6119]	protein_coding	8,909813085	-1,03974139	0,037277356	1
ENSG00000233021			antisense_RNA	1,089419066	1,163965151	0,037279771	1
ENSG00000102967	DHODH	dihydroorotate dehydrogenase (quinone) [Source:HGNC Symbol;Acc:HGNC:2867]	protein_coding	217,8216642	-0,358834926	0,037349716	1
ENSG00000157734	SNX22	sorting nexin 22 [Source:HGNC Symbol;Acc:HGNC:16315]	protein_coding	243,5526501	0,649814813	0,03735577	1
ENSG00000166483	WEE1	WEE1 G2 checkpoint kinase [Source:HGNC Symbol;Acc:HGNC:12761]	protein_coding	1152,65093	-0,567108978	0,03737642	1
ENSG00000054277	OPN3	opsin 3 [Source:HGNC Symbol;Acc:HGNC:14007]	protein_coding	151,9709914	-0,864870239	0,03741	1
ENSG00000203650	LINC01285	long intergenic non-protein coding RNA 1285 [Source:HGNC Symbol;Acc:HGNC:50344]	lincRNA	3,145744208	-1,105210193	0,037429034	1
ENSG00000235563			antisense_RNA	4,611049214	-1,19775108	0,037451397	1
ENSG00000108384	RAD51C	RAD51 paralog C [Source:HGNC Symbol;Acc:HGNC:9820]	protein_coding	304,8810897	-0,364030141	0,03754099	1
ENSG00000165868	HSPA12A	heat shock protein family A (Hsp70) member 12A [Source:HGNC Symbol;Acc:HGNC:19022]	protein_coding	527,2719428	0,656934373	0,037548902	1
ENSG00000162980	ARL5A	ADP ribosylation factor like GTPase 5A [Source:HGNC Symbol;Acc:HGNC:696]	protein_coding	1232,703699	-0,227605664	0,037583264	1
ENSG00000233799			antisense_RNA	10,6094089	0,808803734	0,037644437	1
ENSG00000234696	GPR50-AS1	GPR50 antisense RNA 1 [Source:HGNC Symbol;Acc:HGNC:40259]	antisense_RNA	1,672810635	-1,170147632	0,037665423	1
ENSG00000143512	HHIPL2	HHIP like 2 [Source:HGNC Symbol;Acc:HGNC:25842]	protein_coding	8,711161444	-0,977827969	0,037696623	1
ENSG00000146039	SLC17A4	solute carrier family 17 member 4 [Source:HGNC Symbol;Acc:HGNC:10932]	protein_coding	13,30181698	-1,182923433	0,037711455	1
ENSG00000102802	MEDAG	mesenteric estrogen dependent adipogenesis [Source:HGNC Symbol;Acc:HGNC:25926]	protein_coding	6,358152205	-1,167592874	0,037740206	1
ENSG00000251273	LINC02228	long intergenic non-protein coding RNA 2228 [Source:HGNC Symbol;Acc:HGNC:53097]	lincRNA	9,609627777	-0,801961979	0,037748302	1
ENSG00000273262			antisense_RNA	11,62129518	1,09618775	0,037756415	1
ENSG00000142765	SYTL1	synaptotagmin like 1 [Source:HGNC Symbol;Acc:HGNC:15584]	protein_coding	18,16781345	-1,09157614	0,037788721	1
ENSG00000174326	SLC16A11	solute carrier family 16 member 11 [Source:HGNC Symbol;Acc:HGNC:23093]	protein_coding	5,652035526	-1,071271236	0,037840581	1
ENSG00000240668	KRT8P36	keratin 8 pseudogene 36 [Source:HGNC Symbol;Acc:HGNC:39870]	processed_pseudogene	3,335932289	-1,108243911	0,037889887	1
ENSG00000163518	FCRL4	Fc receptor like 4 [Source:HGNC Symbol;Acc:HGNC:18507]	protein_coding	2,255271597	-1,196702664	0,03789329	1
ENSG00000244183	PPIAP71	peptidylprolyl isomerase A pseudogene 71 [Source:HGNC Symbol;Acc:HGNC:53695]	processed_pseudogene	1,944055958	-1,189862148	0,037900174	1
ENSG00000222844	RNU6-321P	RNA, U6 small nuclear 321, pseudogene [Source:HGNC Symbol;Acc:HGNC:47284]	snRNA	1,178946594	-1,187591634	0,03798205	1
ENSG00000228478			lincRNA	0,986474174	-1,097116106	0,037983382	1
ENSG00000243284	VSIG8	V-set and immunoglobulin domain containing 8 [Source:HGNC Symbol;Acc:HGNC:32063]	protein_coding	2,685706796	-1,13673158	0,037987189	1
ENSG00000124232	RBPJL	recombination signal binding protein for immunoglobulin kappa J region like [Source:HGNC Symbol;Acc:HGNC:13761]	protein_coding	5,314051187	-1,147330593	0,037994338	1
ENSG00000273455			antisense_RNA	1,877779089	1,193624605	0,038097961	1
ENSG00000280321			TEC	7,094873301	-0,887785069	0,038120604	1

ENSG00000255120	OVOL1-AS1	OVOL1 antisense RNA 1 [Source:HGNC Symbol;Acc:HGNC:49319]	antisense_RNA	3,319024724	-1,19007274	0,038149292	1
ENSG00000279232			antisense_RNA	32,87245563	1,088504889	0,038167074	1
ENSG00000224914	LINC00863	long intergenic non-protein coding RNA 863 [Source:HGNC Symbol;Acc:HGNC:45162]	lincRNA	131,1980128	-0,45833488	0,03818933	1
ENSG00000226490			protein_coding	2,637362194	-1,193914841	0,038194318	1
ENSG00000238033			lincRNA	3,93828099	-1,194957731	0,038200763	1
ENSG00000176024	ZNF613	zinc finger protein 613 [Source:HGNC Symbol;Acc:HGNC:25827]	protein_coding	282,0139045	0,414452942	0,038209406	1
ENSG00000205176	REXO1L1P	REXO1 like 1, pseudogene [Source:HGNC Symbol;Acc:HGNC:24660]	transcribed_processed_pseudogene	4,735507672	-1,192985632	0,038219418	1
ENSG00000276210	LINC00226	long intergenic non-protein coding RNA 226 [Source:HGNC Symbol;Acc:HGNC:20168]	lincRNA	1,351953901	-1,083798043	0,038356866	1
ENSG00000163235	TGFA	transforming growth factor alpha [Source:HGNC Symbol;Acc:HGNC:11765]	protein_coding	56,23735985	1,092434702	0,038510325	1
ENSG00000227487	NCAM1-AS1	NCAM1 antisense RNA1 [Source:HGNC Symbol;Acc:HGNC:48675]	antisense_RNA	21,38523417	1,051469526	0,038535802	1
ENSG00000106302	HYAL4	hyaluronoglucosaminidase 4 [Source:HGNC Symbol;Acc:HGNC:5323]	protein_coding	2,549186159	-1,19032577	0,038537389	1
ENSG00000176868			antisense_RNA	52,89647547	0,904440389	0,038569979	1
ENSG00000284237			lincRNA	9,574324411	-1,147304465	0,038588647	1
ENSG00000173141	MRPL57	mitochondrial ribosomal protein L57 [Source:HGNC Symbol;Acc:HGNC:14514]	protein_coding	835,6060462	-0,246374764	0,038626061	1
ENSG00000198108	CHSY3	chondroitin sulfate synthase 3 [Source:HGNC Symbol;Acc:HGNC:24293]	protein_coding	174,9480229	0,602870654	0,038640576	1
ENSG00000101292	PROKR2	prokineticin receptor 2 [Source:HGNC Symbol;Acc:HGNC:15836]	protein_coding	57,54022641	1,183414325	0,03866476	1
ENSG00000169507	SLC38A11	solute carrier family 38 member 11 [Source:HGNC Symbol;Acc:HGNC:26836]	protein_coding	6,633753837	-1,186961611	0,038769914	1
ENSG00000199059	MIR135B	microRNA 135b [Source:HGNC Symbol;Acc:HGNC:31760]	miRNA	4,601721938	1,007011819	0,038800464	1
ENSG00000173482	PTPRM	protein tyrosine phosphatase, receptor type M [Source:HGNC Symbol;Acc:HGNC:9675]	protein_coding	2366,278426	0,478869225	0,038829987	1
ENSG00000234497	ERICH3-AS1	ERICH3 antisense RNA 1 [Source:HGNC Symbol;Acc:HGNC:41093]	antisense_RNA	3,772310242	-1,187258133	0,038857884	1
ENSG00000197245	FAM110D	family with sequence similarity 110 member D [Source:HGNC Symbol;Acc:HGNC:25860]	protein_coding	15,70480708	-0,937665051	0,038867587	1
ENSG00000127561	SYNGR3	synaptogyrin 3 [Source:HGNC Symbol;Acc:HGNC:11501]	protein_coding	525,4035838	0,627497187	0,038920061	1
ENSG00000121316	PLBD1	phospholipase B domain containing 1 [Source:HGNC Symbol;Acc:HGNC:26215]	protein_coding	8,15420042	-1,052530961	0,03893513	1
ENSG00000184058	TBX1	T-box 1 [Source:HGNC Symbol;Acc:HGNC:11592]	protein_coding	56,31587011	-1,187238508	0,039013152	1
ENSG00000196290	NIF3L1	NGG1 interacting factor 3 like 1 [Source:HGNC Symbol;Acc:HGNC:13390]	protein_coding	521,712116	-0,292921825	0,039030911	1
ENSG00000199223		Y RNA [Source:RFAM;Acc:RF00019]	misc_RNA	4,452038085	1,027170371	0,039069861	1
ENSG00000239923	RN7SL864P	RNA, 7SL, cytoplasmic 864, pseudogene [Source:HGNC Symbol;Acc:HGNC:46880]	misc_RNA	1,736126346	1,183910269	0,039104508	1
ENSG00000138778	CENPE	centromere protein E [Source:HGNC Symbol;Acc:HGNC:1856]	protein_coding	1292,259026	-0,738508727	0,039165476	1
ENSG00000123146	ADGRE5	adhesion G protein-coupled receptor E5 [Source:HGNC Symbol;Acc:HGNC:1711]	protein_coding	29,44051621	-0,819017898	0,039171886	1
ENSG00000284632			lincRNA	2,860381115	-1,181394441	0,039284922	1
ENSG00000198128	OR2L3	olfactory receptor family 2 subfamily L member 3 [Source:HGNC Symbol;Acc:HGNC:15009]	protein_coding	3,751209259	-1,174109866	0,039314727	1
ENSG00000061455	PRDM6	PR/SET domain 6 [Source:HGNC Symbol;Acc:HGNC:9350]	protein_coding	13,53112988	-0,930748363	0,039328373	1
ENSG00000267023	LRRC37A16P	leucine rich repeat containing 37 member A16, pseudogene [Source:HGNC Symbol;Acc:HGNC:43820]	transcribed_unprocessed_pseudogene	41,74261125	1,08902921	0,039475683	1
ENSG00000181291	TMEM132E	transmembrane protein 132E [Source:HGNC Symbol;Acc:HGNC:26991]	protein_coding	97,27826519	-0,896142068	0,039612247	1
ENSG00000179855	GIPC3	GIPC PDZ domain containing family member 3 [Source:HGNC Symbol;Acc:HGNC:18183]	protein_coding	2,125120581	-1,185113387	0,039647408	1
ENSG00000130182	ZSCAN10	zinc finger and SCAN domain containing 10 [Source:HGNC Symbol;Acc:HGNC:12997]	protein_coding	2,49640214	-1,181771886	0,039687902	1
ENSG00000251259			lincRNA	2,549348061	1,154268987	0,039705783	1
ENSG00000164362	TERT	telomerase reverse transcriptase [Source:HGNC Symbol;Acc:HGNC:11730]	protein_coding	7,041332768	-1,173068465	0,039752988	1
ENSG00000061656	SPAG4	sperm associated antigen 4 [Source:HGNC Symbol;Acc:HGNC:11214]	protein_coding	22,39340226	-0,659736876	0,039765758	1
ENSG00000232759			antisense_RNA	9,204773035	-1,099112154	0,039768844	1
ENSG00000153044	CENPH	centromere protein H [Source:HGNC Symbol;Acc:HGNC:17268]	protein_coding	503,4735979	-0,69390779	0,039789424	1
ENSG00000226363	HAGLROS	HAGLR opposite strand (non-protein coding) [Source:HGNC Symbol;Acc:HGNC:50646]	lincRNA	2,519241747	-1,166379084	0,039799206	1
ENSG00000143321	HDGF	heparin binding growth factor [Source:HGNC Symbol;Acc:HGNC:4856]	protein_coding	5201,70447	-0,396510624	0,039801147	1
ENSG00000234478	ACBD3-AS1	ACBD3 antisense RNA 1 [Source:HGNC Symbol;Acc:HGNC:40701]	antisense_RNA	9,806532731	1,132827792	0,039859972	1
ENSG00000267731			lincRNA	19,64969677	0,788148285	0,039863658	1
ENSG00000228748			antisense_RNA	24,91126446	1,102760804	0,039885826	1
ENSG00000162398	LEXM	lymphocyte expansion molecule [Source:HGNC Symbol;Acc:HGNC:26854]	protein_coding	3,269394249	-1,177683698	0,039888758	1
ENSG00000138769	CDKL2	cyclin dependent kinase like 2 [Source:HGNC Symbol;Acc:HGNC:1782]	protein_coding	403,3439467	0,61540897	0,039920441	1
ENSG00000171735	CAMTA1	calmodulin binding transcription activator 1 [Source:HGNC Symbol;Acc:HGNC:18806]	protein_coding	2240,878629	0,325102804	0,039922202	1
ENSG00000204644	ZFP57	ZFP57 zinc finger protein [Source:HGNC Symbol;Acc:HGNC:18791]	protein_coding	1,825882615	-1,183510704	0,040066009	1
ENSG00000186790	FOXE3	forkhead box E3 [Source:HGNC Symbol;Acc:HGNC:3808]	protein_coding	6,572605296	-1,141828758	0,040096811	1
ENSG00000256512			antisense_RNA	8,218154908	1,150194043	0,040121622	1
ENSG00000263293		thyroid cancer-associated transcript 158 [Source:NCBI gene;Acc:102724508]	antisense_RNA	4,448123608	-1,097848989	0,040128085	1
ENSG00000281376	ABALON	apoptotic BCL2L1-antisense long non-coding RNA [Source:HGNC Symbol;Acc:HGNC:49667]	antisense_RNA	23,19969175	0,824003057	0,040156578	1
ENSG00000272791			lincRNA	14,41611482	0,692876262	0,040157944	1
ENSG00000185313	SCN10A	sodium voltage-gated channel alpha subunit 10 [Source:HGNC Symbol;Acc:HGNC:10582]	protein_coding	4,607861963	-1,183068623	0,040162126	1
ENSG00000259780			lincRNA	9,730766735	0,888107756	0,040307749	1
ENSG00000167995	BEST1	bestrophin 1 [Source:HGNC Symbol;Acc:HGNC:12703]	protein_coding	199,184654	0,837385109	0,040341539	1
ENSG00000164398	ACSL6	acyl-CoA synthetase long chain family member 6 [Source:HGNC Symbol;Acc:HGNC:16496]	protein_coding	359,5918464	0,78200251	0,040565097	1
ENSG00000227733			lincRNA	3,909456118	-1,180200939	0,040623112	1
ENSG00000245685	FRG1-DT	FRG1 divergent transcript [Source:HGNC Symbol;Acc:HGNC:51590]	lincRNA	22,85112156	0,601237769	0,040745165	1
ENSG00000178297	TMPRSS9	transmembrane protease, serine 9 [Source:HGNC Symbol;Acc:HGNC:30079]	protein_coding	10,20039634	-1,107524935	0,040745623	1
ENSG00000233293			antisense_RNA	1,723584763	1,169453835	0,04079036	1
ENSG00000221878	PSG7	pregnancy specific beta-1-glycoprotein 7 (gene/pseudogene) [Source:HGNC Symbol;Acc:HGNC:9524]	protein_coding	1,943406982	-1,154067421	0,04082267	1
ENSG00000265188			processed_pseudogene	4,922048397	1,151627679	0,040824786	1
ENSG00000198547	C20orf203	chromosome 20 open reading frame 203 [Source:HGNC Symbol;Acc:HGNC:26592]	lincRNA	34,25337325	0,656720538	0,040921413	1
ENSG00000243738	RN7SL181P	RNA, 7SL, cytoplasmic 181, pseudogene [Source:HGNC Symbol;Acc:HGNC:46197]	misc_RNA	20,70125567	0,76143326	0,04093074	1

ENSG00000258232			antisense_RNA	5,009178842	1,173966501	0,040931339	1
ENSG00000107105	ELAVL2	ELAV like RNA binding protein 2 [Source:HGNC Symbol;Acc:HGNC:3313]	protein_coding	3071,857559	0,529589725	0,04093269	1
ENSG00000128713	HOXD11	homeobox D11 [Source:HGNC Symbol;Acc:HGNC:5134]	protein_coding	1,559625252	-1,084140995	0,040980694	1
ENSG00000157335	CLEC18C	C-type lectin domain family 18 member C [Source:HGNC Symbol;Acc:HGNC:28538]	protein_coding	8,30778193	-1,176919355	0,040995653	1
ENSG00000201839	SNORD114-3	small nucleolar RNA, C/D box 114-3 [Source:HGNC Symbol;Acc:HGNC:32991]	snoRNA	18,23653586	1,072078875	0,041040159	1
ENSG00000234661	CHL1-AS1	CHL1 antisense RNA 1 [Source:HGNC Symbol;Acc:HGNC:40148]	antisense_RNA	38,44809524	1,09738719	0,041158895	1
ENSG00000275741			antisense_RNA	6,712029807	-1,047699796	0,041169767	1
ENSG00000261997			lincRNA	5,968490113	-0,956649519	0,041172881	1
ENSG00000279094	LINC01670	long intergenic non-protein coding RNA 1670 [Source:HGNC Symbol;Acc:HGNC:52458]	lincRNA	1,685668527	-1,168888374	0,041247491	1
ENSG00000070731	ST6GALNAC2	ST6 N-acetylgalactosaminide alpha-2,6-sialyltransferase 2 [Source:HGNC Symbol;Acc:HGNC:10867]	protein_coding	4,591703452	-1,0013266	0,041262022	1
ENSG00000238045			antisense_RNA	107,4872544	0,823734534	0,041302937	1
ENSG00000200169	RNU5D-1	RNA, U5D small nuclear 1 [Source:HGNC Symbol;Acc:HGNC:10214]	snRNA	26,04399806	-0,913164294	0,041348059	1
ENSG00000243437	RN7SL370P	RNA, 7SL, cytoplasmic 370, pseudogene [Source:HGNC Symbol;Acc:HGNC:46386]	misc_RNA	2,065443123	-1,172337895	0,041369796	1
ENSG00000160994	CCDC105	coiled-coil domain containing 105 [Source:HGNC Symbol;Acc:HGNC:26866]	protein_coding	3,545947136	-1,168833343	0,041392349	1
ENSG00000143416	SELENBP1	selenium binding protein 1 [Source:HGNC Symbol;Acc:HGNC:10719]	protein_coding	369,8994417	-0,554690289	0,041404674	1
ENSG00000011260	UTP18	UTP18, small subunit processome component [Source:HGNC Symbol;Acc:HGNC:24274]	protein_coding	576,9826265	-0,376705186	0,041412968	1
ENSG00000274092			antisense_RNA	3,226218209	-1,173028626	0,041472991	1
ENSG00000246740	PLA2G4E-AS1	PLA2G4E antisense RNA 1 [Source:HGNC Symbol;Acc:HGNC:51419]	antisense_RNA	2,325772163	-1,159332103	0,04152429	1
ENSG00000232043			antisense_RNA	10,00729932	0,974349657	0,041529077	1
ENSG00000240972	MIF	macrophage migration inhibitory factor [Source:HGNC Symbol;Acc:HGNC:7097]	protein_coding	1238,750147	-0,653182373	0,041539559	1
ENSG00000140481	CCDC33	coiled-coil domain containing 33 [Source:HGNC Symbol;Acc:HGNC:26552]	protein_coding	13,693404	-1,051617299	0,041551055	1
ENSG00000228737			antisense_RNA	7,004304686	1,069223505	0,041551554	1
ENSG00000279818			TEC	3,367643021	-1,169952336	0,041555642	1
ENSG00000132205	EMILIN2	elastin microfibril interfacer 2 [Source:HGNC Symbol;Acc:HGNC:19881]	protein_coding	205,6028787	-1,151093616	0,041591672	1
ENSG00000267042			antisense_RNA	3,808631473	-0,949436791	0,041645457	1
ENSG00000259705			lincRNA	21,24462004	-0,809130887	0,041729408	1
ENSG00000134954	ETS1	ETS proto-oncogene 1, transcription factor [Source:HGNC Symbol;Acc:HGNC:3488]	protein_coding	202,1872086	1,169028919	0,041774423	1
ENSG00000244588	RAD21L1	RAD21 cohesin complex component like 1 [Source:HGNC Symbol;Acc:HGNC:16271]	protein_coding	2,1860541	-1,173426674	0,041774857	1
ENSG00000213839	TMX2P1	thioredoxin related transmembrane protein 2 pseudogene 1 [Source:HGNC Symbol;Acc:HGNC:49916]	processed_pseudogene	8,802272399	0,893348321	0,041778578	1
ENSG00000263218			antisense_RNA	2,114367396	1,14955452	0,041820847	1
ENSG00000108849	PPY	pancreatic polypeptide [Source:HGNC Symbol;Acc:HGNC:9327]	protein_coding	1,713728709	-1,171738121	0,041849888	1
ENSG00000264029			unprocessed_pseudogene	3,713026062	-1,144047359	0,04185546	1
ENSG00000233033	CASK-AS1	CASK antisense RNA 1 [Source:HGNC Symbol;Acc:HGNC:40126]	antisense_RNA	6,020223774	1,169574747	0,04190599	1
ENSG00000257195	HNRNPA1P50	heterogeneous nuclear ribonucleoprotein A1 pseudogene 50 [Source:HGNC Symbol;Acc:HGNC:48780]	processed_pseudogene	1,744359339	-1,16314462	0,041937322	1
ENSG00000199440		Y RNA [Source:RFAM;Acc:RF00019]	misc_RNA	1,478352906	1,169944019	0,042008346	1
ENSG00000136531	SCN2A	sodium voltage-gated channel alpha subunit 2 [Source:HGNC Symbol;Acc:HGNC:10588]	protein_coding	2405,821081	0,740684451	0,042046095	1
ENSG00000188162	OTOG	otogelin [Source:HGNC Symbol;Acc:HGNC:8516]	protein_coding	15,75573472	-1,010176422	0,042054653	1
ENSG00000275302	CCL4	C-C motif chemokine ligand 4 [Source:HGNC Symbol;Acc:HGNC:10630]	protein_coding	1,713934293	-1,159224079	0,042058536	1
ENSG00000260903	XKR7	XK related 7 [Source:HGNC Symbol;Acc:HGNC:23062]	protein_coding	920,1882497	0,672940023	0,042112819	1
ENSG00000251739	RNU6-1053P	RNA, U6 small nuclear 1053, pseudogene [Source:HGNC Symbol;Acc:HGNC:48016]	snRNA	3,8777249	1,040481871	0,042113702	1
ENSG00000275097			3prime_overlapping_ncRNA	1,026769475	-1,171165787	0,042116788	1
ENSG00000230149			antisense_RNA	8,358518983	0,847025351	0,042132844	1
ENSG00000125798	FOXA2	forkhead box A2 [Source:HGNC Symbol;Acc:HGNC:5022]	protein_coding	2,116199853	-1,170183197	0,04214077	1
ENSG00000244165	P2RY11	purinergic receptor P2Y11 [Source:HGNC Symbol;Acc:HGNC:8540]	protein_coding	52,94347551	0,661811517	0,042198153	1
ENSG00000256315			processed_transcript	3,795102468	1,139503375	0,042238384	1
ENSG00000277873			antisense_RNA	14,22812492	0,696052112	0,042243054	1
ENSG00000204209	DAXX	death domain associated protein [Source:HGNC Symbol;Acc:HGNC:2681]	protein_coding	1020,835751	-0,254401565	0,042245008	1
ENSG00000180861	LINC01559	long intergenic non-protein coding RNA 1559 [Source:HGNC Symbol;Acc:HGNC:26598]	lincRNA	3,238955335	-1,155540539	0,042245958	1
ENSG00000185352	HS6ST3	heparan sulfate 6-O-sulfotransferase 3 [Source:HGNC Symbol;Acc:HGNC:19134]	protein_coding	509,1169118	0,895138352	0,042255657	1
ENSG00000165392	WRN	Werner syndrome RecQ like helicase [Source:HGNC Symbol;Acc:HGNC:12791]	protein_coding	667,3984921	-0,547739134	0,042271108	1
ENSG00000242659			lincRNA	4,424894736	1,07604701	0,042284466	1
ENSG00000264364	DYNLL2	dynein light chain LC8-type 2 [Source:HGNC Symbol;Acc:HGNC:24596]	protein_coding	3583,032882	0,385068951	0,042286581	1
ENSG00000275945	EIF3FP1	eukaryotic translation initiation factor 3 subunit F pseudogene 1 [Source:HGNC Symbol;Acc:HGNC:3276]	processed_pseudogene	1,45276743	-1,165725519	0,042304479	1
ENSG00000005102	MEOX1	mesenchyme homeobox 1 [Source:HGNC Symbol;Acc:HGNC:7013]	protein_coding	33,14485151	-1,145594891	0,042310989	1
ENSG00000276110			antisense_RNA	2,092670755	-1,162833748	0,042340329	1
ENSG00000254452			antisense_RNA	11,41068831	1,016328777	0,042375099	1
ENSG00000279862			TEC	4,029592131	-1,163909018	0,042384067	1
ENSG00000151208	DLG5	discs large MAGUK scaffold protein 5 [Source:HGNC Symbol;Acc:HGNC:2904]	protein_coding	4004,956735	0,476078394	0,042387012	1
ENSG00000017260	ATP2C1	ATPase secretory pathway Ca2+ transporting 1 [Source:HGNC Symbol;Acc:HGNC:13211]	protein_coding	2009,547424	0,27550364	0,042398446	1
ENSG00000132911	NMUR2	neuromedin U receptor 2 [Source:HGNC Symbol;Acc:HGNC:16454]	protein_coding	2,497349315	1,166688637	0,042413501	1
ENSG00000263165			antisense_RNA	6,886139725	0,934110916	0,042418537	1
ENSG00000129514	FOXA1	forkhead box A1 [Source:HGNC Symbol;Acc:HGNC:5021]	protein_coding	5,759049331	-1,093576129	0,042423881	1
ENSG00000223519	KIF28P	kinesin family member 28, pseudogene [Source:HGNC Symbol;Acc:HGNC:49205]	transcribed_unitary_pseudogene	6,572920168	-1,092248186	0,042427665	1
ENSG00000181652	ATG9B	autophagy related 9B [Source:HGNC Symbol;Acc:HGNC:21899]	protein_coding	22,9129593	-0,713769763	0,042486251	1
ENSG00000263301			antisense_RNA	4,241192023	0,978085939	0,042509984	1
ENSG00000238275	HOMER2P2	homer scaffolding protein 2 pseudogene 2 [Source:HGNC Symbol;Acc:HGNC:20334]	processed_pseudogene	1,342220323	-1,140684443	0,042518484	1
ENSG00000223343			antisense_RNA	23,09648772	0,667074145	0,042545074	1

ENSG00000253974	NRG1-IT1	NRG1 intronic transcript 1 [Source:HGNC Symbol;Acc:HGNC:43633]	sense_intronic	33,76317875	1,017621794	0,042563453	1
ENSG00000137463	MGARP	mitochondria localized glutamic acid rich protein [Source:HGNC Symbol;Acc:HGNC:29969]	protein_coding	19,96720811	1,01873225	0,042583103	1
ENSG00000261798			antisense_RNA	1,27084678	-1,161669427	0,04273826	1
ENSG00000157510	AFAP1L1	actin filament associated protein 1 like 1 [Source:HGNC Symbol;Acc:HGNC:26714]	protein_coding	16,32303102	-0,851656045	0,042779599	1
ENSG00000188060	RAB42	RAB42, member RAS oncogene family [Source:HGNC Symbol;Acc:HGNC:28702]	protein_coding	51,76771057	-0,653012113	0,042885563	1
ENSG00000177000	MTHFR	methylenetetrahydrofolate reductase [Source:HGNC Symbol;Acc:HGNC:7436]	protein_coding	1102,051525	0,250491097	0,042973298	1
ENSG00000166845	C18orf54	chromosome 18 open reading frame 54 [Source:HGNC Symbol;Acc:HGNC:13796]	protein_coding	211,0815703	-0,653357558	0,042976986	1
ENSG00000272512			lincRNA	26,02041939	0,905139466	0,042986899	1
ENSG00000118046	STK11	serine/threonine kinase 11 [Source:HGNC Symbol;Acc:HGNC:11389]	protein_coding	1598,969056	-0,211208943	0,043016932	1
ENSG00000105576	TNPO2	transportin 2 [Source:HGNC Symbol;Acc:HGNC:19998]	protein_coding	2036,7086863078	0,39774206	0,043039343	1
ENSG00000123570	RAB9B	RAB9B, member RAS oncogene family [Source:HGNC Symbol;Acc:HGNC:14090]	protein_coding	622,3118373	0,494378585	0,04304448	1
ENSG00000201535		Y RNA [Source:RFAM;Acc:RF00019]	misc_RNA	12,11357695	0,974734078	0,043072244	1
ENSG00000156466	GDF6	growth differentiation factor 6 [Source:HGNC Symbol;Acc:HGNC:4221]	protein_coding	20,526184	-1,123798986	0,043085902	1
ENSG00000275359		ST7 antisense RNA 1 conserved region 1 [Source:RFAM;Acc:RF02179]	misc_RNA	1,627402985	1,15815398	0,043168443	1
ENSG00000273162			lincRNA	12,53755491	-0,976265223	0,04317289	1
ENSG00000276547	PCDHGB5	protocadherin gamma subfamily B, 5 [Source:HGNC Symbol;Acc:HGNC:8712]	protein_coding	38,16588921	0,976064797	0,043228296	1
ENSG00000202354	RNY3	RNA, Ro-associated Y3 [Source:HGNC Symbol;Acc:HGNC:10243]	misc_RNA	31,57800312	1,016038154	0,043359342	1
ENSG00000162869	PPP1R21	protein phosphatase 1 regulatory subunit 21 [Source:HGNC Symbol;Acc:HGNC:30595]	protein_coding	503,7261958	0,394925897	0,043359854	1
ENSG00000183379	SYNDIG1L	synapse differentiation inducing 1 like [Source:HGNC Symbol;Acc:HGNC:32388]	protein_coding	68,11195487	-1,132342116	0,043417899	1
ENSG00000163982	OTOP1	otopetrin 1 [Source:HGNC Symbol;Acc:HGNC:19656]	protein_coding	1,862898763	-1,159894758	0,04352694	1
ENSG00000275713	HIST1H2BH	histone cluster 1 H2B family member h [Source:HGNC Symbol;Acc:HGNC:4755]	protein_coding	2315,654321	-0,884461779	0,043528801	1
ENSG00000248911			processed_pseudogene	1,712343506	-1,152160533	0,043536526	1
ENSG00000136352	NKX2-1	NK2 homeobox 1 [Source:HGNC Symbol;Acc:HGNC:11825]	protein_coding	157,0392603	1,146477222	0,043547163	1
ENSG00000237424	FOXD2-AS1	FOXD2 adjacent opposite strand RNA 1 [Source:HGNC Symbol;Acc:HGNC:44256]	antisense_RNA	1,938481457	-1,156533666	0,043561195	1
ENSG00000253929	CASC21	cancer susceptibility 21 (non-protein coding) [Source:HGNC Symbol;Acc:HGNC:49836]	lincRNA	1,954079542	1,159808783	0,04356746	1
ENSG00000234171	RNASEH1-AS1	RNASEH1 antisense RNA 1 [Source:HGNC Symbol;Acc:HGNC:49289]	antisense_RNA	239,4541307	-0,4461976	0,043615019	1
ENSG00000279526			TEC	3,451989151	1,100405237	0,043652044	1
ENSG00000222915	RNU6-564P	RNA, U6 small nuclear 564, pseudogene [Source:HGNC Symbol;Acc:HGNC:47527]	snRNA	2,275176761	1,113378353	0,043688563	1
ENSG00000267659	LINC01482	long intergenic non-protein coding RNA 1482 [Source:HGNC Symbol;Acc:HGNC:51128]	lincRNA	5,323838868	-1,162002099	0,04374874	1
ENSG00000223551	TMSB4XP4	thymosin beta 4, X-linked pseudogene 4 [Source:HGNC Symbol;Acc:HGNC:11886]	processed_pseudogene	9,179288177	0,851755486	0,04383514	1
ENSG00000138821	SLC39A8	solute carrier family 39 member 8 [Source:HGNC Symbol;Acc:HGNC:20862]	protein_coding	80,0106723	-0,830073876	0,043860294	1
ENSG00000240622	RPL7P15	ribosomal protein L7 pseudogene 15 [Source:HGNC Symbol;Acc:HGNC:36740]	processed_pseudogene	4,488863253	-1,158696133	0,043871484	1
ENSG00000139168	ZCRB1	zinc finger CCHC-type and RNA binding motif containing 1 [Source:HGNC Symbol;Acc:HGNC:29620]	protein_coding	1592,998595	-0,260340305	0,043902959	1
ENSG00000152464	RPP38	ribonuclease P/MRP subunit p38 [Source:HGNC Symbol;Acc:HGNC:30329]	protein_coding	191,1375445	-0,321870275	0,043942378	1
ENSG00000118729	CASQ2	calsequestrin 2 [Source:HGNC Symbol;Acc:HGNC:1513]	protein_coding	3,905308921	-1,161456982	0,043947585	1
ENSG00000266446			sense_intronic	1,769460133	-1,160479361	0,043981178	1
ENSG00000228802			antisense_RNA	3,753051536	1,156997558	0,044009129	1
ENSG00000267801			antisense_RNA	8,396056977	-1,069508691	0,044032178	1
ENSG00000277233		Metazoan signal recognition particle RNA [Source:RFAM;Acc:RF00017]	misc_RNA	62,94144574	0,696731198	0,044038563	1
ENSG00000133424	LARGE1	LARGE xylosyl- and glucuronyltransferase 1 [Source:HGNC Symbol;Acc:HGNC:6511]	protein_coding	953,9015353	0,671956454	0,044038813	1
ENSG00000231194	FARP1-AS1	FARP1 antisense RNA 1 [Source:HGNC Symbol;Acc:HGNC:40229]	antisense_RNA	2,29665858	1,121472833	0,044055067	1
ENSG00000230342	FANCD2P2	Fanconi anemia complementation group D2 pseudogene 2 [Source:HGNC Symbol;Acc:HGNC:44488]	unprocessed_pseudogene	3,316738191	-1,157472052	0,044058978	1
ENSG00000013293	SLC7A14	solute carrier family 7 member 14 [Source:HGNC Symbol;Acc:HGNC:29326]	protein_coding	193,9719938	1,012118657	0,044114653	1
ENSG00000167747	C19orf48	chromosome 19 open reading frame 48 [Source:HGNC Symbol;Acc:HGNC:29667]	protein_coding	841,0121482	-0,491275231	0,044140864	1
ENSG00000181359	HSP90AA6P		transcribed_processed_pseudogene	3,387471439	-1,155263597	0,044178513	1
ENSG00000153233	PTPRR	protein tyrosine phosphatase, receptor type R [Source:HGNC Symbol;Acc:HGNC:9680]	protein_coding	91,29030603	-0,976901915	0,044206628	1
ENSG00000008323	PLEKHG6	pleckstrin homology and RhoGEF domain containing G6 [Source:HGNC Symbol;Acc:HGNC:25562]	protein_coding	15,39846817	-1,139989477	0,044241308	1
ENSG00000229659	RPL26P6	ribosomal protein L26 pseudogene 6 [Source:HGNC Symbol;Acc:HGNC:34023]	processed_pseudogene	30,74793392	0,835762871	0,04424904	1
ENSG00000214867	SRSF9P1	serine and arginine rich splicing factor 9 pseudogene 1 [Source:HGNC Symbol;Acc:HGNC:10792]	processed_pseudogene	1,79806537	-1,083225346	0,044274705	1
ENSG00000207217	SNORA80D	small nucleolar RNA, H/ACA box 80D [Source:HGNC Symbol;Acc:HGNC:50435]	snoRNA	11,93697667	-0,889785819	0,044393437	1
ENSG00000243316	GUCY2GP	guanylate cyclase 2G, pseudogene [Source:HGNC Symbol;Acc:HGNC:31863]	transcribed_unitary_pseudogene	2,316307611	-1,15262013	0,044403013	1
ENSG00000225533	PAWRP1	pro-apoptotic WT1 regulator pseudogene 1 [Source:HGNC Symbol;Acc:HGNC:45201]	processed_pseudogene	4,261979589	-1,156690132	0,044405922	1
ENSG00000236393			lincRNA	5,089683832	-1,101456345	0,04446978	1
ENSG00000201321	RNA5S9	RNA, 5S ribosomal 9 [Source:HGNC Symbol;Acc:HGNC:34370]	rRNA	1,915813968	-1,107517852	0,0444803	1
ENSG00000254661			lincRNA	2,082651248	-1,153229543	0,044528643	1
ENSG00000115590	IL1R2	interleukin 1 receptor type 2 [Source:HGNC Symbol;Acc:HGNC:5994]	protein_coding	3,214548894	-1,132610731	0,04464877	1
ENSG00000103657	HERC1	HECT and RLD domain containing E3 ubiquitin protein ligase family member 1 [Source:HGNC Symbol;Acc:HGNC:4867]	protein_coding	8413,746845	0,196584675	0,044707655	1
ENSG00000255316	MTND6P25	mitochondrially encoded NADH:ubiquinone oxidoreductase core subunit 6 pseudogene 25 [Source:HGNC Symbol;Acc:I	unprocessed_pseudogene	1,01144492	-1,101758765	0,044716855	1
ENSG00000181790	ADGRB1	adhesion G protein-coupled receptor B1 [Source:HGNC Symbol;Acc:HGNC:943]	protein_coding	639,8593073	0,841532185	0,044738949	1
ENSG00000096433	ITPR3	inositol 1,4,5-trisphosphate receptor type 3 [Source:HGNC Symbol;Acc:HGNC:6182]	protein_coding	117,0715177	-0,645481349	0,044798339	1
ENSG00000156103	MMP16	matrix metallopeptidase 16 [Source:HGNC Symbol;Acc:HGNC:7162]	protein_coding	2541,676919	0,600722189	0,044799798	1
ENSG00000142538	PTH2	parathyroid hormone 2 [Source:HGNC Symbol;Acc:HGNC:30828]	protein_coding	1,65197139	-1,156635154	0,044817226	1
ENSG00000179058	C9orf50	chromosome 9 open reading frame 50 [Source:HGNC Symbol;Acc:HGNC:23677]	protein_coding	6,835799422	-0,905513097	0,044893471	1
ENSG00000105369	CD79A	CD79a molecule [Source:HGNC Symbol;Acc:HGNC:1698]	protein_coding	4,728531988	-0,919842922	0,044968312	1
ENSG00000255487			antisense_RNA	4,211774701	1,003012369	0,044978255	1
ENSG00000187550	SBK2	SH3 domain binding kinase family member 2 [Source:HGNC Symbol;Acc:HGNC:34416]	protein_coding	1,323709259	-1,081348453	0,044991319	1
ENSG00000278862			TEC	4,258239748	-1,154955031	0,04502277	1

ENSG00000206767	RNU6-949P	RNA, U6 small nuclear 949, pseudogene [Source:HGNC Symbol;Acc:HGNC:47912]	snRNA	2,219283488	-1,142581058	0,045155004	1
ENSG00000200486	SNORD115-11	small nucleolar RNA, C/D box 115-11 [Source:HGNC Symbol;Acc:HGNC:33030]	snoRNA	2,167794606	1,145957936	0,045234369	1
ENSG00000259696			processed_pseudogene	1,365507029	-1,142601379	0,045247855	1
ENSG00000237764			lincRNA	11,02046118	0,815132058	0,045261946	1
ENSG00000151963	ZNF37CP	zinc finger protein 37C, pseudogene [Source:HGNC Symbol;Acc:HGNC:29445]	unprocessed_pseudogene	1,15598398	-1,116286264	0,045270287	1
ENSG00000222714	RN7SKP38	RNA, 7SK small nuclear pseudogene 38 [Source:HGNC Symbol;Acc:HGNC:45762]	misc_RNA	3,22358536	-1,032099545	0,045336233	1
ENSG00000101955	SRPX	sushi repeat containing protein, X-linked [Source:HGNC Symbol;Acc:HGNC:11309]	protein_coding	77,14739446	-0,927325436	0,045345345	1
ENSG00000179639	FCER1A	Fc fragment of IgE receptor Ia [Source:HGNC Symbol;Acc:HGNC:3609]	protein_coding	0,963115628	-1,136821979	0,045358971	1
ENSG00000005381	MPO	myeloperoxidase [Source:HGNC Symbol;Acc:HGNC:7218]	protein_coding	3,974195081	-1,149730243	0,045418391	1
ENSG00000177197	PCNPP5	PEST containing nuclear protein pseudogene 5 [Source:HGNC Symbol;Acc:HGNC:41977]	processed_pseudogene	1,553024915	-1,136272444	0,045452674	1
ENSG00000019505	SYT13	synaptotagmin 13 [Source:HGNC Symbol;Acc:HGNC:14962]	protein_coding	1640,856919	0,68228162	0,045454312	1
ENSG00000272037			antisense_RNA	9,711377638	0,969933982	0,045481338	1
ENSG00000257950	P2RX5-TAX1BP3	P2RX5-TAX1BP3 readthrough (NMD candidate) [Source:HGNC Symbol;Acc:HGNC:49191]	protein_coding	15,95408252	0,821484691	0,045499286	1
ENSG00000236165	PRADC1P1	protease associated domain containing 1 pseudogene 1 [Source:HGNC Symbol;Acc:HGNC:44534]	processed_pseudogene	2,291344917	-1,108131315	0,045516953	1
ENSG00000253001	RN7SKP105	RNA, 7SK small nuclear pseudogene 105 [Source:HGNC Symbol;Acc:HGNC:45829]	misc_RNA	1,890836655	1,144406927	0,045605493	1
ENSG00000102195	GPR50	G protein-coupled receptor 50 [Source:HGNC Symbol;Acc:HGNC:4506]	protein_coding	61,06185519	1,135504199	0,045620667	1
ENSG00000096060	FKBP5	FK506 binding protein 5 [Source:HGNC Symbol;Acc:HGNC:3721]	protein_coding	154,0789077	-0,956390374	0,045625963	1
ENSG00000280206			lincRNA	54,79696054	-0,764086748	0,045675328	1
ENSG00000139178	C1RL	complement C1r subcomponent like [Source:HGNC Symbol;Acc:HGNC:21265]	protein_coding	219,7813915	-0,674562204	0,045708705	1
ENSG00000177873	ZNF619	zinc finger protein 619 [Source:HGNC Symbol;Acc:HGNC:26910]	protein_coding	136,2720135	-0,69342461	0,045726402	1
ENSG00000137672	TRPC6	transient receptor potential cation channel subfamily C member 6 [Source:HGNC Symbol;Acc:HGNC:12338]	protein_coding	17,65675571	0,911508009	0,045727665	1
ENSG00000260545			antisense_RNA	10,12852525	1,071858665	0,045741642	1
ENSG00000163283	ALPP	alkaline phosphatase, placental [Source:HGNC Symbol;Acc:HGNC:439]	protein_coding	1,642597139	-1,078037644	0,04575539	1
ENSG00000259281	LINGO1-AS2	LINGO1 antisense RNA 2 [Source:HGNC Symbol;Acc:HGNC:51423]	antisense_RNA	1,494650882	-1,109844056	0,045760762	1
ENSG00000128815	WDFY4	WDFY family member 4 [Source:HGNC Symbol;Acc:HGNC:29323]	protein_coding	9,443670386	-1,127033513	0,045772268	1
ENSG00000007038	PRSS21	protease, serine 21 [Source:HGNC Symbol;Acc:HGNC:9485]	protein_coding	1,464951028	-1,137973729	0,045851927	1
ENSG00000201988		Y RNA [Source:RFAM;Acc:RF00019]	misc_RNA	1,485618393	1,149034934	0,045924012	1
ENSG00000255003	CYCSP28	cytochrome c, somatic pseudogene 28 [Source:HGNC Symbol;Acc:HGNC:24402]	processed_pseudogene	1,39717098	-1,146189665	0,045976382	1
ENSG00000269921			lincRNA	18,58405259	0,697742695	0,046016015	1
ENSG00000165490	DDIAS	DNA damage induced apoptosis suppressor [Source:HGNC Symbol;Acc:HGNC:26351]	protein_coding	213,7635105	-0,630578536	0,046034078	1
ENSG00000151577	DRD3	dopamine receptor D3 [Source:HGNC Symbol;Acc:HGNC:3024]	protein_coding	7,566010557	-1,140051708	0,046065034	1
ENSG00000119862	LGALSL	galectin like [Source:HGNC Symbol;Acc:HGNC:25012]	protein_coding	863,120225	0,389388218	0,04608959	1
ENSG00000259867			antisense_RNA	11,1712154	-1,02614137	0,046142117	1
ENSG00000184515	BEX5	brain expressed X-linked 5 [Source:HGNC Symbol;Acc:HGNC:27990]	protein_coding	313,590752	0,806694589	0,04616186	1
ENSG00000069482	GAL	galanin and GMAP prepropeptide [Source:HGNC Symbol;Acc:HGNC:4114]	protein_coding	4,020357974	-1,141848353	0,046192914	1
ENSG00000166508	MCM7	minichromosome maintenance complex component 7 [Source:HGNC Symbol;Acc:HGNC:6950]	protein_coding	3438,191315	-0,622843615	0,046278251	1
ENSG00000163214	DHX57	DExH-box helicase 57 [Source:HGNC Symbol;Acc:HGNC:20086]	protein_coding	1394,863958	0,199904424	0,046295572	1
ENSG00000269386	RAB11B-AS1	RAB11B antisense RNA 1 [Source:HGNC Symbol;Acc:HGNC:44178]	antisense_RNA	60,3808813	-0,543928678	0,046322812	1
ENSG00000184814	PRR23B	proline rich 23B [Source:HGNC Symbol;Acc:HGNC:33764]	protein_coding	1,157796133	-0,956813654	0,046333087	1
ENSG00000127241	MASP1	mannan binding lectin serine peptidase 1 [Source:HGNC Symbol;Acc:HGNC:6901]	protein_coding	1609,045122	0,934511691	0,046346134	1
ENSG00000242732	RTL5	retrotransposon Gag like 5 [Source:HGNC Symbol;Acc:HGNC:29430]	protein_coding	317,3701921	0,456840898	0,046351306	1
ENSG00000155833	CYLC2	cylicin 2 [Source:HGNC Symbol;Acc:HGNC:2583]	protein_coding	2,510039486	-1,120237279	0,046379671	1
ENSG00000146250	PRSS35	protease, serine 35 [Source:HGNC Symbol;Acc:HGNC:21387]	protein_coding	162,7737572	1,034755917	0,046398568	1
ENSG00000227582	ADGRF5P1	adhesion G protein-coupled receptor F5 pseudogene 1 [Source:HGNC Symbol;Acc:HGNC:32922]	transcribed_processed_pseudogene	4,770534044	-1,147003576	0,046429501	1
ENSG00000163807	KIAA1143	KIAA1143 [Source:HGNC Symbol;Acc:HGNC:29198]	protein_coding	1177,637474	-0,234379039	0,046574293	1
ENSG00000248810	LINC02432	long intergenic non-protein coding RNA 2432 [Source:HGNC Symbol;Acc:HGNC:53363]	lincRNA	1,793131287	-1,135691022	0,046574586	1
ENSG00000275305		5S ribosomal RNA [Source:RFAM;Acc:RF00001]	rRNA	1,773075014	-1,042910903	0,046648232	1
ENSG00000009765	IYD	iodotyrosine deiodinase [Source:HGNC Symbol;Acc:HGNC:21071]	protein_coding	9,119076785	-1,088016564	0,046682365	1
ENSG00000017483	SLC38A5	solute carrier family 38 member 5 [Source:HGNC Symbol;Acc:HGNC:18070]	protein_coding	18,26324482	-0,840556713	0,046703716	1
ENSG00000278588	HIST1H2BI	histone cluster 1 H2B family member i [Source:HGNC Symbol;Acc:HGNC:4756]	protein_coding	1782,379168	-0,912235986	0,046719177	1
ENSG00000080503	SMARCA2	SWI/SNF related, matrix associated, actin dependent regulator of chromatin, subfamily a, member 2 [Source:HGNC Sy	protein_coding	2973,134726	0,644883003	0,046722565	1
ENSG00000008517	IL32	interleukin 32 [Source:HGNC Symbol;Acc:HGNC:16830]	protein_coding	14,88378205	-1,136135774	0,046758231	1
ENSG00000260759			lincRNA	2,21031903	-1,105755295	0,046782465	1
ENSG00000269984			antisense_RNA	16,25448805	0,917532126	0,04693287	1
ENSG00000115648	MLPH	melanophilin [Source:HGNC Symbol;Acc:HGNC:29643]	protein_coding	33,04874651	-1,110912066	0,047022097	1
ENSG00000236031			antisense_RNA	12,10115231	1,093780164	0,047031746	1
ENSG00000227766	HCG4P5		unprocessed_pseudogene	17,40364182	1,125478139	0,047045774	1
ENSG00000130449	ZSWIM6	zinc finger SWIM-type containing 6 [Source:HGNC Symbol;Acc:HGNC:29316]	protein_coding	1509,156025	0,353588679	0,04708881	1
ENSG00000140623	SEPT12	septin 12 [Source:HGNC Symbol;Acc:HGNC:26348]	protein_coding	7,709243859	0,856839419	0,047089136	1
ENSG00000225234	TRAPPC12-AS1	TRAPPC12 antisense RNA 1 [Source:HGNC Symbol;Acc:HGNC:41046]	antisense_RNA	1,083978895	1,106531116	0,047109044	1
ENSG00000204161	C10orf128	chromosome 10 open reading frame 128 [Source:HGNC Symbol;Acc:HGNC:27274]	protein_coding	1,860823902	-1,144446811	0,047117072	1
ENSG00000117133	RPF1	ribosome production factor 1 homolog [Source:HGNC Symbol;Acc:HGNC:30350]	protein_coding	741,9331805	-0,280261579	0,047192596	1
ENSG00000118402	ELOVL4	ELOVL fatty acid elongase 4 [Source:HGNC Symbol;Acc:HGNC:14415]	protein_coding	445,4058001	0,478011489	0,047195525	1
ENSG00000249359			lincRNA	1,641128945	-1,117549288	0,047222644	1
ENSG00000254588			antisense_RNA	2,086228621	-1,143486307	0,04725747	1
ENSG00000260621			antisense_RNA	20,1114816	1,099087319	0,047296724	1
ENSG00000266265	KLF14	Kruppel like factor 14 [Source:HGNC Symbol;Acc:HGNC:23025]	protein_coding	7,051921506	-1,106165741	0,047324814	1

ENSG00000085832	EPS15	epidermal growth factor receptor pathway substrate 15 [Source:HGNC Symbol;Acc:HGNC:3419]	protein_coding	3323,5369083918	0,371238625	0,047362937	1
ENSG00000156564	LRFN2	leucine rich repeat and fibronectin type III domain containing 2 [Source:HGNC Symbol;Acc:HGNC:21226]	protein_coding	98,16019282	0,868525404	0,047385021	1
ENSG00000133275	CSNK1G2	casein kinase 1 gamma 2 [Source:HGNC Symbol;Acc:HGNC:2455]	protein_coding	1948,891663	-0,408342294	0,047477313	1
ENSG00000129204	USP6	ubiquitin specific peptidase 6 [Source:HGNC Symbol;Acc:HGNC:12629]	protein_coding	17,30489563	-1,10736954	0,047604987	1
ENSG00000182885	ADGRG3	adhesion G protein-coupled receptor G3 [Source:HGNC Symbol;Acc:HGNC:13728]	protein_coding	8,592042568	-0,964938104	0,047615299	1
ENSG00000237121	PIEZO1P2	piezo type mechanosensitive ion channel component 1 pseudogene 2 [Source:HGNC Symbol;Acc:HGNC:42861]	unprocessed_pseudogene	3,398855924	-1,133135894	0,047641663	1
ENSG00000107187	LHX3	LIM homeobox 3 [Source:HGNC Symbol;Acc:HGNC:6595]	protein_coding	4,4730017	-1,135279099	0,047643104	1
ENSG00000185479	KRT6B	keratin 6B [Source:HGNC Symbol;Acc:HGNC:6444]	protein_coding	2,231801493	-1,11846714	0,047665919	1
ENSG00000011426	ANLN	anillin actin binding protein [Source:HGNC Symbol;Acc:HGNC:14082]	protein_coding	985,7146024	-0,646920064	0,047714272	1
ENSG00000254109	RBPMS-AS1	RBPMS antisense RNA 1 [Source:HGNC Symbol;Acc:HGNC:48721]	antisense_RNA	2,515371311	-1,085569452	0,047715737	1
ENSG00000161649	CD300LG	CD300 molecule like family member g [Source:HGNC Symbol;Acc:HGNC:30455]	protein_coding	3,037612045	-1,139778825	0,047720586	1
ENSG00000226780			antisense_RNA	2,205959679	-1,141101263	0,047781036	1
ENSG00000227456	LINC00310	long intergenic non-protein coding RNA 310 [Source:HGNC Symbol;Acc:HGNC:16414]	lincRNA	12,3657011	0,886317581	0,047793266	1
ENSG00000187621	TCL6	T-cell leukemia/lymphoma 6 (non-protein coding) [Source:HGNC Symbol;Acc:HGNC:13463]	processed_transcript	5,894121505	-1,12998565	0,047798957	1
ENSG00000207742	MIR487A	microRNA 487a [Source:HGNC Symbol;Acc:HGNC:32343]	miRNA	1,338787537	0,96557717	0,04785268	1
ENSG00000271494			processed_pseudogene	1,243181009	-1,10500838	0,047858791	1
ENSG00000283459			lincRNA	2,087796594	-1,139103086	0,047887744	1
ENSG00000204963	PCDHA7	protocadherin alpha 7 [Source:HGNC Symbol;Acc:HGNC:8673]	protein_coding	79,20912795	0,521983411	0,04790427	1
ENSG00000276135	FAM27E2	family with sequence similarity 27 member E2 [Source:HGNC Symbol;Acc:HGNC:32013]	processed_pseudogene	1,726518202	-1,14039437	0,047907914	1
ENSG00000261532			lincRNA	3,404431025	1,030336517	0,047986725	1
ENSG00000010295	IFFO1	intermediate filament family orphan 1 [Source:HGNC Symbol;Acc:HGNC:24970]	protein_coding	438,8794299	-0,2934336	0,047997058	1
ENSG00000230076	RPL10P6	ribosomal protein L10 pseudogene 6 [Source:HGNC Symbol;Acc:HGNC:52343]	processed_pseudogene	10,66306211	1,016136816	0,048056964	1
ENSG00000143632	ACTA1	actin, alpha 1, skeletal muscle [Source:HGNC Symbol;Acc:HGNC:129]	protein_coding	8,855904824	-0,965062555	0,048120771	1
ENSG00000101654	RNMT	RNA guanine-7 methyltransferase [Source:HGNC Symbol;Acc:HGNC:10075]	protein_coding	1457,02834	0,216086872	0,048121286	1
ENSG00000261329			antisense_RNA	3,563862372	-1,022683122	0,048207904	1
ENSG00000280744		long intergenic non-protein coding RNA 1173 [Source:NCBI gene;Acc:106144537]	lincRNA	3,434431578	-1,103372828	0,048212885	1
ENSG00000245748			antisense_RNA	78,82998076	1,068033442	0,048246557	1
ENSG00000230751			sense_intronic	3,912516501	0,990711755	0,048263092	1
ENSG00000135898	GPR55	G protein-coupled receptor 55 [Source:HGNC Symbol;Acc:HGNC:4511]	protein_coding	7,103805302	-1,137494734	0,048273061	1
ENSG00000259007			antisense_RNA	31,28200022	1,131128519	0,04832115	1
ENSG00000165816	VWA2	von Willebrand factor A domain containing 2 [Source:HGNC Symbol;Acc:HGNC:24709]	protein_coding	3,837833617	-1,137243884	0,048340055	1
ENSG00000213239	NPM1P32	nucleophosmin 1 pseudogene 32 [Source:HGNC Symbol;Acc:HGNC:45211]	processed_pseudogene	1,891083842	-1,132757793	0,048346248	1
ENSG00000184709	LRRC26	leucine rich repeat containing 26 [Source:HGNC Symbol;Acc:HGNC:31409]	protein_coding	6,676899465	-0,932968006	0,048466472	1
ENSG00000181227	DLSTP1	dihydrolipoamide S-succinyltransferase pseudogene 1 [Source:HGNC Symbol;Acc:HGNC:2912]	processed_pseudogene	1,999620124	1,127972317	0,0484679	1
ENSG00000115207	GTF3C2	general transcription factor IIIC subunit 2 [Source:HGNC Symbol;Acc:HGNC:4665]	protein_coding	967,067894	-0,17707001	0,048502881	1
ENSG00000205116	TMEM88B	transmembrane protein 88B [Source:HGNC Symbol;Acc:HGNC:37099]	protein_coding	2,617276642	-1,109426711	0,048513657	1
ENSG00000139350	NEDD1	neural precursor cell expressed, developmentally down-regulated 1 [Source:HGNC Symbol;Acc:HGNC:7723]	protein_coding	1086,501736	-0,492406445	0,048530343	1
ENSG00000250332			processed_pseudogene	1,726704337	-1,133267928	0,048560241	1
ENSG00000229359	PIN1P1	peptidylprolyl cis/trans isomerase, NIMA-interacting 1 pseudogene 1 [Source:HGNC Symbol;Acc:HGNC:8989]	transcribed_processed_pseudogene	7,685727854	0,952098837	0,048587922	1
ENSG00000156509	FBXO43	F-box protein 43 [Source:HGNC Symbol;Acc:HGNC:28521]	protein_coding	13,36341574	-0,812425935	0,048599065	1
ENSG00000231313	CLIC1P1		processed_pseudogene	1,59321761	-1,135589227	0,048723016	1
ENSG00000229315	MCHR2-AS1	MCHR2 antisense RNA 1 [Source:HGNC Symbol;Acc:HGNC:48980]	antisense_RNA	2,134066459	-1,133402289	0,048752837	1
ENSG00000174358	SLC6A19	solute carrier family 6 member 19 [Source:HGNC Symbol;Acc:HGNC:27960]	protein_coding	3,508776869	-1,131523905	0,048819666	1
ENSG00000140968	IRF8	interferon regulatory factor 8 [Source:HGNC Symbol;Acc:HGNC:5358]	protein_coding	5,820883022	-1,086464955	0,048824974	1
ENSG00000105928	GSDME	gasdermin E [Source:HGNC Symbol;Acc:HGNC:2810]	protein_coding	498,6356582	0,438110894	0,048841017	1
ENSG00000048028	USP28	ubiquitin specific peptidase 28 [Source:HGNC Symbol;Acc:HGNC:12625]	protein_coding	717,7031829	-0,316995689	0,048847543	1
ENSG00000021355	SERPINB1	serpin family B member 1 [Source:HGNC Symbol;Acc:HGNC:3311]	protein_coding	17,53570983	-1,083297042	0,048872355	1
ENSG00000112118	MCM3	minichromosome maintenance complex component 3 [Source:HGNC Symbol;Acc:HGNC:6945]	protein_coding	1897,49903	-0,636481158	0,048911442	1
ENSG00000148604	RGR	retinal G protein coupled receptor [Source:HGNC Symbol;Acc:HGNC:9990]	protein_coding	23,61529909	0,815016436	0,048992654	1
ENSG00000172421	EFCAB3	EF-hand calcium binding domain 3 [Source:HGNC Symbol;Acc:HGNC:26379]	protein_coding	1,607428116	-1,134323261	0,049020915	1
ENSG00000159423	ALDH4A1	aldehyde dehydrogenase 4 family member A1 [Source:HGNC Symbol;Acc:HGNC:406]	protein_coding	261,4697282	-0,755623949	0,049040347	1
ENSG00000214491	SEC14L6	SEC14 like lipid binding 6 [Source:HGNC Symbol;Acc:HGNC:40047]	protein_coding	22,71294311	-0,978950281	0,049076703	1
ENSG00000130270	ATP8B3	ATPase phospholipid transporting 8B3 [Source:HGNC Symbol;Acc:HGNC:13535]	protein_coding	25,93818567	-0,861730264	0,049145999	1
ENSG00000226435	ANKRD18DP	ankyrin repeat domain 18D, pseudogene [Source:HGNC Symbol;Acc:HGNC:28016]	transcribed_unprocessed_pseudogene	2,945975414	-1,111031461	0,049197616	1
ENSG00000176563	CNTD1	cyclin N-terminal domain containing 1 [Source:HGNC Symbol;Acc:HGNC:26847]	protein_coding	25,26632573	0,778829967	0,049216323	1
ENSG00000229110			processed_pseudogene	2,363760038	-1,129100817	0,049219052	1
ENSG00000272277			antisense_RNA	37,60705184	-0,543632271	0,049291627	1
ENSG00000130921	C12orf65	chromosome 12 open reading frame 65 [Source:HGNC Symbol;Acc:HGNC:26784]	protein_coding	325,049117	-0,245236984	0,049318073	1
ENSG00000204965	PCDHA5	protocadherin alpha 5 [Source:HGNC Symbol;Acc:HGNC:8671]	protein_coding	123,051487	0,770814849	0,049385556	1
ENSG00000271969			antisense_RNA	1,87598851	1,092602421	0,049392461	1
ENSG00000225774	SIRPAP1	signal regulatory protein alpha pseudogene 1 [Source:HGNC Symbol;Acc:HGNC:9663]	processed_pseudogene	5,120092149	-0,992352258	0,049401357	1
ENSG00000269535			antisense_RNA	1,827097686	1,109340465	0,049457874	1
ENSG00000167723	TRPV3	transient receptor potential cation channel subfamily V member 3 [Source:HGNC Symbol;Acc:HGNC:18084]	protein_coding	35,6574387	-0,798041038	0,049512418	1
ENSG00000276966	HIST1H4E	histone cluster 1 H4 family member e [Source:HGNC Symbol;Acc:HGNC:4790]	protein_coding	4855,684873	-0,533520464	0,049622354	1
ENSG00000228031			lincRNA	13,22520688	0,918342646	0,049632246	1
ENSG00000160113	NR2F6	nuclear receptor subfamily 2 group F member 6 [Source:HGNC Symbol;Acc:HGNC:7977]	protein_coding	546,9157088	-0,564728622	0,049646677	1
ENSG00000171872	KLF17	Kruppel like factor 17 [Source:HGNC Symbol;Acc:HGNC:18830]	protein_coding	5,345405349	-1,112605188	0,049654908	1

ENSG00000260114			sense_intronic	73,40216326	0,545199443	0,049674146	1
ENSG00000250865			lincRNA	2,62192748	-1,118472282	0,049749133	1
ENSG00000272770			antisense_RNA	31,19688855	1,073397777	0,049814554	1
ENSG00000248685	LINC02484	long intergenic non-protein coding RNA 2484 [Source:HGNC Symbol;Acc:HGNC:53459]	lincRNA	2,946648674	-1,07533984	0,04981542	1
ENSG00000147408	CSGALNACT1	chondroitin sulfate N-acetylgalactosaminyltransferase 1 [Source:HGNC Symbol;Acc:HGNC:24290]	protein_coding	254,5665178	0,926361893	0,049843182	1
ENSG00000171403	KRT9	keratin 9 [Source:HGNC Symbol;Acc:HGNC:6447]	protein_coding	3,683432377	-1,128905271	0,04993949	1
ENSG00000259727			lincRNA	2,010543903	-1,118957702	0,049986052	1
ENSG00000232115			processed_pseudogene	1,101408162	-0,9869638	0,050106344	1
ENSG00000186152	LILRP1	leukocyte immunoglobulin-like receptor pseudogene 1 [Source:HGNC Symbol;Acc:HGNC:15496]	unprocessed_pseudogene	2,632527687	-1,125409177	0,050189428	1
ENSG00000230452	LINC01381	long intergenic non-protein coding RNA 1381 [Source:HGNC Symbol;Acc:HGNC:50653]	antisense_RNA	1,979512038	-1,106750757	0,050200419	1
ENSG00000234597			lincRNA	1,283288442	-1,10495258	0,050228537	1
ENSG00000234612	H2AFZP5	H2A histone family member Z pseudogene 5 [Source:HGNC Symbol;Acc:HGNC:38018]	processed_pseudogene	2,222755496	-1,119547101	0,050230069	1
ENSG00000166881	NEMP1	nuclear envelope integral membrane protein 1 [Source:HGNC Symbol;Acc:HGNC:29001]	protein_coding	924,9650966	-0,421790163	0,050322585	1
ENSG00000254384	MTND6P19	mitochondrially encoded NADH:ubiquinone oxidoreductase core subunit 6 pseudogene 19 [Source:HGNC Symbol;Acc:	unprocessed_pseudogene	1,571173431	-1,091670343	0,050422161	1
ENSG00000253187	HOXA10-AS	HOXA10 antisense RNA [Source:HGNC Symbol;Acc:HGNC:40281]	antisense_RNA	1,808071797	-1,05270924	0,05042378	1
ENSG00000261758			antisense_RNA	14,43461711	-0,815200494	0,050442209	1
