# Supplementary material for: Neurobiological roots of psychopathy
Source: Mol Psychiatry. 2019 Aug 27;25(12):3432–41. doi: 10.1038/s41380-019-0488-z (PMC7714686; doi:10.1038/s41380-019-0488-z)
Supplement: Supplementary file 3 — Supplementary Table 3 [file 41380_2019_488_MOESM3_ESM.pdf]

Suppl Table 3_Differentially expressed genes in astrocytes among violent offenders versus healthy controls

Differential gene expression results							
Ensembl ID	HGNC symbol	Gene description	Gene biotype	Average expression	Log2 foldchange	P-value	Adjusted p-value
ENSG00000233913	RPL10P9	ribosomal protein L10 pseudogene	processed_pseudogene	101,8703887	2,372565097	8,21028E-09	0,000176858
ENSG00000210082	MT-RNR2	mitochondrially encoded 16S RNA	Mt_rRNA	26374,37141	2,039017002	4,85863E-07	0,005232991
ENSG00000186765	FSCN2	fascin actin-bundling protein 2, ret	protein_coding	50,01287224	1,023910391	1,21886E-05	0,056612999
ENSG00000215241	LINC02449	long intergenic non-protein coding	lincRNA	28,62369221	-1,068084294	1,32665E-05	0,056612999
ENSG00000103044	HAS3	hyaluronan synthase 3 [Source:HG	protein_coding	138,7924998	1,660543841	1,7041E-05	0,056612999
ENSG00000258590	NBEAP1	neurobeachin pseudogene 1 [Sour	transcribed_unprocessed_pseudogene	6,85069227	-1,754002427	1,7967E-05	0,056612999
ENSG00000198650	TAT	tyrosine aminotransferase [Source	protein_coding	67,76583675	-1,601558119	1,83971E-05	0,056612999
ENSG00000241213	LINC02024	long intergenic non-protein coding	lincRNA	34,93483448	-1,049111858	2,43302E-05	0,065512124
ENSG00000151388	ADAMTS12	ADAM metallopeptidase with thro	protein_coding	1056,393325	-1,150865345	3,47625E-05	0,083202169
ENSG00000110218	PANX1	pannexin 1 [Source:HGNC Symbol;	protein_coding	1273,982888	0,287040069	4,62013E-05	0,099522137
ENSG00000100095	SEZ6L	seizure related 6 homolog like [So	protein_coding	395,3755439	0,913384927	5,67875E-05	0,111205346
ENSG00000121743	GJA3	gap junction protein alpha 3 [Sour	protein_coding	50,55713739	-1,236037645	6,41095E-05	0,115081842
ENSG00000234323	LINC01505	long intergenic non-protein coding	lincRNA	26,20626261	1,618312395	8,6312E-05	0,143018936
ENSG00000188000	OR7D2	olfactory receptor family 7 subfam	protein_coding	7,099228581	-1,518456798	0,000103664	0,157333789
ENSG00000207181	SNORA14B	small nucleolar RNA, H/ACA box 14	snoRNA	118,4172271	0,898759836	0,000109559	0,157333789
ENSG00000115361	ACADL	acyl-CoA dehydrogenase long chai	protein_coding	81,14375991	-1,210810138	0,000120987	0,162886445
ENSG00000156206	CFAP161	cilia and flagella associated protei	protein_coding	68,07331638	-0,91717864	0,000130908	0,165875739
ENSG00000126016	AMOT	angiomotin [Source:HGNC Symbol;	protein_coding	1502,661952	0,868893411	0,000141906	0,16982172
ENSG00000280660			sense_intronic	20,00588364	-1,465332243	0,0002037	0,216050026
ENSG00000207008	SNORA54	small nucleolar RNA, H/ACA box 54	snoRNA	80,26701521	0,821951422	0,000207174	0,216050026
ENSG00000267270	PARD6G-AS1	PARD6G antisense RNA 1 [Source:I	antisense_RNA	140,1874651	-0,83696511	0,000210624	0,216050026
ENSG00000169213	RAB3B	RAB3B, member RAS oncogene far	protein_coding	181,4503494	1,494866244	0,000256164	0,244004202
ENSG00000237732			transcribed_unprocessed_pseudogene	18,82318234	1,509460172	0,000260531	0,244004202
ENSG00000207016	SNORA36C	small nucleolar RNA, H/ACA box 36	snoRNA	5,787161565	1,338181941	0,000338153	0,291453781
ENSG00000171234	UGT2B7	UDP glucuronosyltransferase famil	protein_coding	6,503340494	-1,470011099	0,000338255	0,291453781
ENSG00000263731			lincRNA	378,7011361	0,318558775	0,000359313	0,293344744
ENSG00000169085	C8orf46	chromosome 8 open reading frame	protein_coding	131,9559041	1,154927625	0,000367685	0,293344744
ENSG00000155761	SPAG17	sperm associated antigen 17 [Sour	protein_coding	30,32098924	-1,258559552	0,000398089	0,306258661
ENSG00000189056	RELN	reelin [Source:HGNC Symbol;Acc:H	protein_coding	8,70028	1,448376239	0,000461796	0,343018623
ENSG00000122359	ANXA11	annexin A11 [Source:HGNC Symbo	protein_coding	190,1726994	-0,955944149	0,000604967	0,427993126
ENSG00000152402	GUCY1A2	guanylate cyclase 1 soluble subuni	protein_coding	167,4124593	1,358053993	0,000634316	0,427993126
ENSG00000159023	EPB41	erythrocyte membrane protein ba	protein_coding	439,6267093	0,679106893	0,000635801	0,427993126
ENSG00000237441	RGL2	ral guanine nucleotide dissociation	protein_coding	2007,240597	-0,335009665	0,000665649	0,434507487
ENSG00000169402	RSPH10B2	radial spoke head 10 homolog B2 [	protein_coding	7,395642471	-1,362832624	0,000686126	0,43470104
ENSG00000105877	DNAH11	dynein axonemal heavy chain 11 [S	protein_coding	405,8490376	-1,249121542	0,000718503	0,442208026
ENSG00000132394	EEFSEC	eukaryotic elongation factor, selen	protein_coding	182,8891671	0,473657211	0,000743291	0,444756584
ENSG00000163263	C1orf189	chromosome 1 open reading frame	protein_coding	10,99448172	-1,179129111	0,000787781	0,458637696
ENSG00000233557	NFU1P2	NFU1 iron-sulfur cluster scaffold ps	processed_pseudogene	4,724679129	-1,383134325	0,000824841	0,467576465
ENSG00000207493	SNORA46	small nucleolar RNA, H/ACA box 46	snoRNA	43,16012302	0,88017744	0,000870625	0,472242783
ENSG00000133315	MACROD1	MACRO domain containing 1 [Sour	protein_coding	385,373857	-0,415651126	0,000876919	0,472242783
ENSG00000254048			sense_intronic	9,765810531	-1,128351218	0,000925022	0,480224349

Suppl Table 3_Differentially expressed genes in astrocytes among violent offenders versus healthy controls

ENSG00000132872	SYT4	synaptotagmin 4 [Source:HGNC Sy	protein_coding	101,5907971	1,348758956	0,00093635	0,480224349
ENSG00000047579	DTNBP1	dystrobrevin binding protein 1 [Sou	protein_coding	157,6827113	0,924362615	0,000958621	0,480224349
ENSG00000149294	NCAM1	neural cell adhesion molecule 1 [S	protein_coding	6327,324094	0,512283785	0,001002442	0,490763911
ENSG00000236780	LINC01829	long intergenic non-protein coding	lincRNA	2,73171212	-1,343578798	0,00111333	0,53060674
ENSG00000112667	DNPH1	2'-deoxynucleoside 5'-phosphate N	protein_coding	540,7957359	-0,493450953	0,001133091	0,53060674
ENSG00000113532	ST8SIA4	ST8 alpha-N-acetyl-neuraminide al	protein_coding	3108,019543	-1,139733556	0,001184454	0,542857751
ENSG00000244378	RPS2P45	ribosomal protein S2 pseudogene 4	transcribed_processed_pseudogene	2,956075138	1,335983228	0,001232918	0,544376201
ENSG00000151233	GXYLT1	glucoside xylosyltransferase 1 [Sou	protein_coding	459,9628202	0,390087559	0,00123831	0,544376201
ENSG00000167767	KRT80	keratin 80 [Source:HGNC Symbol;A	protein_coding	11,39953458	1,185823754	0,001316357	0,567112858
ENSG00000198842	DUSP27	dual specificity phosphatase 27, at	protein_coding	13,27261269	1,3210083	0,001370621	0,578912567
ENSG00000102043	MTMR8	myotubularin related protein 8 [So	protein_coding	24,35090076	0,773675663	0,001479078	0,61270823
ENSG00000266439	RN7SL493P	RNA, 7SL, cytoplasmic 493, pseudo	misc_RNA	40,75129798	1,178369939	0,001542986	0,614515517
ENSG00000118322	ATP10B	ATPase phospholipid transporting	protein_coding	837,1714004	1,293526028	0,001591838	0,614515517
ENSG00000111913	RIPOR2	RHO family interacting cell polariza	protein_coding	287,3556822	1,277460928	0,001619669	0,614515517
ENSG00000184486	POU3F2	POU class 3 homeobox 2 [Source:H	protein_coding	2065,550096	0,718912718	0,001624108	0,614515517
ENSG00000158258	CLSTN2	calsyntenin 2 [Source:HGNC Symbo	protein_coding	202,3308469	1,301517296	0,00162608	0,614515517
ENSG00000142700	DMRTA2	DMRT like family A2 [Source:HGNC	protein_coding	19,14816732	1,300979139	0,001669949	0,620213361
ENSG00000177459	ERICH5	glutamate rich 5 [Source:HGNC Syr	protein_coding	6,620149877	-1,187726773	0,001761115	0,64298611
ENSG00000207067	SNORA72	small nucleolar RNA, H/ACA box 72	snoRNA	35,5168994	0,748533638	0,001820847	0,645772632
ENSG00000215187	FAM166B	family with sequence similarity 166	protein_coding	4,443879264	-1,27633469	0,001944208	0,645772632
ENSG00000150873	C2orf50	chromosome 2 open reading frame	protein_coding	232,9048551	-1,066841745	0,001949428	0,645772632
ENSG00000207475	SNORA80E	small nucleolar RNA, H/ACA box 80	snoRNA	86,70826485	0,675845999	0,001963432	0,645772632
ENSG00000169220	RGS14	regulator of G protein signaling 14	protein_coding	213,2206971	-0,933336307	0,001965298	0,645772632
ENSG00000259905	PWRN1	Prader-Willi region non-protein co	lincRNA	39,30692376	-1,267803983	0,001971384	0,645772632
ENSG00000021300	PLEKHB1	pleckstrin homology domain conta	protein_coding	1017,40795	-0,666600195	0,001978599	0,645772632
ENSG00000172799	ZBTB8OSP2	zinc finger and BTB domain contair	processed_pseudogene	6,438650982	-1,140292285	0,002057868	0,651904891
ENSG00000160972	PPP1R16A	protein phosphatase 1 regulatory s	protein_coding	1434,289416	-0,398717144	0,002057914	0,651904891
ENSG00000206917	RNU1-52P	RNA, U1 small nuclear 52, pseudog	snRNA	3,675850275	1,245799746	0,002132682	0,665798741
ENSG00000006282	SPATA20	spermatogenesis associated 20 [So	protein_coding	1028,569292	-0,611454541	0,002242498	0,690080832
ENSG00000101049	SGK2	SGK2, serine/threonine kinase 2 [S	protein_coding	13,91984063	-1,01508977	0,002515431	0,757626962
ENSG00000210049	MT-TF	mitochondrially encoded tRNA phe	Mt_tRNA	14,6239912	1,044314788	0,00255611	0,757626962
ENSG00000159625	DRC7	dynein regulatory complex subunit	protein_coding	233,1063617	-1,084404381	0,002567512	0,757626962
ENSG00000232063			lincRNA	5,587366321	-1,064809702	0,002603056	0,757735527
ENSG00000107968	MAP3K8	mitogen-activated protein kinase k	protein_coding	577,1514391	-0,578896271	0,002712792	0,775306786
ENSG00000231721	LINC-PINT	long intergenic non-protein coding	antisense_RNA	235,752068	-0,473403639	0,002735574	0,775306786
ENSG00000129757	CDKN1C	cyclin dependent kinase inhibitor 1	protein_coding	138,7488486	1,055478721	0,002771395	0,775306786
ENSG00000210112	MT-TM	mitochondrially encoded tRNA me	Mt_tRNA	14,65267356	1,077595754	0,002867816	0,791995209
ENSG00000108231	LGI1	leucine rich glioma inactivated 1 [S	protein_coding	138,9732335	-1,156574728	0,002977668	0,800277734
ENSG00000133101	CCNA1	cyclin A1 [Source:HGNC Symbol;Ac	protein_coding	48,68391784	-1,125327216	0,002994521	0,800277734
ENSG00000140941	MAP1LC3B	microtubule associated protein 1 li	protein_coding	1595,66469	-0,411902339	0,003009261	0,800277734
ENSG00000174844	DNAH12	dynein axonemal heavy chain 12 [S	protein_coding	63,89430463	-1,179997397	0,003063099	0,804661206
ENSG00000132383	RPA1	replication protein A1 [Source:HGN	protein_coding	768,373076	0,479173044	0,003142047	0,81534257
ENSG00000282961	PRNCR1	prostate cancer associated non-co	lincRNA	4,432500106	1,203179434	0,003179461	0,81534257
ENSG00000142798	HSPG2	heparan sulfate proteoglycan 2 [Sc	protein_coding	1024,777332	1,040562778	0,003234354	0,81966134

Suppl Table 3_Differentially expressed genes in astrocytes among violent offenders versus healthy controls

ENSG00000234275			lincRNA	3,50592547	1,207857651	0,003392877	0,846054737
ENSG00000180440	SERTM1	serine rich and transmembrane do	protein_coding	44,76574024	-1,205106853	0,003417054	0,846054737
ENSG00000077327	SPAG6	sperm associated antigen 6 [Sourc	protein_coding	3,010593818	-1,199656307	0,003607317	0,863216032
ENSG00000272217			lincRNA	2,73442216	-1,187997106	0,003608853	0,863216032
ENSG00000123119	NECAB1	N-terminal EF-hand calcium bindin	protein_coding	133,166945	1,153364517	0,003646251	0,863216032
ENSG00000251664	PCDHA12	protocadherin alpha 12 [Source:HG	protein_coding	253,0762908	-0,610887016	0,003646658	0,863216032
ENSG00000160401	CFAP157	cilia and flagella associated protein	protein_coding	17,3214461	-1,171021919	0,003752334	0,872388008
ENSG00000185664	PMEL	premelanosome protein [Source:H	protein_coding	18,70090001	1,185123107	0,00380422	0,872388008
ENSG00000234665			lincRNA	4,566630886	-1,156107118	0,003807881	0,872388008
ENSG00000185055	EFCAB10	EF-hand calcium binding domain 1	protein_coding	60,90746879	-1,06530419	0,003877564	0,872388008
ENSG00000273557			sense_intronic	8,359730888	-1,132459617	0,0038879	0,872388008
ENSG00000206805		Y RNA [Source:RFAM;Acc:RF00019	misc_RNA	21,08115427	0,894289432	0,003976541	0,883079008
ENSG00000147403	RPL10	ribosomal protein L10 [Source:HGN	protein_coding	4185,313842	-0,497699316	0,004074295	0,895554919
ENSG00000212283	SNORD89	small nucleolar RNA, C/D box 89 [S	snoRNA	470,6010071	0,719536063	0,004152491	0,903523411
ENSG00000243629	LINC00880	long intergenic non-protein coding	lincRNA	88,04562317	-1,183414571	0,004245116	0,913216637
ENSG00000229178			lincRNA	7,671002109	1,045639216	0,004283616	0,913216637
ENSG00000071205	ARHGAP10	Rho GTPase activating protein 10 [	protein_coding	108,2745079	1,178185694	0,004324223	0,913216637
ENSG00000186676	EEF1GP1	eukaryotic translation elongation f	processed_pseudogene	28,33038079	-1,163224171	0,004407572	0,921781735
ENSG00000180221	TPT1P10	tumor protein, translationally-cont	processed_pseudogene	2,740021856	-1,152882924	0,004575456	0,947691237
ENSG00000086570	FAT2	FAT atypical cadherin 2 [Source:HG	protein_coding	16,75435332	1,165446612	0,004660085	0,952758446
ENSG00000104435	STMN2	stathmin 2 [Source:HGNC Symbol;	protein_coding	201,8384398	1,119028177	0,004748325	0,952758446
ENSG00000212135	SNORD67	small nucleolar RNA, C/D box 67 [S	snoRNA	290,9174403	0,663245642	0,00483985	0,952758446
ENSG00000131951	LRRC9	leucine rich repeat containing 9 [S	protein_coding	201,6206397	-1,081707414	0,004907569	0,952758446
ENSG00000214391	TUBAP2	tubulin alpha pseudogene 2 [Sourc	processed_pseudogene	30,81389409	0,819941604	0,004919792	0,952758446
ENSG00000275673			sense_intronic	12,50970973	-0,888124295	0,004945558	0,952758446
ENSG00000185742	C11orf87	chromosome 11 open reading fran	protein_coding	793,1936376	-0,808078467	0,004955647	0,952758446
ENSG00000134007	ADAM20	ADAM metallopeptidase domain 2	protein_coding	13,30906077	-0,871230064	0,005058738	0,952758446
ENSG00000039139	DNAH5	dynein axonemal heavy chain 5 [S	protein_coding	584,2563763	-0,844828403	0,005061694	0,952758446
ENSG00000181481	RNF135	ring finger protein 135 [Source:HG	protein_coding	31,98843882	-0,999233477	0,005070895	0,952758446
ENSG00000226800	CACTIN-AS1	CACTIN antisense RNA 1 [Source:H	antisense_RNA	4,139588652	1,154641107	0,005094984	0,952758446
ENSG00000152760	TCTEX1D1	Tctex1 domain containing 1 [Sourc	protein_coding	188,3990167	-1,153504095	0,005170077	0,952758446
ENSG00000204186	ZDBF2	zinc finger DBF-type containing 2 [	protein_coding	882,2266942	-0,82548854	0,00517491	0,952758446
ENSG00000166535	A2ML1	alpha-2-macroglobulin like 1 [Sour	protein_coding	83,32483114	-1,01655706	0,005269423	0,961937658
ENSG00000234171	RNASEH1-AS1	RNASEH1 antisense RNA 1 [Source	antisense_RNA	312,650677	-0,506142225	0,005371539	0,972338804
ENSG00000090975	PITPNM2	phosphatidylinositol transfer prote	protein_coding	471,8412375	-0,664523392	0,005507393	0,981509269
ENSG00000206732	RNU6-936P	RNA, U6 small nuclear 936, pseudo	snRNA	6,139849507	-0,984233353	0,005518652	0,981509269
ENSG00000172346	CSDC2	cold shock domain containing C2 [	protein_coding	74,51952343	1,076679934	0,00560105	0,981509269
ENSG00000227110	LMCD1-AS1	LMCD1 antisense RNA 1 (head to h	antisense_RNA	68,07299611	0,823281635	0,005633341	0,981509269
ENSG00000182551	ADI1	acireductone dioxygenase 1 [Sourc	protein_coding	962,2806997	0,782107625	0,005672069	0,981509269
ENSG00000171766	GATM	glycine amidinotransferase [Source	protein_coding	9543,333918	-0,93336025	0,005695588	0,981509269
ENSG00000229848			antisense_RNA	17,60446376	0,786487468	0,005748591	0,982152799
ENSG00000175104	TRAF6	TNF receptor associated factor 6 [S	protein_coding	1147,812556	-0,25977909	0,005790511	0,982152799
ENSG00000127990	SGCE	sarcoglycan epsilon [Source:HGNC	protein_coding	1304,945724	-0,595393309	0,005916565	0,992836781
ENSG00000273783			antisense_RNA	30,69466783	0,560438987	0,005945682	0,992836781

Suppl Table 3_Differentially expressed genes in astrocytes among violent offenders versus healthy controls

ENSG00000267034			lincRNA	28,27598787	1,101984435	0,005996807	0,993670872
ENSG00000171617	ENC1	ectodermal-neural cortex 1 [Source	protein_coding	13527,75832	0,862393634	0,006161783	0,999657601
ENSG00000238835	SCARNA18	small Cajal body-specific RNA 18 [S	snoRNA	32,42935882	0,628799887	0,006291152	0,999657601
ENSG00000160188	RSPH1	radial spoke head 1 homolog [Sour	protein_coding	165,4033279	-0,9502281	0,006319846	0,999657601
ENSG00000173281	PPP1R3B	protein phosphatase 1 regulatory s	protein_coding	423,5683353	-0,420711089	0,00634149	0,999657601
ENSG00000146859	TMEM140	transmembrane protein 140 [Sour	protein_coding	2,735090772	1,108905669	0,006368847	0,999657601
ENSG00000143494	VASH2	vasohibin 2 [Source:HGNC Symbol;	protein_coding	92,41683681	0,765152836	0,006393636	0,999657601
ENSG00000123360	PDE1B	phosphodiesterase 1B [Source:HGI	protein_coding	8,925762001	1,069319284	0,006513573	0,999657601
ENSG00000004838	ZMYND10	zinc finger MYND-type containing 1	protein_coding	1015,063821	-0,912098065	0,006627681	0,999657601
ENSG00000168010	ATG16L2	autophagy related 16 like 2 [Source	protein_coding	285,2425978	-0,507052895	0,006651681	0,999657601
ENSG00000166596	CFAP52	cilia and flagella associated protein	protein_coding	301,1679072	-1,052381343	0,006664279	0,999657601
ENSG00000263316			antisense_RNA	3,114777865	-1,115741049	0,006679535	0,999657601
ENSG00000169885	CALML6	calmodulin like 6 [Source:HGNC Sy	protein_coding	9,523118284	-1,054693114	0,00668643	0,999657601
ENSG00000025708	TYMP	thymidine phosphorylase [Source:I	protein_coding	19,4326857	-0,85096791	0,00676712	0,999657601
ENSG00000234161	PABPC5-AS1	PABPC5 antisense RNA 1 [Source:H	antisense_RNA	17,91939668	0,865089698	0,006930344	0,999657601
ENSG00000249158	PCDHA11	protocadherin alpha 11 [Source:HC	protein_coding	214,5392231	1,03905577	0,007219159	0,999657601
ENSG00000253333			unprocessed_pseudogene	3,824209231	1,092297391	0,007310927	0,999657601
ENSG00000227145	IL21-AS1	IL21 antisense RNA 1 [Source:HGN	antisense_RNA	10,55076562	-1,039727719	0,007398038	0,999657601
ENSG00000145491	ROPN1L	rhophilin associated tail protein 1 l	protein_coding	36,35540676	-0,983443662	0,007414592	0,999657601
ENSG00000154099	DNAAF1	dynein axonemal assembly factor 1	protein_coding	67,93036052	-0,996760581	0,007484662	0,999657601
ENSG00000274020	LINC01138	long intergenic non-protein coding	processed_transcript	121,2206182	-0,895356419	0,007489027	0,999657601
ENSG00000165309	ARMC3	armadillo repeat containing 3 [Sou	protein_coding	141,7756156	-1,096940247	0,007491513	0,999657601
ENSG00000224525			antisense_RNA	7,832660442	-1,100748757	0,007536891	0,999657601
ENSG00000196071	OR2L13	olfactory receptor family 2 subfam	protein_coding	6,431386943	-1,056072213	0,007538106	0,999657601
ENSG00000207292		Y RNA [Source:RFAM;Acc:RF00019	misc_RNA	12,09918409	1,04725443	0,007643839	0,999657601
ENSG00000158623	COPG2	coatomer protein complex subunit	protein_coding	1149,36144	0,284228067	0,007646482	0,999657601
ENSG00000171735	CAMTA1	calmodulin binding transcription ac	protein_coding	1073,028612	0,351796096	0,007697636	0,999657601
ENSG00000270765	GAS2L2	growth arrest specific 2 like 2 [Sou	protein_coding	19,50242837	-1,098385311	0,007748633	0,999657601
ENSG00000262890			lincRNA	21,34441328	-1,062444184	0,007779281	0,999657601
ENSG00000277135			lincRNA	4,603891969	1,097241584	0,007816839	0,999657601
ENSG00000171714	ANO5	anoctamin 5 [Source:HGNC Symbo	protein_coding	144,8691543	-1,09635389	0,00784224	0,999657601
ENSG00000136918	WDR38	WD repeat domain 38 [Source:HGI	protein_coding	88,53509758	-1,027182592	0,007888576	0,999657601
ENSG00000109107	ALDOC	aldolase, fructose-bisphosphate C	protein_coding	534,4844665	0,693615934	0,007905871	0,999657601
ENSG00000163885	CFAP100	cilia and flagella associated protein	protein_coding	80,32045965	-1,074718171	0,007921049	0,999657601
ENSG00000243696			protein_coding	15,5291921	-0,937142785	0,007960792	0,999657601
ENSG00000102445	RUBCNL	RUN and cysteine rich domain cont	protein_coding	27,6617923	1,075940131	0,007990921	0,999657601
ENSG00000182871	COL18A1	collagen type XVIII alpha 1 chain [S	protein_coding	306,9184364	1,094374013	0,008057421	0,999657601
ENSG00000207233	SNORA37	small nucleolar RNA, H/ACA box 37	snoRNA	55,45010433	0,71993635	0,008139854	0,999657601
ENSG00000182568	SATB1	SATB homeobox 1 [Source:HGNC S	protein_coding	1148,74373	0,348464941	0,008151919	0,999657601
ENSG00000075035	WSCD2	WSC domain containing 2 [Source:	protein_coding	653,9710114	0,999785742	0,008229017	0,999657601
ENSG00000165973	NELL1	neural EGFL like 1 [Source:HGNC S	protein_coding	2,68280634	1,041717288	0,008281355	0,999657601
ENSG00000165164	CFAP47	cilia and flagella associated protein	protein_coding	14,42325117	-1,058149237	0,008319413	0,999657601
ENSG00000185610	DBX2	developing brain homeobox 2 [Sou	protein_coding	260,4985085	-0,984937621	0,008333864	0,999657601
ENSG00000152582	SPEF2	sperm flagellar 2 [Source:HGNC Sy	protein_coding	403,804604	-0,888861397	0,00837143	0,999657601

Suppl Table 3_Differentially expressed genes in astrocytes among violent offenders versus healthy controls

ENSG00000089280	FUS	FUS RNA binding protein [Source:H	protein_coding	987,6213027	0,867236967	0,008402414	0,999657601
ENSG00000113327	GABRG2	gamma-aminobutyric acid type A r	protein_coding	3,326838397	1,020229878	0,008438025	0,999657601
ENSG00000260593			antisense_RNA	6,981660059	-1,042052563	0,008637664	0,999657601
ENSG00000121691	CAT	catalase [Source:HGNC Symbol;Acc	protein_coding	2236,525301	-0,467134891	0,008669183	0,999657601
ENSG00000172260	NEGR1	neuronal growth regulator 1 [Sour	protein_coding	1351,625876	-0,853990751	0,008680573	0,999657601
ENSG00000153347	FAM81B	family with sequence similarity 81	protein_coding	98,72143907	-1,056307019	0,008723468	0,999657601
ENSG00000197057	DTHD1	death domain containing 1 [Source	protein_coding	119,9944251	-1,07667029	0,008746843	0,999657601
ENSG00000183248	PRR36	proline rich 36 [Source:HGNC Syml	protein_coding	45,21079125	1,052503988	0,008783724	0,999657601
ENSG00000143811	PYCR2	pyrroline-5-carboxylate reductase	protein_coding	637,8943507	-0,391479685	0,008809757	0,999657601
ENSG00000205476	CCDC85C	coiled-coil domain containing 85C	protein_coding	861,399676	0,366586952	0,008814304	0,999657601
ENSG00000167858	TEKT1	tektin 1 [Source:HGNC Symbol;Acc	protein_coding	217,3956362	-1,076221122	0,008829001	0,999657601
ENSG00000242251	RN7SL20P	RNA, 7SL, cytoplasmic 20, pseudog	misc_RNA	6,799455527	0,96779168	0,008855483	0,999657601
ENSG00000272986			antisense_RNA	7,231713278	-0,915851422	0,008878423	0,999657601
ENSG00000175048	ZDHHC14	zinc finger DHHC-type containing 1	protein_coding	337,3468734	-0,569588372	0,008925455	0,999657601
ENSG00000240063			antisense_RNA	2,739778347	-0,99367315	0,008954452	0,999657601
ENSG00000072778	ACADVL	acyl-CoA dehydrogenase very long	protein_coding	7220,456652	-0,351630947	0,009010951	0,999657601
ENSG00000123080	CDKN2C	cyclin dependent kinase inhibitor 2	protein_coding	585,5499695	0,486654503	0,009031494	0,999657601
ENSG00000074706	IPCEF1	interaction protein for cytohesin e:	protein_coding	16,15581202	1,000796443	0,009043823	0,999657601
ENSG00000203772	SPRN	shadow of prion protein [Source:H	protein_coding	446,0513047	-0,556753724	0,009053505	0,999657601
ENSG00000280269			TEC	6,53736894	-0,928117774	0,009055257	0,999657601
ENSG00000133789	SWAP70	switching B-cell complex subunit S'	protein_coding	1811,031901	-0,385097474	0,009116418	0,999657601
ENSG00000123977	DAW1	dynein assembly factor with WD re	protein_coding	177,073624	-1,059417706	0,009134219	0,999657601
ENSG00000235618	FAM21EP	family with sequence similarity 21	transcribed_unprocessed_pseudogene	19,38347736	0,740716587	0,009141334	0,999657601
ENSG00000189283	FHIT	fragile histidine triad [Source:HGN(	protein_coding	45,64143733	-0,592913375	0,009201876	0,999657601
ENSG00000198400	NTRK1	neurotrophic receptor tyrosine kin	protein_coding	9,461585383	0,87485926	0,00925435	0,999657601
ENSG00000243244	STON1	stonin 1 [Source:HGNC Symbol;Acc	protein_coding	644,38741	-0,638571898	0,009306923	0,999657601
ENSG00000162643	WDR63	WD repeat domain 63 [Source:HGI	protein_coding	120,817087	-0,91918472	0,009335377	0,999657601
ENSG00000235590	GNAS-AS1	GNAS antisense RNA 1 [Source:HG	antisense_RNA	19,10155372	0,969032028	0,009351862	0,999657601
ENSG00000264204	AGAP7P	ArfGAP with GTPase domain, anky	unprocessed_pseudogene	11,11943134	-0,89078132	0,009394503	0,999657601
ENSG00000167377	ZNF23	zinc finger protein 23 [Source:HGN	protein_coding	131,1719446	-0,372322122	0,009510142	0,999657601
ENSG00000001460	STPG1	sperm tail PG-rich repeat containir	protein_coding	205,689008	-0,881213278	0,009566697	0,999657601
ENSG00000101457	DNTTIP1	deoxynucleotidyltransferase termi	protein_coding	309,9444969	0,766521401	0,009627135	0,999657601
ENSG00000118004	COLEC11	collectin subfamily member 11 [So	protein_coding	2,637201865	-1,050703702	0,009636323	0,999657601
ENSG00000077942	FBLN1	fibulin 1 [Source:HGNC Symbol;Acc	protein_coding	409,9374133	1,029674603	0,009667929	0,999657601
ENSG00000141622	RNF165	ring finger protein 165 [Source:HG	protein_coding	172,4356584	0,608239779	0,009702033	0,999657601
ENSG00000163075	CFAP221	cilia and flagella associated proteir	protein_coding	153,0519981	-0,874857605	0,009713044	0,999657601
ENSG00000224367	OACYLP	O-acyltransferase like, pseudogene	transcribed_unitary_pseudogene	2,904297421	-1,058099412	0,009834425	0,999657601
ENSG00000197168	NEK5	NIMA related kinase 5 [Source:HGI	protein_coding	164,5482586	-0,954283511	0,009836999	0,999657601
ENSG00000175267	VWA3A	von Willebrand factor A domain co	protein_coding	407,9348276	-0,951440539	0,009962176	0,999657601
ENSG00000171843	MLLT3	MLLT3, super elongation complex :	protein_coding	832,5944082	0,60424681	0,00997342	0,999657601
ENSG00000243710	CFAP57	cilia and flagella associated proteir	protein_coding	85,38556797	-0,889925325	0,010038635	0,999657601
ENSG00000086730	LAT2	linker for activation of T-cells famil	protein_coding	8,412890207	-0,975430799	0,010102161	0,999657601
ENSG00000158113	LRRC43	leucine rich repeat containing 43 [!	protein_coding	59,40185265	-0,713908347	0,010114124	0,999657601
ENSG00000087191	PSMC5	proteasome 26S subunit, ATPase 5	protein_coding	714,9319391	0,440470612	0,010148519	0,999657601

Suppl Table 3_Differentially expressed genes in astrocytes among violent offenders versus healthy controls

ENSG00000034239	EFCAB1	EF-hand calcium binding domain 1	protein_coding	170,7728487	-1,034552883	0,010203903	0,999657601
ENSG00000198000	NOL8	nucleolar protein 8 [Source:HGNC	protein_coding	919,7638185	0,241264709	0,010243684	0,999657601
ENSG00000117245	KIF17	kinesin family member 17 [Source:	protein_coding	92,44011433	-0,856384902	0,010249402	0,999657601
ENSG00000182667	NTM	neurotrimin [Source:HGNC Symbol	protein_coding	485,2179515	0,934505677	0,010261764	0,999657601
ENSG00000267191			antisense_RNA	8,651371871	0,920236137	0,010387952	0,999657601
ENSG00000181378	CFAP65	cilia and flagella associated protein	protein_coding	53,67151895	-0,904984843	0,010453932	0,999657601
ENSG00000225091	SNORA71A	small nucleolar RNA, H/ACA box 7	snoRNA	22,47354206	0,808615606	0,010493931	0,999657601
ENSG00000102871	TRADD	TNFRSF1A associated via death do	protein_coding	200,9532355	-0,331389018	0,010503559	0,999657601
ENSG00000242547	RN7SL169P	RNA, 7SL, cytoplasmic 169, pseudo	misc_RNA	3,989787296	1,022324131	0,010637992	0,999657601
ENSG00000198865	CCDC152	coiled-coil domain containing 152	protein_coding	29,08734048	0,919413828	0,010672765	0,999657601
ENSG00000254067			transcribed_unprocessed_pseudogene	2,652441375	-1,041899617	0,010715211	0,999657601
ENSG00000174130	TLR6	toll like receptor 6 [Source:HGNC S	protein_coding	7,866370156	-0,996222637	0,010854853	0,999657601
ENSG00000178235	SLITRK1	SLIT and NTRK like family member	protein_coding	7,389778165	0,993919309	0,01086412	0,999657601
ENSG00000197748	CFAP43	cilia and flagella associated protein	protein_coding	388,8875879	-0,937590668	0,010890597	0,999657601
ENSG00000156564	LRFN2	leucine rich repeat and fibronectin	protein_coding	17,76889028	1,044294528	0,010928523	0,999657601
ENSG00000162545	CAMK2N1	calcium/calmodulin dependent pro	protein_coding	442,5793673	0,76670669	0,010971646	0,999657601
ENSG00000148180	GSN	gelsolin [Source:HGNC Symbol;Acc	protein_coding	1996,251537	0,889096953	0,011026582	0,999657601
ENSG00000278673			pseudogene	4,761356457	-1,046397339	0,011246017	0,999657601
ENSG00000276141	WHAMMP3	WAS protein homolog associated w	transcribed_unprocessed_pseudogene	34,15444402	-1,012880875	0,011267563	0,999657601
ENSG00000265417			processed_pseudogene	4,913708956	-1,006098341	0,011292733	0,999657601
ENSG00000157087	ATP2B2	ATPase plasma membrane Ca2+ tr	protein_coding	309,053458	0,722737823	0,011296734	0,999657601
ENSG00000135951	TSGA10	testis specific 10 [Source:HGNC Sy	protein_coding	208,8372655	-0,52721653	0,011354673	0,999657601
ENSG00000264229	RNU4ATAC	RNA, U4atac small nuclear (U12-de	snRNA	441,0265556	0,594813503	0,011355386	0,999657601
ENSG00000232411			lincRNA	8,276369991	-0,900011501	0,011411284	0,999657601
ENSG00000213305	HNRNPCP6	heterogeneous nuclear ribonucleo	processed_pseudogene	18,44537204	-0,792018354	0,011547389	0,999657601
ENSG00000279583			TEC	6,654713383	0,920450534	0,011661221	0,999657601
ENSG00000198822	GRM3	glutamate metabotropic receptor 3	protein_coding	55,97742427	1,039063485	0,011718552	0,999657601
ENSG00000157404	KIT	KIT proto-oncogene receptor tyros	protein_coding	135,0140531	1,015587336	0,011726606	0,999657601
ENSG00000110723	EXPH5	exophilin 5 [Source:HGNC Symbol;	protein_coding	185,066917	-0,99276167	0,011761494	0,999657601
ENSG00000206113	CFAP99	cilia and flagella associated protein	protein_coding	68,40445815	-1,006071599	0,011768255	0,999657601
ENSG00000278910	BANCR	BRAF-activated non-protein coding	lincRNA	33,96713805	0,86016574	0,011773627	0,999657601
ENSG00000140807	NKD1	naked cuticle homolog 1 [Source:H	protein_coding	1951,131063	0,876245882	0,011994595	0,999657601
ENSG00000179455	MKRN3	makorin ring finger protein 3 [Sour	protein_coding	308,9620901	-0,548669019	0,011998615	0,999657601
ENSG00000128052	KDR	kinase insert domain receptor [Sou	protein_coding	20,76937695	1,020532925	0,012307093	0,999657601
ENSG00000155970	MICU3	mitochondrial calcium uptake fami	protein_coding	335,6351228	-0,36254557	0,012316974	0,999657601
ENSG00000211448	DIO2	iodothyronine deiodinase 2 [Sourc	protein_coding	118,4021697	1,031659503	0,012329252	0,999657601
ENSG00000087495	PHACTR3	phosphatase and actin regulator 3	protein_coding	44,59064679	1,031672173	0,012480843	0,999657601
ENSG00000130653	PNPLA7	patatin like phospholipase domain	protein_coding	33,48881761	-0,7057841	0,012502609	0,999657601
ENSG00000178031	ADAMTSL1	ADAMTS like 1 [Source:HGNC Sym	protein_coding	318,6229904	1,030347506	0,012512761	0,999657601
ENSG00000167676	PLIN4	perilipin 4 [Source:HGNC Symbol;A	protein_coding	14,29757344	1,032755275	0,012523736	0,999657601
ENSG00000201785	SNORD117	small nucleolar RNA, C/D box 117		snoRNA	11,5174622	0,796281748	0,012666584	0,999657601
ENSG00000236297			transcribed_processed_pseudogene	4,634169118	-0,961079971	0,0126973	0,999657601
ENSG00000201592		Small nucleolar RNA U2-19 [Source	snoRNA	44,85810059	0,597206998	0,012709107	0,999657601
ENSG00000263878	DLGAP1-AS4	DLGAP1 antisense RNA 4 [Source:H	antisense_RNA	3,191500788	-1,013746999	0,012723784	0,999657601

Suppl Table 3_Differentially expressed genes in astrocytes among violent offenders versus healthy controls

ENSG00000171757	LRRC34	leucine rich repeat containing 34 [S	protein_coding	289,1203438	-0,989098242	0,012754054	0,999657601
ENSG00000236816	ANKRD20A7P	ankyrin repeat domain 20 family m	unprocessed_pseudogene	3,717603004	-1,01852578	0,012826982	0,999657601
ENSG00000198718	TOGARAM1	TOG array regulator of axonemal n	protein_coding	1718,965839	-0,249033122	0,012867489	0,999657601
ENSG00000218823	PAPOLB	poly(A) polymerase beta [Source:H	protein_coding	6,294795582	-0,998103272	0,012907646	0,999657601
ENSG00000103550	KNOP1	lysine rich nucleolar protein 1 [Sou	protein_coding	1594,467754	0,24865129	0,012934162	0,999657601
ENSG00000204172	AGAP9	ArfGAP with GTPase domain, anky	protein_coding	53,30528107	-0,595755153	0,012951473	0,999657601
ENSG00000140527	WDR93	WD repeat domain 93 [Source:HGI	protein_coding	47,99101354	-0,88705133	0,01298981	0,999657601
ENSG00000199667		Y RNA [Source:RFAM;Acc:RF00019	misc_RNA	18,83583561	0,793174226	0,013043599	0,999657601
ENSG00000162753	SLC9C2	solute carrier family 9 member C2	protein_coding	93,66014824	-0,670555637	0,013100948	0,999657601
ENSG00000105968	H2AFV	H2A histone family member V [Sou	protein_coding	3711,338912	0,17472058	0,013339683	0,999657601
ENSG00000137819	PAQR5	progestin and adipoQ receptor fam	protein_coding	8,854553799	1,015762057	0,013399348	0,999657601
ENSG00000163482	STK36	serine/threonine kinase 36 [Source	protein_coding	2613,231692	-0,306552475	0,013433548	0,999657601
ENSG00000110002	VWA5A	von Willebrand factor A domain co	protein_coding	1127,7455	-0,406675641	0,013464632	0,999657601
ENSG00000057468	MSH4	mutS homolog 4 [Source:HGNC Sy	protein_coding	28,56929863	0,868069898	0,013655496	0,999657601
ENSG00000259663			antisense_RNA	2,626151698	-1,01432715	0,013672513	0,999657601
ENSG00000130783	CCDC62	coiled-coil domain containing 62 [S	protein_coding	57,42637916	0,476336139	0,013836582	0,999657601
ENSG00000238363	SNORA13	small nucleolar RNA, H/ACA box 13	snoRNA	70,90779767	0,5989784	0,01387021	0,999657601
ENSG00000200792	SNORA80A	small nucleolar RNA, H/ACA box 80	snoRNA	27,04081426	0,823787374	0,013963437	0,999657601
ENSG00000144369	FAM171B	family with sequence similarity 17:	protein_coding	12591,15291	-0,452007251	0,01399574	0,999657601
ENSG00000124610	HIST1H1A	histone cluster 1 H1 family membe	protein_coding	27,28064803	0,999699598	0,014067171	0,999657601
ENSG00000188523	CFAP77	cilia and flagella associated protein	protein_coding	61,15429006	-0,90254234	0,014113669	0,999657601
ENSG00000275084	SNORD91B	small nucleolar RNA, C/D box 91B		snoRNA	14,02244235	0,830971386	0,014152052	0,999657601
ENSG00000183346	CABCOCO1	ciliary associated calcium binding c	protein_coding	284,5822385	-0,802215037	0,014249959	0,999657601
ENSG00000152611	CAPSL	calcyphosine like [Source:HGNC Sy	protein_coding	27,27200982	-1,012974633	0,014338554	0,999657601
ENSG00000236114			unprocessed_pseudogene	15,16101325	-0,962744716	0,014352645	0,999657601
ENSG00000164344	KLKB1	kallikrein B1 [Source:HGNC Symbo	protein_coding	26,34180017	-0,817931435	0,014368089	0,999657601
ENSG00000231875			processed_pseudogene	4,526668861	-1,007952036	0,014379707	0,999657601
ENSG00000105974	CAV1	caveolin 1 [Source:HGNC Symbol;A	protein_coding	544,5521564	1,008956731	0,014591086	0,999657601
ENSG00000134020	PEBP4	phosphatidylethanolamine binding	protein_coding	8,423412799	-0,924604621	0,014596737	0,999657601
ENSG00000102096	PIM2	Pim-2 proto-oncogene, serine/thre	protein_coding	242,1410855	-0,534899108	0,014660471	0,999657601
ENSG00000185813	PCYT2	phosphate cytidylyltransferase 2, e	protein_coding	1498,110529	0,270640541	0,0146615	0,999657601
ENSG00000047365	ARAP2	ArfGAP with RhoGAP domain, anky	protein_coding	6759,672212	-0,572688186	0,014751055	0,999657601
ENSG00000126562	WNK4	WNK lysine deficient protein kinase	protein_coding	9,047656489	-0,90851589	0,01487881	0,999657601
ENSG00000084453	SLCO1A2	solute carrier organic anion transpo	protein_coding	98,26512152	0,979000078	0,014883396	0,999657601
ENSG00000162444	RBP7	retinol binding protein 7 [Source:H	protein_coding	3,927116635	-1,00424275	0,015046719	0,999657601
ENSG00000092607	TBX15	T-box 15 [Source:HGNC Symbol;Ac	protein_coding	129,5706173	-0,969073011	0,015108027	0,999657601
ENSG00000261371	PECAM1	platelet and endothelial cell adhesi	protein_coding	7,037621287	0,972378602	0,015256357	0,999657601
ENSG00000152818	UTRN	utrophin [Source:HGNC Symbol;Ac	protein_coding	3425,353473	0,511897221	0,015270883	0,999657601
ENSG00000081041	CXCL2	C-X-C motif chemokine ligand 2 [S	protein_coding	25,37343571	0,996362331	0,015424715	0,999657601
ENSG00000208892	SNORA49	small nucleolar RNA, H/ACA box 49	snoRNA	87,36515336	0,6027461	0,015520844	0,999657601
ENSG00000162836	ACP6	acid phosphatase 6, lysophosphati	protein_coding	419,0776715	-0,347645292	0,015558806	0,999657601
ENSG00000166819	PLIN1	perilipin 1 [Source:HGNC Symbol;A	protein_coding	8,807720131	-0,828058658	0,015750492	0,999657601
ENSG00000136859	ANGPTL2	angiopoietin like 2 [Source:HGNC S	protein_coding	479,7944398	0,726442674	0,01576426	0,999657601
ENSG00000131849	ZNF132	zinc finger protein 132 [Source:HG	protein_coding	115,1104732	0,994489844	0,015822443	0,999657601

Suppl Table 3_Differentially expressed genes in astrocytes among violent offenders versus healthy controls

ENSG00000169330	KIAA1024	KIAA1024 [Source:HGNC Symbol;A	protein_coding	56,16604066	0,550897841	0,01583552	0,999657601
ENSG00000264364	DYNLL2	dynein light chain LC8-type 2 [Sour	protein_coding	3369,202532	0,271477211	0,015842245	0,999657601
ENSG00000183684	ALYREF	Aly/REF export factor [Source:HGN	protein_coding	2706,833027	0,285060985	0,015981769	0,999657601
ENSG00000241074	RN7SL813P	RNA, 7SL, cytoplasmic 813, pseudo	misc_RNA	6,960080192	0,925868892	0,015999694	0,999657601
ENSG00000166816	LDHD	lactate dehydrogenase D [Source:H	protein_coding	42,96870601	-0,559023082	0,016070531	0,999657601
ENSG00000202408	RNU1-122P	RNA, U1 small nuclear 122, pseudo	snRNA	2,846854132	-0,994191891	0,016111071	0,999657601
ENSG00000272447			lincRNA	234,2302044	-0,530602096	0,016162695	0,999657601
ENSG00000175544	CABP4	calcium binding protein 4 [Source:I	protein_coding	3,620739629	-0,993012892	0,016290763	0,999657601
ENSG00000259275			lincRNA	15,08976649	0,986555018	0,016295483	0,999657601
ENSG00000119283	TRIM67	tripartite motif containing 67 [Sour	protein_coding	28,42229158	0,98981587	0,016554683	0,999657601
ENSG00000277453			lincRNA	11,39393806	-0,796427993	0,016644046	0,999657601
ENSG00000176601	MAP3K19	mitogen-activated protein kinase k	protein_coding	216,942817	-0,987401117	0,016816766	0,999657601
ENSG00000243905	RN7SL679P	RNA, 7SL, cytoplasmic 679, pseudo	misc_RNA	11,86208172	0,753219842	0,016830531	0,999657601
ENSG00000257038			antisense_RNA	19,40309894	0,779084369	0,016891158	0,999657601
ENSG00000253379			lincRNA	3,022372679	-0,987436408	0,01695947	0,999657601
ENSG00000135447	PPP1R1A	protein phosphatase 1 regulatory i	protein_coding	7,114092692	0,947148659	0,01704666	0,999657601
ENSG00000011422	PLAUR	plasminogen activator, urokinase r	protein_coding	43,33859143	0,759778221	0,017061703	0,999657601
ENSG00000114166	KAT2B	lysine acetyltransferase 2B [Source	protein_coding	1804,590417	-0,386613955	0,017134673	0,999657601
ENSG00000154265	ABCA5	ATP binding cassette subfamily A n	protein_coding	375,3283324	-0,971570608	0,017163184	0,999657601
ENSG00000187553	CYP26C1	cytochrome P450 family 26 subfan	protein_coding	4,646739707	-0,948761183	0,017188936	0,999657601
ENSG00000263711			processed_transcript	34,56526217	-0,763095024	0,017189006	0,999657601
ENSG00000188488	SERPINA5	serpin family A member 5 [Source:	protein_coding	5,390571031	0,963311034	0,017471184	0,999657601
ENSG00000138363	ATIC	5-aminoimidazole-4-carboxamide	protein_coding	252,350206	0,51568603	0,017571726	0,999657601
ENSG00000124243	BCAS4	breast carcinoma amplified sequer	protein_coding	171,5476505	-0,375050637	0,017671539	0,999657601
ENSG00000145365	TIFA	TRAF interacting protein with forkh	protein_coding	32,59478832	0,981855605	0,017682011	0,999657601
ENSG00000118307	CASC1	cancer susceptibility 1 [Source:HGI	protein_coding	156,9753468	-0,699491714	0,017696615	0,999657601
ENSG00000169752	NRG4	neuregulin 4 [Source:HGNC Symbo	protein_coding	11,29177802	-0,931040273	0,017827633	0,999657601
ENSG00000240583	AQP1	aquaporin 1 (Colton blood group) [	protein_coding	96,64233117	0,975413678	0,017874544	0,999657601
ENSG00000222489	SNORA79B	small nucleolar RNA, H/ACA box 79	snoRNA	196,577916	0,582934119	0,017877859	0,999657601
ENSG00000122778	KIAA1549	KIAA1549 [Source:HGNC Symbol;A	protein_coding	1155,287573	0,464175152	0,017894828	0,999657601
ENSG00000145075	CCDC39	coiled-coil domain containing 39 [S	protein_coding	57,17851002	-0,890352204	0,017970409	0,999657601
ENSG00000242083	RPL7AP31		processed_pseudogene	5,429729555	0,95636302	0,01797337	0,999657601
ENSG00000223573	TINCR	TINCR ubiquitin domain containing	protein_coding	58,59566002	0,976239379	0,018019318	0,999657601
ENSG00000221716	SNORA11	small nucleolar RNA, H/ACA box 1:	snoRNA	241,842086	0,584314808	0,018028764	0,999657601
ENSG00000111218	PRMT8	protein arginine methyltransferase	protein_coding	3,905338517	0,948993827	0,018082347	0,999657601
ENSG00000164669	INTS4P1	integrator complex subunit 4 pseu	transcribed_unprocessed_pseudogene	5,89481911	-0,973929801	0,018088242	0,999657601
ENSG00000230102	LINC02028	long intergenic non-protein coding	processed_transcript	4,601603878	-0,965266711	0,01809402	0,999657601
ENSG00000221638		Small nucleolar RNA U3 [Source:RF	snoRNA	3,062227919	0,976998419	0,018131259	0,999657601
ENSG00000145808	ADAMTS19	ADAM metallopeptidase with thro	protein_coding	8,639938438	-0,975671562	0,018250022	0,999657601
ENSG00000271858		cytochrome b561 family member I	antisense_RNA	3,261886205	-0,963003987	0,018271171	0,999657601
ENSG00000210154	MT-TD	mitochondrially encoded tRNA asp	Mt_tRNA	6,289129745	0,940257928	0,018295234	0,999657601
ENSG00000166922	SCG5	secretogranin V [Source:HGNC Syn	protein_coding	96,87132326	-0,783437798	0,018321138	0,999657601
ENSG00000156042	CFAP70	cilia and flagella associated protein	protein_coding	209,4653216	-0,756564148	0,018322248	0,999657601
ENSG00000225206	MIR137HG	MIR137 host gene [Source:HGNC S	lincRNA	33,88808286	-0,938750185	0,018347567	0,999657601

Suppl Table 3_Differentially expressed genes in astrocytes among violent offenders versus healthy controls

ENSG00000206535	LNP1	leukemia NUP98 fusion partner 1 [	protein_coding	181,2563328	0,581329813	0,018390426	0,999657601
ENSG00000258940			antisense_RNA	9,609947726	0,812730473	0,018391809	0,999657601
ENSG00000148908	RGS10	regulator of G protein signaling 10	protein_coding	68,25707218	-0,910379904	0,018400969	0,999657601
ENSG00000157856	DRC1	dynein regulatory complex subunit	protein_coding	484,2606112	-0,743257391	0,018426876	0,999657601
ENSG00000260281	ITFG1-AS1	ITFG1 antisense RNA 1 [Source:HG	antisense_RNA	2,738144741	0,96888813	0,018514658	0,999657601
ENSG00000141560	FN3KRP	fructosamine 3 kinase related prot	protein_coding	658,2028971	0,335623913	0,018643862	0,999657601
ENSG00000168646	AXIN2	axin 2 [Source:HGNC Symbol;Acc:H	protein_coding	483,2490044	0,697687048	0,018656677	0,999657601
ENSG00000204632	HLA-G	major histocompatibility complex,	protein_coding	205,1042275	-0,969627772	0,018749261	0,999657601
ENSG00000158023	WDR66	WD repeat domain 66 [Source:HGI	protein_coding	385,2471872	-0,736287754	0,018770302	0,999657601
ENSG00000275426			sense_intronic	8,847986851	-0,86999244	0,018826835	0,999657601
ENSG00000007174	DNAH9	dynein axonemal heavy chain 9 [Sc	protein_coding	265,8421996	-0,827458881	0,018874215	0,999657601
ENSG00000215067	ALOX12-AS1	ALOX12 antisense RNA 1 [Source:H	antisense_RNA	117,3000332	-0,340146164	0,018907	0,999657601
ENSG00000054356	PTPRN	protein tyrosine phosphatase, rece	protein_coding	89,5522402	-0,935192777	0,018964665	0,999657601
ENSG00000157766	ACAN	aggrecan [Source:HGNC Symbol;Ac	protein_coding	114,7543696	0,947392887	0,019007625	0,999657601
ENSG00000159592	GPBP1L1	GC-rich promoter binding protein 1	protein_coding	3150,341496	-0,152813224	0,019045621	0,999657601
ENSG00000272824			lincRNA	8,932058109	-0,887799177	0,019058774	0,999657601
ENSG00000186212	SOWAHB	sosondowah ankyrin repeat domai	protein_coding	12,29418433	0,958144658	0,019245409	0,999657601
ENSG00000160963	COL26A1	collagen type XXVI alpha 1 chain [S	protein_coding	2250,834453	0,960204906	0,019431666	0,999657601
ENSG00000219481	NBPF1	NBPF member 1 [Source:HGNC Syr	protein_coding	615,6686132	0,302724585	0,019488114	0,999657601
ENSG00000171303	KCNK3	potassium two pore domain chann	protein_coding	55,10753855	0,715593451	0,019558123	0,999657601
ENSG00000196535	MYO18A	myosin XVIIIA [Source:HGNC Symb	protein_coding	2494,040673	0,424288525	0,019609608	0,999657601
ENSG00000196510	ANAPC7	anaphase promoting complex subu	protein_coding	1570,093675	0,296353092	0,019685018	0,999657601
ENSG00000142867	BCL10	B-cell CLL/lymphoma 10 [Source:H	protein_coding	510,92793	0,191935567	0,019876241	0,999657601
ENSG00000232031			sense_intronic	5,019414021	0,923621845	0,019984952	0,999657601
ENSG00000248673	LINC01331	long intergenic non-protein coding	lincRNA	4,426792688	0,941879439	0,019994963	0,999657601
ENSG00000135697	BCO1	beta-carotene oxygenase 1 [Source	protein_coding	551,5951381	-0,850434576	0,020106033	0,999657601
ENSG00000092201	SUPT16H	SPT16 homolog, facilitates chroma	protein_coding	4859,11365	0,153603129	0,020130852	0,999657601
ENSG00000214784			processed_pseudogene	2,738071517	0,937683075	0,020251835	0,999657601
ENSG00000267284			lincRNA	6,21230871	0,873659898	0,020291877	0,999657601
ENSG00000185551	NR2F2	nuclear receptor subfamily 2 group	protein_coding	383,5808009	0,71001105	0,020403309	0,999657601
ENSG00000111834	RSPH4A	radial spoke head 4 homolog A [So	protein_coding	933,9651987	-0,790222507	0,020434013	0,999657601
ENSG00000154678	PDE1C	phosphodiesterase 1C [Source:HGI	protein_coding	1449,529058	0,93876016	0,020452176	0,999657601
ENSG00000196756	SNHG17	small nucleolar RNA host gene 17 [	processed_transcript	172,123613	0,389166522	0,020465569	0,999657601
ENSG00000055044	NOP58	NOP58 ribonucleoprotein [Source:	protein_coding	1153,240334	0,19563761	0,020498731	0,999657601
ENSG00000207392	SNORA20	small nucleolar RNA, H/ACA box 2(	snoRNA	205,6332135	0,575968091	0,020519695	0,999657601
ENSG00000125734	GPR108	G protein-coupled receptor 108 [S	protein_coding	1093,679845	-0,282639589	0,020561313	0,999657601
ENSG00000166886	NAB2	NGFI-A binding protein 2 [Source:H	protein_coding	1025,477904	-0,60383875	0,020627953	0,999657601
ENSG00000252712	SCARNA14	small Cajal body-specific RNA 14 [S	scaRNA	4,341180848	0,93041948	0,020722677	0,999657601
ENSG00000181856	SLC2A4	solute carrier family 2 member 4 [S	protein_coding	13,90138775	0,804996062	0,020765775	0,999657601
ENSG00000188596	CFAP54	cilia and flagella associated protein	protein_coding	1320,757094	-0,78071429	0,020769751	0,999657601
ENSG00000187186			protein_coding	8,504621921	-0,829192033	0,020797786	0,999657601
ENSG00000105519	CAPS	calcyphosine [Source:HGNC Symbo	protein_coding	709,0078815	-0,821474566	0,020804559	0,999657601
ENSG00000200816	SNORA38	small nucleolar RNA, H/ACA box 3{	snoRNA	162,7395124	0,682076266	0,0208238	0,999657601
ENSG00000155254	MARVELD1	MARVEL domain containing 1 [Sou	protein_coding	152,84978	0,863945556	0,020878671	0,999657601

Suppl Table 3_Differentially expressed genes in astrocytes among violent offenders versus healthy controls

ENSG00000241015	TPM3P9	tropomyosin 3 pseudogene 9 [Sour	transcribed_processed_pseudogene	202,3516902	0,527480838	0,020944881	0,999657601
ENSG00000128917	DLL4	delta like canonical Notch ligand 4	protein_coding	17,34861736	0,915364806	0,02096163	0,999657601
ENSG00000284624			processed_transcript	36,99916064	-0,811237834	0,021034835	0,999657601
ENSG00000269473			lincRNA	32,49115426	-0,580078908	0,021212229	0,999657601
ENSG00000207088	SNORA7B	small nucleolar RNA, H/ACA box 7E	snoRNA	30,45308389	0,603068996	0,021372596	0,999657601
ENSG00000116032	GRIN3B	glutamate ionotropic receptor NM	protein_coding	43,5065444	-0,849640274	0,021420189	0,999657601
ENSG00000196115	ADAM5	ADAM metallopeptidase domain 5	transcribed_unitary_pseudogene	10,59576749	-0,861234167	0,021553079	0,999657601
ENSG00000246130			antisense_RNA	4,691983139	0,934621069	0,021727995	0,999657601
ENSG00000170091	NSG2	neuronal vesicle trafficking associa	protein_coding	99,18163818	0,937945688	0,021781042	0,999657601
ENSG00000159079	C21orf59	chromosome 21 open reading fran	protein_coding	462,9207291	-0,322575235	0,021824049	0,999657601
ENSG00000106565	TMEM176B	transmembrane protein 176B [Sou	protein_coding	21,17053133	-0,938368395	0,021858349	0,999657601
ENSG00000069812	HES2	hes family bHLH transcription facto	protein_coding	65,91210772	0,928717107	0,021909024	0,999657601
ENSG00000100592	DAAM1	dishevelled associated activator of	protein_coding	1626,337506	0,345702994	0,021972118	0,999657601
ENSG00000233225			processed_pseudogene	21,21557767	0,843223707	0,022147123	0,999657601
ENSG00000280166			TEC	3,957922174	0,927604321	0,022158114	0,999657601
ENSG00000148735	PLEKHS1	pleckstrin homology domain conta	protein_coding	5,945472216	0,919163607	0,02220934	0,999657601
ENSG00000226742	HSBP1L1	heat shock factor binding protein 1	protein_coding	24,58422861	-0,804929134	0,022227968	0,999657601
ENSG00000272858			sense_intronic	5,147604555	-0,842964369	0,022252921	0,999657601
ENSG00000158109	TPRG1L	tumor protein p63 regulated 1 like	protein_coding	163,7610532	0,422775762	0,022365997	0,999657601
ENSG00000106799	TGFBR1	transforming growth factor beta re	protein_coding	3723,041802	0,440759218	0,02238444	0,999657601
ENSG00000135744	AGT	angiotensinogen [Source:HGNC Sy	protein_coding	1285,862897	0,875537514	0,02239633	0,999657601
ENSG00000200485		Y RNA [Source:RFAM;Acc:RF00019	misc_RNA	5,431822379	0,940815811	0,022569657	0,999657601
ENSG00000171962	DRC3	dynein regulatory complex subunit	protein_coding	314,8473343	-0,540930278	0,022805528	0,999657601
ENSG00000278540	ACACA	acetyl-CoA carboxylase alpha [Sou	protein_coding	2869,850584	0,214520735	0,022907048	0,999657601
ENSG00000253284			sense_intronic	44,55343527	-0,628600747	0,022933252	0,999657601
ENSG00000272862			lincRNA	4,315889622	-0,862539452	0,022966287	0,999657601
ENSG00000245864	MEF2C-AS2	MEF2C antisense RNA 2 [Source:H	antisense_RNA	18,65048197	0,84907994	0,023061913	0,999657601
ENSG00000107438	PDLIM1	PDZ and LIM domain 1 [Source:HG	protein_coding	446,0280541	0,837682487	0,023078994	0,999657601
ENSG00000027847	B4GALT7	beta-1,4-galactosyltransferase 7 [S	protein_coding	804,1408395	0,238967144	0,023107277	0,999657601
ENSG00000253223			processed_pseudogene	24,70937081	-0,831353896	0,023199627	0,999657601
ENSG00000178038	ALS2CL	ALS2 C-terminal like [Source:HGNC	protein_coding	52,51756899	0,937009889	0,023234662	0,999657601
ENSG00000265753	RN7SL444P	RNA, 7SL, cytoplasmic 444, pseudo	misc_RNA	21,63776988	0,889659895	0,023304529	0,999657601
ENSG00000133069	TMCC2	transmembrane and coiled-coil do	protein_coding	336,6838777	0,748462048	0,023338905	0,999657601
ENSG00000185250	PPIL6	peptidylprolyl isomerase like 6 [So	protein_coding	875,0743785	-0,683669434	0,023403467	0,999657601
ENSG00000166573	GALR1	galanin receptor 1 [Source:HGNC S	protein_coding	7,07373944	0,937881006	0,023442754	0,999657601
ENSG00000134864	GGACT	gamma-glutamylamine cyclotransf	protein_coding	64,65621738	-0,703915488	0,023542737	0,999657601
ENSG00000196482	ESRRG	estrogen related receptor gamma	protein_coding	126,9120736	-0,779495123	0,023567917	0,999657601
ENSG00000276417			lincRNA	10,62893438	-0,879479567	0,023576049	0,999657601
ENSG00000008441	NFIX	nuclear factor I X [Source:HGNC Sy	protein_coding	1762,588232	0,464929878	0,023605007	0,999657601
ENSG00000010626	LRRC23	leucine rich repeat containing 23 [S	protein_coding	620,2504882	-0,602914668	0,023702629	0,999657601
ENSG00000153037	SRP19	signal recognition particle 19 [Sour	protein_coding	270,6307725	0,237332784	0,023804147	0,999657601
ENSG00000185499	MUC1	mucin 1, cell surface associated [So	protein_coding	42,03434577	0,645381691	0,02381348	0,999657601
ENSG00000204583	LRCOL1	leucine rich colipase like 1 [Source:	protein_coding	7,277120336	-0,863537615	0,023830599	0,999657601
ENSG00000260643			protein_coding	37,4794479	-0,509105976	0,023833723	0,999657601

Suppl Table 3_Differentially expressed genes in astrocytes among violent offenders versus healthy controls

ENSG00000154258	ABCA9	ATP binding cassette subfamily A n	protein_coding	3,138259147	-0,929211873	0,023874506	0,999657601
ENSG00000114698	PLSCR4	phospholipid scramblase 4 [Source	protein_coding	803,8279559	-0,576166302	0,023914431	0,999657601
ENSG00000213250	RBMS2P1	RNA binding motif single stranded	processed_pseudogene	4,031436058	0,894403749	0,023999233	0,999657601
ENSG00000238490		Y RNA [Source:RFAM;Acc:RF00019	misc_RNA	3,577177975	0,914993942	0,024182389	0,999657601
ENSG00000143669	LYST	lysosomal trafficking regulator [Sou	protein_coding	1729,247005	-0,446313126	0,024189903	0,999657601
ENSG00000111252	SH2B3	SH2B adaptor protein 3 [Source:HG	protein_coding	201,8812591	0,448571491	0,024222822	0,999657601
ENSG00000267750	RUNDC3A-AS1	RUNDC3A antisense RNA 1 [Source	antisense_RNA	10,13290784	-0,741014698	0,024282847	0,999657601
ENSG00000088448	ANKRD10	ankyrin repeat domain 10 [Source:	protein_coding	3620,910026	-0,291722003	0,024298021	0,999657601
ENSG00000072422	RHOBTB1	Rho related BTB domain containing	protein_coding	1214,627105	-0,320098657	0,024329015	0,999657601
ENSG00000179133	C10orf67	chromosome 10 open reading fran	protein_coding	19,51621288	-0,782614971	0,024356768	0,999657601
ENSG00000174236	REP15	RAB15 effector protein [Source:HG	protein_coding	4,473149135	0,910552505	0,024367179	0,999657601
ENSG00000129691	ASH2L	ASH2 like histone lysine methyltran	protein_coding	453,425962	0,3576954	0,024478619	0,999657601
ENSG00000134245	WNT2B	Wnt family member 2B [Source:HG	protein_coding	96,87010306	0,871554503	0,024537061	0,999657601
ENSG00000250366	TUNAR	TCL1 upstream neural differentiati	protein_coding	7,043742355	0,900760569	0,024583766	0,999657601
ENSG00000206737	RNVU1-18	RNA, variant U1 small nuclear 18 [S	snRNA	14,81788884	0,67054702	0,024630104	0,999657601
ENSG00000205129	C4orf47	chromosome 4 open reading frame	protein_coding	122,0271879	-0,739853921	0,024653731	0,999657601
ENSG00000185201	IFITM2	interferon induced transmembrane	protein_coding	123,2627735	0,916360091	0,024661244	0,999657601
ENSG00000117122	MFAP2	microfibril associated protein 2 [So	protein_coding	45,36099524	0,905984627	0,024970707	0,999657601
ENSG00000100139	MICALL1	MICAL like 1 [Source:HGNC Symbo	protein_coding	2019,136204	0,505473677	0,024989257	0,999657601
ENSG00000254671	STT3A-AS1	STT3A antisense RNA 1 [Source:HG	antisense_RNA	3,977229907	0,87541627	0,025091779	0,999657601
ENSG00000228253	MT-ATP8	mitochondrially encoded ATP syntl	protein_coding	54,60215555	0,791465313	0,025163166	0,999657601
ENSG00000144649	FAM198A	family with sequence similarity 19	protein_coding	88,21096577	0,827991043	0,025378548	0,999657601
ENSG00000142949	PTPRF	protein tyrosine phosphatase, rece	protein_coding	3529,191959	0,365970297	0,025611914	0,999657601
ENSG00000280870		MIR325 host gene [Source:NCBI ge	lincRNA	48,01172757	0,627780967	0,025671192	0,999657601
ENSG00000100427	MLC1	megalencephalic leukoencephalop	protein_coding	21005,14843	-0,463871115	0,025694621	0,999657601
ENSG00000187627	RGPD1	RANBP2-like and GRIP domain con	protein_coding	7,678497556	-0,877113142	0,025801452	0,999657601
ENSG00000185507	IRF7	interferon regulatory factor 7 [Sou	protein_coding	800,5572487	-0,543540378	0,025866296	0,999657601
ENSG00000224094	RPS24P8	ribosomal protein S24 pseudogene	processed_pseudogene	4,370197672	-0,847236441	0,025910472	0,999657601
ENSG00000275367			lincRNA	64,96920752	-0,757338319	0,025927708	0,999657601
ENSG00000134769	DTNA	dystrobrevin alpha [Source:HGNC S	protein_coding	4430,559888	-0,325105088	0,025943569	0,999657601
ENSG00000225568			processed_pseudogene	4,160624677	-0,861667385	0,026013568	0,999657601
ENSG00000103257	SLC7A5	solute carrier family 7 member 5 [S	protein_coding	408,1637238	0,73533471	0,026103212	0,999657601
ENSG00000141337	ARSG	arylsulfatase G [Source:HGNC Sym	protein_coding	37,63081878	0,794679317	0,026118601	0,999657601
ENSG00000186973	FAM183A	family with sequence similarity 18	protein_coding	80,06142582	-0,844138308	0,026191499	0,999657601
ENSG00000139044	B4GALNT3	beta-1,4-N-acetyl-galactosaminyltr	protein_coding	686,0617171	-0,775803399	0,026523279	0,999657601
ENSG00000249921			processed_pseudogene	4,817921286	0,847421697	0,026609519	0,999657601
ENSG00000176771	NCKAP5	NCK associated protein 5 [Source:H	protein_coding	581,3776273	0,792136892	0,026642698	0,999657601
ENSG00000152705	CATSPER3	cation channel sperm associated 3	protein_coding	3,511440249	-0,881442612	0,026665573	0,999657601
ENSG00000131507	NDFIP1	Nedd4 family interacting protein 1	protein_coding	7167,992199	0,601827679	0,026901456	0,999657601
ENSG00000260742			antisense_RNA	10,19231094	-0,708488468	0,026924881	0,999657601
ENSG00000251188			processed_pseudogene	7,642779168	0,860168081	0,026937405	0,999657601
ENSG00000258548	LINC00645	long intergenic non-protein coding	lincRNA	19,92300156	-0,849743247	0,027059164	0,999657601
ENSG00000248527	MTATP6P1	mitochondrially encoded ATP syntl	unprocessed_pseudogene	160,0460064	0,813763256	0,027104919	0,999657601
ENSG00000104731	KLHDC4	kelch domain containing 4 [Source	protein_coding	539,481712	0,424936102	0,027187282	0,999657601

Suppl Table 3_Differentially expressed genes in astrocytes among violent offenders versus healthy controls

ENSG00000138356	AOX1	aldehyde oxidase 1 [Source:HGNC	protein_coding	41,61849045	-0,674946196	0,02722547	0,999657601
ENSG00000144031	ANKRD53	ankyrin repeat domain 53 [Source:	protein_coding	37,39405711	-0,837942169	0,027241453	0,999657601
ENSG00000008323	PLEKHG6	pleckstrin homology and RhoGEF d	protein_coding	5,282193763	-0,846148152	0,027417078	0,999657601
ENSG00000096401	CDC5L	cell division cycle 5 like [Source:HG	protein_coding	3358,986034	0,138379622	0,02748911	0,999657601
ENSG00000072786	STK10	serine/threonine kinase 10 [Source	protein_coding	565,7914136	0,611426791	0,027515764	0,999657601
ENSG00000086159	AQP6	aquaporin 6 [Source:HGNC Symbol	protein_coding	16,88821115	-0,785080551	0,027699537	0,999657601
ENSG00000270641	TSIX	TSIX transcript, XIST antisense RNA	lincRNA	2,865026321	-0,909872697	0,027802721	0,999657601
ENSG00000219642	BMPR1APS1	bone morphogenetic protein recep	processed_pseudogene	84,78848891	0,906699826	0,027856517	0,999657601
ENSG00000177989	ODF3B	outer dense fiber of sperm tails 3B	protein_coding	45,39629807	-0,890688675	0,027863647	0,999657601
ENSG00000253626	EIF5AL1	eukaryotic translation initiation fac	protein_coding	5,129558925	0,811280727	0,027898977	0,999657601
ENSG00000072121	ZFYVE26	zinc finger FYVE-type containing 26	protein_coding	2036,342466	-0,225873754	0,027965175	0,999657601
ENSG00000110880	CORO1C	coronin 1C [Source:HGNC Symbol;	protein_coding	2613,799917	0,415381662	0,02798229	0,999657601
ENSG00000206609	SNORD116-11	small nucleolar RNA, C/D box 116-	snoRNA	4,942117008	-0,825186969	0,027987314	0,999657601
ENSG00000240322	RN7SL481P	RNA, 7SL, cytoplasmic 481, pseudo	misc_RNA	7,533621579	0,715290838	0,027992778	0,999657601
ENSG00000149150	SLC43A1	solute carrier family 43 member 1	protein_coding	40,61573226	-0,746133089	0,027993418	0,999657601
ENSG00000151838	CCDC175	coiled-coil domain containing 175	protein_coding	45,92187021	-0,720076811	0,028014498	0,999657601
ENSG00000140416	TPM1	tropomyosin 1 [Source:HGNC Syml	protein_coding	6263,842385	0,87483816	0,028118992	0,999657601
ENSG00000082269	FAM135A	family with sequence similarity 13!	protein_coding	4578,707666	-0,458666177	0,028167108	0,999657601
ENSG00000104490	NCALD	neurocalcin delta [Source:HGNC Sy	protein_coding	4404,194844	0,538134701	0,028195189	0,999657601
ENSG00000106244	PDAP1	PDGFA associated protein 1 [Sourc	protein_coding	2730,476223	0,204438634	0,028310927	0,999657601
ENSG00000145721	LIX1	limb and CNS expressed 1 [Source:	protein_coding	1524,606226	0,748704007	0,028422237	0,999657601
ENSG00000144481	TRPM8	transient receptor potential cation	protein_coding	627,9253523	-0,85111226	0,028452494	0,999657601
ENSG00000066322	ELOVL1	ELOVL fatty acid elongase 1 [Sourc	protein_coding	986,6576755	-0,294255758	0,0284604	0,999657601
ENSG00000127423	AUNIP	aurora kinase A and ninein interact	protein_coding	111,0973856	0,485127511	0,028503351	0,999657601
ENSG00000174453	VWC2L	von Willebrand factor C domain co	protein_coding	12,38722821	0,654759085	0,028576724	0,999657601
ENSG00000157765	SLC34A2	solute carrier family 34 member 2	protein_coding	162,6060562	0,897116817	0,028580378	0,999657601
ENSG00000240509	RPL34P18	ribosomal protein L34 pseudogene	processed_pseudogene	19,11444323	0,718165186	0,028678651	0,999657601
ENSG00000250900			antisense_RNA	3,73027575	0,898292292	0,028794189	0,999657601
ENSG00000156535	CD109	CD109 molecule [Source:HGNC Syı	protein_coding	207,0515307	-0,831778494	0,028808746	0,999657601
ENSG00000206262	FOXL2NB	FOXL2 neighbor [Source:HGNC Syn	protein_coding	27,09741687	-0,891148589	0,028974156	0,999657601
ENSG00000173947	PIFO	primary cilia formation [Source:HG	protein_coding	797,1633105	-0,706738709	0,029010754	0,999657601
ENSG00000271860			lincRNA	30,62970285	0,831441726	0,02904098	0,999657601
ENSG00000141294	LRRC46	leucine rich repeat containing 46 [S	protein_coding	21,3531714	-0,798876645	0,02906839	0,999657601
ENSG00000179902	C1orf194	chromosome 1 open reading frame	protein_coding	60,89627503	-0,700537369	0,029088646	0,999657601
ENSG00000278948			TEC	482,5493447	0,65112731	0,029096037	0,999657601
ENSG00000164574	GALNT10	polypeptide N-acetylgalactosaminy	protein_coding	1192,833553	0,832838797	0,029235008	0,999657601
ENSG00000265142	MIR133A1HG	MIR133A1 host gene [Source:HGN	antisense_RNA	3,56865999	0,888193687	0,02927394	0,999657601
ENSG00000101311	FERMT1	fermitin family member 1 [Source:	protein_coding	29,59529978	0,901655574	0,029315992	0,999657601
ENSG00000172785	CBWD1	COBW domain containing 1 [Sourc	protein_coding	441,2822086	0,542359634	0,029331139	0,999657601
ENSG00000271254			protein_coding	391,2885364	0,404603971	0,029383493	0,999657601
ENSG00000225093	RPL3P7	ribosomal protein L3 pseudogene 7	processed_pseudogene	3,566705044	0,867966548	0,029390143	0,999657601
ENSG00000224114			processed_pseudogene	34,22284541	-0,882354313	0,029483811	0,999657601
ENSG00000189292	ALKAL2	ALK and LTK ligand 2 [Source:HGN	protein_coding	24,5701782	-0,852115927	0,029491856	0,999657601
ENSG00000146409	SLC18B1	solute carrier family 18 member B1	protein_coding	334,4712534	-0,588723437	0,029524588	0,999657601

Suppl Table 3_Differentially expressed genes in astrocytes among violent offenders versus healthy controls

ENSG00000214401	KANSL1-AS1	KANSL1 antisense RNA 1 [Source:H	antisense_RNA	31,4866631	0,756937878	0,029738833	0,999657601
ENSG00000183091	NEB	nebulin [Source:HGNC Symbol;Acc	protein_coding	319,7586773	-0,540475311	0,029812356	0,999657601
ENSG00000156253	RWDD2B	RWD domain containing 2B [Sourc	protein_coding	252,9631511	0,808957262	0,029816627	0,999657601
ENSG00000238365	RNU7-57P	RNA, U7 small nuclear 57 pseudog	snRNA	6,971620788	-0,81072875	0,029930165	0,999657601
ENSG00000166435	XRRA1	X-ray radiation resistance associate	protein_coding	243,4081923	-0,682086908	0,029984118	0,999657601
ENSG00000078018	MAP2	microtubule associated protein 2 [	protein_coding	20888,38604	0,423481932	0,029998597	0,999657601
ENSG00000234773			transcribed_unprocessed_pseudogene	158,2758672	-0,439422959	0,030095426	0,999657601
ENSG00000118492	ADGB	androglobin [Source:HGNC Symbo	protein_coding	3,453250485	-0,885771036	0,030143056	0,999657601
ENSG00000212607	SNORA3B	small nucleolar RNA, H/ACA box 3	snoRNA	129,9712068	0,499230531	0,030146284	0,999657601
ENSG00000177133	LINC00982	long intergenic non-protein coding	antisense_RNA	178,1470468	-0,683045465	0,030173449	0,999657601
ENSG00000142227	EMP3	epithelial membrane protein 3 [So	protein_coding	1234,726547	-0,45678294	0,030209065	0,999657601
ENSG00000149256	TENM4	teneurin transmembrane protein 4	protein_coding	1330,34836	0,722515753	0,030217793	0,999657601
ENSG00000140057	AK7	adenylate kinase 7 [Source:HGNC S	protein_coding	269,6687419	-0,86516482	0,03024927	0,999657601
ENSG00000049449	RCN1	reticulocalbin 1 [Source:HGNC Sym	protein_coding	3359,58624	0,546727592	0,030390042	0,999657601
ENSG00000180592	SKIDA1	SKI/DACH domain containing 1 [So	protein_coding	636,2752538	0,354712091	0,030398193	0,999657601
ENSG00000160007	ARHGAP35	Rho GTPase activating protein 35 [	protein_coding	4439,377586	0,191022282	0,03040033	0,999657601
ENSG00000138670	RASGEF1B	RasGEF domain family member 1B	protein_coding	517,4006143	-0,739203435	0,030466905	0,999657601
ENSG00000284526			protein_coding	4,17759222	0,895169732	0,030472073	0,999657601
ENSG00000247363			lincRNA	3,824438164	0,861881913	0,030472594	0,999657601
ENSG00000088727	KIF9	kinesin family member 9 [Source:H	protein_coding	358,9473344	-0,512713711	0,030506344	0,999657601
ENSG00000199751		Y RNA [Source:RFAM;Acc:RF00019	misc_RNA	17,95165586	0,791946662	0,030515432	0,999657601
ENSG00000152192	POU4F1	POU class 4 homeobox 1 [Source:H	protein_coding	23,01766083	0,874328162	0,030520816	0,999657601
ENSG00000267114			lincRNA	6,029407008	-0,78253152	0,030626369	0,999657601
ENSG00000272606			antisense_RNA	13,56439033	0,587414465	0,030834573	0,999657601
ENSG00000002746	HECW1	HECT, C2 and WW domain contain	protein_coding	106,8339701	0,745842312	0,030910884	0,999657601
ENSG00000271013	LRRC37A9P	leucine rich repeat containing 37 n	processed_pseudogene	5,78585505	0,891316028	0,031012538	0,999657601
ENSG00000185437	SH3BGR	SH3 domain binding glutamate rich	protein_coding	119,0833651	0,828349756	0,03104064	0,999657601
ENSG00000105971	CAV2	caveolin 2 [Source:HGNC Symbol;A	protein_coding	656,9407058	0,889292441	0,03113639	0,999657601
ENSG00000168785	TSPAN5	tetraspanin 5 [Source:HGNC Symb	protein_coding	3287,582033	-0,248731918	0,031153623	0,999657601
ENSG00000206199	ANKUB1	ankyrin repeat and ubiquitin doma	protein_coding	27,9748165	-0,862466607	0,031220348	0,999657601
ENSG00000089101	CFAP61	cilia and flagella associated protein	protein_coding	74,90638804	-0,708201338	0,03132893	0,999657601
ENSG00000283010			lincRNA	12,52634651	0,827390937	0,031466758	0,999657601
ENSG00000229007	EXOSC3P1	exosome component 3 pseudogen	processed_pseudogene	4,56702122	-0,839043795	0,031841652	0,999657601
ENSG00000201302	SNORA65	small nucleolar RNA, H/ACA box 6	snoRNA	85,24537242	0,386106596	0,031889243	0,999657601
ENSG00000206702	RNU1-11P	RNA, U1 small nuclear 11, pseudog	snRNA	44,08692477	0,641715057	0,031958292	0,999657601
ENSG00000076716	GPC4	glypican 4 [Source:HGNC Symbol;A	protein_coding	1327,559394	0,630652508	0,032260324	0,999657601
ENSG00000261210	CLEC19A	C-type lectin domain containing 19	protein_coding	891,509661	0,819948272	0,032402688	0,999657601
ENSG00000103365	GGA2	golgi associated, gamma adaptin e	protein_coding	695,0693995	0,500940056	0,032417437	0,999657601
ENSG00000257954			processed_pseudogene	4,543036266	-0,858883562	0,032615266	0,999657601
ENSG00000104518	GSDMD	gasdermin D [Source:HGNC Symbo	protein_coding	67,25525514	0,8685492	0,032666043	0,999657601
ENSG00000164746	C7orf57	chromosome 7 open reading fram	protein_coding	222,8155184	-0,860802515	0,032755655	0,999657601
ENSG00000183833	MAATS1	MYCBP associated and testis expre	protein_coding	432,7725367	-0,630047441	0,032756537	0,999657601
ENSG00000028528	SNX1	sorting nexin 1 [Source:HGNC Sym	protein_coding	1176,866689	0,238523068	0,03279849	0,999657601
ENSG00000187323	DCC	DCC netrin 1 receptor [Source:HGN	protein_coding	38,54560091	0,880468097	0,03288467	0,999657601

Suppl Table 3_Differentially expressed genes in astrocytes among violent offenders versus healthy controls

ENSG00000272690	LINC02018	long intergenic non-protein coding	lincRNA	15,65816441	0,567729331	0,032957823	0,999657601
ENSG00000162997	PRORSD1P	prolyl-tRNA synthetase associated	transcribed_unitary_pseudogene	14,19920039	-0,769480743	0,033343893	0,999657601
ENSG00000203546			protein_coding	9,254564951	0,718278282	0,033376026	0,999657601
ENSG00000180530	NRIP1	nuclear receptor interacting protei	protein_coding	3373,313422	-0,365465981	0,033457898	0,999657601
ENSG00000177508	IRX3	iroquois homeobox 3 [Source:HGN	protein_coding	67,84690094	0,798071886	0,033546448	0,999657601
ENSG00000152763	WDR78	WD repeat domain 78 [Source:HGI	protein_coding	1430,191881	-0,573722952	0,033738062	0,999657601
ENSG00000064763	FAR2	fatty acyl-CoA reductase 2 [Source	protein_coding	50,29851009	-0,875236813	0,033740209	0,999657601
ENSG00000111696	NT5DC3	5'-nucleotidase domain containing	protein_coding	352,9297752	0,492468729	0,033764899	0,999657601
ENSG00000157343	ARMC12	armadillo repeat containing 12 [So	protein_coding	40,26474815	-0,613097318	0,033836184	0,999657601
ENSG00000138758	SEPT11	septin 11 [Source:HGNC Symbol;Ac	protein_coding	16210,35488	0,558308254	0,033936147	0,999657601
ENSG00000185710	SMG1P4	SMG1 pseudogene 4 [Source:HGN	transcribed_unprocessed_pseudogene	7,114638274	0,839998538	0,033964111	0,999657601
ENSG00000254343			lincRNA	7,445518632	0,858259173	0,034019339	0,999657601
ENSG00000108641	B9D1	B9 domain containing 1 [Source:H(	protein_coding	292,6306297	-0,327664779	0,034138461	0,999657601
ENSG00000271167	LINC01109	long intergenic non-protein coding	lincRNA	34,98971089	0,764511263	0,034156647	0,999657601
ENSG00000265123	RN7SL200P	RNA, 7SL, cytoplasmic 200, pseudc	misc_RNA	8,804514516	0,668416984	0,034194639	0,999657601
ENSG00000240065	PSMB9	proteasome subunit beta 9 [Source	protein_coding	362,9697637	-0,493481106	0,034257012	0,999657601
ENSG00000140795	MYLK3	myosin light chain kinase 3 [Source	protein_coding	73,80244617	-0,760180495	0,034258275	0,999657601
ENSG00000119673	ACOT2	acyl-CoA thioesterase 2 [Source:H(	protein_coding	97,35876005	0,807422519	0,034291051	0,999657601
ENSG00000144278	GALNT13	polypeptide N-acetylgalactosaminy	protein_coding	144,3096344	-0,872968767	0,034362943	0,999657601
ENSG00000279733			TEC	4,173701945	-0,858509704	0,034372436	0,999657601
ENSG00000130638	ATXN10	ataxin 10 [Source:HGNC Symbol;Ac	protein_coding	1247,82122	0,400455885	0,034372684	0,999657601
ENSG00000226644			lincRNA	8,787141477	-0,843458999	0,03442967	0,999657601
ENSG00000105767	CADM4	cell adhesion molecule 4 [Source:H	protein_coding	4679,859764	0,324039698	0,034438346	0,999657601
ENSG00000162975	KCNF1	potassium voltage-gated channel r	protein_coding	331,0493722	-0,756430333	0,034586527	0,999657601
ENSG00000161149	TUBA3FP	tubulin alpha 3f pseudogene [Sour	processed_transcript	59,72529871	0,687801247	0,034603033	0,999657601
ENSG00000130844	ZNF331	zinc finger protein 331 [Source:HG	protein_coding	524,5601282	-0,302610397	0,034741178	0,999657601
ENSG00000185522	LMNTD2	lamin tail domain containing 2 [Sou	protein_coding	15,96945203	-0,727569466	0,034759283	0,999657601
ENSG00000244389	RN7SL242P	RNA, 7SL, cytoplasmic 242, pseudc	misc_RNA	8,650729368	0,778337861	0,034833928	0,999657601
ENSG00000224383	PRR29	proline rich 29 [Source:HGNC Syml	protein_coding	240,3039529	-0,733418434	0,034833933	0,999657601
ENSG00000155962	CLIC2	chloride intracellular channel 2 [So	protein_coding	100,1673354	-0,736974174	0,034973123	0,999657601
ENSG00000140832	MARVELD3	MARVEL domain containing 3 [Sou	protein_coding	405,8634011	-0,740650237	0,035007708	0,999657601
ENSG00000148737	TCF7L2	transcription factor 7 like 2 [Source	protein_coding	234,5942057	0,490898021	0,03502281	0,999657601
ENSG00000110697	PITPNM1	phosphatidylinositol transfer prote	protein_coding	1645,204098	-0,367758222	0,035042029	0,999657601
ENSG00000167371	PRRT2	proline rich transmembrane protei	protein_coding	404,9118367	-0,627305262	0,035065533	0,999657601
ENSG00000276101			antisense_RNA	5,520765969	0,778174613	0,03515722	0,999657601
ENSG00000160838	LRRC71	leucine rich repeat containing 71 [S	protein_coding	2,829524676	-0,840487736	0,035195664	0,999657601
ENSG00000156313	RPGR	retinitis pigmentosa GTPase regula	protein_coding	750,7112898	-0,541054643	0,03520756	0,999657601
ENSG00000162493	PDPN	podoplanin [Source:HGNC Symbol;	protein_coding	953,6970316	0,856667466	0,035303069	0,999657601
ENSG00000120156	TEK	TEK receptor tyrosine kinase [Sour	protein_coding	50,34069896	0,866781523	0,035352233	0,999657601
ENSG00000169093	ASMTL	acetylserotonin O-methyltransfera	protein_coding	593,6801913	0,237944291	0,035413062	0,999657601
ENSG00000234840	LINC01239	long intergenic non-protein coding	lincRNA	8,219468367	0,845390103	0,035485344	0,999657601
ENSG00000135205	CCDC146	coiled-coil domain containing 146	protein_coding	580,8755962	-0,580803992	0,035528744	0,999657601
ENSG00000186710	CFAP73	cilia and flagella associated protein	protein_coding	95,77113008	-0,809547112	0,035663563	0,999657601
ENSG00000100979	PLTP	phospholipid transfer protein [Sou	protein_coding	7230,788534	-0,407982597	0,035783844	0,999657601

Suppl Table 3_Differentially expressed genes in astrocytes among violent offenders versus healthy controls

ENSG00000197646	PDCD1LG2	programmed cell death 1 ligand 2	protein_coding	7,981886336	0,868340552	0,035884767	0,999657601
ENSG00000253797	UTP14C	UTP14C, small subunit processome	protein_coding	1085,034876	0,242872249	0,035961625	0,999657601
ENSG00000158428	CATIP	ciliogenesis associated TTC17 inter	protein_coding	32,21071751	-0,808596423	0,036335167	0,999657601
ENSG00000120306	CYSTM1	cysteine rich transmembrane mod	protein_coding	629,9464096	-0,366345742	0,036414736	0,999657601
ENSG00000137473	TTC29	tetratricopeptide repeat domain 2	protein_coding	21,92203978	-0,865077262	0,036457446	0,999657601
ENSG00000106483	SFRP4	secreted frizzled related protein 4	protein_coding	3996,95721	-0,850408985	0,036462803	0,999657601
ENSG00000268089	GABRQ	gamma-aminobutyric acid type A r	protein_coding	886,8592683	0,832948913	0,036585277	0,999657601
ENSG00000259232			processed_pseudogene	12,29650424	-0,786910453	0,036687723	0,999657601
ENSG00000008083	JARID2	jumonji and AT-rich interaction do	protein_coding	1072,803598	0,447446876	0,036706207	0,999657601
ENSG00000007080	CCDC124	coiled-coil domain containing 124	protein_coding	680,1193462	0,275179355	0,036742438	0,999657601
ENSG00000144410	CPO	carboxypeptidase O [Source:HGNC	protein_coding	4,028287854	-0,862533327	0,036829168	0,999657601
ENSG00000234231			unprocessed_pseudogene	20,3854512	0,761318203	0,036869673	0,999657601
ENSG00000179151	EDC3	enhancer of mRNA decapping 3 [S	protein_coding	408,0070915	0,281954359	0,036885918	0,999657601
ENSG00000182109			antisense_RNA	90,94496385	-0,834488037	0,036888267	0,999657601
ENSG00000096093	EFHC1	EF-hand domain containing 1 [Sou	protein_coding	2051,402288	-0,453652936	0,036925416	0,999657601
ENSG00000104998	IL27RA	interleukin 27 receptor subunit alp	protein_coding	115,52462	-0,703790665	0,036932776	0,999657601
ENSG00000227001	NBPF2P	NBPF member 2, pseudogene [Sou	unprocessed_pseudogene	12,53294322	-0,786835946	0,037001669	0,999657601
ENSG00000168301	KCTD6	potassium channel tetramerizatior	protein_coding	400,1764378	-0,395974635	0,037061519	0,999657601
ENSG00000139055	ERP27	endoplasmic reticulum protein 27	protein_coding	109,1755436	-0,820409881	0,037138071	0,999657601
ENSG00000079974	RABL2B	RAB, member of RAS oncogene far	protein_coding	192,3144543	-0,309624203	0,03717792	0,999657601
ENSG00000251733	SCARNA8	small Cajal body-specific RNA 8 [Sc	scaRNA	115,6871205	0,501836344	0,037212618	0,999657601
ENSG00000174137	FAM53A	family with sequence similarity 53	protein_coding	16,23813237	-0,722234614	0,03749256	0,999657601
ENSG00000112977	DAP	death associated protein [Source:H	protein_coding	306,5402669	0,480268429	0,037497545	0,999657601
ENSG00000269895			lincRNA	12,08517225	0,802086972	0,037544512	0,999657601
ENSG00000113456	RAD1	RAD1 checkpoint DNA exonuclease	protein_coding	1176,842211	0,167146983	0,037604978	0,999657601
ENSG00000226913	BSN-AS2	BSN antisense RNA 2 (head to hea	lincRNA	16,87381491	-0,636396007	0,037612833	0,999657601
ENSG00000237892	KLF7-IT1	KLF7 intronic transcript 1 [Source:H	sense_intronic	10,4383903	0,747668067	0,037658336	0,999657601
ENSG00000144028	SNRNP200	small nuclear ribonucleoprotein U	protein_coding	6382,788069	0,266114335	0,037773532	0,999657601
ENSG00000111249	CUX2	cut like homeobox 2 [Source:HGN	protein_coding	22,33892442	0,854654332	0,038042133	0,999657601
ENSG00000214753	HNRNPUL2	heterogeneous nuclear ribonucleo	protein_coding	289,9748293	0,448985644	0,038159388	0,999657601
ENSG00000072501	SMC1A	structural maintenance of chromos	protein_coding	2369,437504	0,226700693	0,03819154	0,999657601
ENSG00000188931	CFAP126	cilia and flagella associated protein	protein_coding	216,0457441	-0,762331151	0,038222427	0,999657601
ENSG00000280076			TEC	3,258289689	-0,8562487	0,038267947	0,999657601
ENSG00000187726	DNAJB13	DnaJ heat shock protein family (Hs	protein_coding	33,68100406	-0,794964394	0,038357521	0,999657601
ENSG00000165055	METTL2B	methyltransferase like 2B [Source:	protein_coding	1399,759935	0,271453712	0,038541381	0,999657601
ENSG00000100379	KCTD17	potassium channel tetramerizatior	protein_coding	1362,591684	-0,303313854	0,038592928	0,999657601
ENSG00000197140	ADAM32	ADAM metallopeptidase domain 3	protein_coding	674,6343085	-0,282779176	0,038612844	0,999657601
ENSG00000168505	GBX2	gastrulation brain homeobox 2 [So	protein_coding	94,36256048	0,85561502	0,038636229	0,999657601
ENSG00000225299			antisense_RNA	4,883768292	-0,767381608	0,038734813	0,999657601
ENSG00000187714	SLC18A3	solute carrier family 18 member A	protein_coding	7,4660184	-0,852845901	0,038840229	0,999657601
ENSG00000122786	CALD1	caldesmon 1 [Source:HGNC Symbo	protein_coding	13798,50348	0,827096495	0,038845656	0,999657601
ENSG00000054179	ENTPD2	ectonucleoside triphosphate dipho	protein_coding	2,683728543	-0,841785337	0,038888119	0,999657601
ENSG00000144061	NPHP1	nephrocystin 1 [Source:HGNC Sym	protein_coding	777,6638694	-0,463833486	0,038899611	0,999657601
ENSG00000153291	SLC25A27	solute carrier family 25 member 2	protein_coding	128,0791979	-0,655838079	0,039002862	0,999657601

Suppl Table 3_Differentially expressed genes in astrocytes among violent offenders versus healthy controls

ENSG00000187258	NPSR1	neuropeptide S receptor 1 [Source	protein_coding	84,50657463	0,852458122	0,039047976	0,999657601
ENSG00000217512			processed_pseudogene	7,11136614	0,817750404	0,0391699	0,999657601
ENSG00000064199	SPA17	sperm autoantigenic protein 17 [So	protein_coding	577,140462	-0,451677594	0,039170702	0,999657601
ENSG00000198626	RYR2	ryanodine receptor 2 [Source:HGN	protein_coding	11,72616081	0,836897923	0,039266995	0,999657601
ENSG00000186897	C1QL4	complement C1q like 4 [Source:HG	protein_coding	477,0321321	0,852800289	0,039357131	0,999657601
ENSG00000008517	IL32	interleukin 32 [Source:HGNC Symb	protein_coding	4,64845251	0,750623586	0,039424899	0,999657601
ENSG00000008516	MMP25	matrix metallopeptidase 25 [Sourc	protein_coding	10,2611261	-0,846317358	0,039484264	0,999657601
ENSG00000277452	RN7SL473P	RNA, 7SL, cytoplasmic 473, pseudo	misc_RNA	12,28532674	0,670080777	0,039500177	0,999657601
ENSG00000232615			processed_pseudogene	10,18015699	0,708895586	0,039582624	0,999657601
ENSG00000108375	RNF43	ring finger protein 43 [Source:HGN	protein_coding	23,99222113	0,84831357	0,039625258	0,999657601
ENSG00000171914	TLN2	talin 2 [Source:HGNC Symbol;Acc:H	protein_coding	1497,461654	0,382705025	0,039652595	0,999657601
ENSG00000206808		Y RNA [Source:RFAM;Acc:RF00019	misc_RNA	13,66124925	0,725994209	0,039743784	0,999657601
ENSG00000270964			lincRNA	30,55998178	0,474631564	0,039787775	0,999657601
ENSG00000204520	MICA	MHC class I polypeptide-related se	protein_coding	119,2039509	-0,817779666	0,039908925	0,999657601
ENSG00000139714	MORN3	MORN repeat containing 3 [Source	protein_coding	189,9832528	-0,727048683	0,039987895	0,999657601
ENSG00000121064	SCPEP1	serine carboxypeptidase 1 [Source	protein_coding	403,5287355	-0,592940304	0,040024059	0,999657601
ENSG00000005302	MSL3	MSL complex subunit 3 [Source:HG	protein_coding	955,9666459	-0,193624833	0,040047682	0,999657601
ENSG00000104361	NIPAL2	NIPA like domain containing 2 [Sou	protein_coding	19,47820741	0,848530172	0,040173123	0,999657601
ENSG00000178623	GPR35	G protein-coupled receptor 35 [Sou	protein_coding	40,58951124	-0,688232823	0,040267289	0,999657601
ENSG00000262583			transcribed_unprocessed_pseudogene	9,294992093	-0,789742702	0,040326419	0,999657601
ENSG00000138668	HNRNPD	heterogeneous nuclear ribonucleo	protein_coding	1085,516997	0,447787155	0,040327773	0,999657601
ENSG00000096746	HNRNPH3	heterogeneous nuclear ribonucleo	protein_coding	784,013981	0,392864899	0,040478461	0,999657601
ENSG00000226970			processed_pseudogene	10,02164053	-0,644013342	0,040539615	0,999657601
ENSG00000216480			processed_pseudogene	6,101990149	-0,73221462	0,040682652	0,999657601
ENSG00000281207	SLFNL1-AS1	SLFNL1 antisense RNA 1 [Source:H	antisense_RNA	17,66937734	0,682496405	0,040712946	0,999657601
ENSG00000176903	PNMA1	PNMA family member 1 [Source:H	protein_coding	2956,069841	0,337965836	0,040753518	0,999657601
ENSG00000171956	FOXB1	forkhead box B1 [Source:HGNC Sy	protein_coding	209,9748984	0,807205059	0,040997352	0,999657601
ENSG00000201098	RNY1	RNA, Ro-associated Y1 [Source:HG	misc_RNA	165532,2953	0,530529656	0,04103033	0,999657601
ENSG00000007264	MATK	megakaryocyte-associated tyrosine	protein_coding	5,823544127	-0,825649357	0,041157737	0,999657601
ENSG00000139719	VPS33A	VPS33A, CORVET/HOPS core subu	protein_coding	460,9093422	0,184429698	0,041315298	0,999657601
ENSG00000270492			processed_pseudogene	2,765625626	0,840918444	0,041322053	0,999657601
ENSG00000100473	COCH	cochlin [Source:HGNC Symbol;Acc:	protein_coding	301,9746872	0,74571904	0,041515559	0,999657601
ENSG00000261537			sense_intronic	2,855361255	-0,836371563	0,041551305	0,999657601
ENSG00000202538	RNU4-2	RNA, U4 small nuclear 2 [Source:H	snRNA	18111,11726	0,382494244	0,041596781	0,999657601
ENSG00000112078	KCTD20	potassium channel tetramerizatior	protein_coding	4717,655538	0,146071821	0,041640199	0,999657601
ENSG00000270689	BUD13P1	BUD13 homolog pseudogene 1 [Sc	processed_pseudogene	3,345559299	-0,830444279	0,041911454	0,999657601
ENSG00000147588	PMP2	peripheral myelin protein 2 [Sourc	protein_coding	93879,10741	0,549437002	0,041920597	0,999657601
ENSG00000171533	MAP6	microtubule associated protein 6 [	protein_coding	4355,251432	-0,392841697	0,041927426	0,999657601
ENSG00000129991	TNNI3	troponin I3, cardiac type [Source:H	protein_coding	3,121293889	-0,826469451	0,041978177	0,999657601
ENSG00000177426	TGIF1	TGFB induced factor homeobox 1 [	protein_coding	1923,142863	-0,376425426	0,042018731	0,999657601
ENSG00000196187	TMEM63A	transmembrane protein 63A [Sour	protein_coding	863,9433451	-0,401045144	0,042020003	0,999657601
ENSG00000154263	ABCA10	ATP binding cassette subfamily A n	protein_coding	9,667360724	-0,838936926	0,042207749	0,999657601
ENSG00000168394	TAP1	transporter 1, ATP binding cassette	protein_coding	1264,061636	-0,28573921	0,042360497	0,999657601
ENSG00000181085	MAPK15	mitogen-activated protein kinase 1	protein_coding	634,2405826	-0,730765946	0,042442522	0,999657601

Suppl Table 3_Differentially expressed genes in astrocytes among violent offenders versus healthy controls

ENSG00000105810	CDK6	cyclin dependent kinase 6 [Source:	protein_coding	8194,869056	0,622256806	0,042498071	0,999657601
ENSG00000183963	SMTN	smoothelin [Source:HGNC Symbol;	protein_coding	311,3309102	0,823509582	0,042547212	0,999657601
ENSG00000235408	SNORA71B	small nucleolar RNA, H/ACA box 71	snoRNA	36,02200042	0,510910082	0,042624462	0,999657601
ENSG00000100714	MTHFD1	methylenetetrahydrofolate dehydr	protein_coding	1994,86544	0,220997533	0,042653698	0,999657601
ENSG00000259871			sense_intronic	85,68550375	0,824247714	0,042722274	0,999657601
ENSG00000126705	AHDC1	AT-hook DNA binding motif contai	protein_coding	335,0869721	0,434511032	0,042815895	0,999657601
ENSG00000158715	SLC45A3	solute carrier family 45 member 3	protein_coding	207,2640896	-0,74209885	0,042911055	0,999657601
ENSG00000105193	RPS16	ribosomal protein S16 [Source:HGI	protein_coding	2213,222719	0,301066305	0,042924632	0,999657601
ENSG00000130764	LRRC47	leucine rich repeat containing 47 [S	protein_coding	2107,522009	0,168056056	0,043045595	0,999657601
ENSG00000232450			transcribed_processed_pseudogene	39,77505697	-0,531319942	0,0430898	0,999657601
ENSG00000174514	MFSD4A	major facilitator superfamily doma	protein_coding	39,23873929	-0,595917232	0,043271954	0,999657601
ENSG00000260257			lincRNA	247,7997814	0,34454005	0,043373547	0,999657601
ENSG00000189212	DPY19L2P1	DPY19L2 pseudogene 1 [Source:HC	transcribed_unprocessed_pseudogene	14,30032966	-0,615433993	0,043496956	0,999657601
ENSG00000090006	LTBP4	latent transforming growth factor I	protein_coding	394,4155307	0,641833332	0,043542476	0,999657601
ENSG00000164023	SGMS2	sphingomyelin synthase 2 [Source:	protein_coding	159,0409414	-0,675656831	0,043556583	0,999657601
ENSG00000154134	ROBO3	roundabout guidance receptor 3 [S	protein_coding	779,4207678	-0,371521568	0,043585556	0,999657601
ENSG00000259846			lincRNA	6,608053428	-0,823587159	0,0435934	0,999657601
ENSG00000133265	HSPBP1	HSPA (Hsp70) binding protein 1 [So	protein_coding	970,4478828	-0,196882797	0,043637499	0,999657601
ENSG00000126091	ST3GAL3	ST3 beta-galactoside alpha-2,3-sial	protein_coding	620,6525281	0,22382641	0,043828345	0,999657601
ENSG00000228570	NUTM2E	NUT family member 2E [Source:HC	protein_coding	3,214043808	-0,744503923	0,043895193	0,999657601
ENSG00000174740	PABPC5	poly(A) binding protein cytoplasmi	protein_coding	196,5629008	0,497640161	0,043991635	0,999657601
ENSG00000199223		Y RNA [Source:RFAM;Acc:RF00019	misc_RNA	6,21190515	0,759780083	0,04408637	0,999657601
ENSG00000277483	RN7SL321P	RNA, 7SL, cytoplasmic 321, pseudo	misc_RNA	4,806539048	0,819450497	0,044326667	0,999657601
ENSG00000157796	WDR19	WD repeat domain 19 [Source:HGI	protein_coding	2175,904326	-0,335062506	0,044369317	0,999657601
ENSG00000011009	LYPLA2	lysophospholipase II [Source:HGNC	protein_coding	873,7210574	-0,341325958	0,044373342	0,999657601
ENSG00000108468	CBX1	chromobox 1 [Source:HGNC Symbo	protein_coding	4598,612459	0,157967576	0,044422787	0,999657601
ENSG00000186471	AKAP14	A-kinase anchoring protein 14 [Sou	protein_coding	50,8674022	-0,773675658	0,044440729	0,999657601
ENSG00000079335	CDC14A	cell division cycle 14A [Source:HGN	protein_coding	376,4966204	-0,360728297	0,044446806	0,999657601
ENSG00000164440	TXLNB	taxilin beta [Source:HGNC Symbol;	protein_coding	48,80761239	0,796629633	0,044448795	0,999657601
ENSG00000164619	BMPER	BMP binding endothelial regulator	protein_coding	76,0257248	0,810894064	0,044465689	0,999657601
ENSG00000275803	RN7SL736P	RNA, 7SL, cytoplasmic 736, pseudo	misc_RNA	50,62663171	0,578335112	0,044481936	0,999657601
ENSG00000232872	CTAGE3P	CTAGE family member 3, pseudoge	processed_pseudogene	3,28027953	-0,822162389	0,044538727	0,999657601
ENSG00000164808	SPIDR	scaffolding protein involved in DNA	protein_coding	1660,716598	0,150104043	0,044578375	0,999657601
ENSG00000126453	BCL2L12	BCL2 like 12 [Source:HGNC Symbol	protein_coding	50,46701512	-0,390052985	0,044608132	0,999657601
ENSG00000233956	BTF3P6	basic transcription factor 3 pseudo	processed_pseudogene	4,565796608	-0,80821327	0,044792529	0,999657601
ENSG00000185052	SLC24A3	solute carrier family 24 member 3	protein_coding	593,4514622	0,716772199	0,04483916	0,999657601
ENSG00000106789	CORO2A	coronin 2A [Source:HGNC Symbol;.	protein_coding	6,17329143	0,824974153	0,044911108	0,999657601
ENSG00000120948	TARDBP	TAR DNA binding protein [Source:H	protein_coding	513,5454052	0,409758578	0,045070191	0,999657601
ENSG00000136110	CNMD	chondromodulin [Source:HGNC Sy	protein_coding	162,3543087	0,829422096	0,045073101	0,999657601
ENSG00000280543	ASAP1-IT2	ASAP1 intronic transcript 2 [Source	sense_intronic	4,207198898	-0,79626939	0,045315571	0,999657601
ENSG00000214110	LDHAP4	lactate dehydrogenase A pseudoge	processed_pseudogene	10,86404381	-0,634052298	0,045352677	0,999657601
ENSG00000146085	MUT	methylmalonyl-CoA mutase [Sourc	protein_coding	688,5888161	0,301480346	0,045373581	0,999657601
ENSG00000168038	ULK4	unc-51 like kinase 4 [Source:HGNC	protein_coding	418,341737	-0,470049921	0,045481372	0,999657601
ENSG00000224769	MUC20P1	mucin 20, cell surface associated p	unprocessed_pseudogene	6,871014675	0,821111034	0,045487923	0,999657601

Suppl Table 3_Differentially expressed genes in astrocytes among violent offenders versus healthy controls

ENSG00000185163	DDX51	DEAD-box helicase 51 [Source:HGN	protein_coding	763,0945778	0,328768124	0,045571604	0,999657601
ENSG00000110318	CEP126	centrosomal protein 126 [Source:H	protein_coding	156,6185326	-0,628006109	0,04557629	0,999657601
ENSG00000131781	FMO5	flavin containing monooxygenase 5	protein_coding	22,48952969	-0,580298393	0,045598315	0,999657601
ENSG00000253854			antisense_RNA	13,09857841	-0,642695239	0,045680262	0,999657601
ENSG00000078369	GNB1	G protein subunit beta 1 [Source:H	protein_coding	7209,7749	0,363231759	0,045761378	0,999657601
ENSG00000135903	PAX3	paired box 3 [Source:HGNC Symbo	protein_coding	871,0743105	0,669324063	0,045775122	0,999657601
ENSG00000148175	STOM	stomatin [Source:HGNC Symbol;Ac	protein_coding	231,6137121	0,786771379	0,04578044	0,999657601
ENSG00000182179	UBA7	ubiquitin like modifier activating er	protein_coding	568,0781096	-0,274339272	0,045957948	0,999657601
ENSG00000135587	SMPD2	sphingomyelin phosphodiesterase	protein_coding	207,03641	-0,294735778	0,046042622	0,999657601
ENSG00000172687	ZNF738	zinc finger protein 738 [Source:HG	protein_coding	1238,277036	0,251734583	0,046106235	0,999657601
ENSG00000102904	TSNAXIP1	translin associated factor X interac	protein_coding	381,5528108	-0,42208331	0,046107949	0,999657601
ENSG00000135617	PRADC1	protease associated domain contai	protein_coding	359,9304592	0,235998338	0,046204867	0,999657601
ENSG00000279685	MAPT-IT1	MAPT intronic transcript 1 [Source	sense_intronic	5,757483464	0,819002498	0,046297928	0,999657601
ENSG00000124098	FAM210B	family with sequence similarity 21(	protein_coding	1653,579181	0,295611393	0,046320802	0,999657601
ENSG00000204228	HSD17B8	hydroxysteroid 17-beta dehydroge	protein_coding	178,8941138	-0,361191796	0,046326338	0,999657601
ENSG00000087338	GMCL1	germ cell-less, spermatogenesis as	protein_coding	545,5584021	0,257551073	0,046367825	0,999657601
ENSG00000279801			TEC	7,027815308	-0,803571974	0,046413261	0,999657601
ENSG00000178053	MLF1	myeloid leukemia factor 1 [Source:	protein_coding	2192,472678	-0,537862221	0,046516075	0,999657601
ENSG00000163071	SPATA18	spermatogenesis associated 18 [Sc	protein_coding	346,934774	-0,698678589	0,046546848	0,999657601
ENSG00000142794	NBPF3	NBPF member 3 [Source:HGNC Syr	protein_coding	207,408269	0,587832496	0,046567313	0,999657601
ENSG00000250410			antisense_RNA	4,361978151	-0,811869103	0,046582719	0,999657601
ENSG00000243150			antisense_RNA	3,116013143	-0,807366385	0,04658825	0,999657601
ENSG00000153391	INO80C	INO80 complex subunit C [Source:I	protein_coding	54,31946792	0,453214371	0,046726085	0,999657601
ENSG00000278861			TEC	6,245615655	-0,766293913	0,046730898	0,999657601
ENSG00000260368			sense_overlapping	15,04889316	-0,672168343	0,046744915	0,999657601
ENSG00000269793	ZIM2-AS1	ZIM2 antisense RNA 1 [Source:HGI	antisense_RNA	3,45605658	0,822535427	0,046772805	0,999657601
ENSG00000165084	C8orf34	chromosome 8 open reading frame	protein_coding	15,4421228	-0,818662513	0,046784804	0,999657601
ENSG00000230695			antisense_RNA	3,48234225	-0,821226595	0,046796891	0,999657601
ENSG00000207971	MIR125B1	microRNA 125b-1 [Source:HGNC S	miRNA	6,519386003	-0,773333109	0,046837414	0,999657601
ENSG00000169064	ZBBX	zinc finger B-box domain containin	protein_coding	334,5152582	-0,714496268	0,047017542	0,999657601
ENSG00000207426		Y RNA [Source:RFAM;Acc:RF00019	misc_RNA	28,24298965	0,655674096	0,04702877	0,999657601
ENSG00000167705	RILP	Rab interacting lysosomal protein [	protein_coding	94,96803354	-0,517756126	0,047037067	0,999657601
ENSG00000279837			TEC	11,38946691	-0,70347462	0,047074342	0,999657601
ENSG00000120332	TNN	tenascin N [Source:HGNC Symbol;A	protein_coding	5,271537218	0,816378128	0,047093707	0,999657601
ENSG00000129682	FGF13	fibroblast growth factor 13 [Source	protein_coding	485,0171385	-0,587520179	0,047110559	0,999657601
ENSG00000168918	INPP5D	inositol polyphosphate-5-phosphat	protein_coding	41,88407979	-0,702459481	0,047355553	0,999657601
ENSG00000126773	PCNX4	pecanex homolog 4 [Source:HGNC	protein_coding	2898,977829	-0,192735513	0,047460122	0,999657601
ENSG00000184635	ZNF93	zinc finger protein 93 [Source:HGN	protein_coding	507,3150715	0,256802452	0,047478632	0,999657601
ENSG00000204666			sense_overlapping	18,32041125	-0,754994869	0,047567467	0,999657601
ENSG00000241472	PTPRG-AS1	PTPRG antisense RNA 1 [Source:H(	processed_transcript	80,59801133	0,50524874	0,047603889	0,999657601
ENSG00000198700	IPO9	importin 9 [Source:HGNC Symbol;A	protein_coding	7254,331608	0,220584057	0,047625235	0,999657601
ENSG00000133943	DGLUCY	D-glutamate cyclase [Source:HGNC	protein_coding	806,3359504	-0,394793072	0,047631163	0,999657601
ENSG00000180353	HCLS1	hematopoietic cell-specific Lyn sub	protein_coding	6,902519954	-0,692030434	0,047655	0,999657601
ENSG00000172175	MALT1	MALT1 paracaspase [Source:HGNC	protein_coding	1850,089989	0,683466387	0,047680071	0,999657601

Suppl Table 3_Differentially expressed genes in astrocytes among violent offenders versus healthy controls

ENSG00000255468			antisense_RNA	164,3018076	0,535648394	0,047703214	0,999657601
ENSG00000180139	ACTA2-AS1	ACTA2 antisense RNA 1 [Source:H(	antisense_RNA	7,325175353	0,802413278	0,047707262	0,999657601
ENSG00000073331	ALPK1	alpha kinase 1 [Source:HGNC Symb	protein_coding	84,66107794	0,813467688	0,047739226	0,999657601
ENSG00000137266	SLC22A23	solute carrier family 22 member 2:	protein_coding	571,2487017	0,365337236	0,04774375	0,999657601
ENSG00000179456	ZBTB18	zinc finger and BTB domain contair	protein_coding	3148,49889	0,309672877	0,047799698	0,999657601
ENSG00000173559	NABP1	nucleic acid binding protein 1 [Sou	protein_coding	164,9535473	-0,802339724	0,04781096	0,999657601
ENSG00000246214			antisense_RNA	23,68426813	-0,627524268	0,047905452	0,999657601
ENSG00000154914	USP43	ubiquitin specific peptidase 43 [So	protein_coding	148,9509475	-0,734346529	0,047967935	0,999657601
ENSG00000079102	RUNX1T1	RUNX1 translocation partner 1 [So	protein_coding	62,00812237	0,775966053	0,047973581	0,999657601
ENSG00000157557	ETS2	ETS proto-oncogene 2, transcriptic	protein_coding	67,81089188	0,811735198	0,047988135	0,999657601
ENSG00000072041	SLC6A15	solute carrier family 6 member 15	protein_coding	8,094863845	0,811517801	0,048008964	0,999657601
ENSG00000196313	POM121	POM121 transmembrane nucleop(	protein_coding	228,9179644	0,291191411	0,048080874	0,999657601
ENSG00000147439	BIN3	bridging integrator 3 [Source:HGN(	protein_coding	640,0963182	-0,198143536	0,048191077	0,999657601
ENSG00000242265	PEG10	paternally expressed 10 [Source:H(	protein_coding	18327,53976	-0,589859524	0,048265393	0,999657601
ENSG00000176428	VPS37D	VPS37D, ESCRT-I subunit [Source:H	protein_coding	316,6286389	-0,33247615	0,048374458	0,999657601
ENSG00000205959			lincRNA	15,30670175	0,581863651	0,048477998	0,999657601
ENSG00000271907	SNORA35B	small nucleolar RNA, H/ACA box 3!	snoRNA	53,35637056	0,611065095	0,048635319	0,999657601
ENSG00000157429	ZNF19	zinc finger protein 19 [Source:HGN	protein_coding	205,0593062	-0,326211286	0,048640149	0,999657601
ENSG00000226102	SEPT7P3	septin 7 pseudogene 3 [Source:HG	unprocessed_pseudogene	21,44823916	-0,719728496	0,04869714	0,999657601
ENSG00000188641	DPYD	dihydropyrimidine dehydrogenase	protein_coding	763,0727018	-0,809122372	0,048772378	0,999657601
ENSG00000271996			antisense_RNA	3,4531053	0,811635813	0,04885591	0,999657601
ENSG00000154309	DISP1	dispatched RND transporter family	protein_coding	191,1353619	0,626998001	0,048882325	0,999657601
ENSG00000114742	WDR48	WD repeat domain 48 [Source:HGI	protein_coding	2419,062823	0,170472163	0,049125033	0,999657601
ENSG00000188739	RBM34	RNA binding motif protein 34 [Sou	protein_coding	42,10847961	0,395836216	0,049190725	0,999657601
ENSG00000156140	ADAMTS3	ADAM metallopeptidase with thro	protein_coding	3241,892481	-0,391790086	0,049203964	0,999657601
ENSG00000161040	FBXL13	F-box and leucine rich repeat prote	protein_coding	140,7484057	-0,463037704	0,049312861	0,999657601
ENSG00000207133	SNORD116-7	small nucleolar RNA, C/D box 116-7	snoRNA	35,65389887	-0,614863611	0,049363173	0,999657601
ENSG00000135469	COQ10A	coenzyme Q10A [Source:HGNC Syr	protein_coding	225,3243345	-0,299791092	0,049367577	0,999657601
ENSG00000143867	OSR1	odd-skipped related transciption fa	protein_coding	136,7677963	0,812791953	0,049389786	0,999657601
ENSG00000084710	EFR3B	EFR3 homolog B [Source:HGNC Syr	protein_coding	2675,598496	0,533518545	0,049418031	0,999657601
ENSG00000152078	TMEM56	transmembrane protein 56 [Sourc(	protein_coding	86,77894641	-0,518966593	0,049467287	0,999657601
ENSG00000079263	SP140	SP140 nuclear body protein [Sourc	protein_coding	11,64364967	-0,675714058	0,049705594	0,999657601
ENSG00000197483	ZNF628	zinc finger protein 628 [Source:HG	protein_coding	91,58547437	0,553393354	0,049734419	0,999657601
ENSG00000235720	GABPAP	GA binding protein transcription fa	processed_pseudogene	2,753194518	0,809643152	0,049777206	0,999657601
ENSG00000011295	TTC19	tetratricopeptide repeat domain 1!	protein_coding	2699,762496	0,273311701	0,049962522	0,999657601
ENSG00000164687	FABP5	fatty acid binding protein 5 [Sourc(	protein_coding	800,526876	-0,75764632	0,049973761	0,999657601
ENSG00000142609	CFAP74	cilia and flagella associated proteir	protein_coding	63,68065549	-0,770362776	0,049994825	0,999657601
ENSG00000238249	HMGN2P17	high mobility group nucleosomal b	processed_pseudogene	2,83953086	-0,807443739	0,050014359	0,999657601
ENSG00000152932	RAB3C	RAB3C, member RAS oncogene far	protein_coding	79,54779149	0,779002556	0,050037678	0,999657601
ENSG00000247121			antisense_RNA	20,62283224	0,561413101	0,050110478	0,999657601
ENSG00000233716			processed_pseudogene	4,74740092	-0,741762395	0,050123655	0,999657601
ENSG00000273338			antisense_RNA	9,21948406	0,745277002	0,050172432	0,999657601
ENSG00000111885	MAN1A1	mannosidase alpha class 1A memb	protein_coding	1401,477327	-0,63086859	0,050229064	0,999657601
ENSG00000171551	ECEL1	endothelin converting enzyme like	protein_coding	374,3261543	-0,766311226	0,050347814	0,999657601

Suppl Table 3_Differentially expressed genes in astrocytes among violent offenders versus healthy controls

ENSG00000187391	MAGI2	membrane associated guanylate ki	protein_coding	877,7433477	-0,448285018	0,050430304	0,999657601
ENSG00000275004	ZNF280B	zinc finger protein 280B [Source:H(	protein_coding	227,1026632	0,514960066	0,050437742	0,999657601
ENSG00000144451	SPAG16	sperm associated antigen 16 [Sour	protein_coding	1147,191796	-0,283754627	0,050443036	0,999657601
