# Supplementary material for: Neurobiological roots of psychopathy
Source: Mol Psychiatry. 2019 Aug 27;25(12):3432–41. doi: 10.1038/s41380-019-0488-z (PMC7714686; doi:10.1038/s41380-019-0488-z)
Supplement: Supplementary file 4 — Supplementary Table 4 [file 41380_2019_488_MOESM4_ESM.pdf]

Suppl Table 4_Gene set enrichment and pathway analysis_10.4.2019

Comparison number	Comparison name	Number of DEGs	Over-representation analysis (using		Gene set enrichment analysis (using all genes)		
			No of enriched GOBPs	No of enriched KEGGs	No of enriched GOBPs	No of enriched KEGGs	No of enriched REACTOME terms
1	Violent vs Control + Non-Violent	34	No significantly enriched	No significantly enriched	29	8	6
2	Violent vs Control	4	No significantly enriched	No significantly enriched	*	*	*
3	Non-Violent vs Control	4	No significantly enriched	No significantly enriched	165	21	27
4	Violent vs Non-Violent	28	No significantly enriched	No significantly enriched	34	7	9

*)Could not be produced because of an internal error. For unidentified reason the test would not run for the gene list.

NEURONS

violent vs. control + non-violent

GOBPs

Term ID	Term Description	Gene Set Size	Normalized Enrichment Score (NES)	P-value	Adjusted P-value
GO:0001817	regulation of cytokine production	485	-1,2110	0,0002	0,039
GO:0002250	adaptive immune response	268	-1,3260	0,0002	0,039
GO:0002253	activation of immune response	435	-1,2508	0,0002	0,039
GO:0002694	regulation of leukocyte activation	371	-1,2191	0,0002	0,039
GO:0002697	regulation of immune effector process	275	-1,2673	0,0002	0,039
GO:0002757	immune response-activating signal transduction	387	-1,2422	0,0002	0,039
GO:0002764	immune response-regulating signaling pathway	419	-1,2670	0,0002	0,039
GO:0002768	immune response-regulating cell surface receptor signaling pathway	304	-1,2877	0,0002	0,039
GO:0009617	response to bacterium	369	-1,2131	0,0002	0,039
GO:0019221	cytokine-mediated signaling pathway	450	-1,2314	0,0002	0,039
GO:0050865	regulation of cell activation	404	-1,2141	0,0002	0,039
GO:0050900	leukocyte migration	299	-1,2719	0,0002	0,039
GO:0051606	detection of stimulus	311	-1,3092	0,0002	0,039
GO:1901615	organic hydroxy compound metabolic process	383	-1,2354	0,0002	0,039
GO:0002443	leukocyte mediated immunity	235	-1,2774	0,0002	0,039
GO:0007606	sensory perception of chemical stimulus	169	-1,3329	0,0002	0,039
GO:0002460	adaptive immune response based on somatic recombination of immune receptors built from immunoglobulin superfamily domains	178	-1,2706	0,0002	0,039
GO:0009593	detection of chemical stimulus	157	-1,3543	0,0002	0,039
GO:0050906	detection of stimulus involved in sensory perception	171	-1,3517	0,0002	0,039

GO:0098656	anion transmembrane transport	182	-1,2671	0,0002	0,039
GO:1901617	organic hydroxy compound biosynthetic process	157	-1,2999	0,0002	0,039
GO:0015698	inorganic anion transport	139	-1,3098	0,0002	0,039
GO:0009566	fertilization	137	-1,3546	0,0002	0,039
GO:0050907	detection of chemical stimulus involved in sensory perception	124	-1,3673	0,0002	0,039
GO:0007608	sensory perception of smell	115	-1,4028	0,0002	0,039
GO:0050911	detection of chemical stimulus involved in sensory perception of smell	90	-1,4285	0,0002	0,039
GO:0009988	cell-cell recognition	54	-1,4257	0,0002	0,039
GO:0030049	muscle filament sliding	36	-1,4492	0,0002	0,039
GO:0033275	actin-myosin filament sliding	36	-1,4492	0,0002	0,039

KEGGs

Term ID	Term Description	Gene Set Size	Normalized Enrichment Score (NES)	P-value	Adjusted P-value
hsa04740	Olfactory transduction	127	-1,4154	0,0002	0,031
hsa05152	Tuberculosis	153	-1,3135	0,0002	0,031
hsa04060	Cytokine-cytokine receptor interaction	179	-1,2987	0,0004	0,032
hsa02010	ABC transporters	44	-1,4406	0,0004	0,032
hsa04650	Natural killer cell mediated cytotoxicity	95	-1,3552	0,0006	0,036
hsa04380	Osteoclast differentiation	109	-1,3290	0,0008	0,036
hsa04610	Complement and coagulation cascades	65	-1,3718	0,0008	0,036
hsa00190	Oxidative phosphorylation	128	-1,2876	0,0012	0,047

REACTOME

Term ID	Term Description	Gene Set Size	Normalized Enrichment Score (NES)	P-value	Adjusted P-value
388396	GPCR downstream signaling	486	-1,2028	0,0002	0,006
168249	Innate Immune System	499	-1,2041	0,0002	0,006
382551	Transmembrane transport of small molecules	497	-1,1993	0,0002	0,006
1428517	The citric acid (TCA) cycle and respiratory electron transport	136	-1,3285	0,0006	0,013
1280218	Adaptive Immune System	395	-1,1864	0,0012	0,020
1280215	Cytokine Signaling in Immune system	247	-1,2084	0,0034	0,048
